# Supplementary figures and images for: BMP4 initiates and patterns ventral-caudal structures in zebrafish and human pluripotent stem cell aggregates (part 1 of 2)
Source: EMBO J. 2025 Nov 24;45(1):210–42. doi: 10.1038/s44318-025-00643-6 (PMC12759085; doi:10.1038/s44318-025-00643-6)

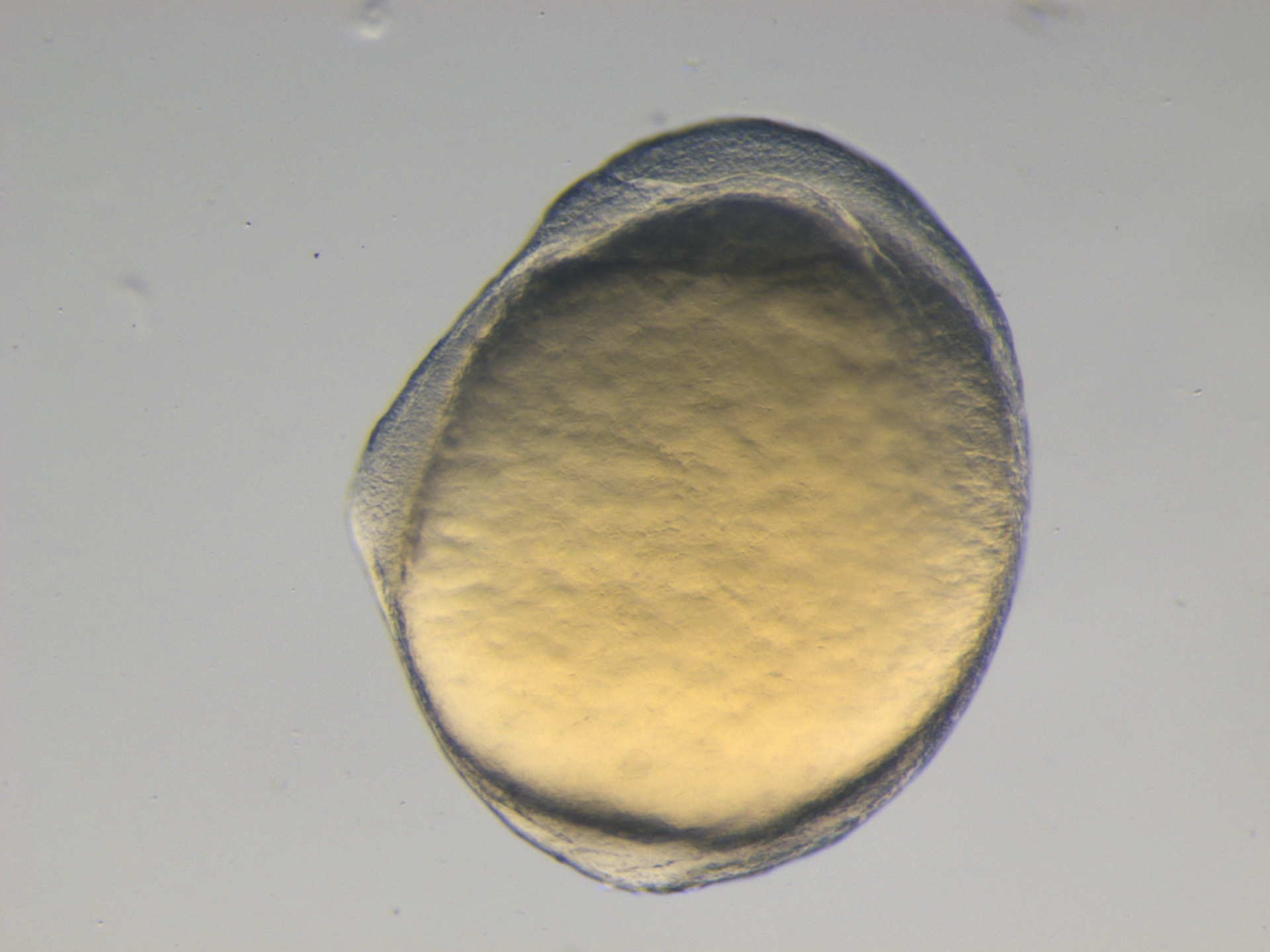

Supplement: Supplementary file 17 — Source data Fig. 1 [file 44318_2025_643_MOESM17_ESM.zip › Figure 1/1A/embryo_10 hpf_bright field.tif]

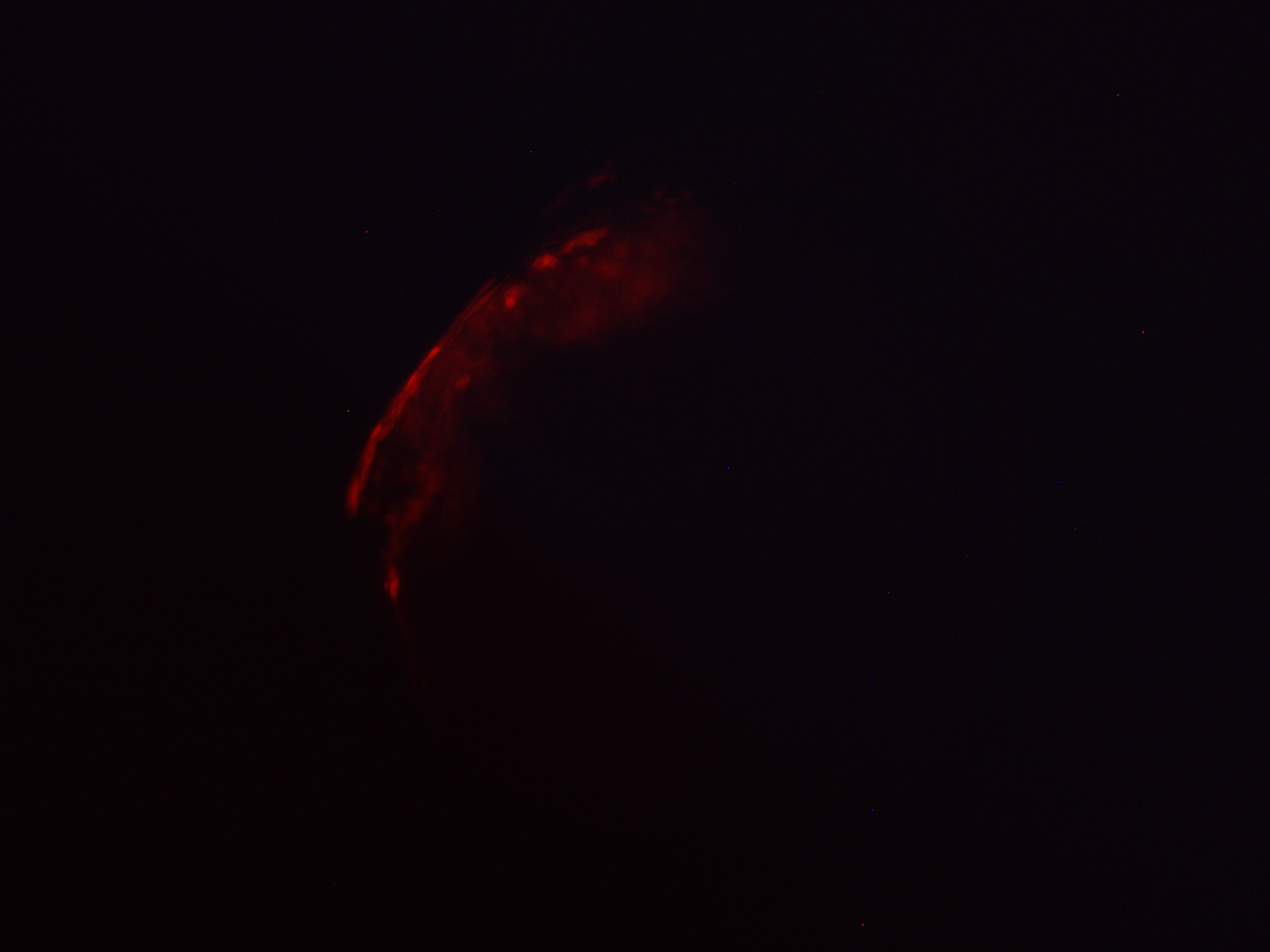

Supplement: Supplementary file 17 — Source data Fig. 1 [file 44318_2025_643_MOESM17_ESM.zip › Figure 1/1A/embryo_10 hpf_red fluorescence.tif]

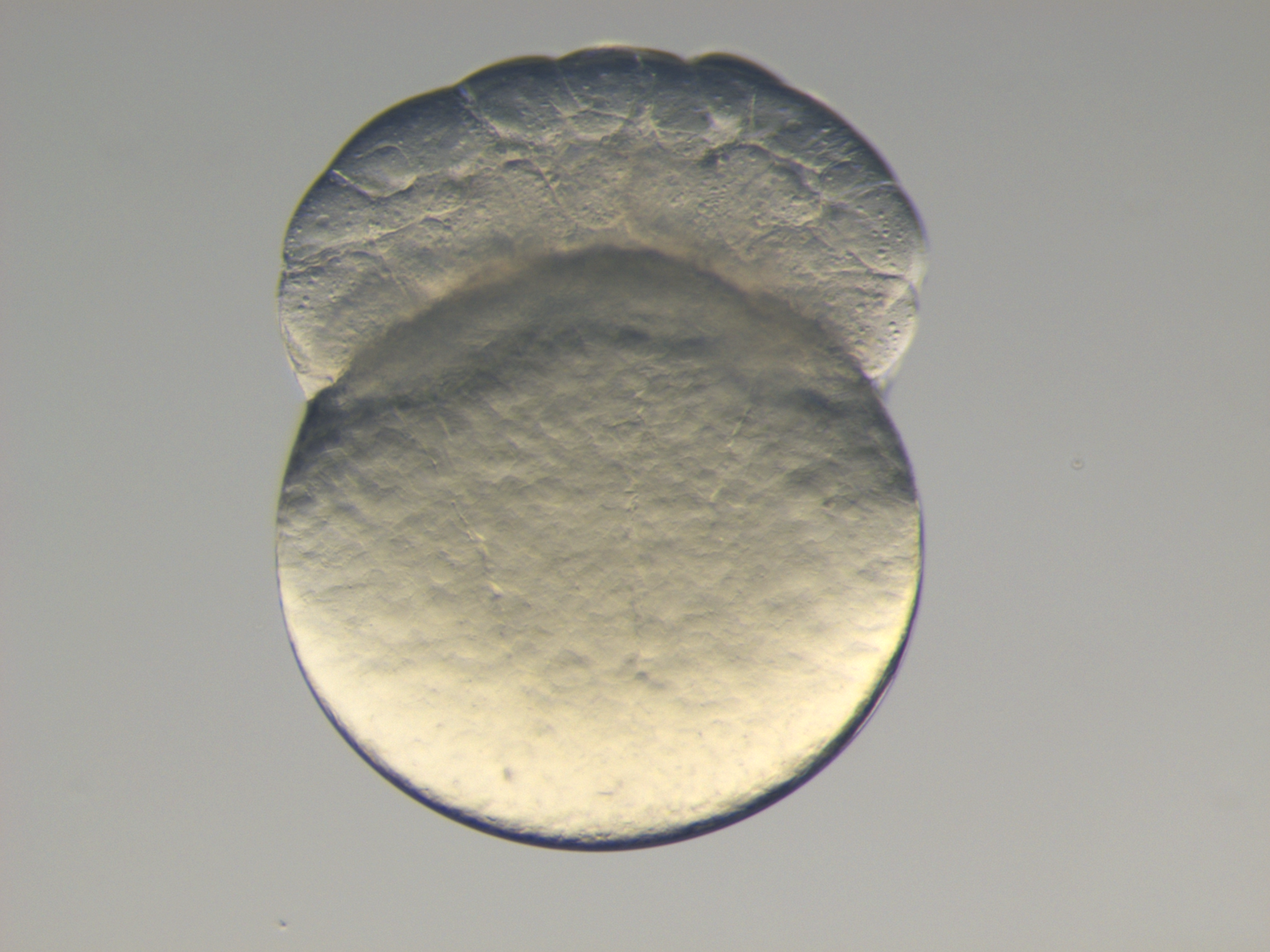

Supplement: Supplementary file 17 — Source data Fig. 1 [file 44318_2025_643_MOESM17_ESM.zip › Figure 1/1A/embryo_2.25 hpf_bright field.tif]

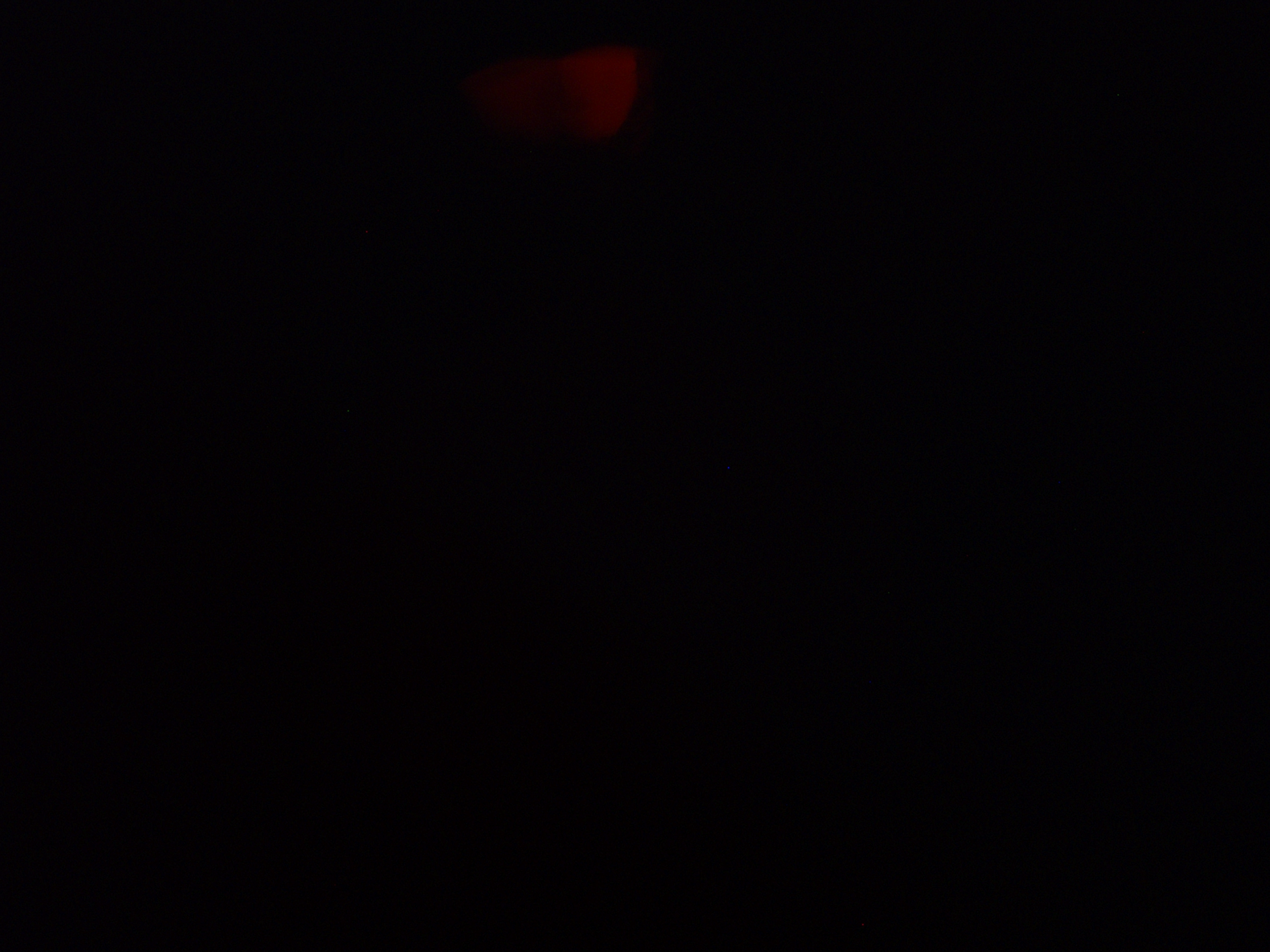

Supplement: Supplementary file 17 — Source data Fig. 1 [file 44318_2025_643_MOESM17_ESM.zip › Figure 1/1A/embryo_2.25 hpf_red fluorescence.tif]

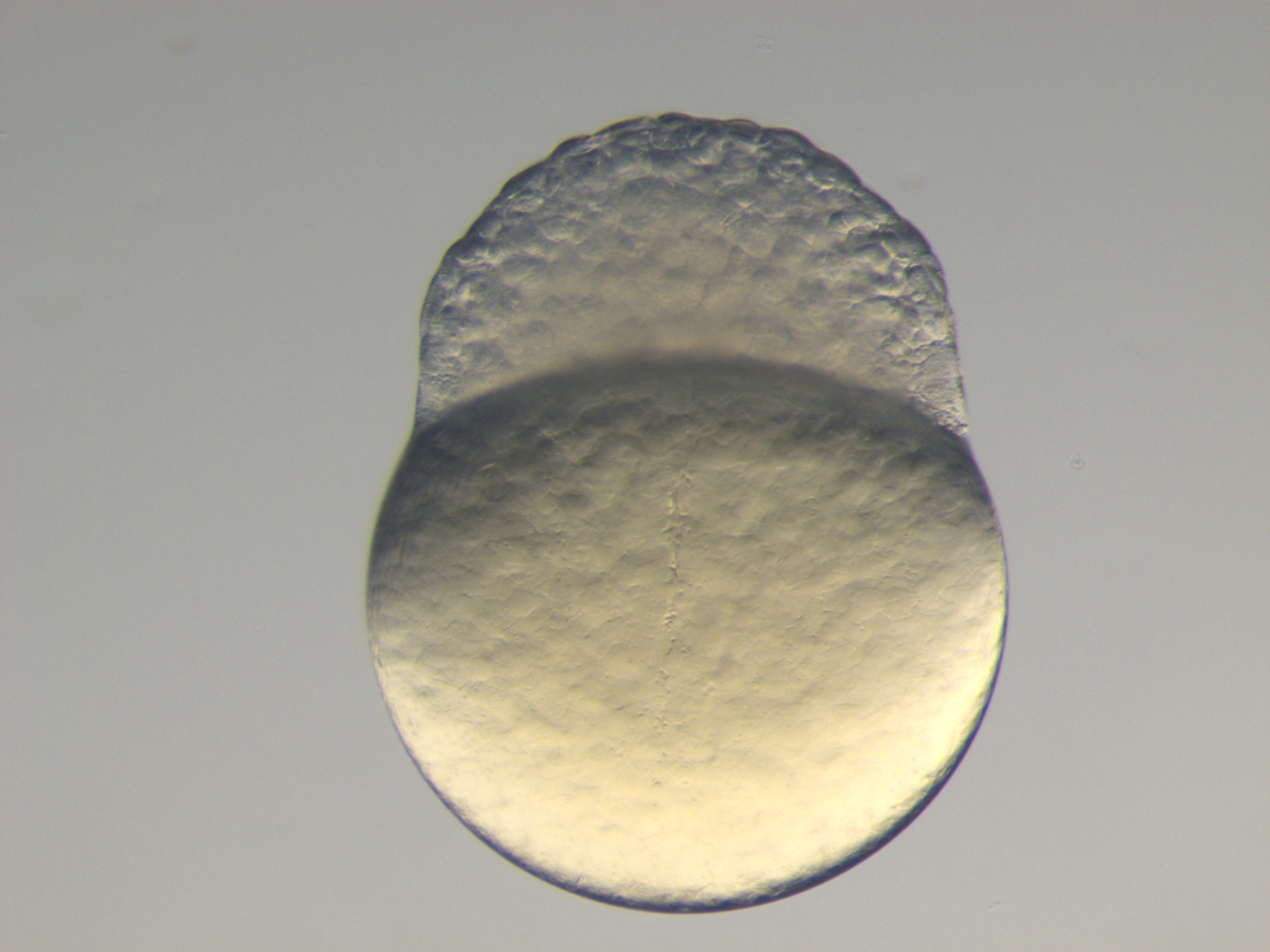

Supplement: Supplementary file 17 — Source data Fig. 1 [file 44318_2025_643_MOESM17_ESM.zip › Figure 1/1A/embryo_2.75 hpf_bright field.tif]

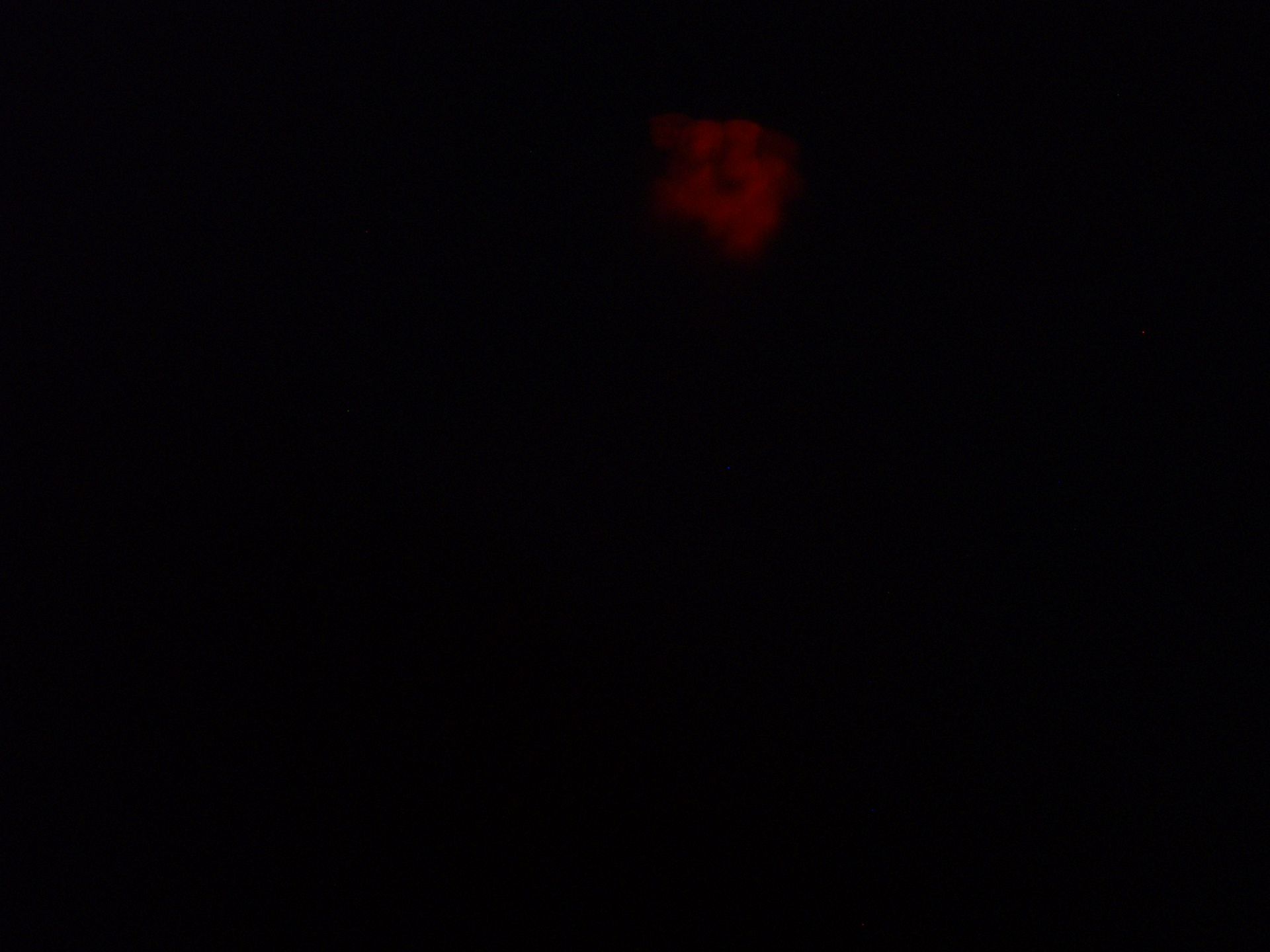

Supplement: Supplementary file 17 — Source data Fig. 1 [file 44318_2025_643_MOESM17_ESM.zip › Figure 1/1A/embryo_2.75 hpf_red fluorescence.tif]

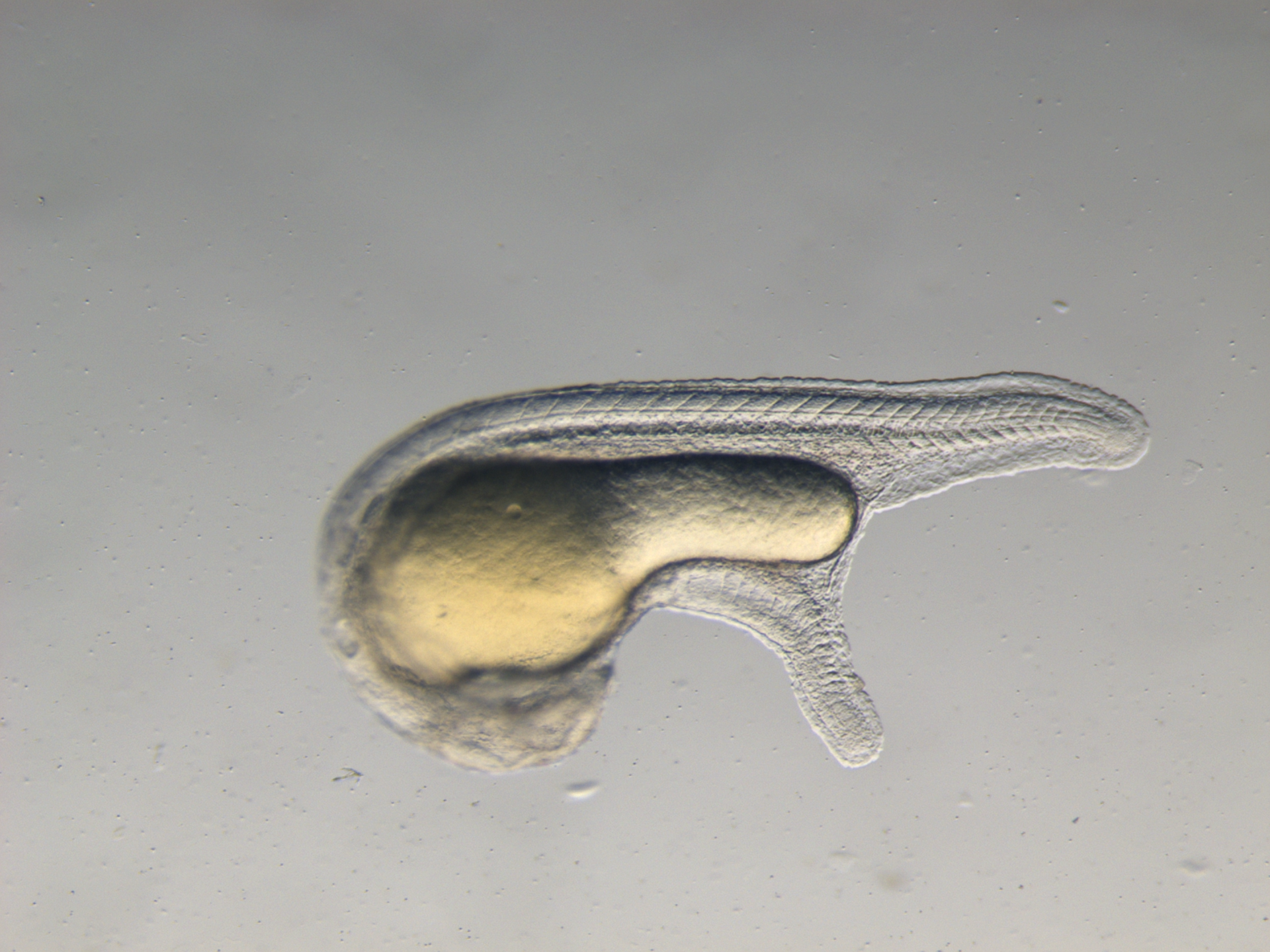

Supplement: Supplementary file 17 — Source data Fig. 1 [file 44318_2025_643_MOESM17_ESM.zip › Figure 1/1A/embryo_24 hpf_bright field.tif]

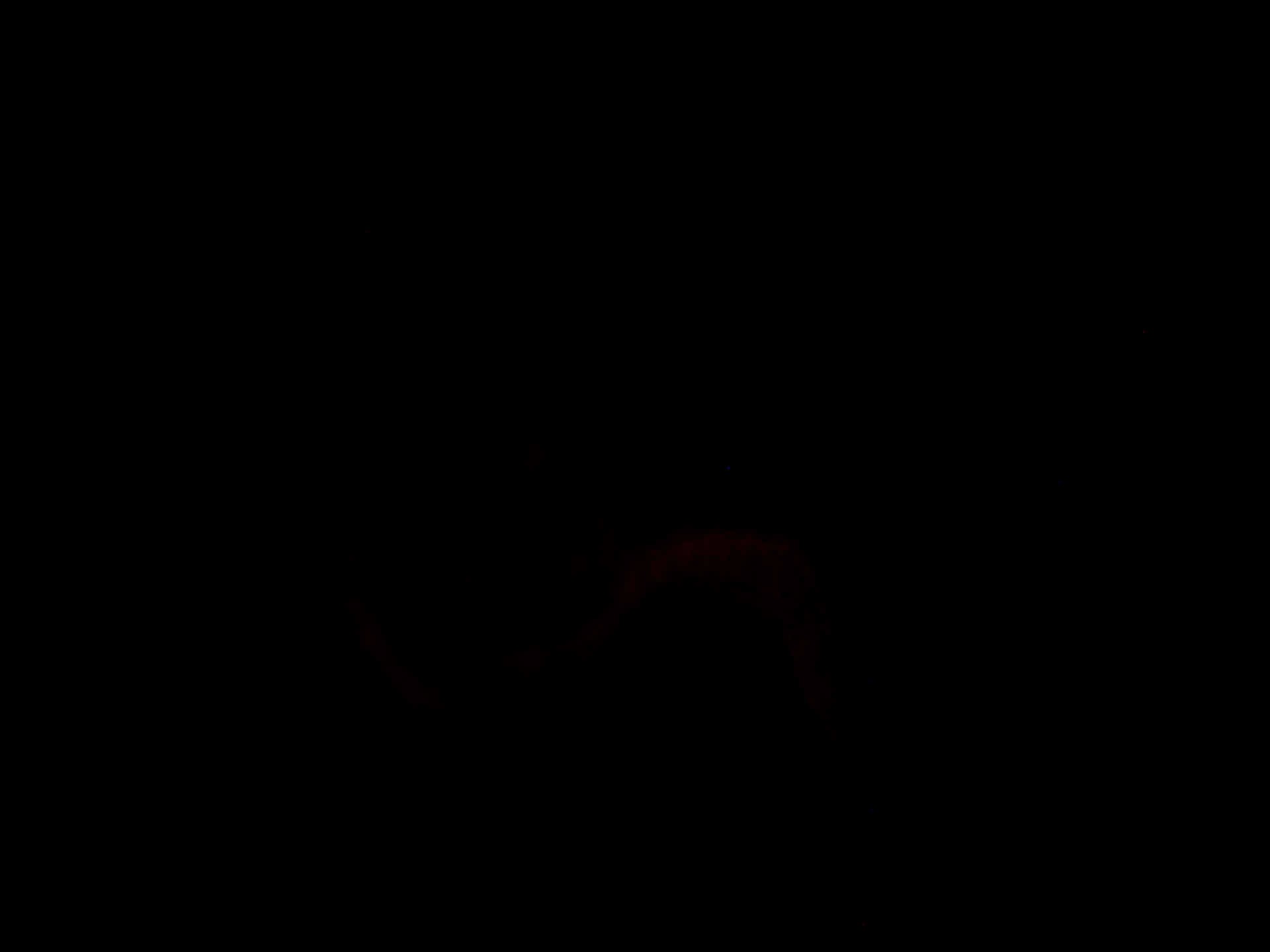

Supplement: Supplementary file 17 — Source data Fig. 1 [file 44318_2025_643_MOESM17_ESM.zip › Figure 1/1A/embryo_24 hpf_red fluorescence.tif]

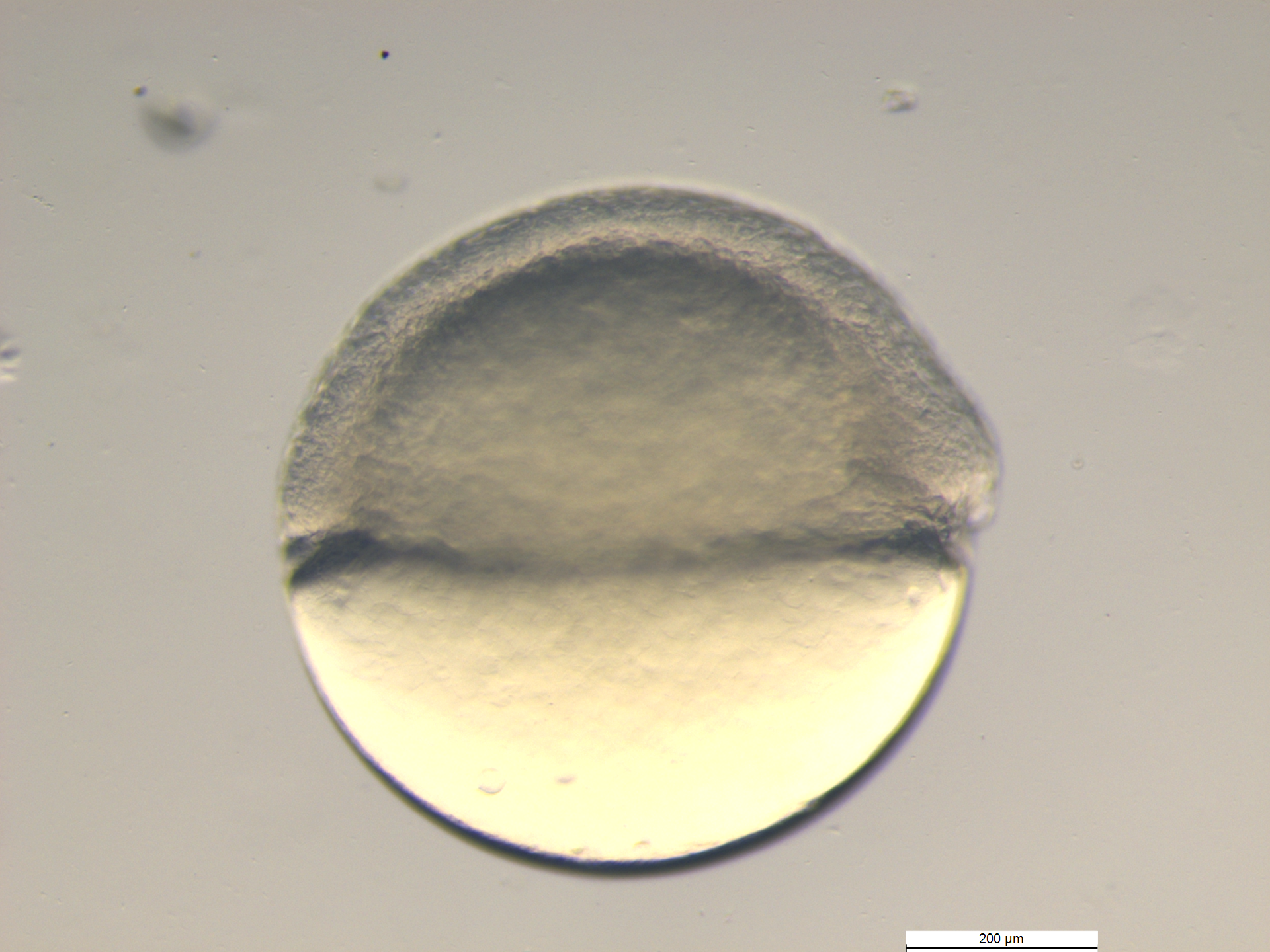

Supplement: Supplementary file 17 — Source data Fig. 1 [file 44318_2025_643_MOESM17_ESM.zip › Figure 1/1A/embryo_6 hpf_bright field.tif]

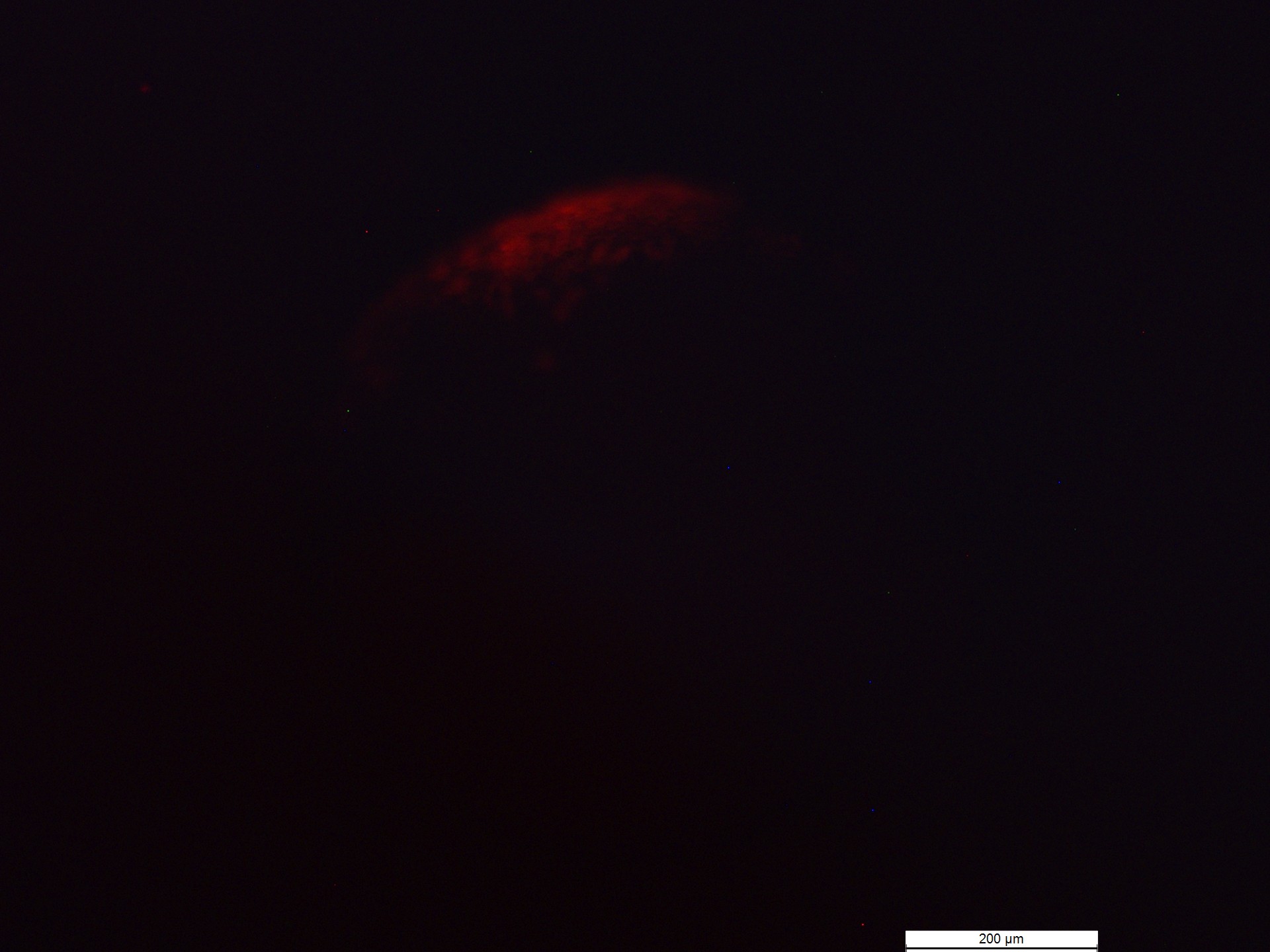

Supplement: Supplementary file 17 — Source data Fig. 1 [file 44318_2025_643_MOESM17_ESM.zip › Figure 1/1A/embryo_6 hpf_red fluorescence.tif]

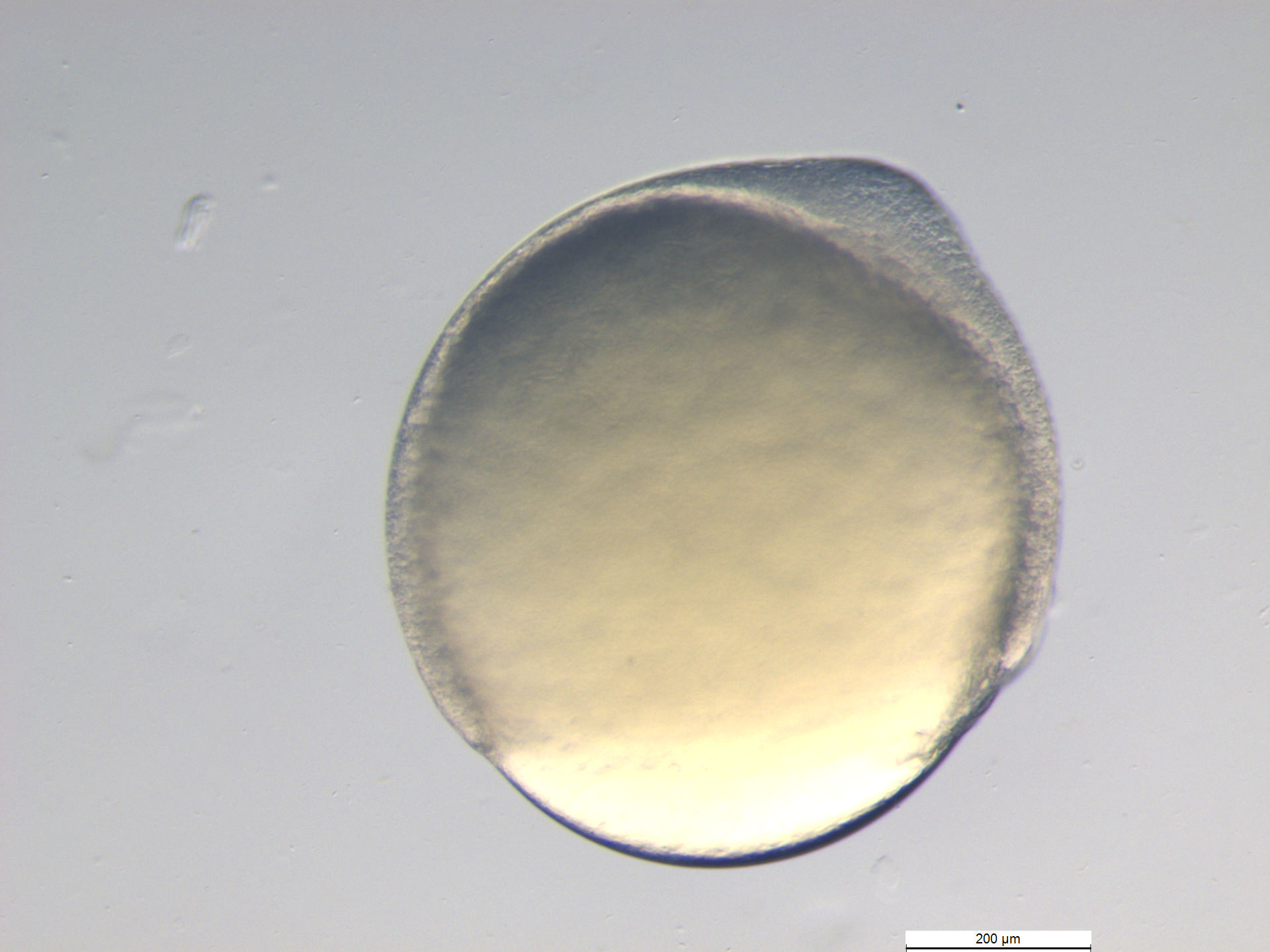

Supplement: Supplementary file 17 — Source data Fig. 1 [file 44318_2025_643_MOESM17_ESM.zip › Figure 1/1A/embryo_8 hpf_bright field.tif]

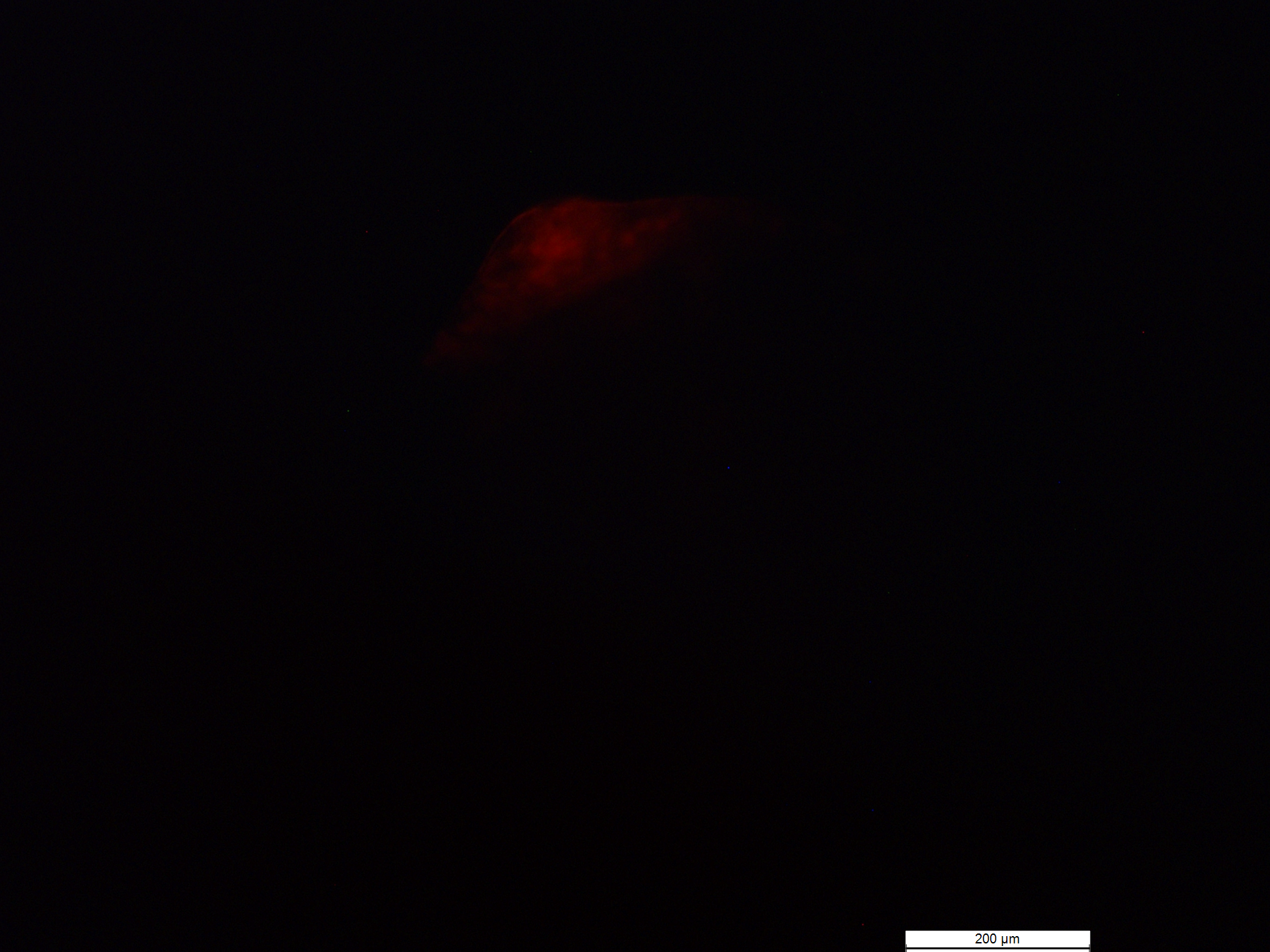

Supplement: Supplementary file 17 — Source data Fig. 1 [file 44318_2025_643_MOESM17_ESM.zip › Figure 1/1A/embryo_8 hpf_red fluorescence.tif]

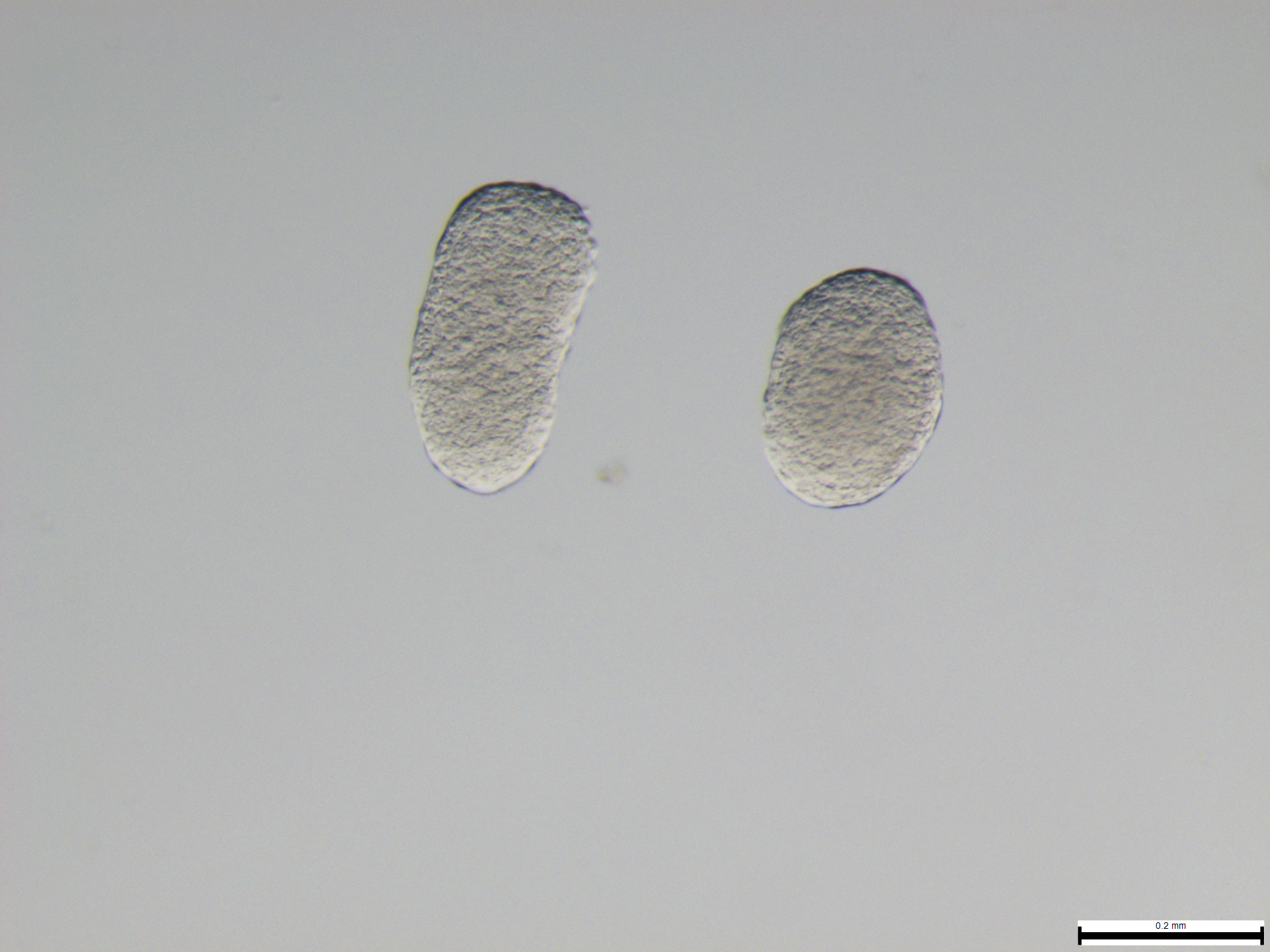

Supplement: Supplementary file 17 — Source data Fig. 1 [file 44318_2025_643_MOESM17_ESM.zip › Figure 1/1A/explant_12 hpf_bright field.tif]

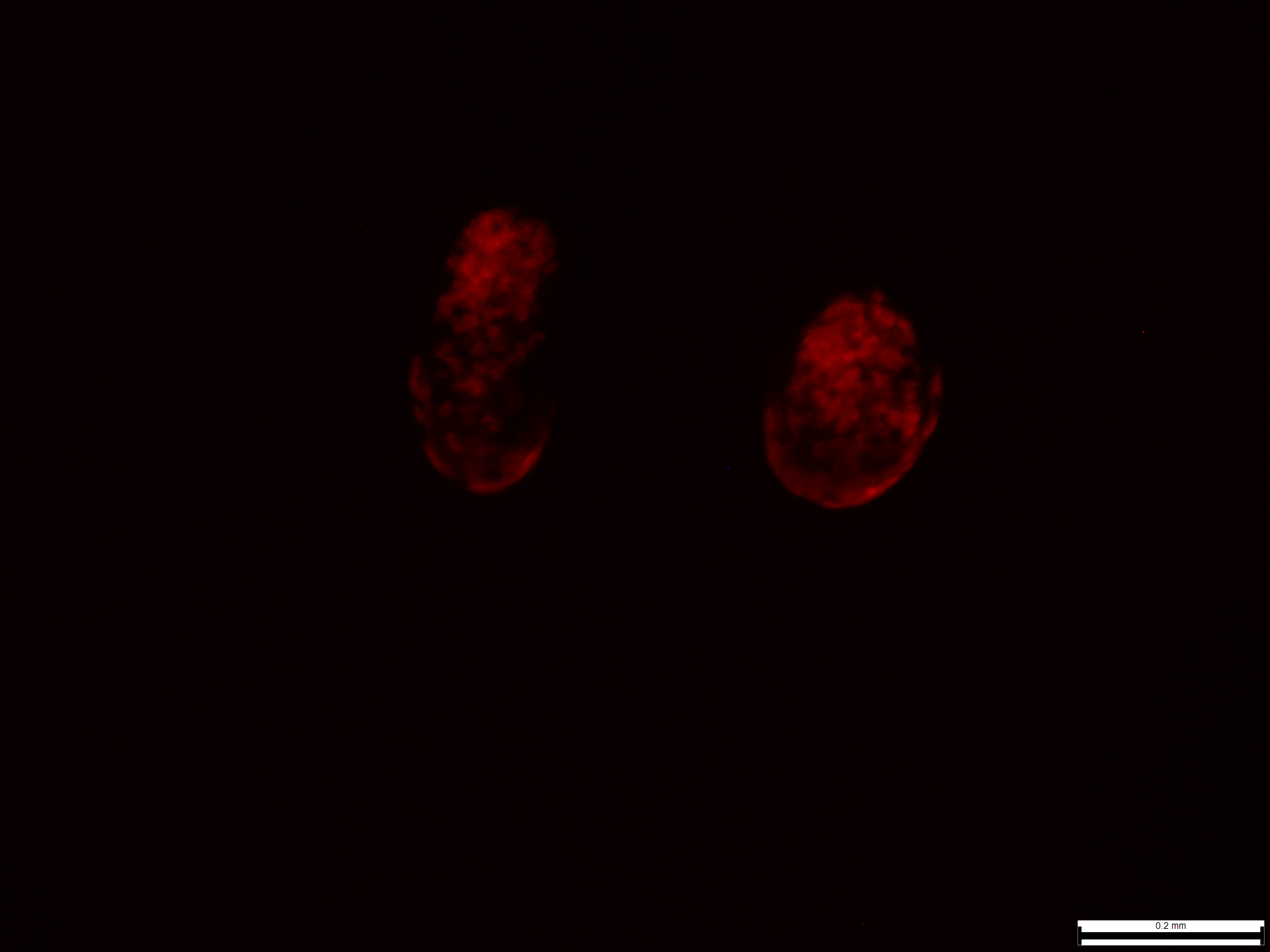

Supplement: Supplementary file 17 — Source data Fig. 1 [file 44318_2025_643_MOESM17_ESM.zip › Figure 1/1A/explant_12 hpf_red fluorescence.tif]

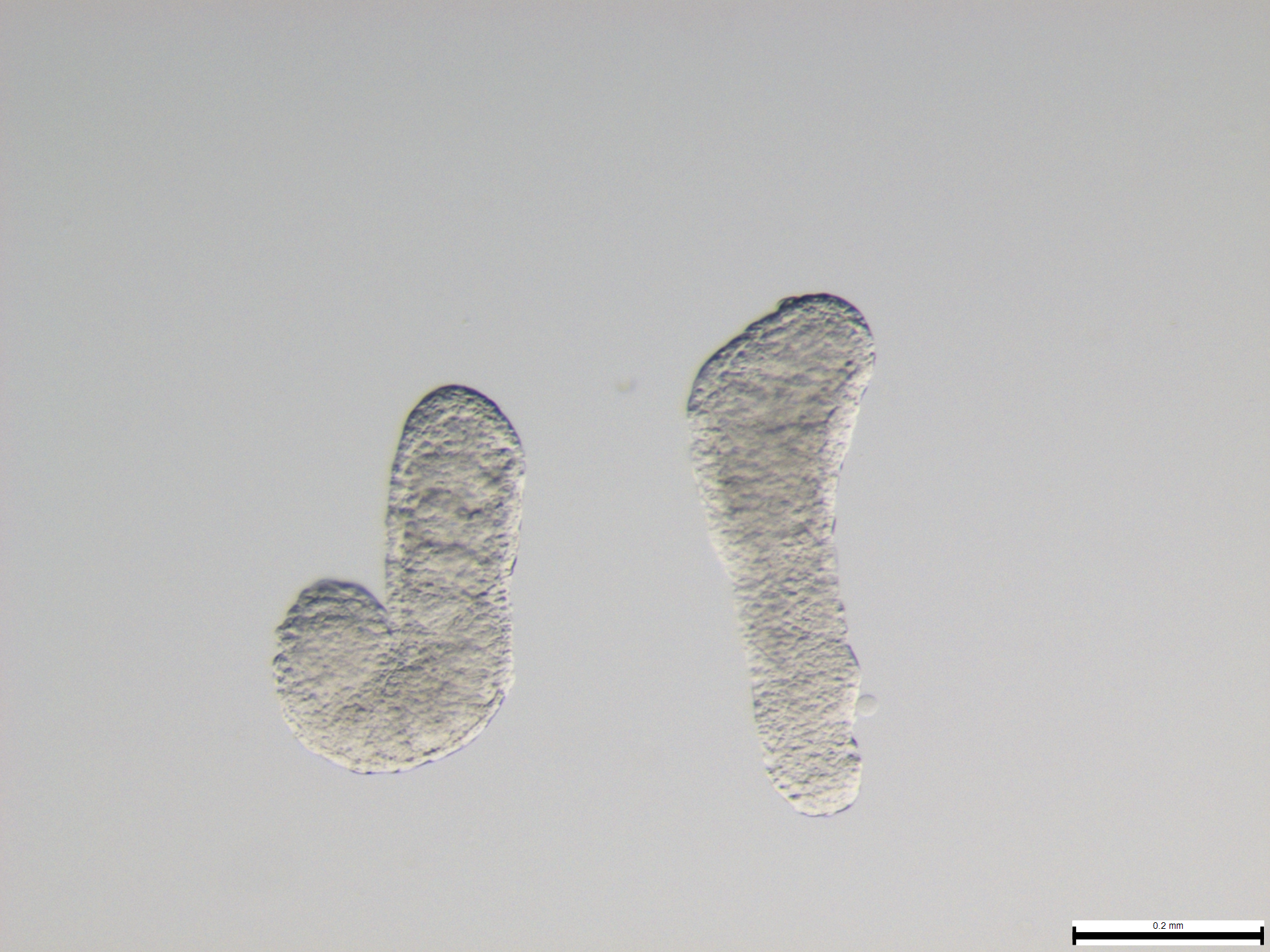

Supplement: Supplementary file 17 — Source data Fig. 1 [file 44318_2025_643_MOESM17_ESM.zip › Figure 1/1A/explant_24 hpf_bright field.tif]

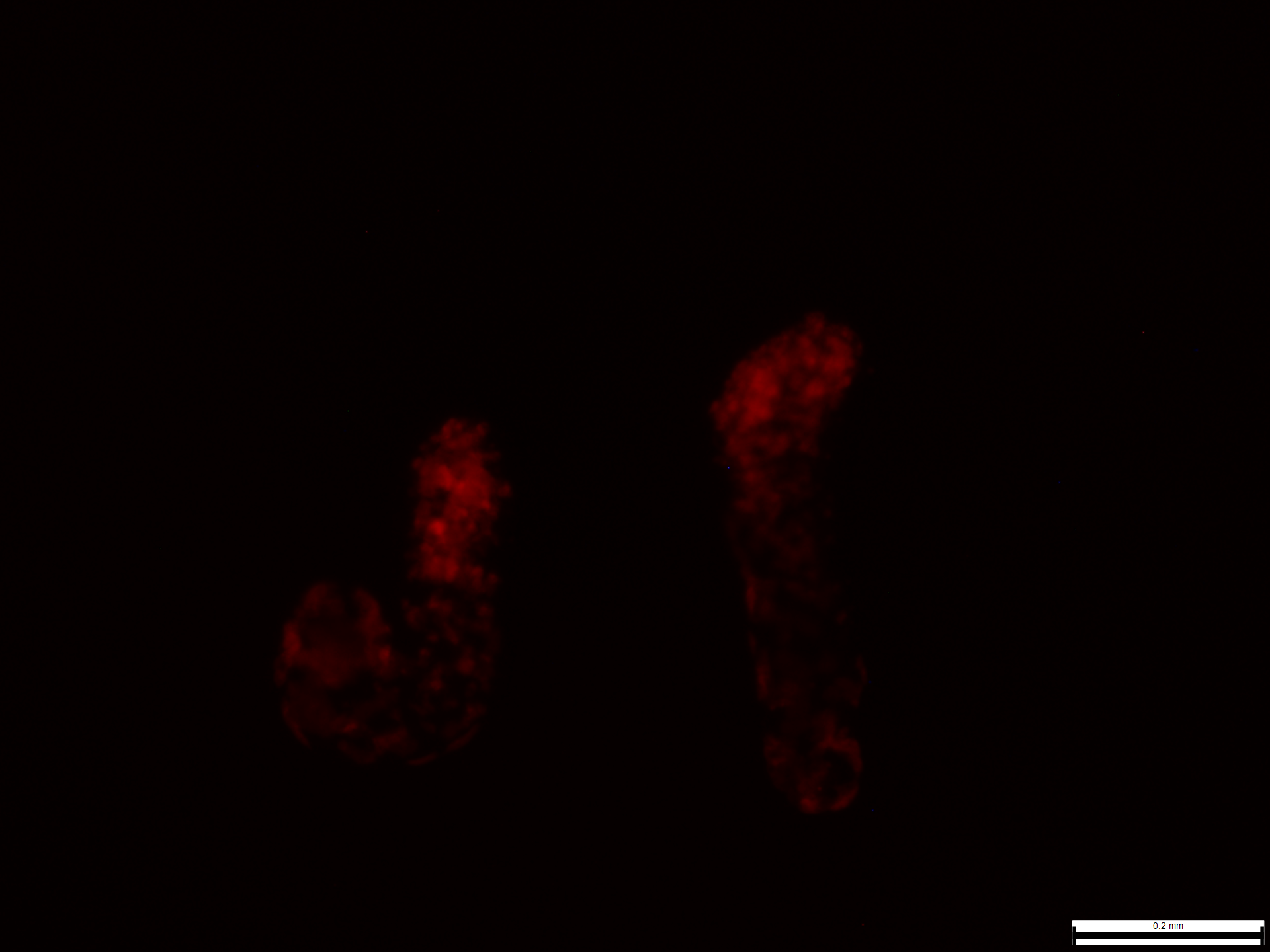

Supplement: Supplementary file 17 — Source data Fig. 1 [file 44318_2025_643_MOESM17_ESM.zip › Figure 1/1A/explant_24 hpf_red fluorescence.tif]

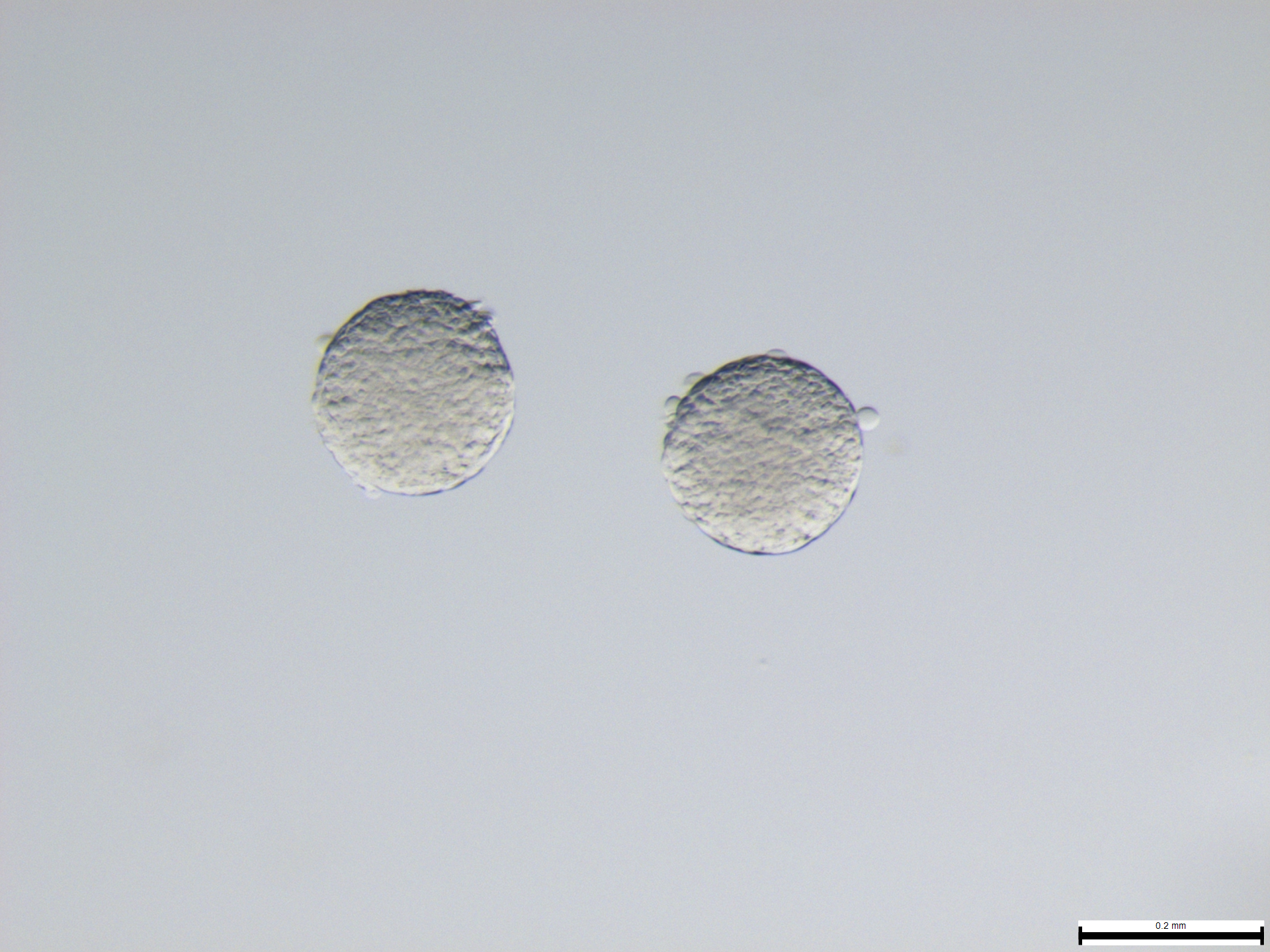

Supplement: Supplementary file 17 — Source data Fig. 1 [file 44318_2025_643_MOESM17_ESM.zip › Figure 1/1A/explant_6 hpf_bright field.tif]

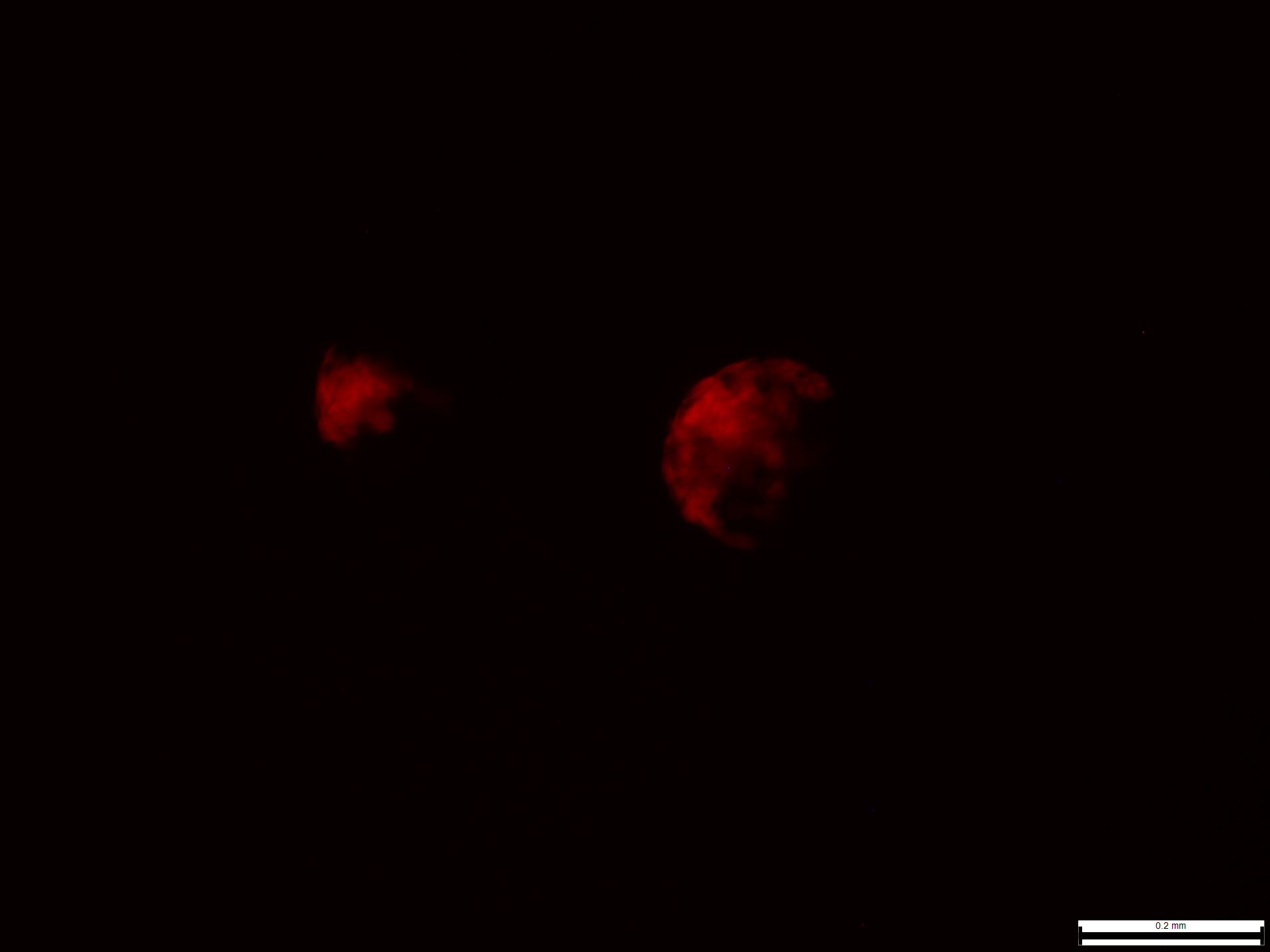

Supplement: Supplementary file 17 — Source data Fig. 1 [file 44318_2025_643_MOESM17_ESM.zip › Figure 1/1A/explant_6 hpf_red fluorescence.tif]

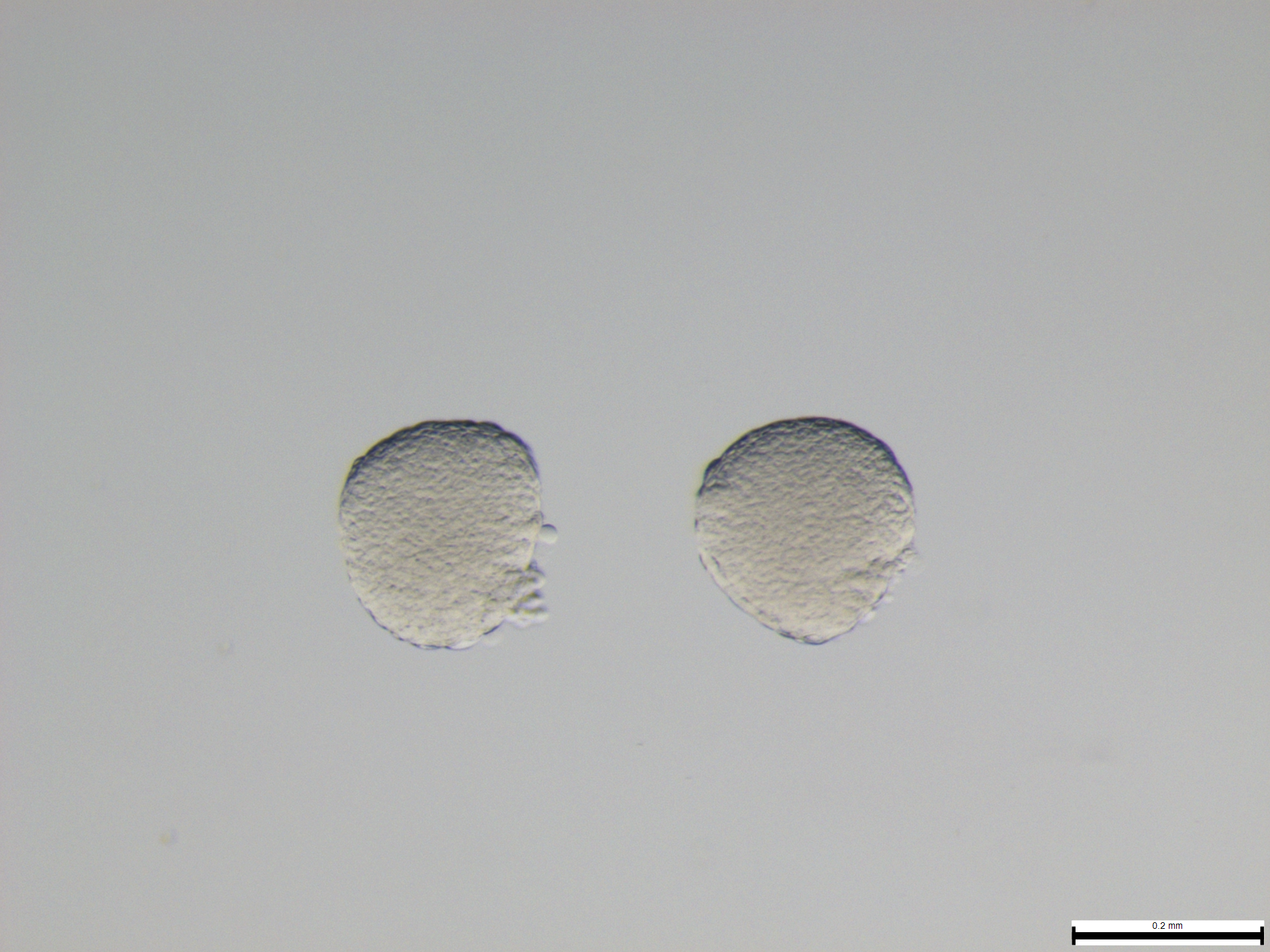

Supplement: Supplementary file 17 — Source data Fig. 1 [file 44318_2025_643_MOESM17_ESM.zip › Figure 1/1A/explant_8 hpf_bright field.tif]

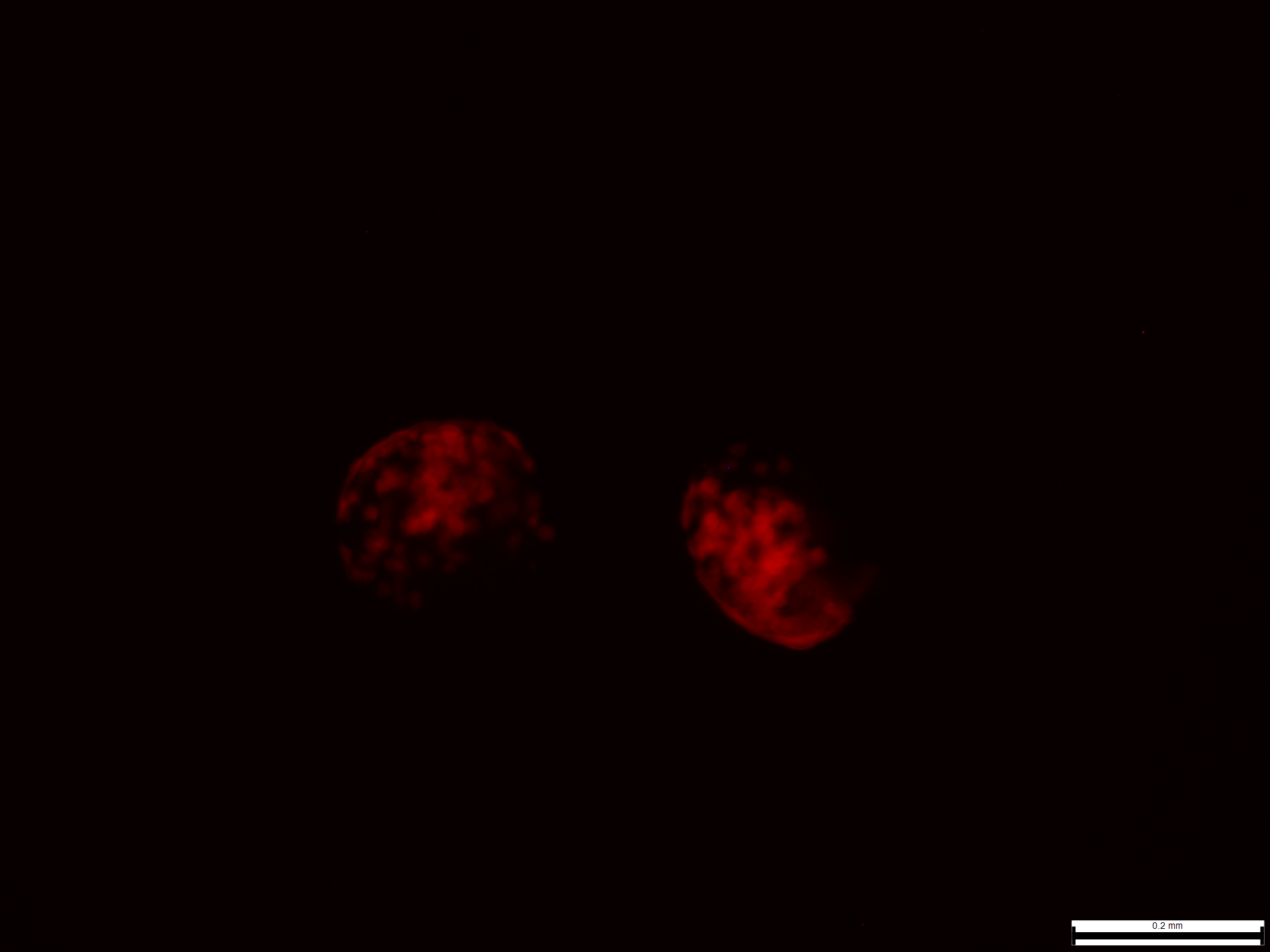

Supplement: Supplementary file 17 — Source data Fig. 1 [file 44318_2025_643_MOESM17_ESM.zip › Figure 1/1A/explant_8 hpf_red fluorescence.tif]

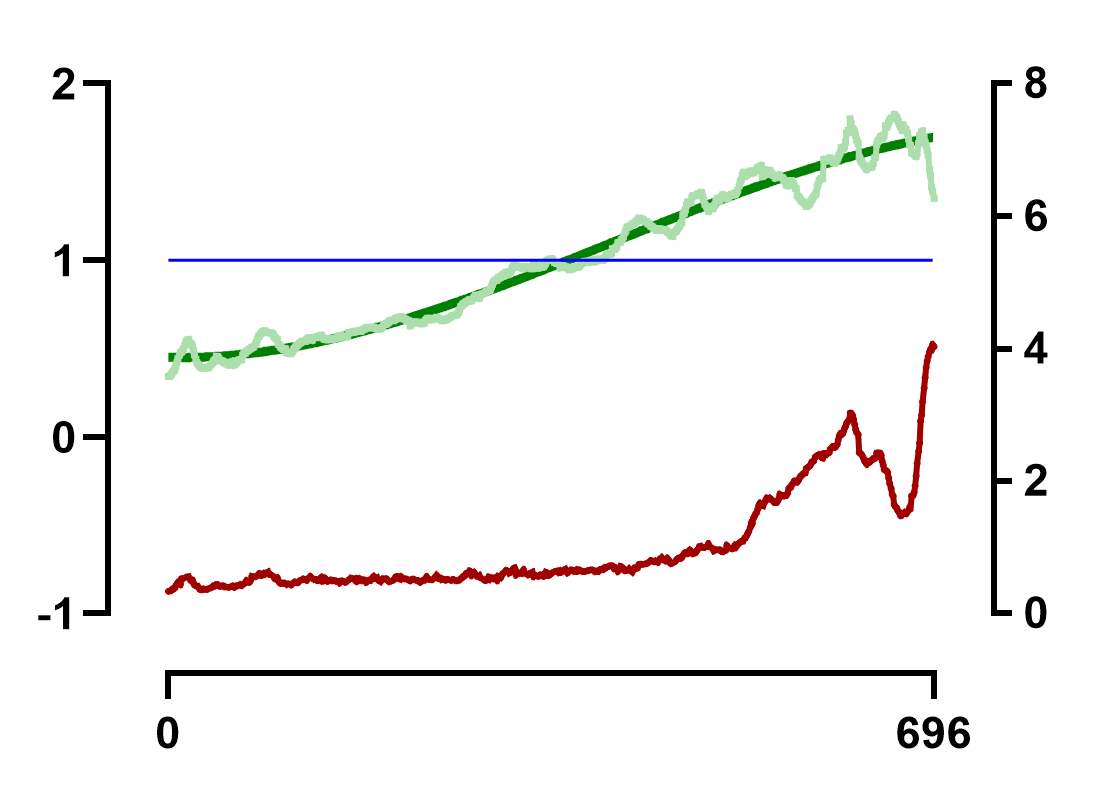

Supplement: Supplementary file 17 — Source data Fig. 1 [file 44318_2025_643_MOESM17_ESM.zip › Figure 1/1B/bmp4 explant.tif]

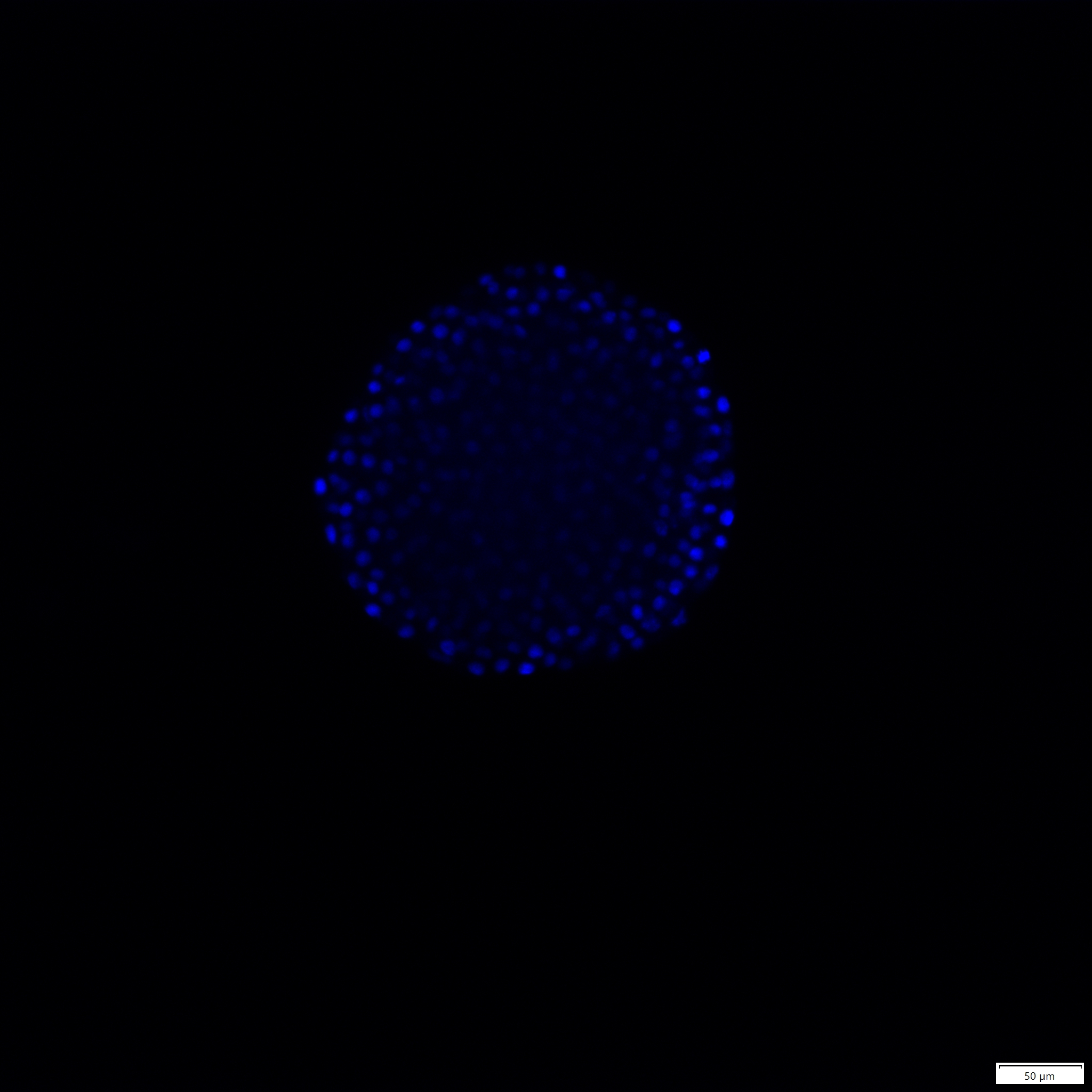

Supplement: Supplementary file 17 — Source data Fig. 1 [file 44318_2025_643_MOESM17_ESM.zip › Figure 1/1B/bmp4 explant_DAPI.jpg]

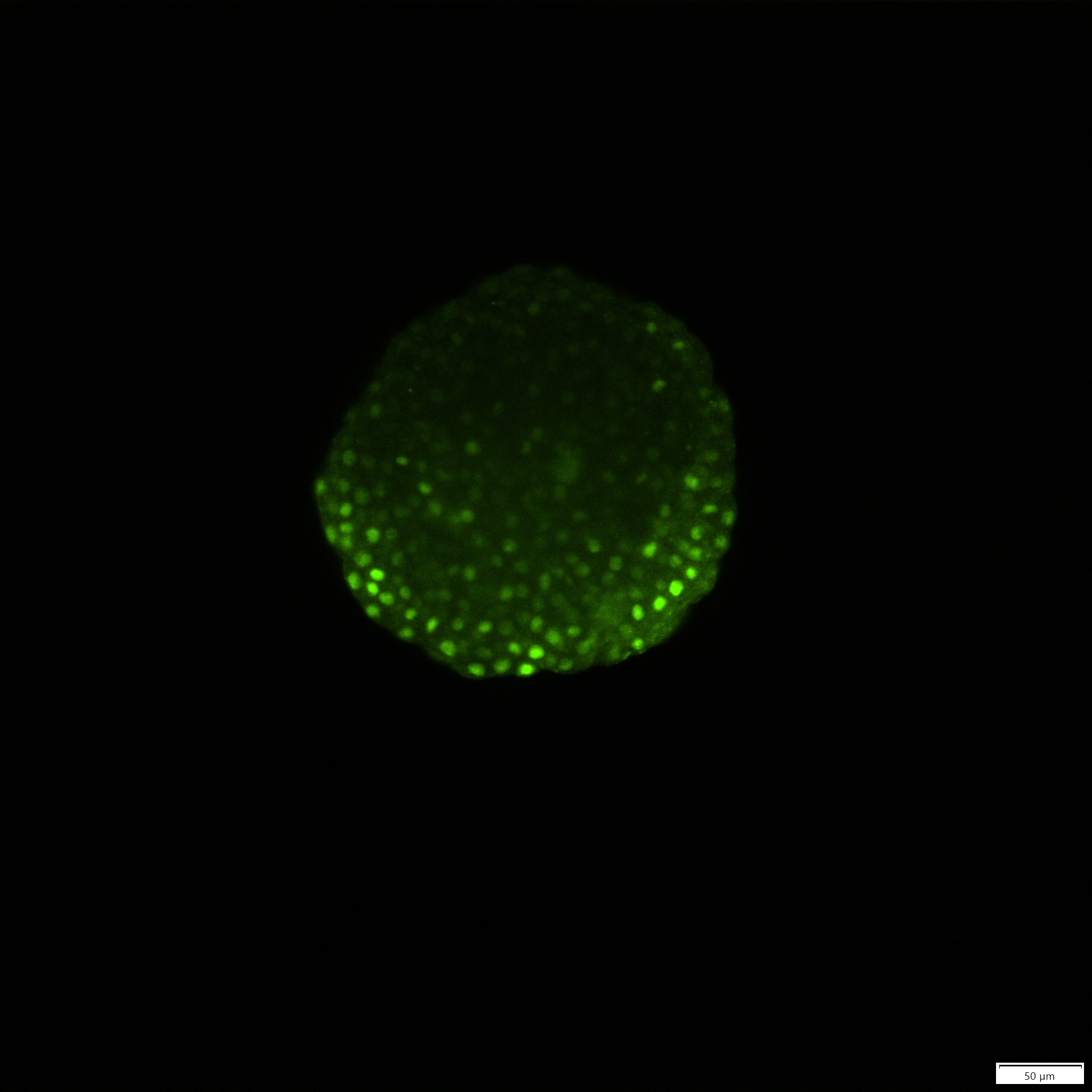

Supplement: Supplementary file 17 — Source data Fig. 1 [file 44318_2025_643_MOESM17_ESM.zip › Figure 1/1B/bmp4 explant_ISH_pSmad 159.jpg]

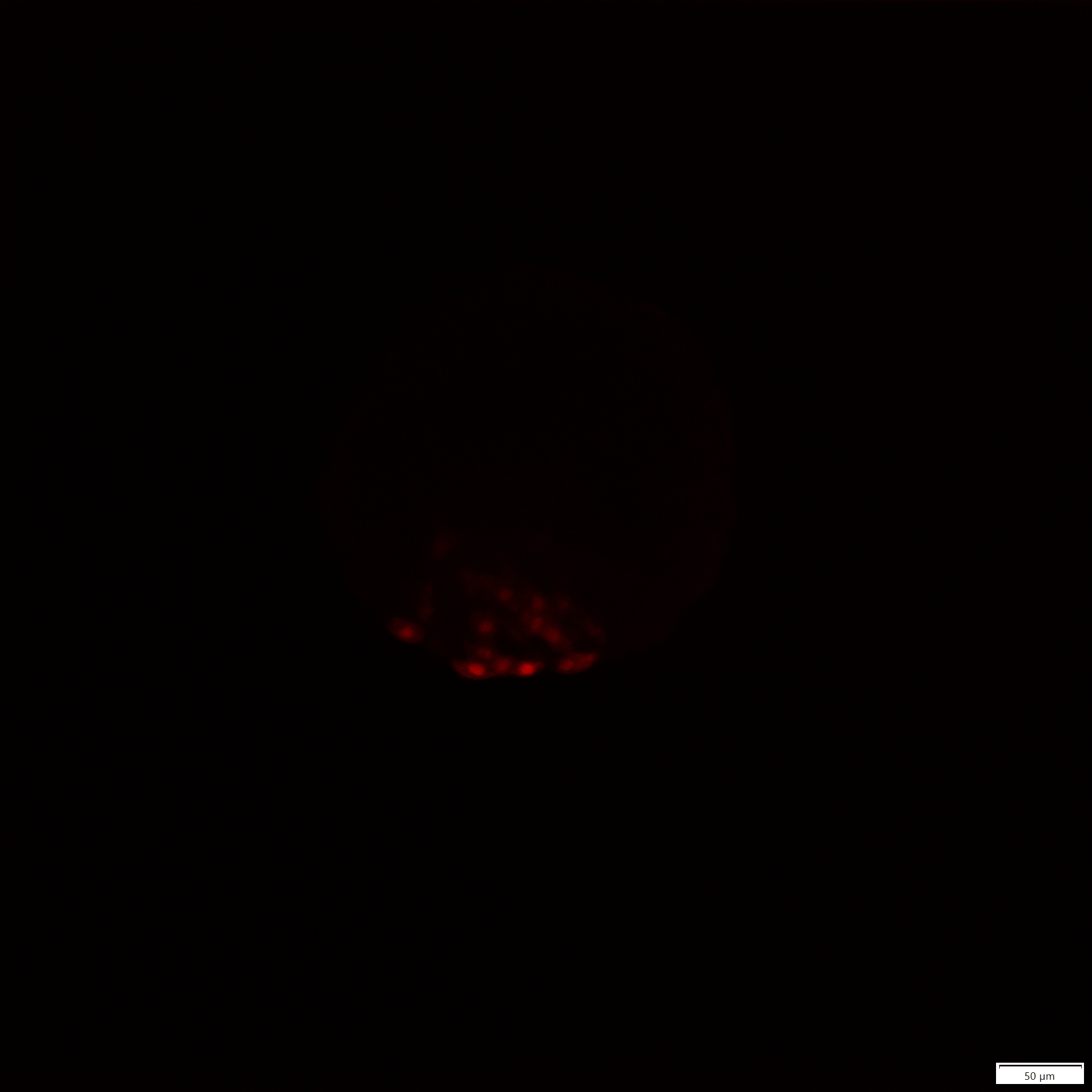

Supplement: Supplementary file 17 — Source data Fig. 1 [file 44318_2025_643_MOESM17_ESM.zip › Figure 1/1B/bmp4 explant_RFP.jpg]

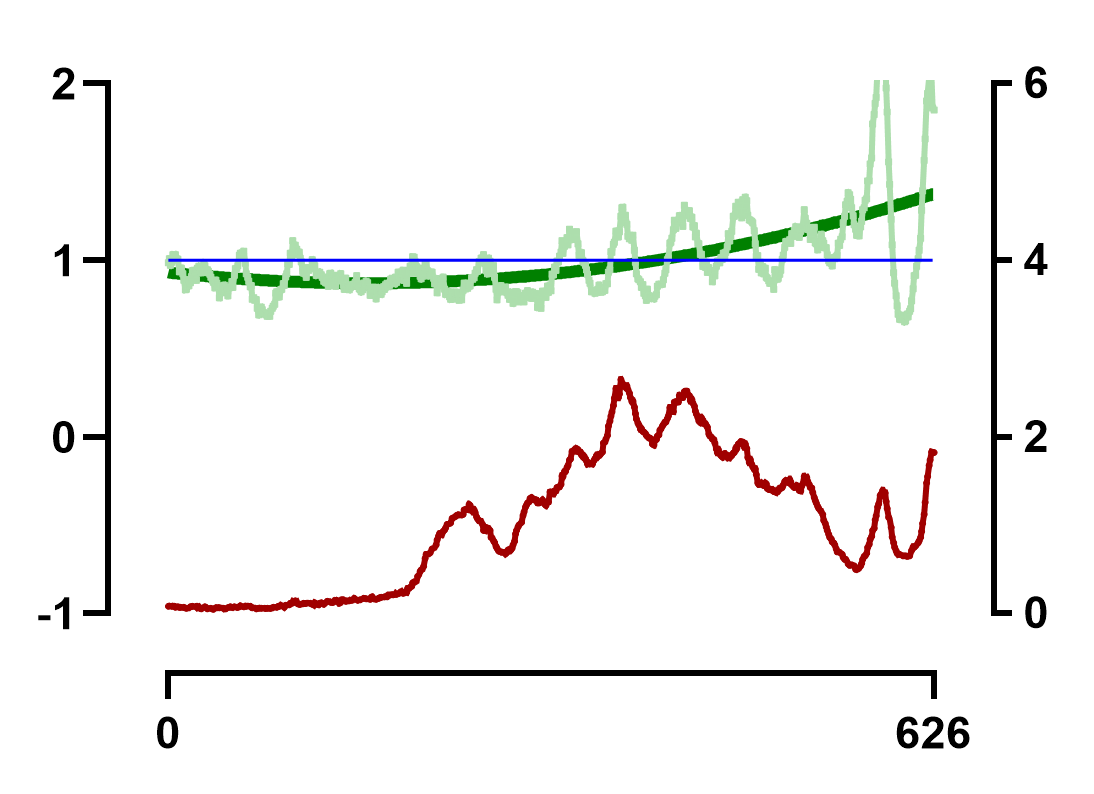

Supplement: Supplementary file 17 — Source data Fig. 1 [file 44318_2025_643_MOESM17_ESM.zip › Figure 1/1B/control explant.tif]

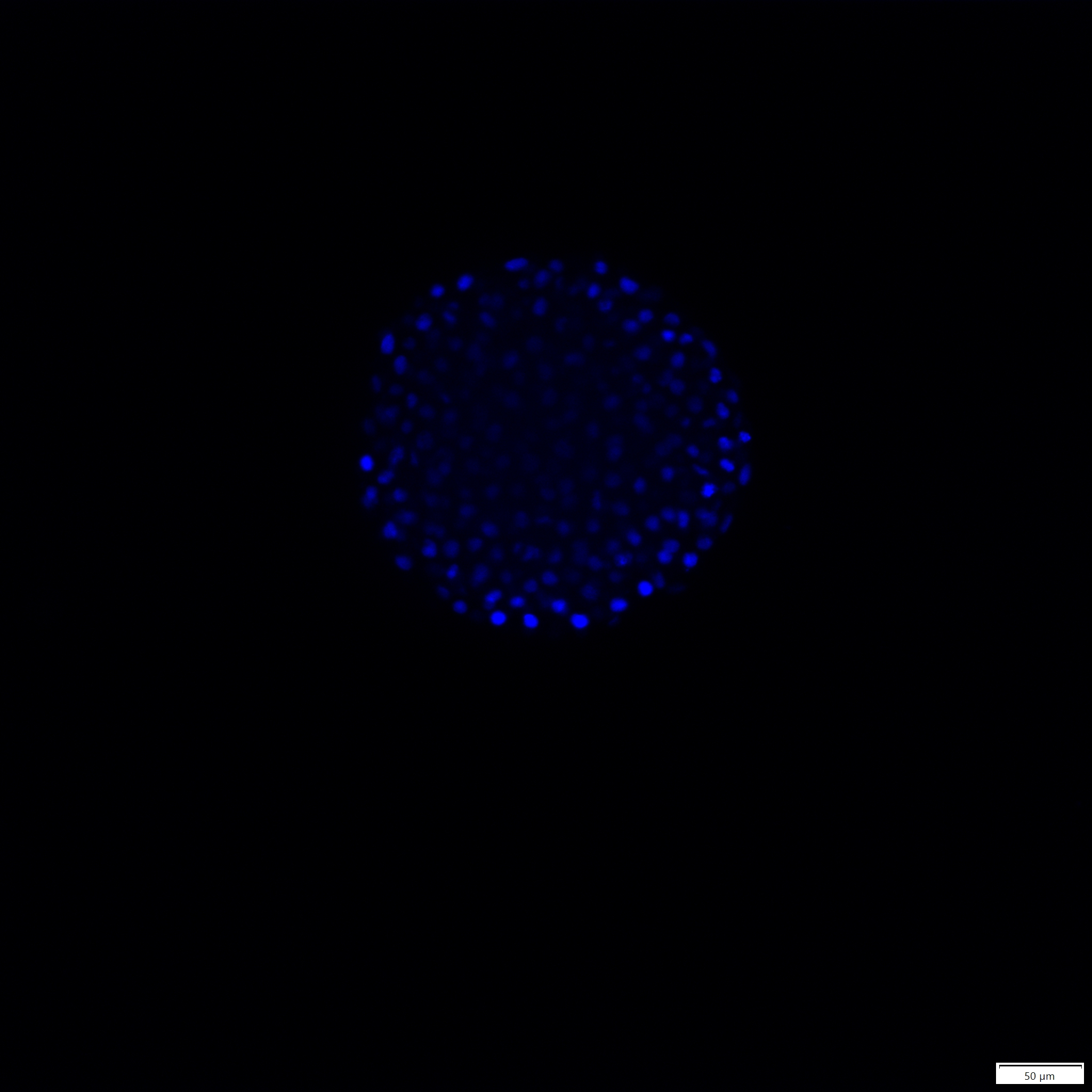

Supplement: Supplementary file 17 — Source data Fig. 1 [file 44318_2025_643_MOESM17_ESM.zip › Figure 1/1B/control explant_DAPI.jpg]

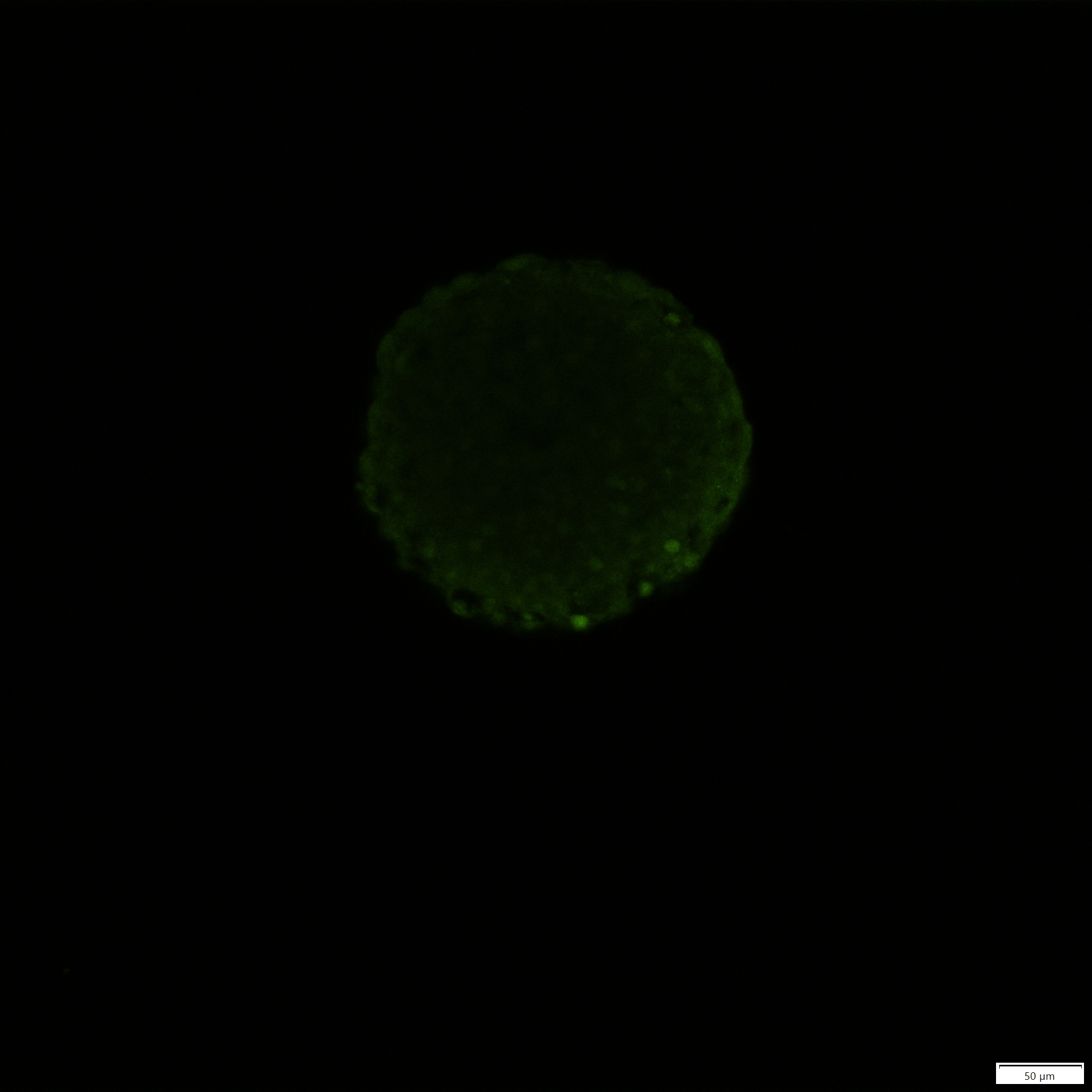

Supplement: Supplementary file 17 — Source data Fig. 1 [file 44318_2025_643_MOESM17_ESM.zip › Figure 1/1B/control explant_ISH_pSmad 159.jpg]

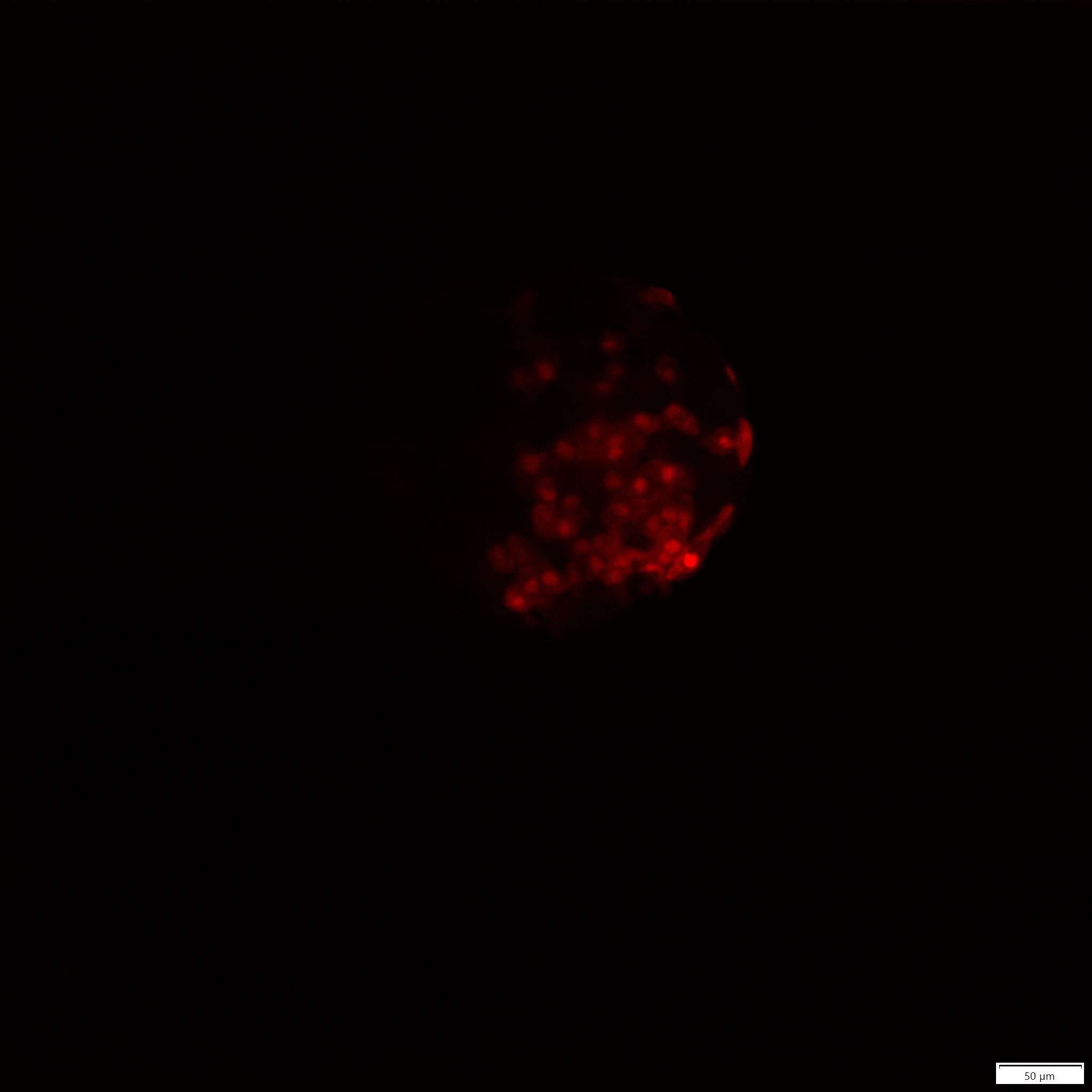

Supplement: Supplementary file 17 — Source data Fig. 1 [file 44318_2025_643_MOESM17_ESM.zip › Figure 1/1B/control explant_RFP.jpg]

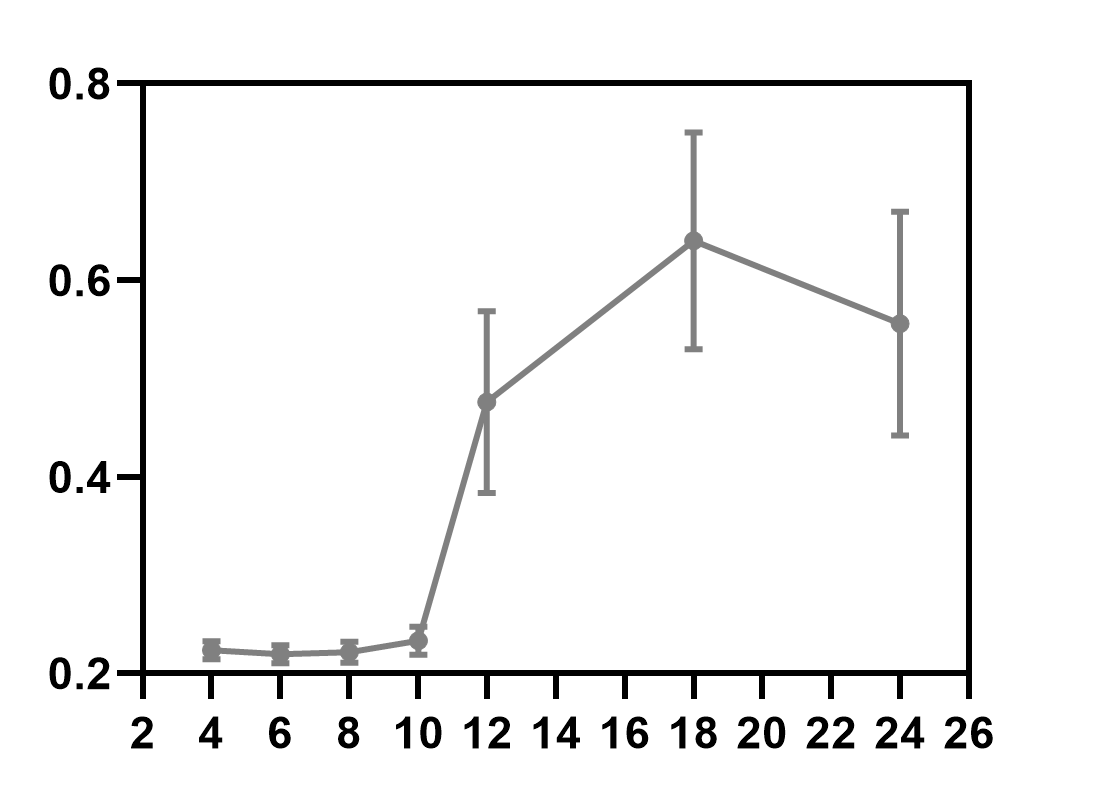

Supplement: Supplementary file 17 — Source data Fig. 1 [file 44318_2025_643_MOESM17_ESM.zip › Figure 1/1C/bmp4 explants.tif]

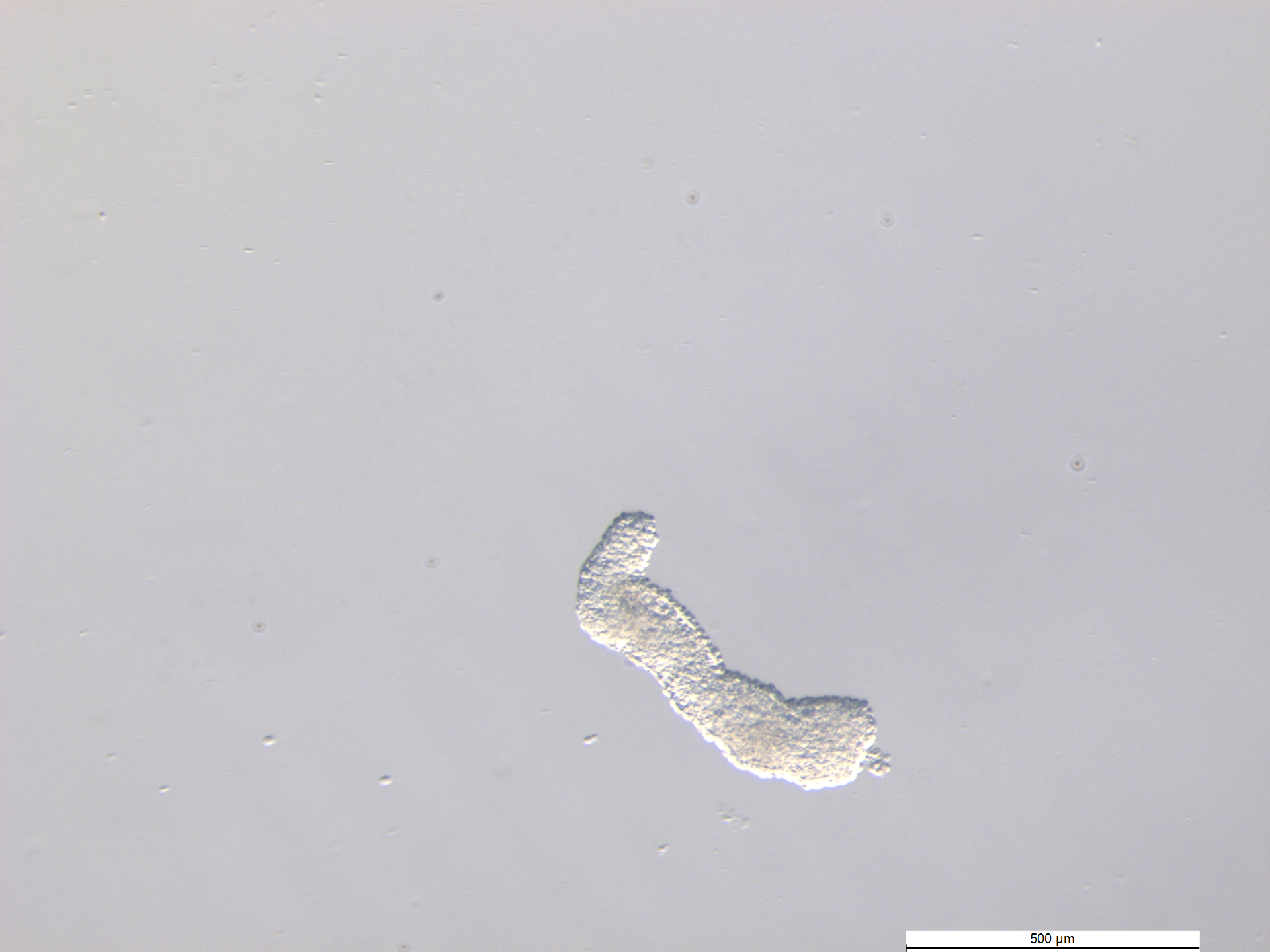

Supplement: Supplementary file 17 — Source data Fig. 1 [file 44318_2025_643_MOESM17_ESM.zip › Figure 1/1C/bmp4 explant_24hpf_inset.tif]

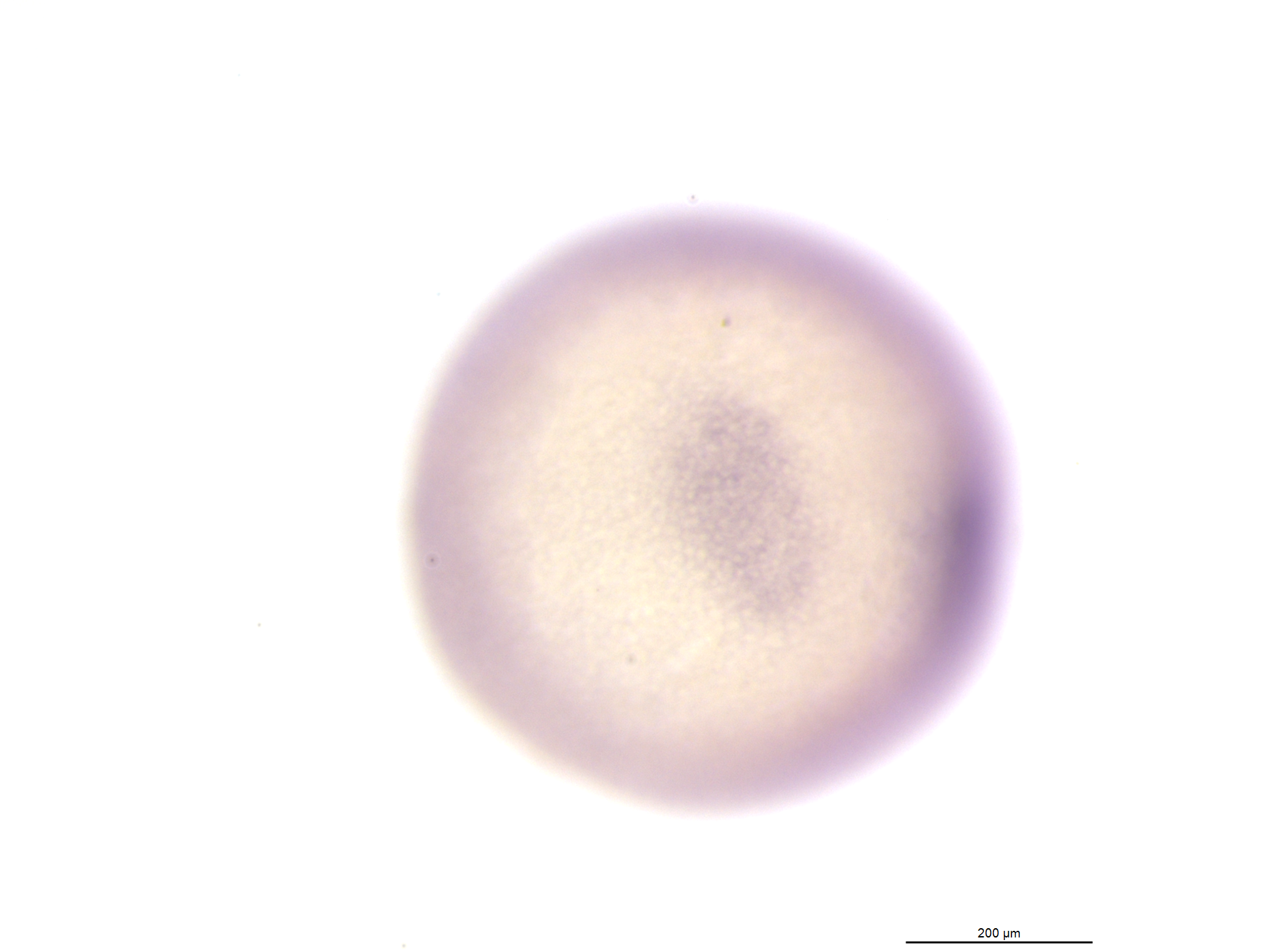

Supplement: Supplementary file 17 — Source data Fig. 1 [file 44318_2025_643_MOESM17_ESM.zip › Figure 1/1D/embryo_6hpf_ndr2.tif]

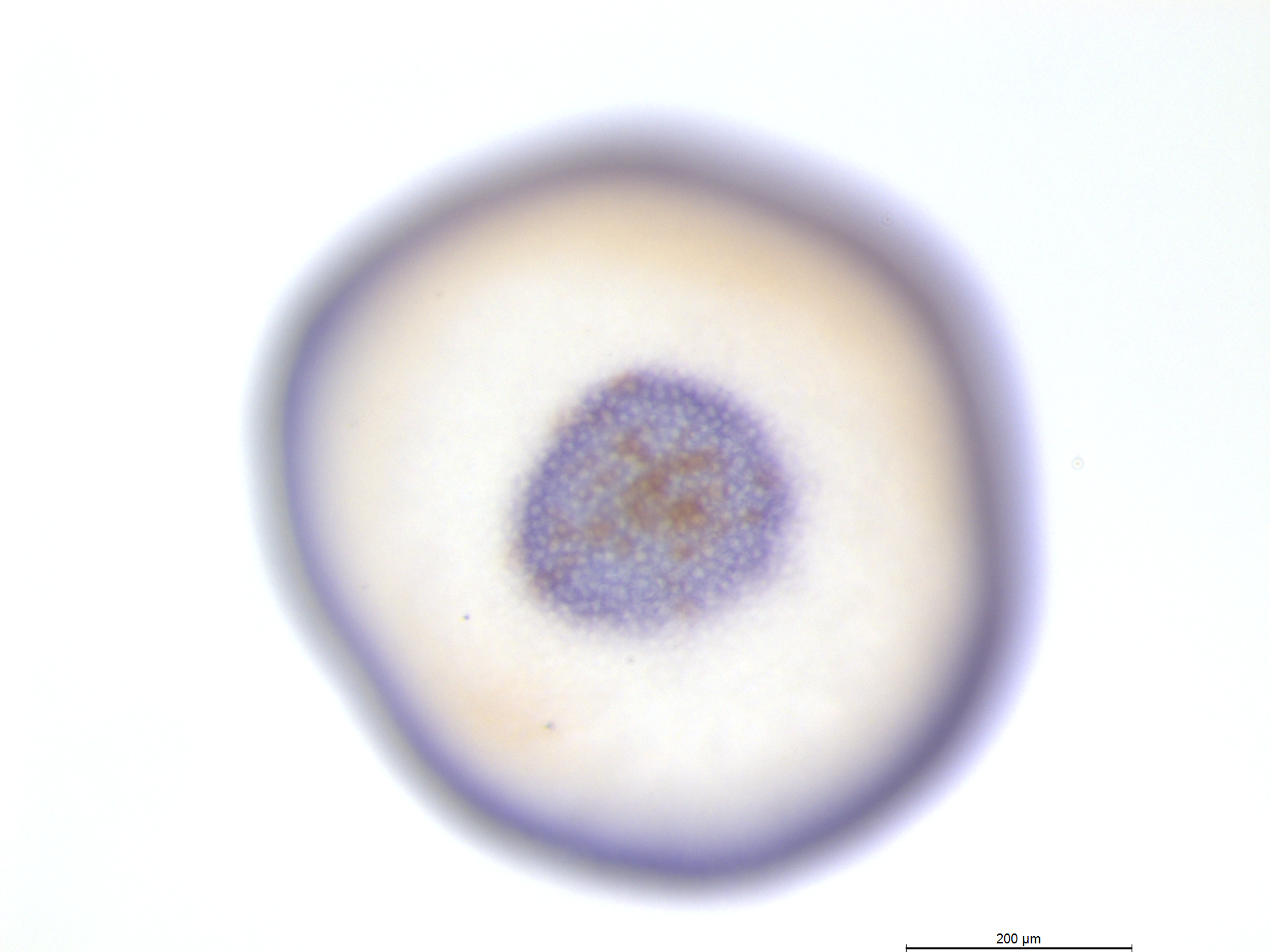

Supplement: Supplementary file 17 — Source data Fig. 1 [file 44318_2025_643_MOESM17_ESM.zip › Figure 1/1D/embryo_6hpf_tbxta.tif]

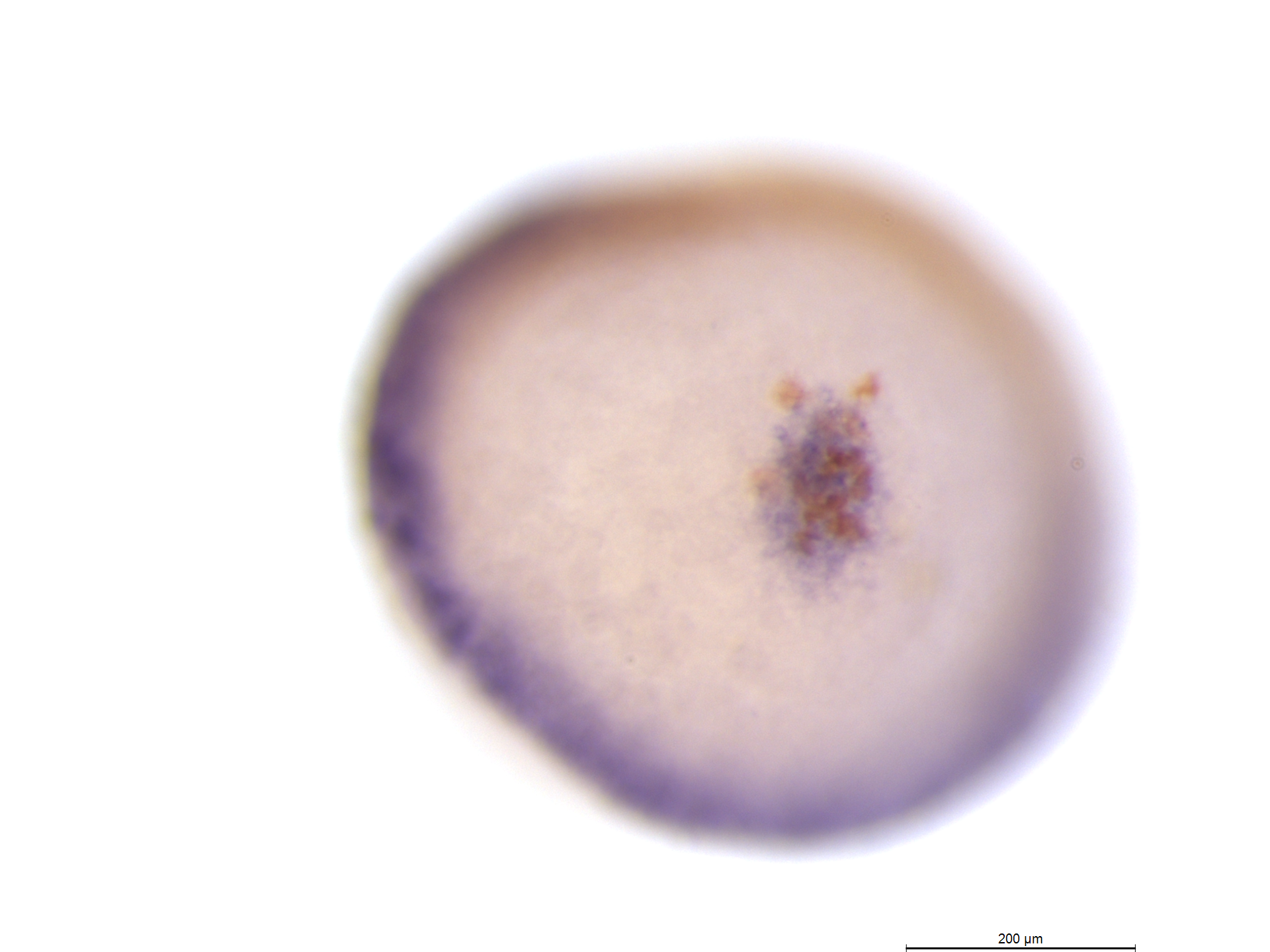

Supplement: Supplementary file 17 — Source data Fig. 1 [file 44318_2025_643_MOESM17_ESM.zip › Figure 1/1D/embryo_6hpf_wnt8a.tif]

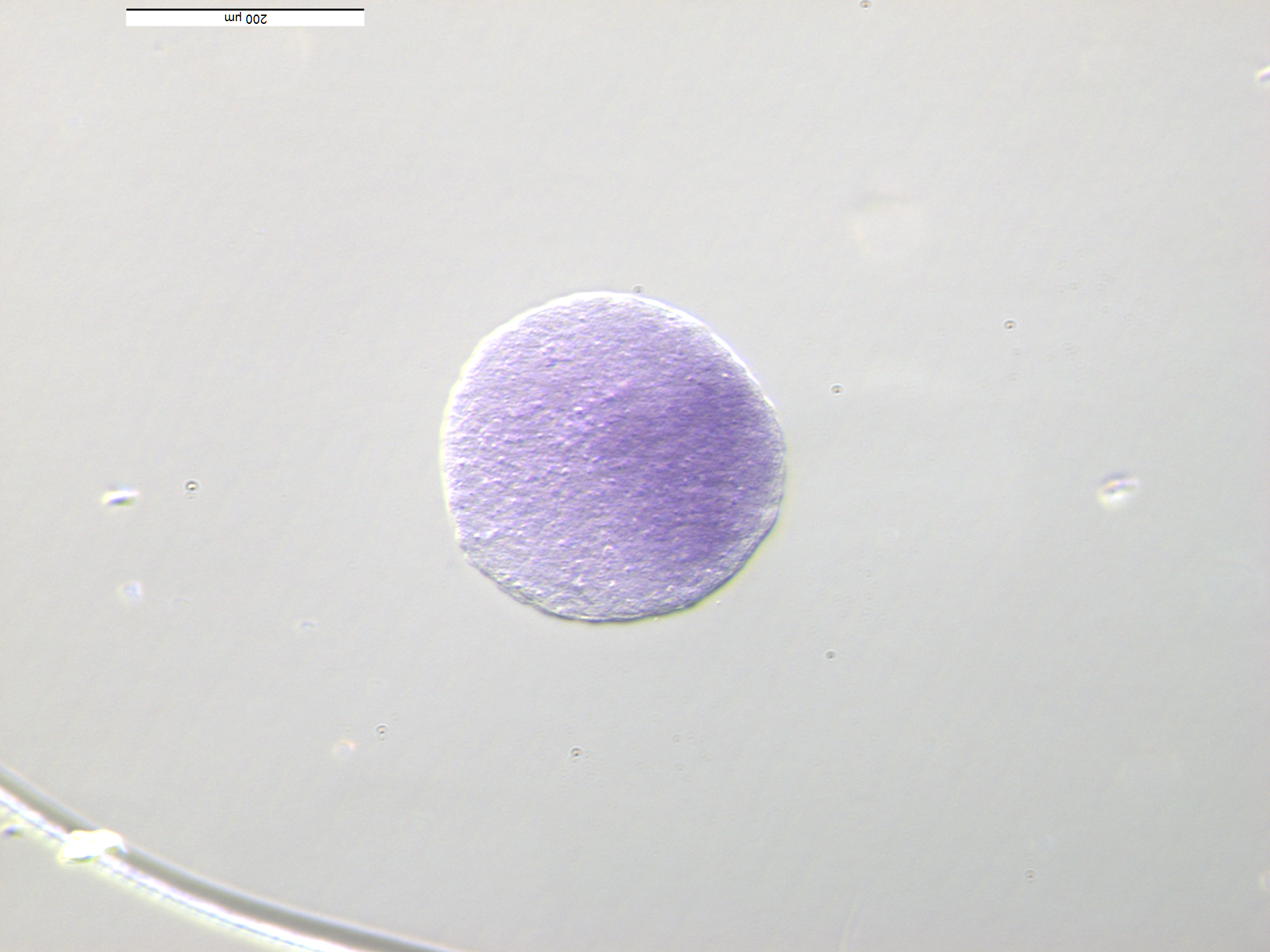

Supplement: Supplementary file 17 — Source data Fig. 1 [file 44318_2025_643_MOESM17_ESM.zip › Figure 1/1D/explant_6hpf_ndr2.tif]

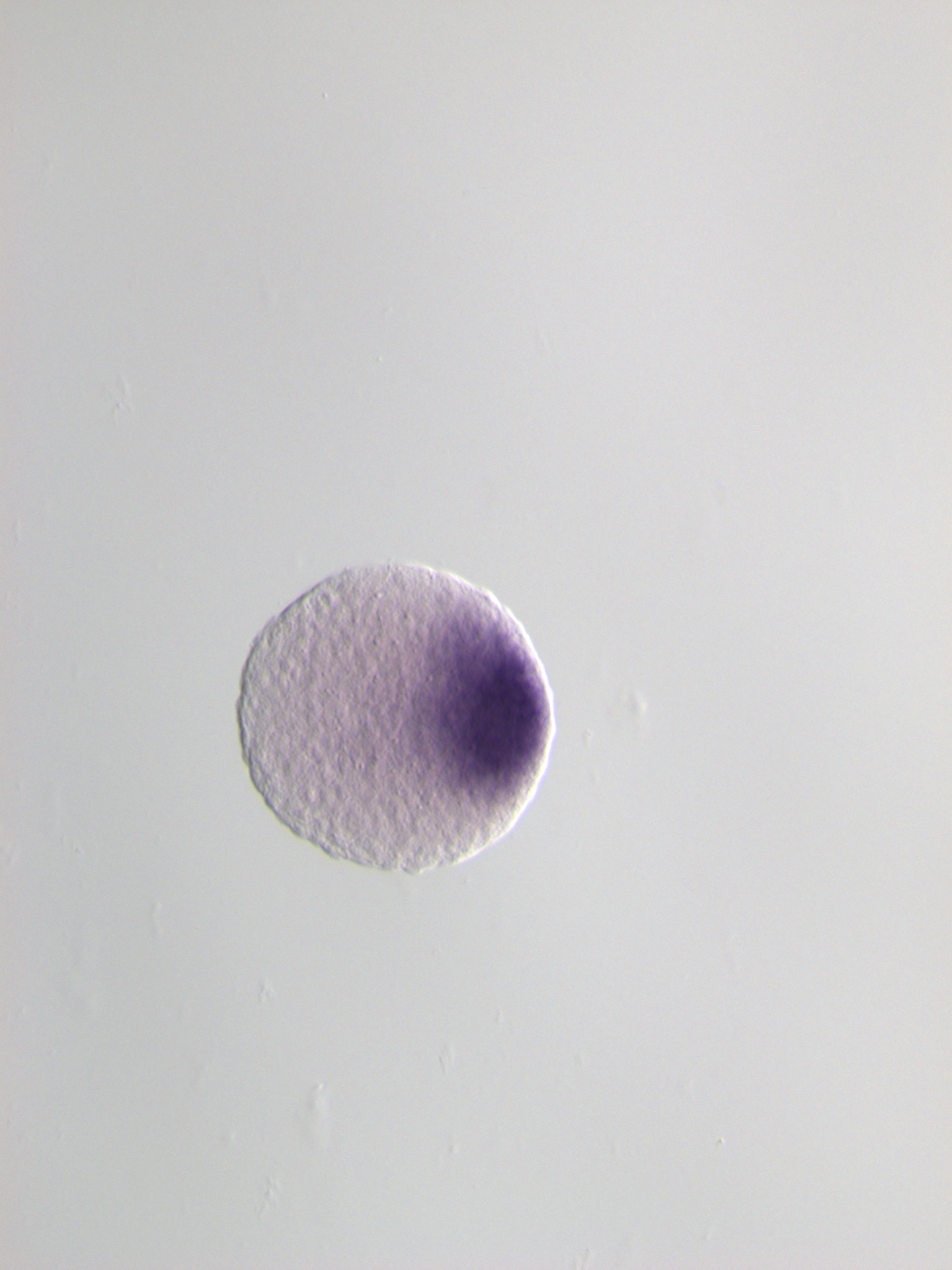

Supplement: Supplementary file 17 — Source data Fig. 1 [file 44318_2025_643_MOESM17_ESM.zip › Figure 1/1D/explant_6hpf_tbxta.tif]

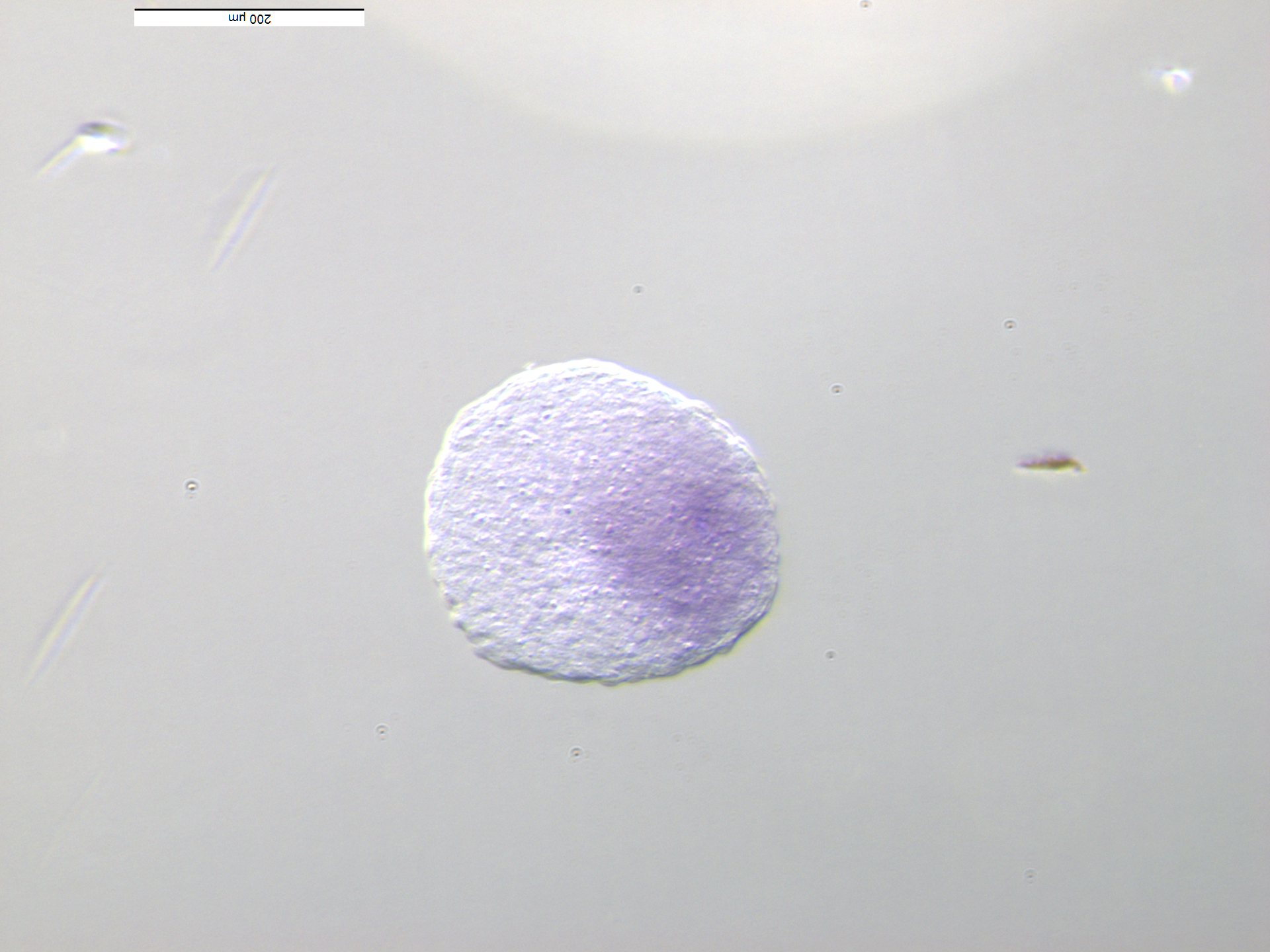

Supplement: Supplementary file 17 — Source data Fig. 1 [file 44318_2025_643_MOESM17_ESM.zip › Figure 1/1D/explant_6hpf_wnt8a.tif]

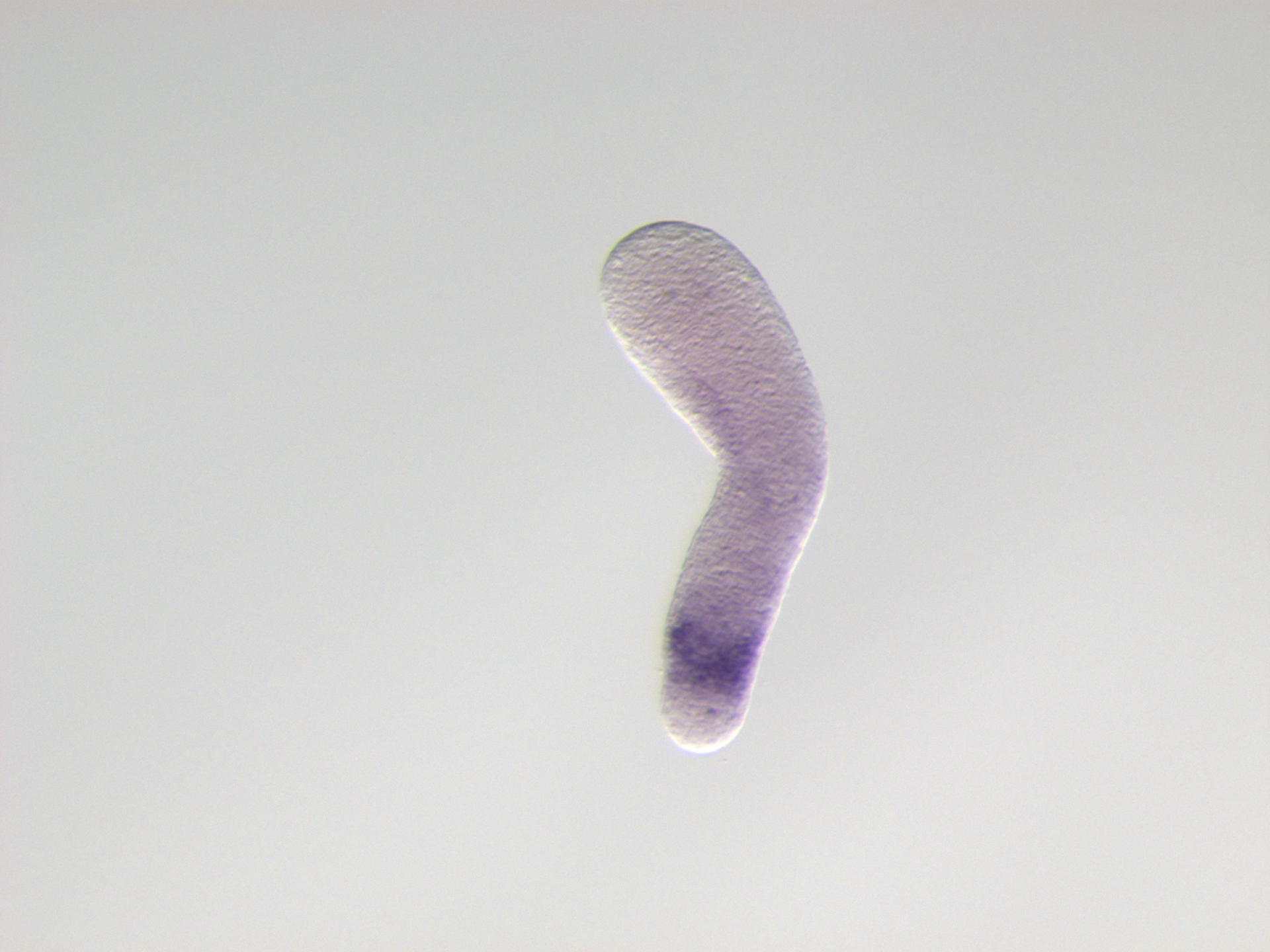

Supplement: Supplementary file 17 — Source data Fig. 1 [file 44318_2025_643_MOESM17_ESM.zip › Figure 1/1E/bmp4 explant_sox2.tif]

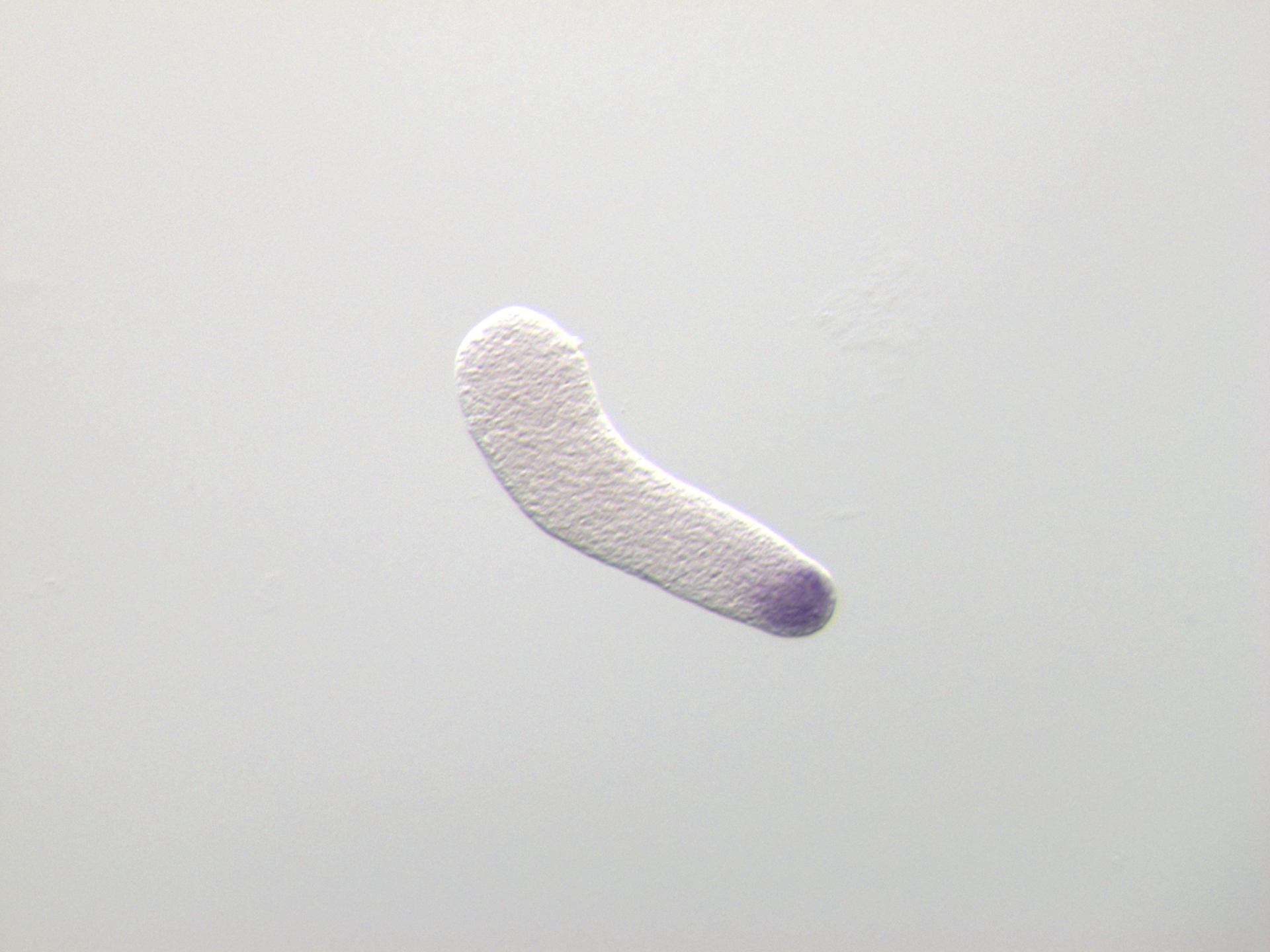

Supplement: Supplementary file 17 — Source data Fig. 1 [file 44318_2025_643_MOESM17_ESM.zip › Figure 1/1E/bmp4 explant_tbxta.tif]

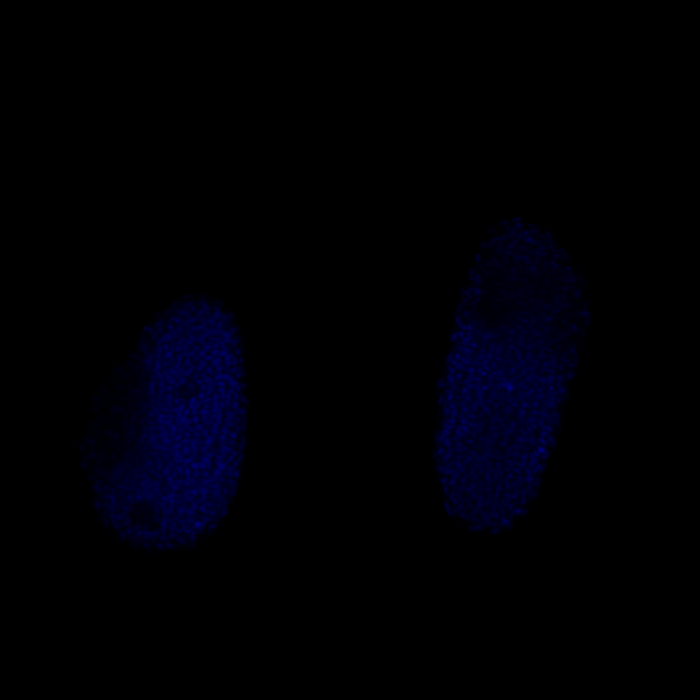

Supplement: Supplementary file 17 — Source data Fig. 1 [file 44318_2025_643_MOESM17_ESM.zip › Figure 1/1F/explant_DAPI.tif]

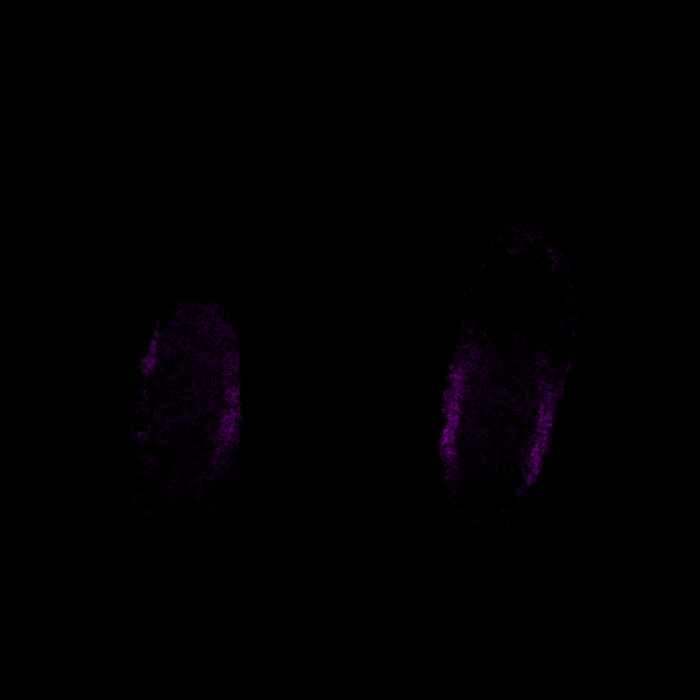

Supplement: Supplementary file 17 — Source data Fig. 1 [file 44318_2025_643_MOESM17_ESM.zip › Figure 1/1F/explant_HCR_sox2.tif]

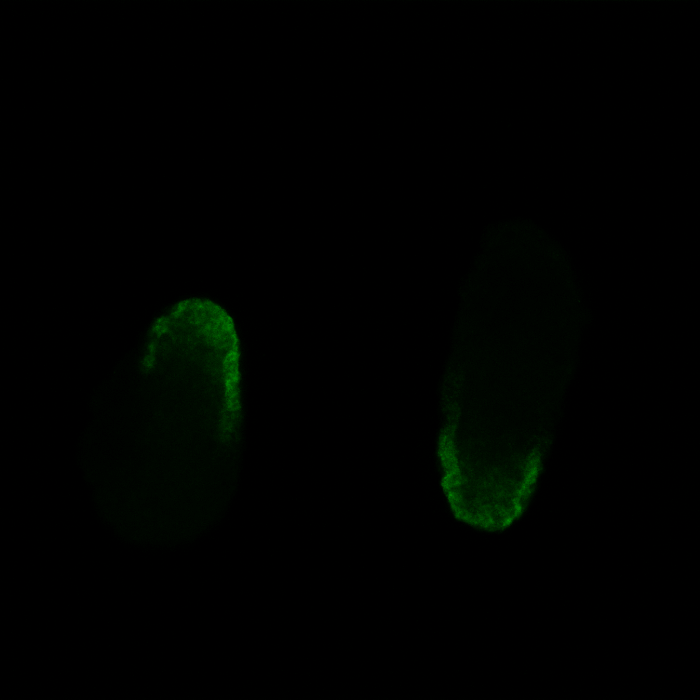

Supplement: Supplementary file 17 — Source data Fig. 1 [file 44318_2025_643_MOESM17_ESM.zip › Figure 1/1F/explant_HCR_tbxta.tif]

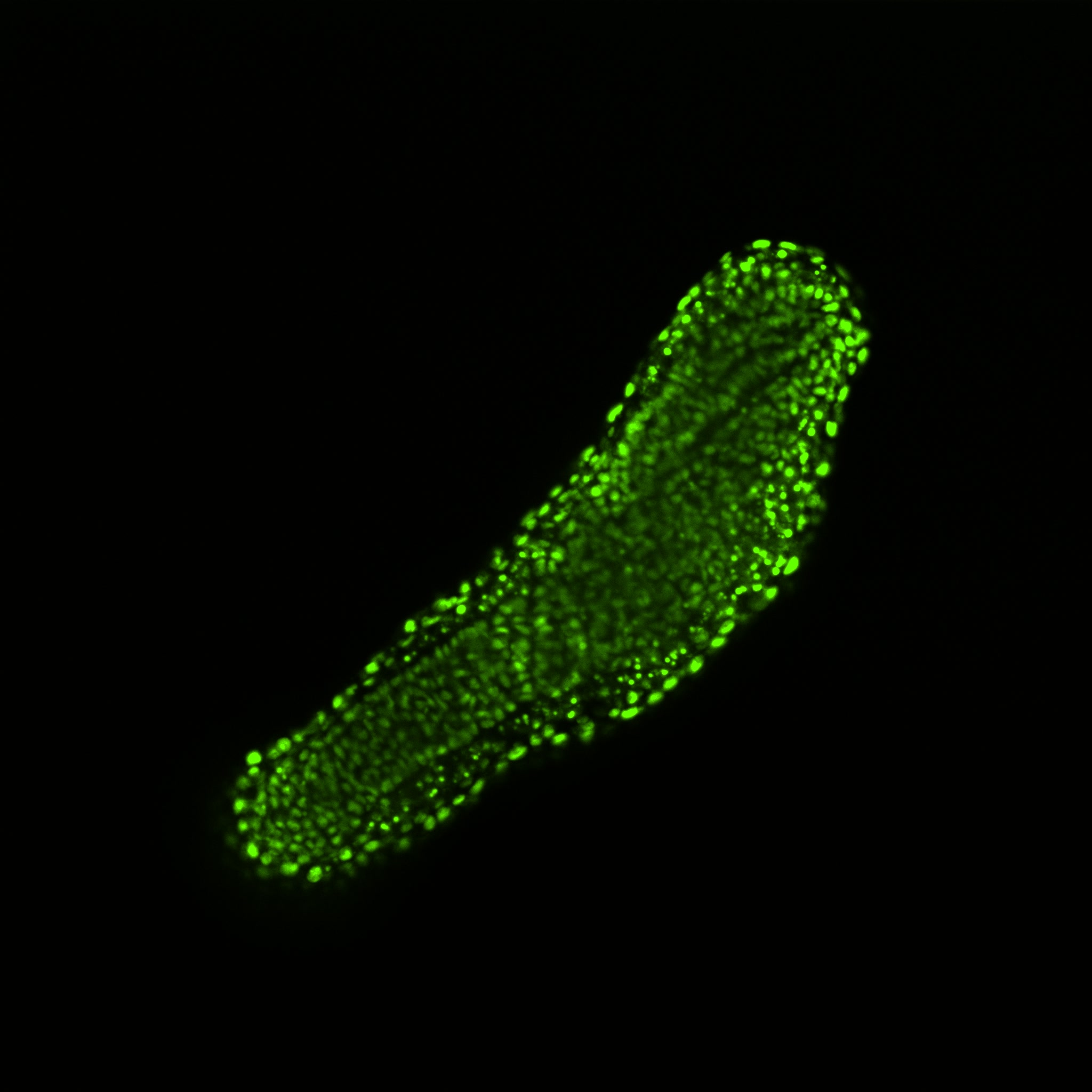

Supplement: Supplementary file 17 — Source data Fig. 1 [file 44318_2025_643_MOESM17_ESM.zip › Figure 1/1G/2s.tif]

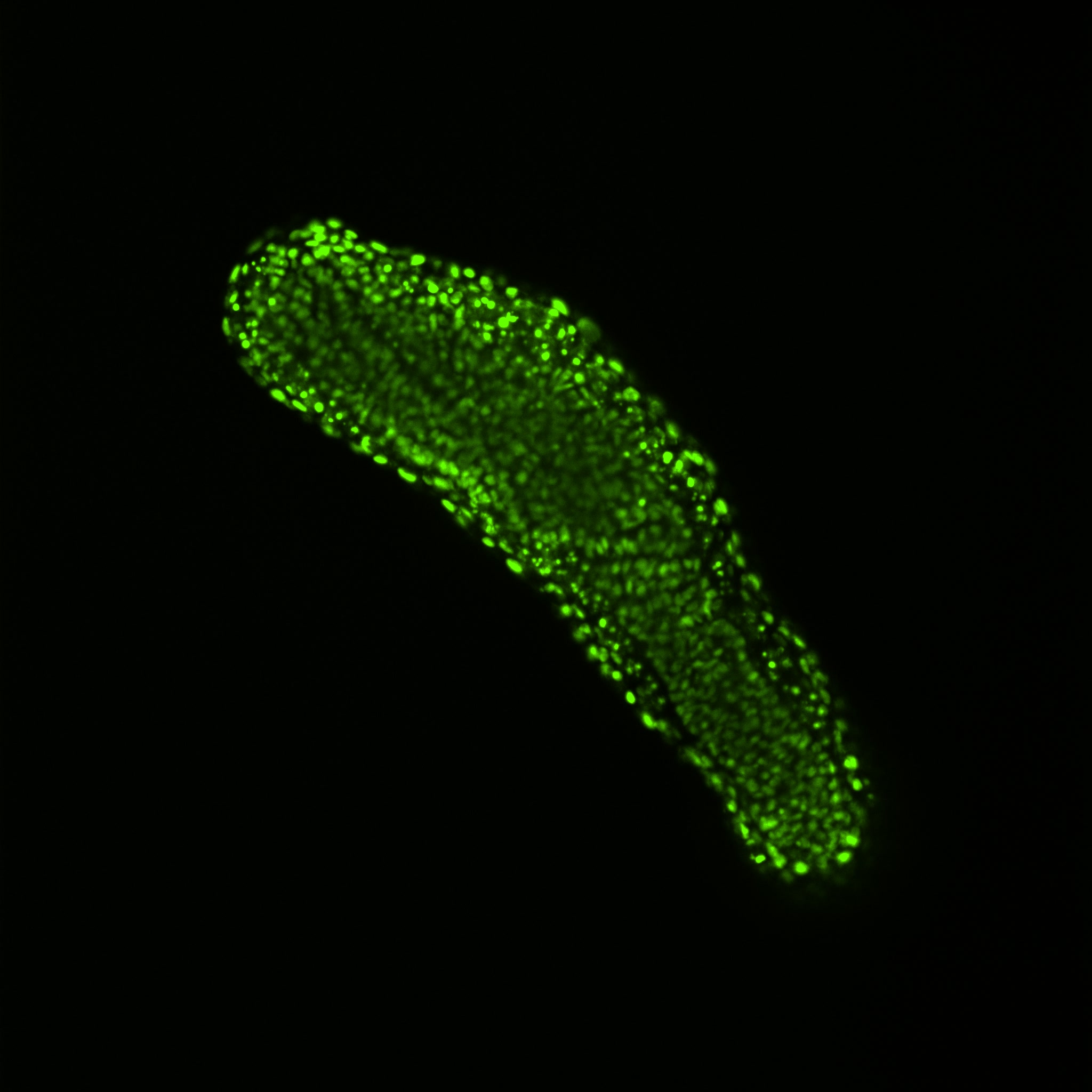

Supplement: Supplementary file 17 — Source data Fig. 1 [file 44318_2025_643_MOESM17_ESM.zip › Figure 1/1G/3s.tif]

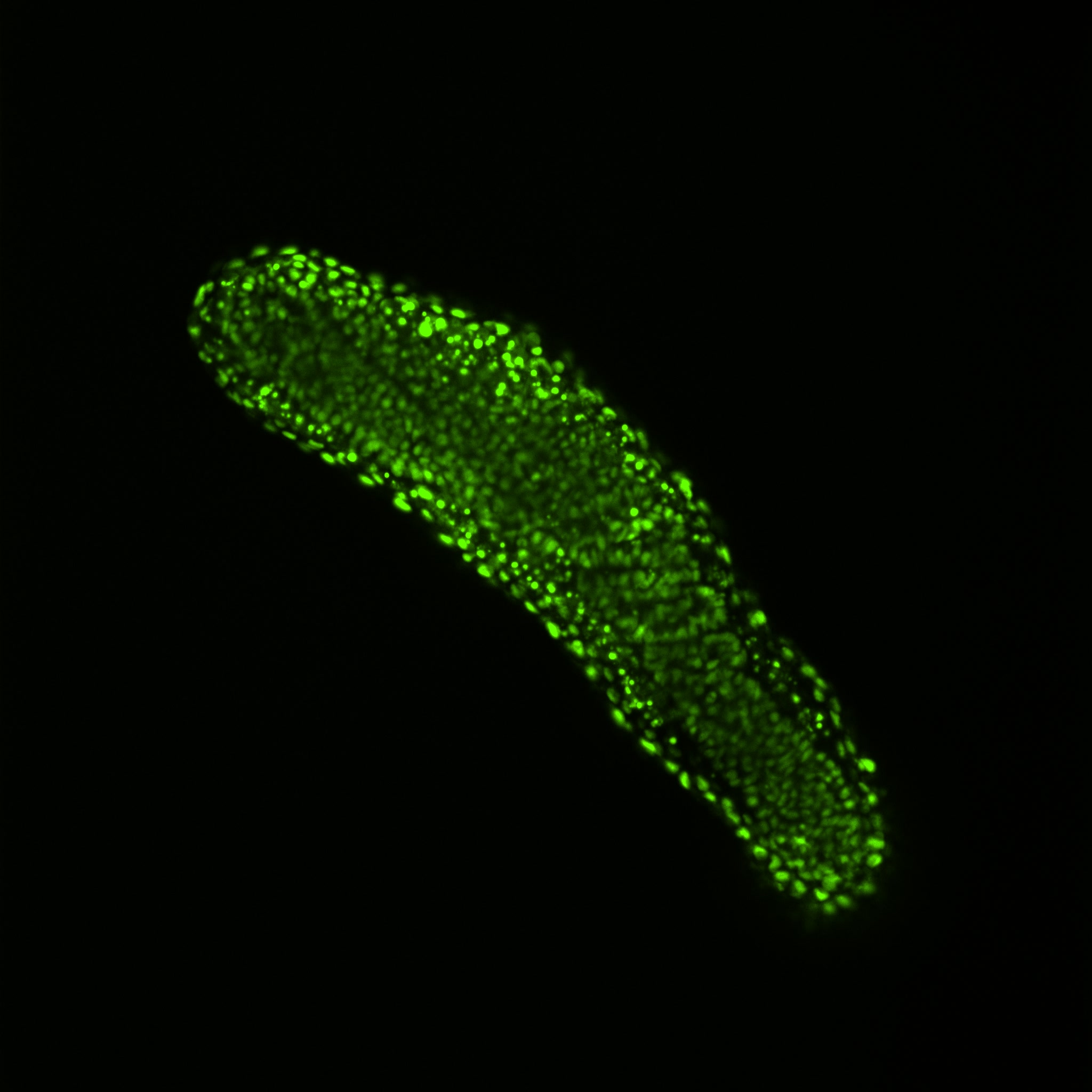

Supplement: Supplementary file 17 — Source data Fig. 1 [file 44318_2025_643_MOESM17_ESM.zip › Figure 1/1G/4s.tif]

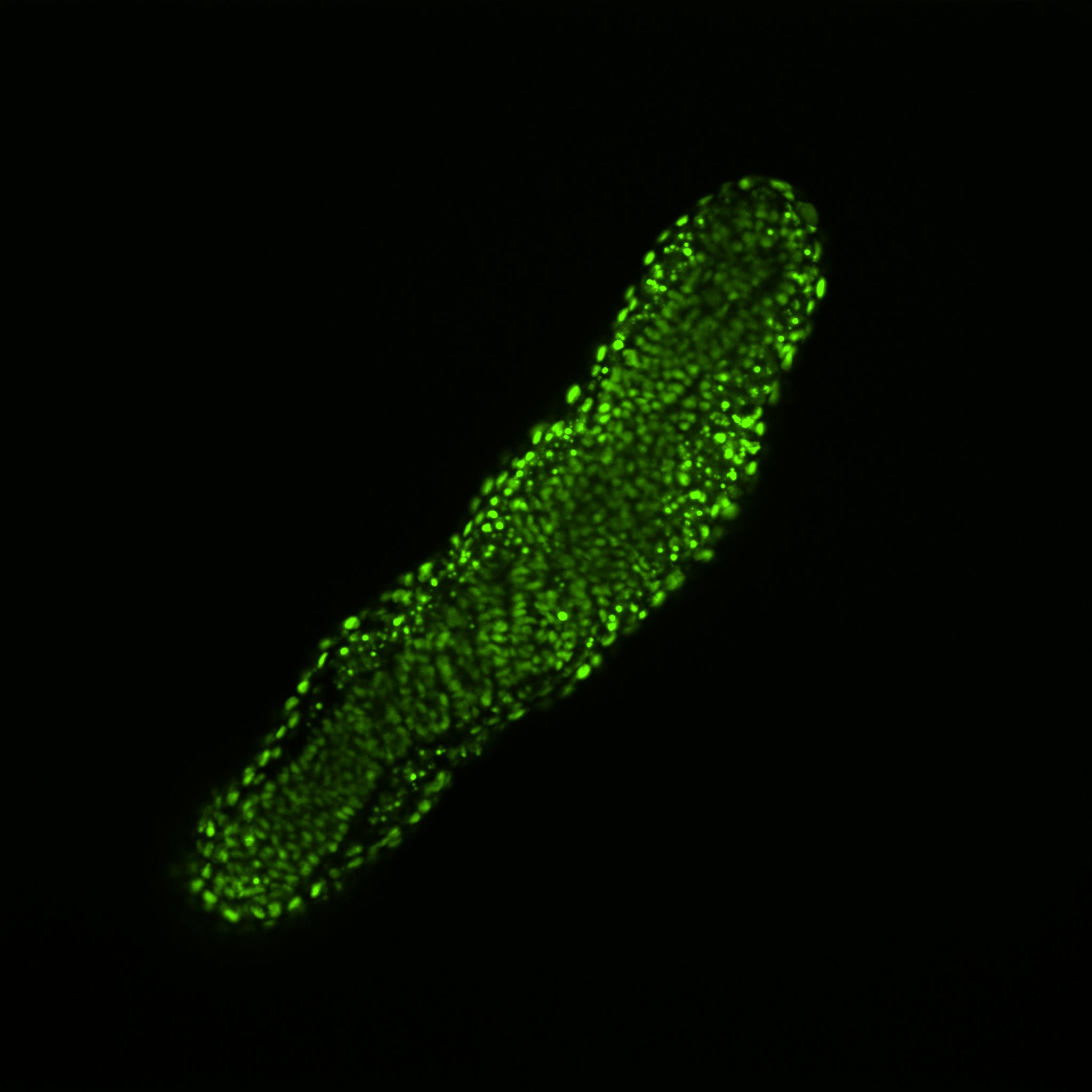

Supplement: Supplementary file 17 — Source data Fig. 1 [file 44318_2025_643_MOESM17_ESM.zip › Figure 1/1G/5s.tif]

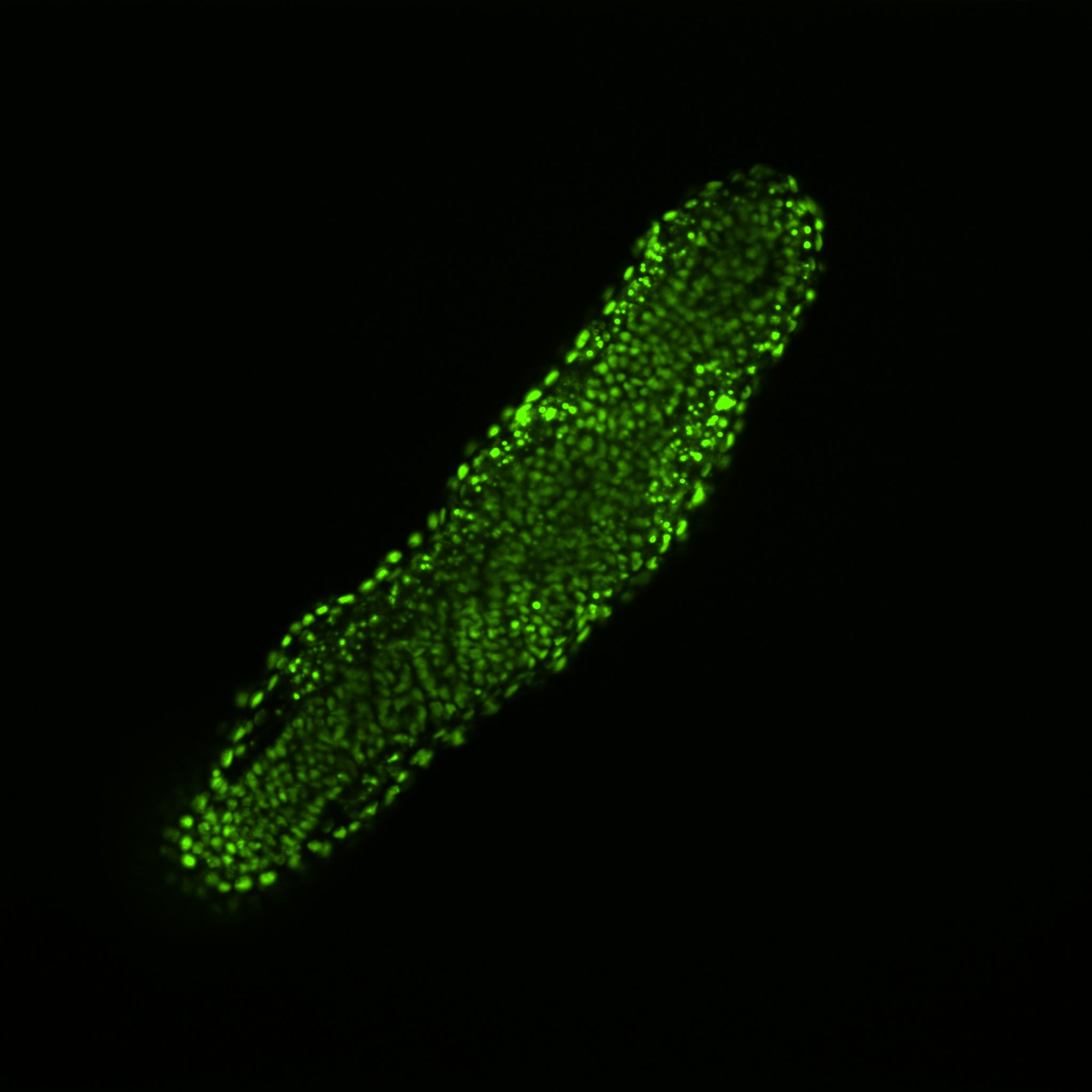

Supplement: Supplementary file 17 — Source data Fig. 1 [file 44318_2025_643_MOESM17_ESM.zip › Figure 1/1G/6s.tif]

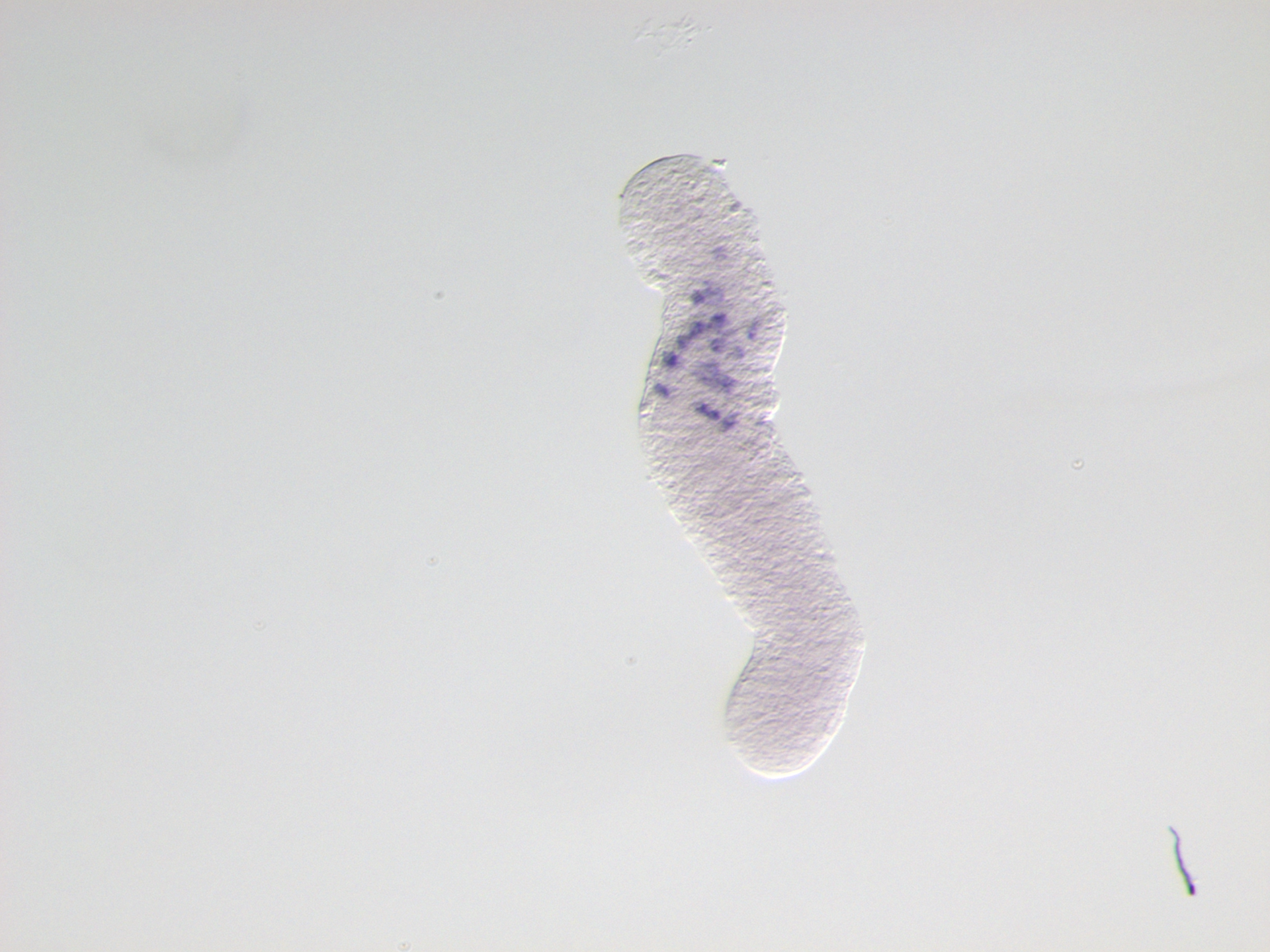

Supplement: Supplementary file 17 — Source data Fig. 1 [file 44318_2025_643_MOESM17_ESM.zip › Figure 1/1H/explant_WISH_gata1a.tif]

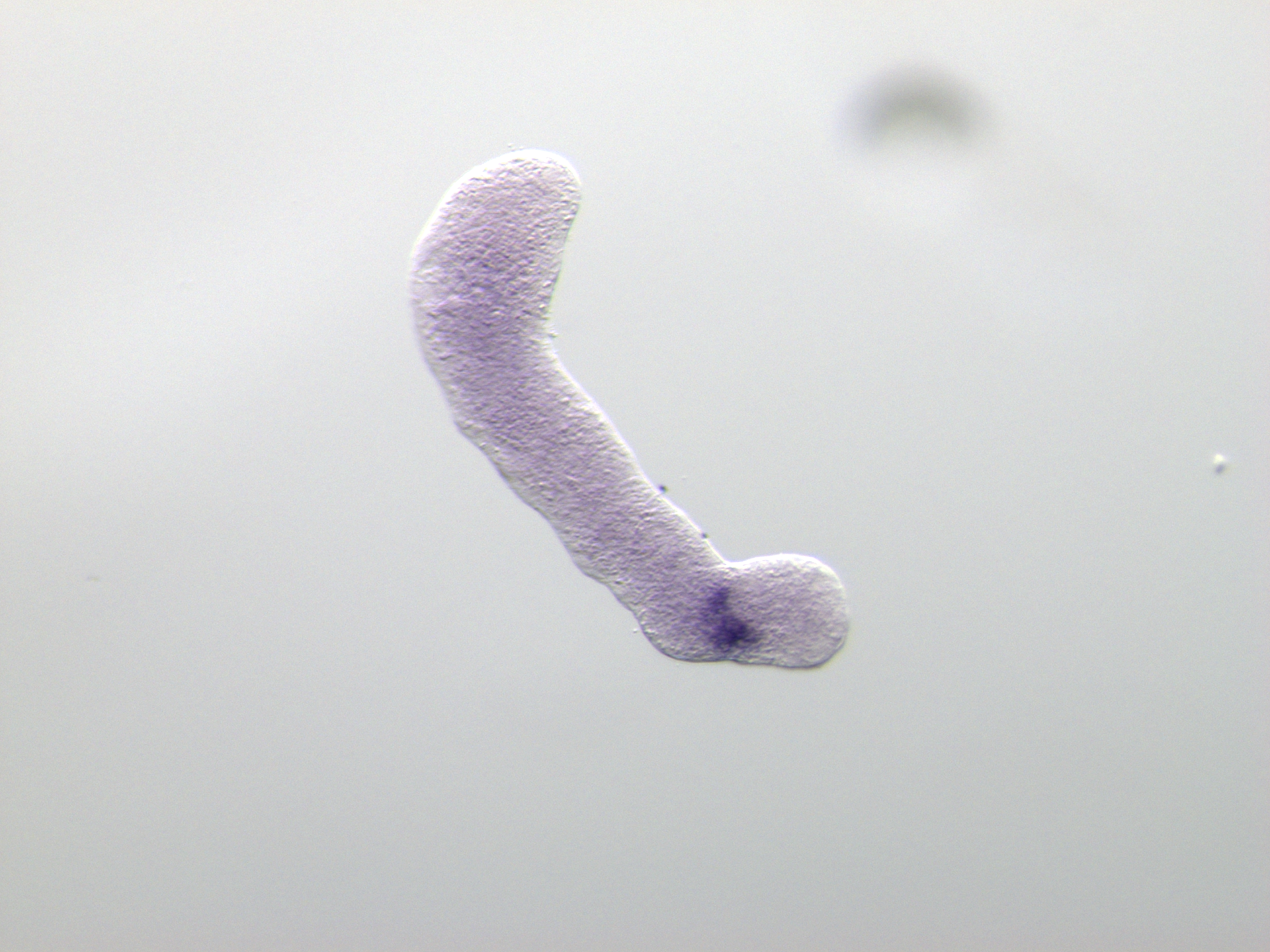

Supplement: Supplementary file 17 — Source data Fig. 1 [file 44318_2025_643_MOESM17_ESM.zip › Figure 1/1H/explant_WISH_gbx1.tif]

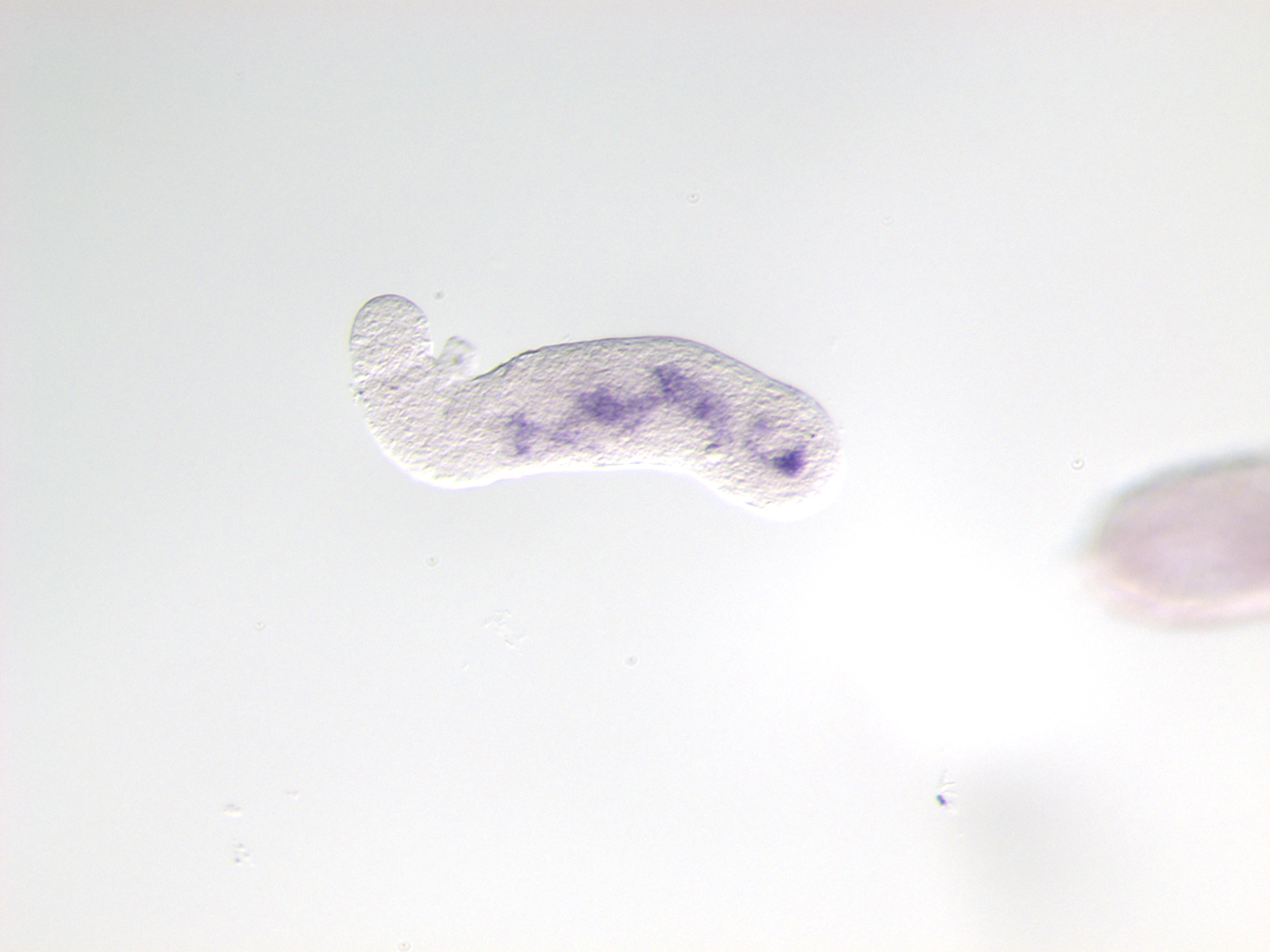

Supplement: Supplementary file 17 — Source data Fig. 1 [file 44318_2025_643_MOESM17_ESM.zip › Figure 1/1H/explant_WISH_kdrl.tif]

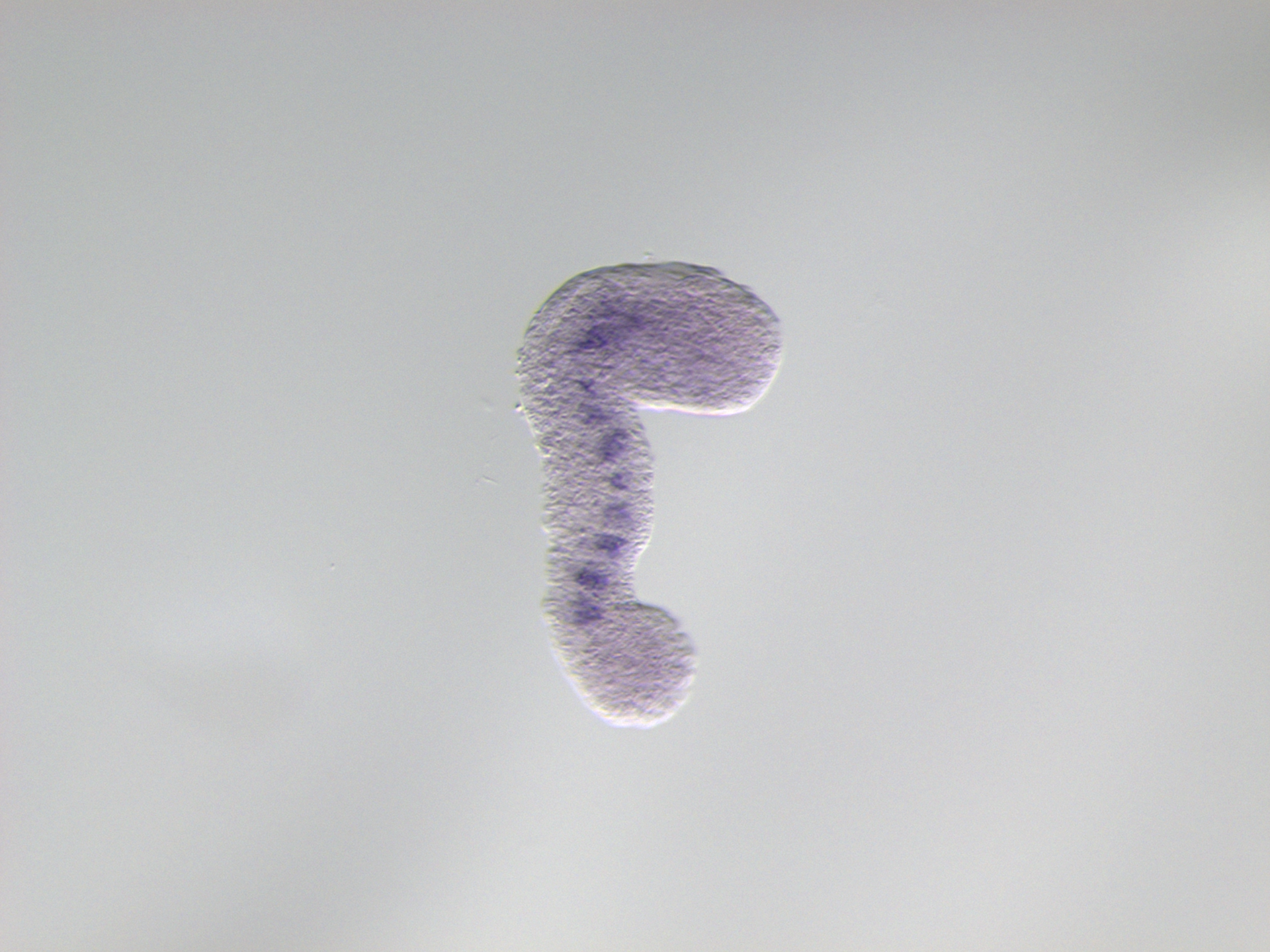

Supplement: Supplementary file 17 — Source data Fig. 1 [file 44318_2025_643_MOESM17_ESM.zip › Figure 1/1H/explant_WISH_myod1.tif]

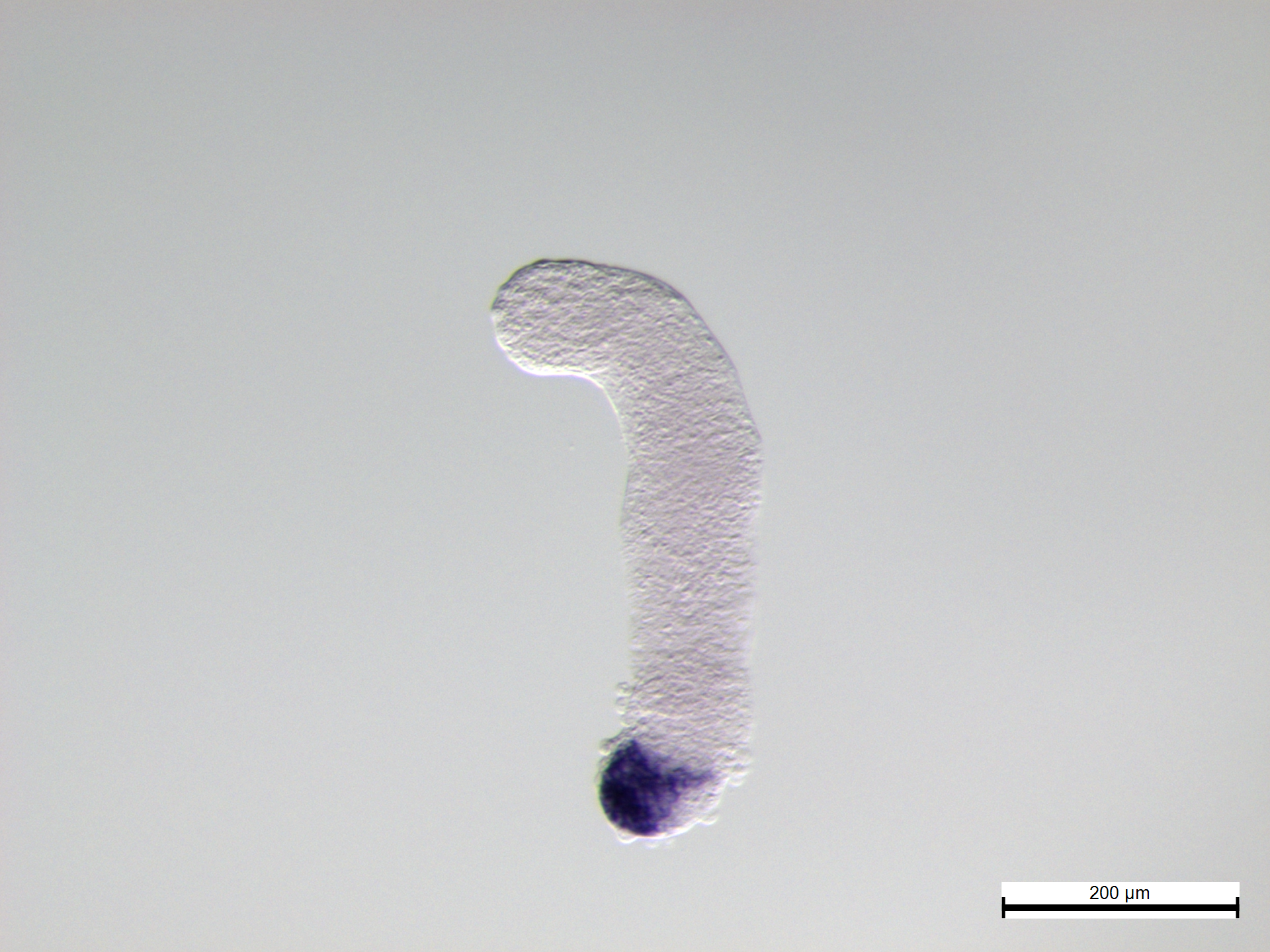

Supplement: Supplementary file 17 — Source data Fig. 1 [file 44318_2025_643_MOESM17_ESM.zip › Figure 1/1H/explant_WISH_tbxta.tif]

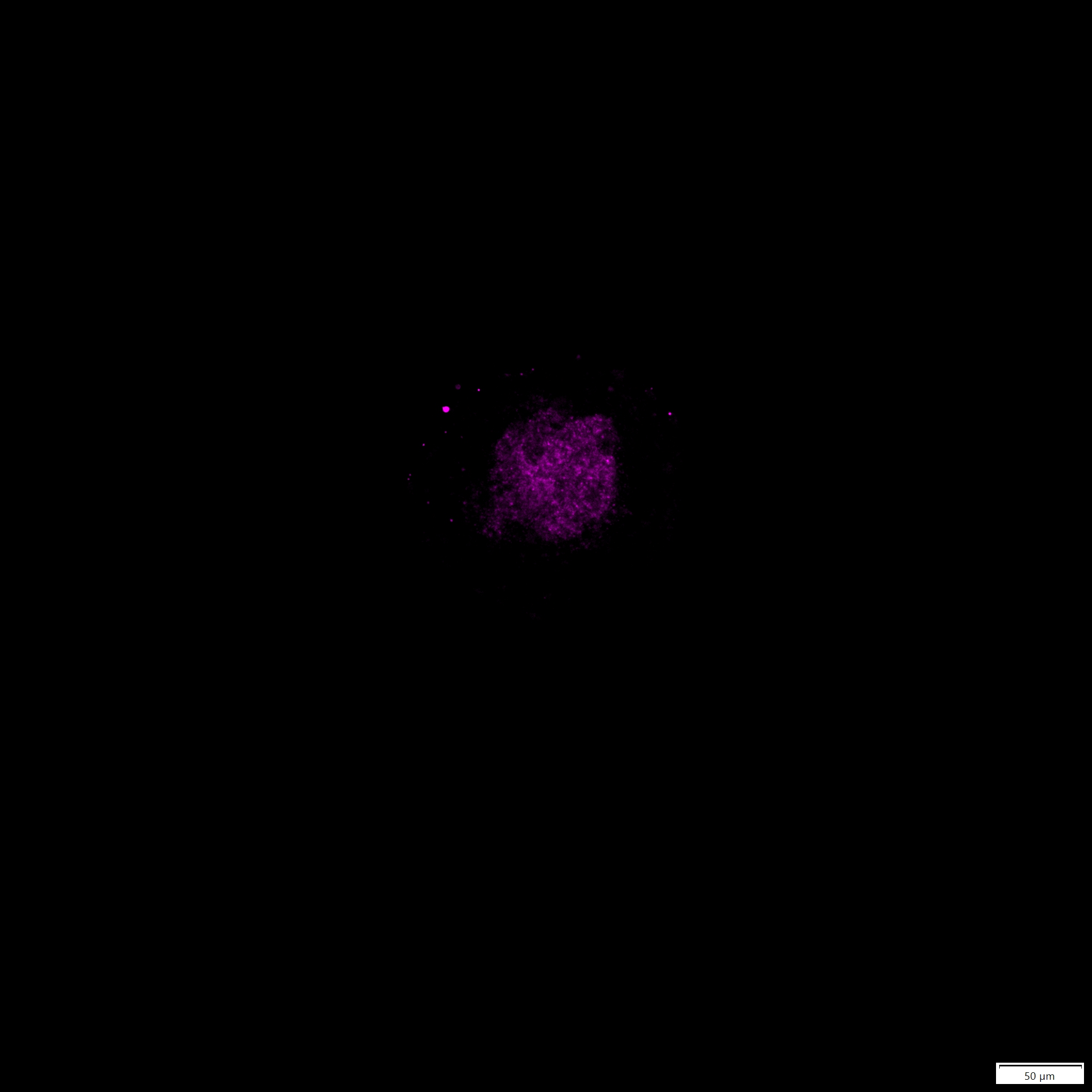

Supplement: Supplementary file 18 — Source data Fig. 2 [file 44318_2025_643_MOESM18_ESM.zip › Figure 2/2D/bmp4 explant_HCR_aldh1a2_sagittal plane.jpg]

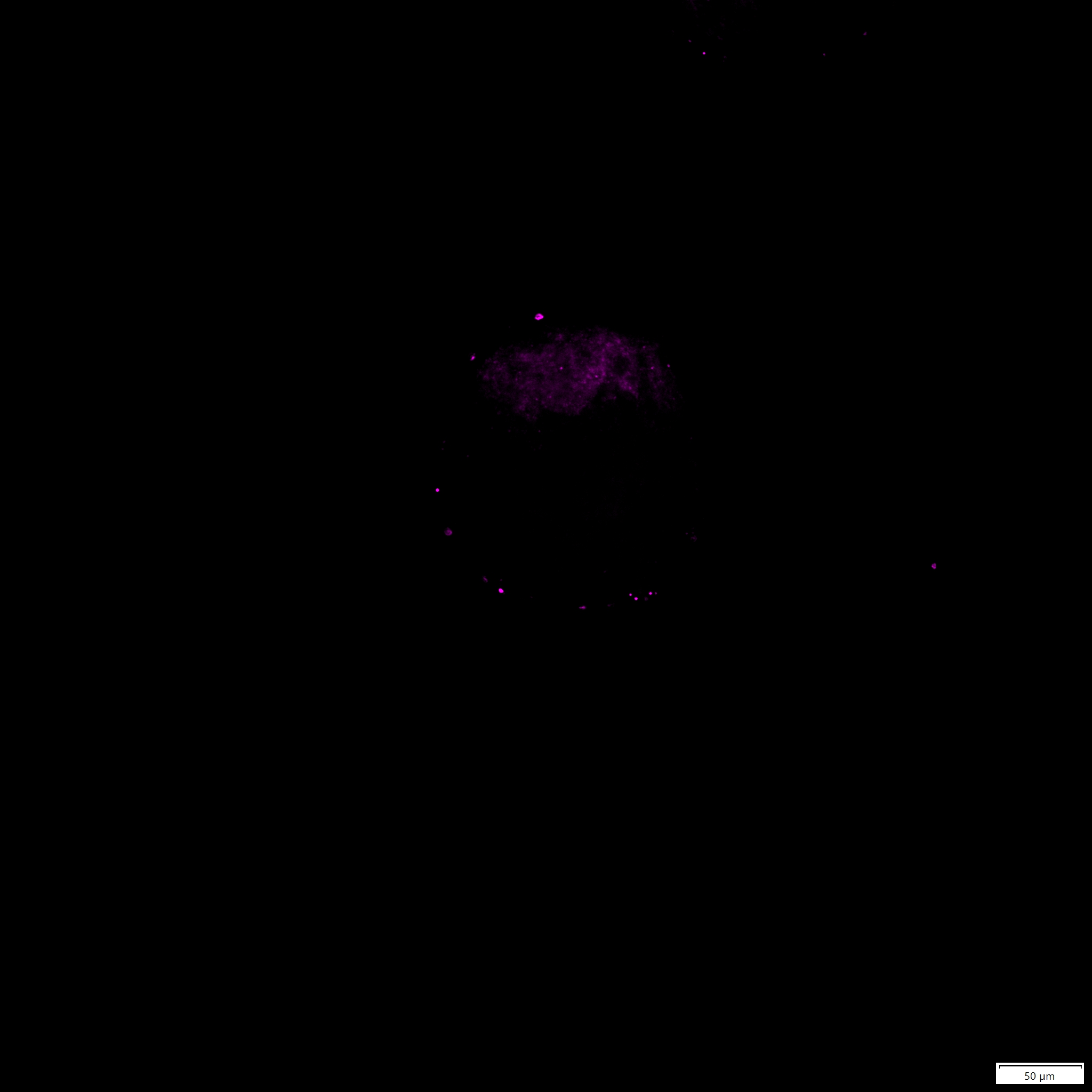

Supplement: Supplementary file 18 — Source data Fig. 2 [file 44318_2025_643_MOESM18_ESM.zip › Figure 2/2D/bmp4 explant_HCR_aldh1a2_transverse plane.jpg]

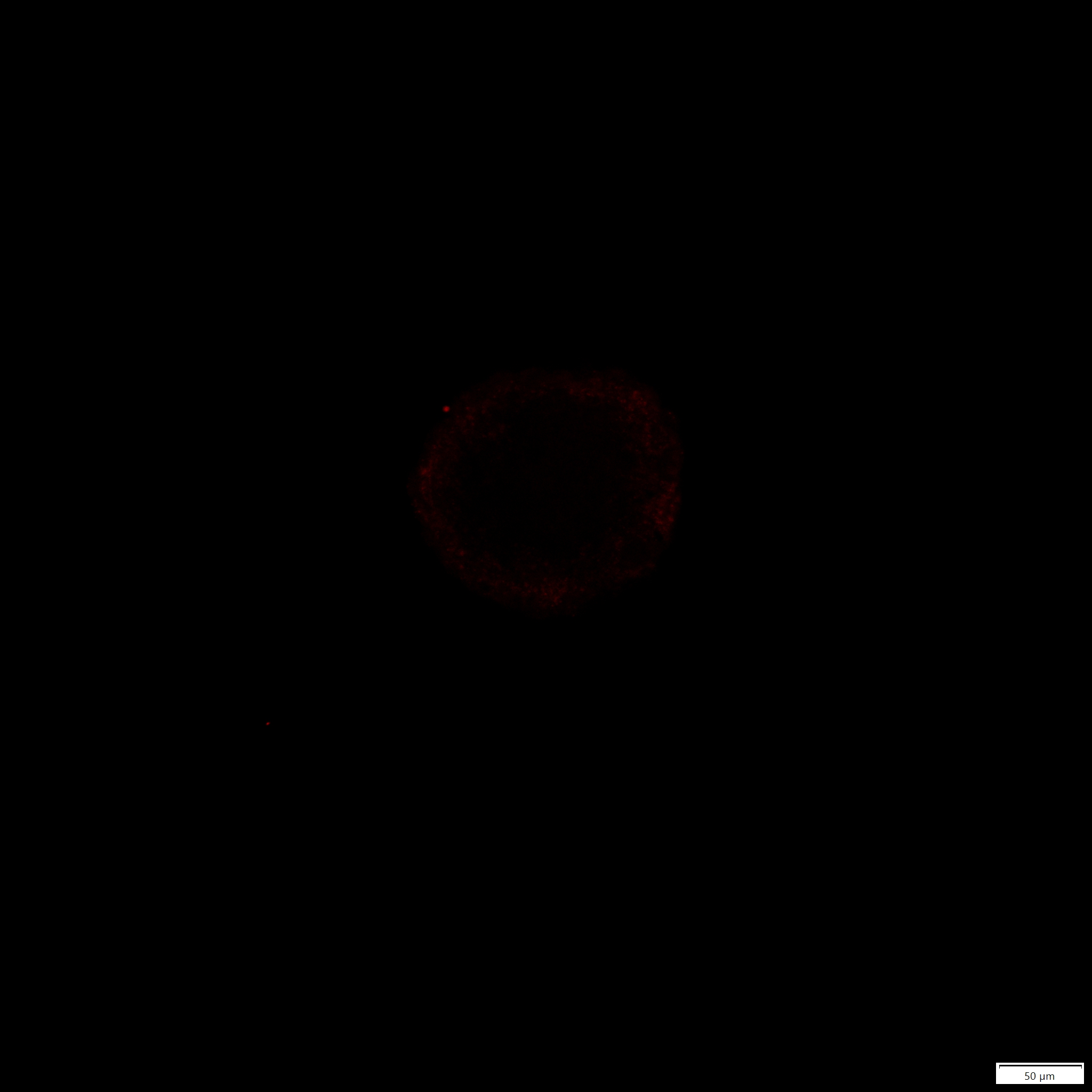

Supplement: Supplementary file 18 — Source data Fig. 2 [file 44318_2025_643_MOESM18_ESM.zip › Figure 2/2D/bmp4 explant_HCR_sox2_sagittal plane.jpg]

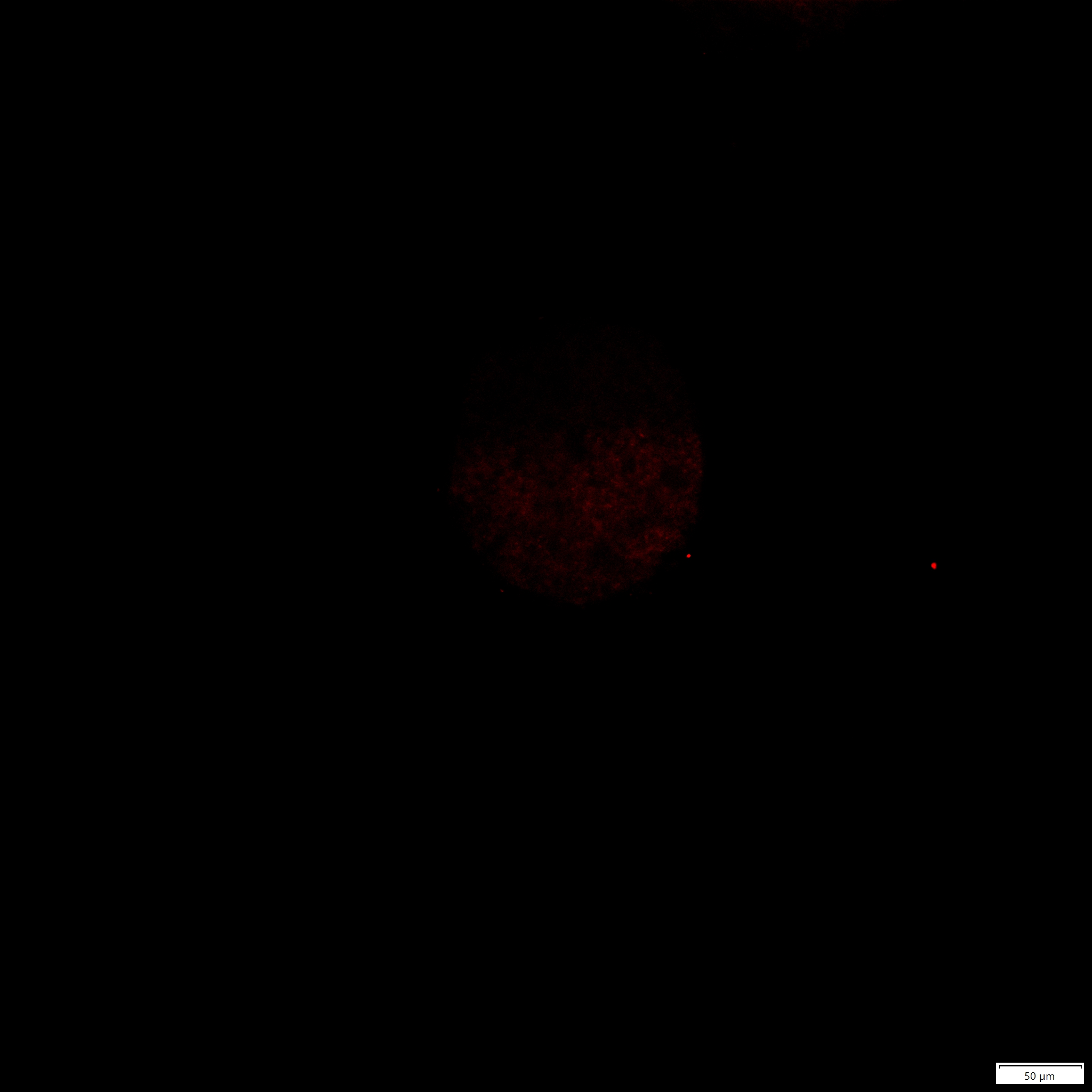

Supplement: Supplementary file 18 — Source data Fig. 2 [file 44318_2025_643_MOESM18_ESM.zip › Figure 2/2D/bmp4 explant_HCR_sox2_transverse plane.jpg]

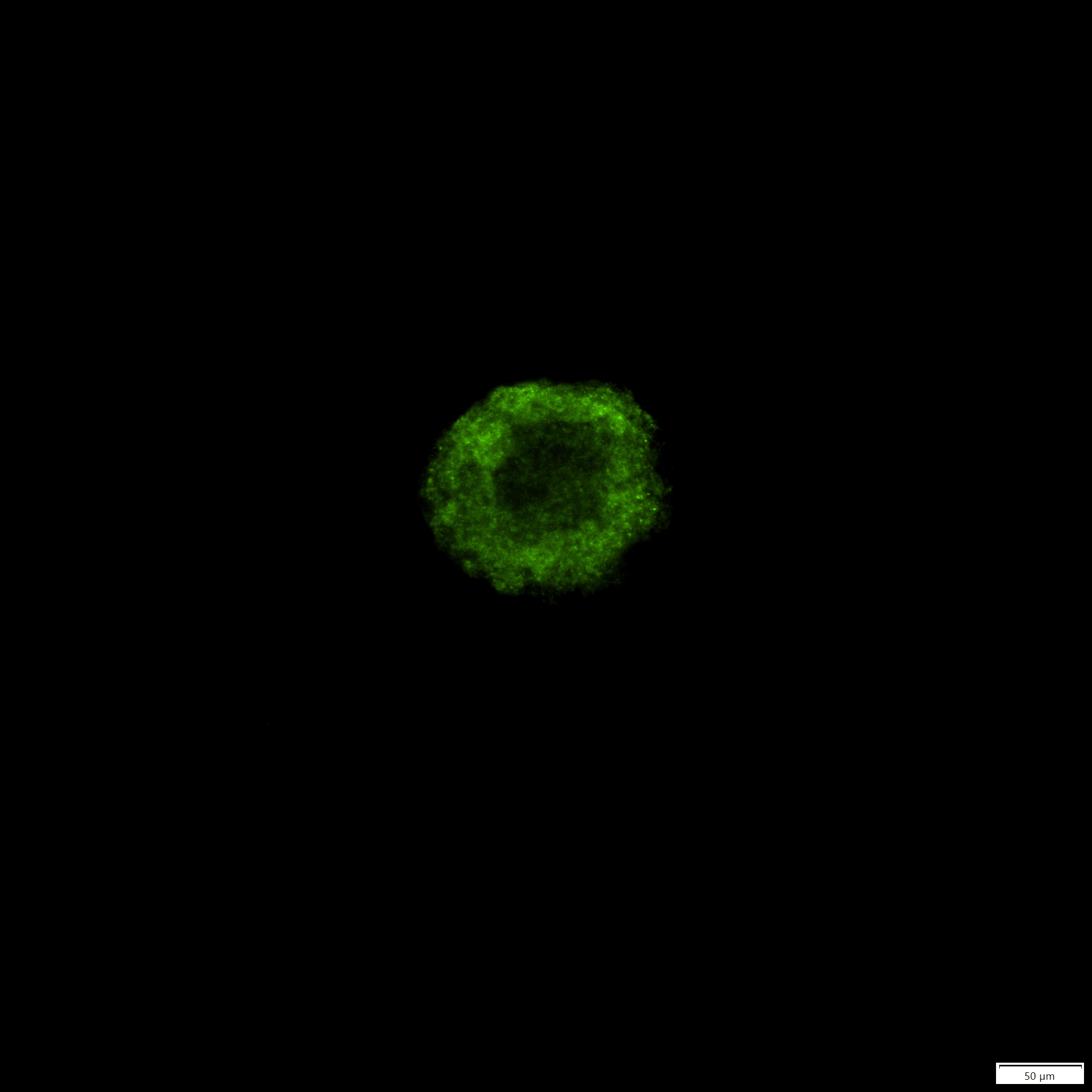

Supplement: Supplementary file 18 — Source data Fig. 2 [file 44318_2025_643_MOESM18_ESM.zip › Figure 2/2D/bmp4 explant_HCR_tbxta_sagittal plane.jpg]

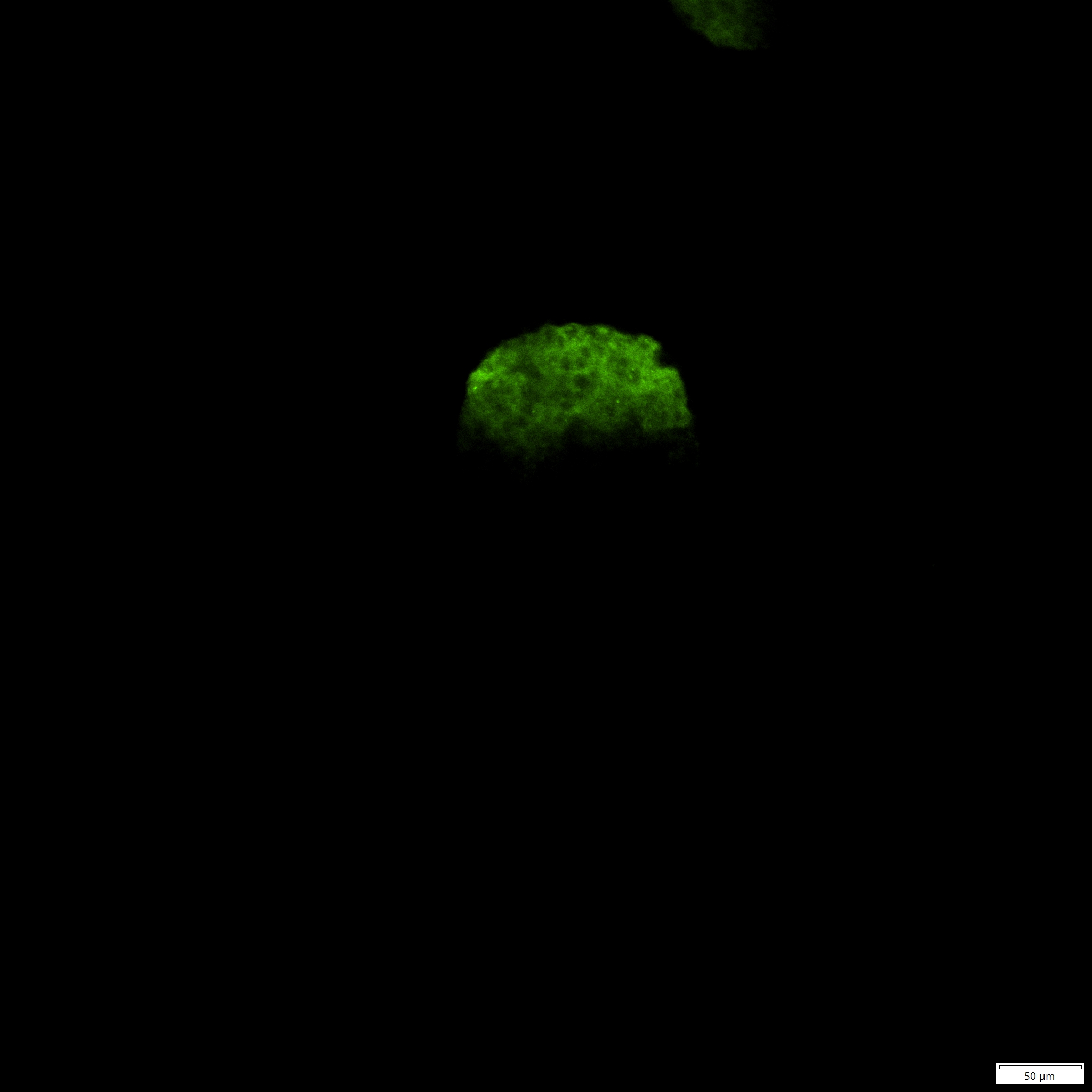

Supplement: Supplementary file 18 — Source data Fig. 2 [file 44318_2025_643_MOESM18_ESM.zip › Figure 2/2D/bmp4 explant_HCR_tbxta_transverse plane.jpg]

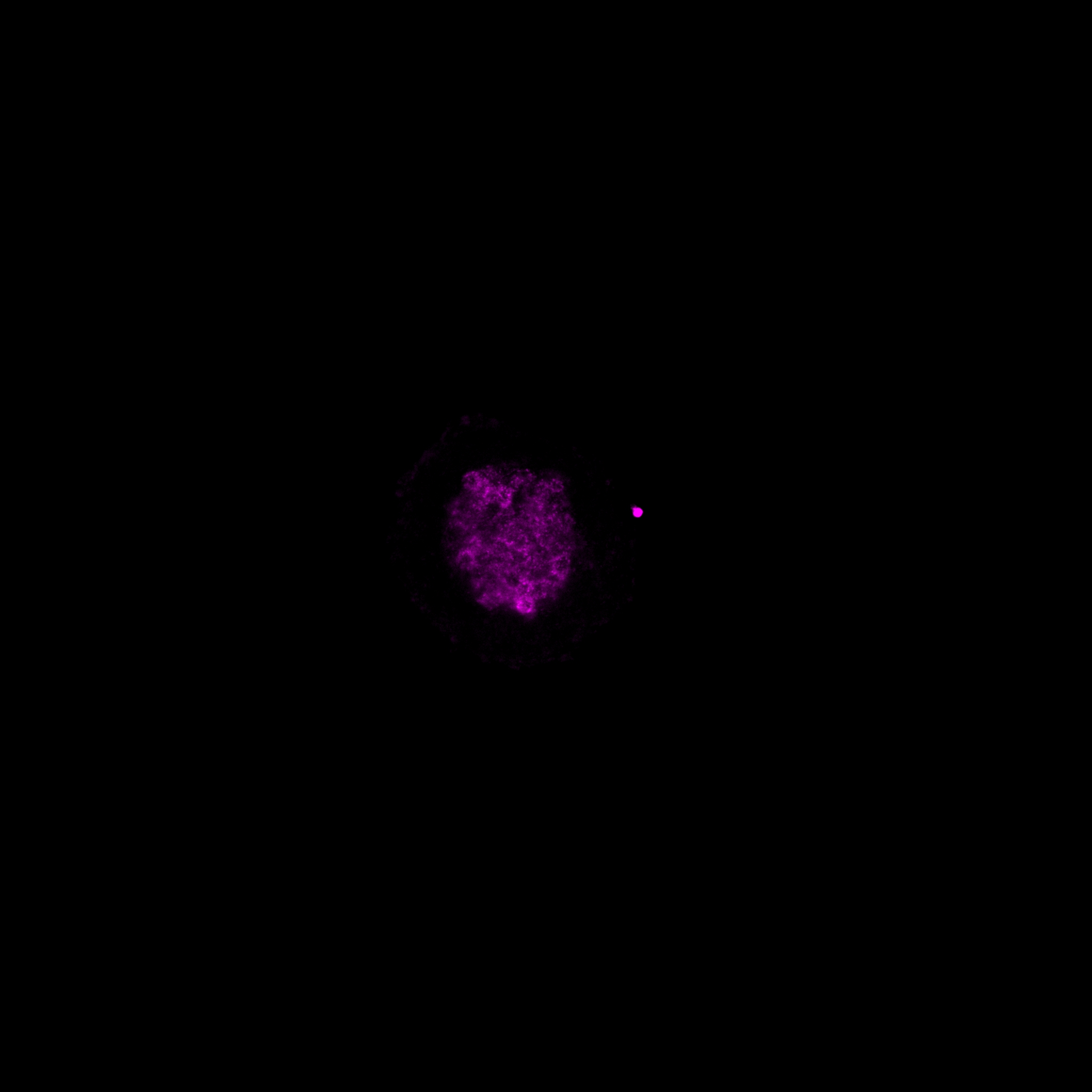

Supplement: Supplementary file 18 — Source data Fig. 2 [file 44318_2025_643_MOESM18_ESM.zip › Figure 2/2F/bmp4 explant_HCR_aldh1a2_sagittal plane.jpg]

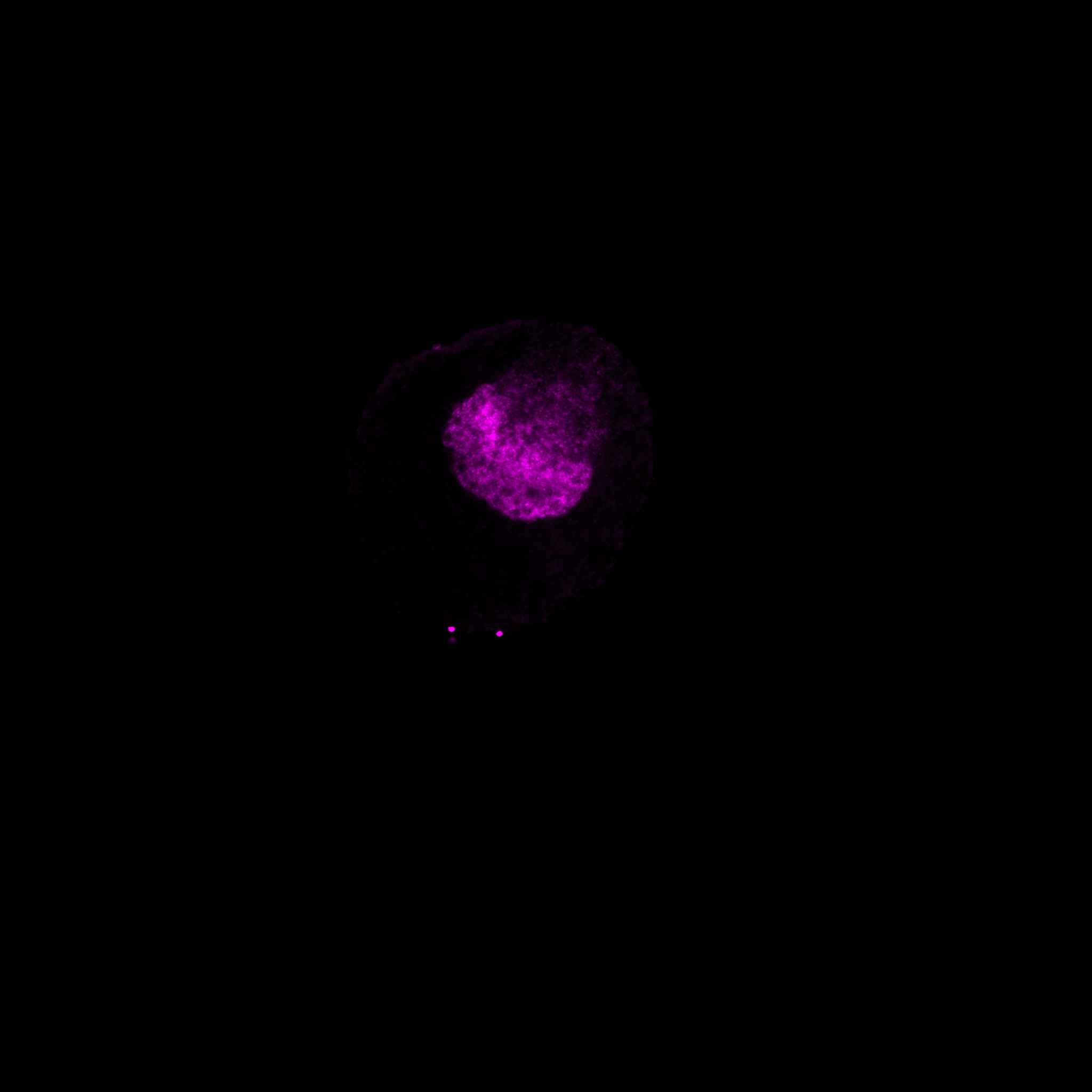

Supplement: Supplementary file 18 — Source data Fig. 2 [file 44318_2025_643_MOESM18_ESM.zip › Figure 2/2F/bmp4 explant_HCR_aldh1a2_transverse plane.jpg]

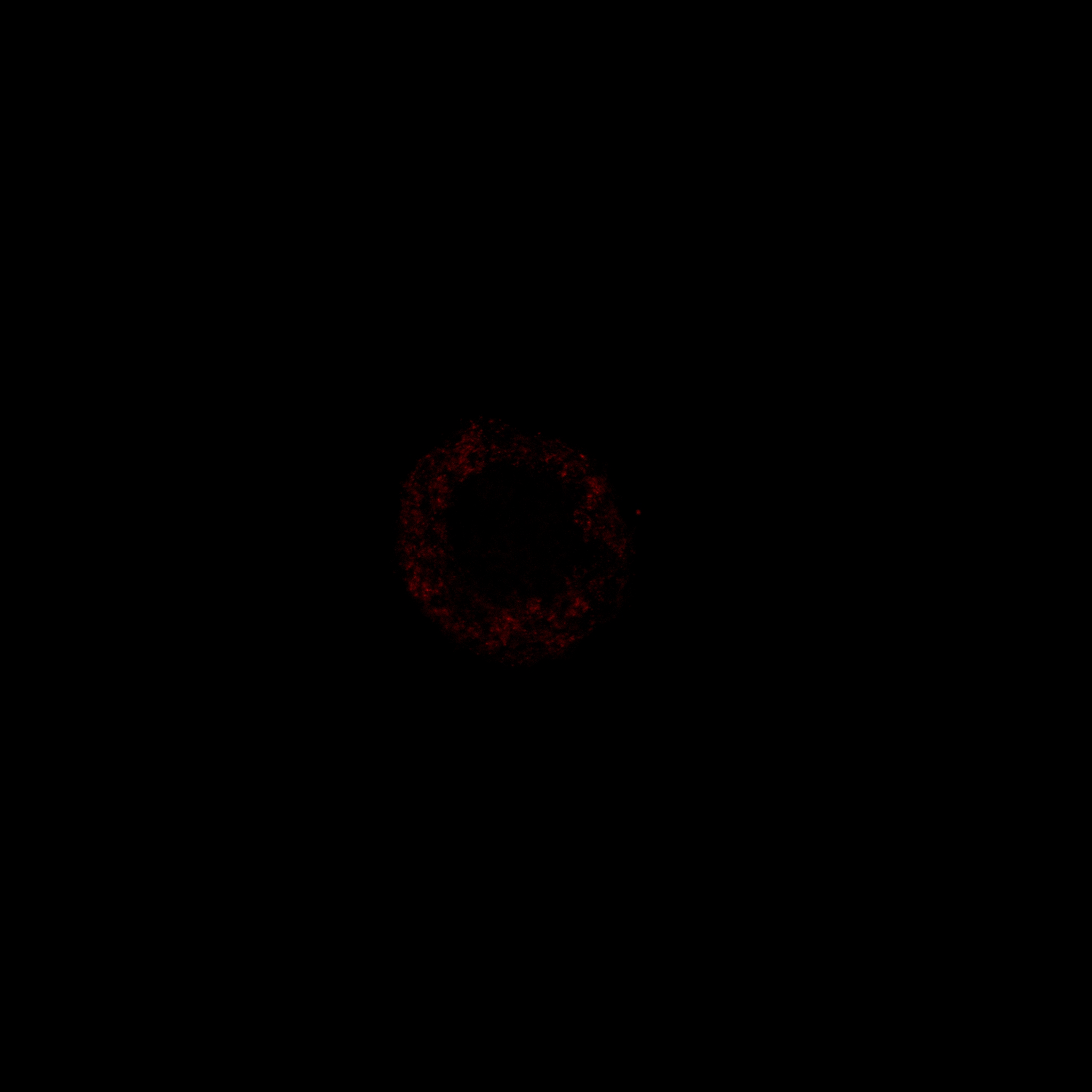

Supplement: Supplementary file 18 — Source data Fig. 2 [file 44318_2025_643_MOESM18_ESM.zip › Figure 2/2F/bmp4 explant_HCR_sox2_sagittal plane.jpg]

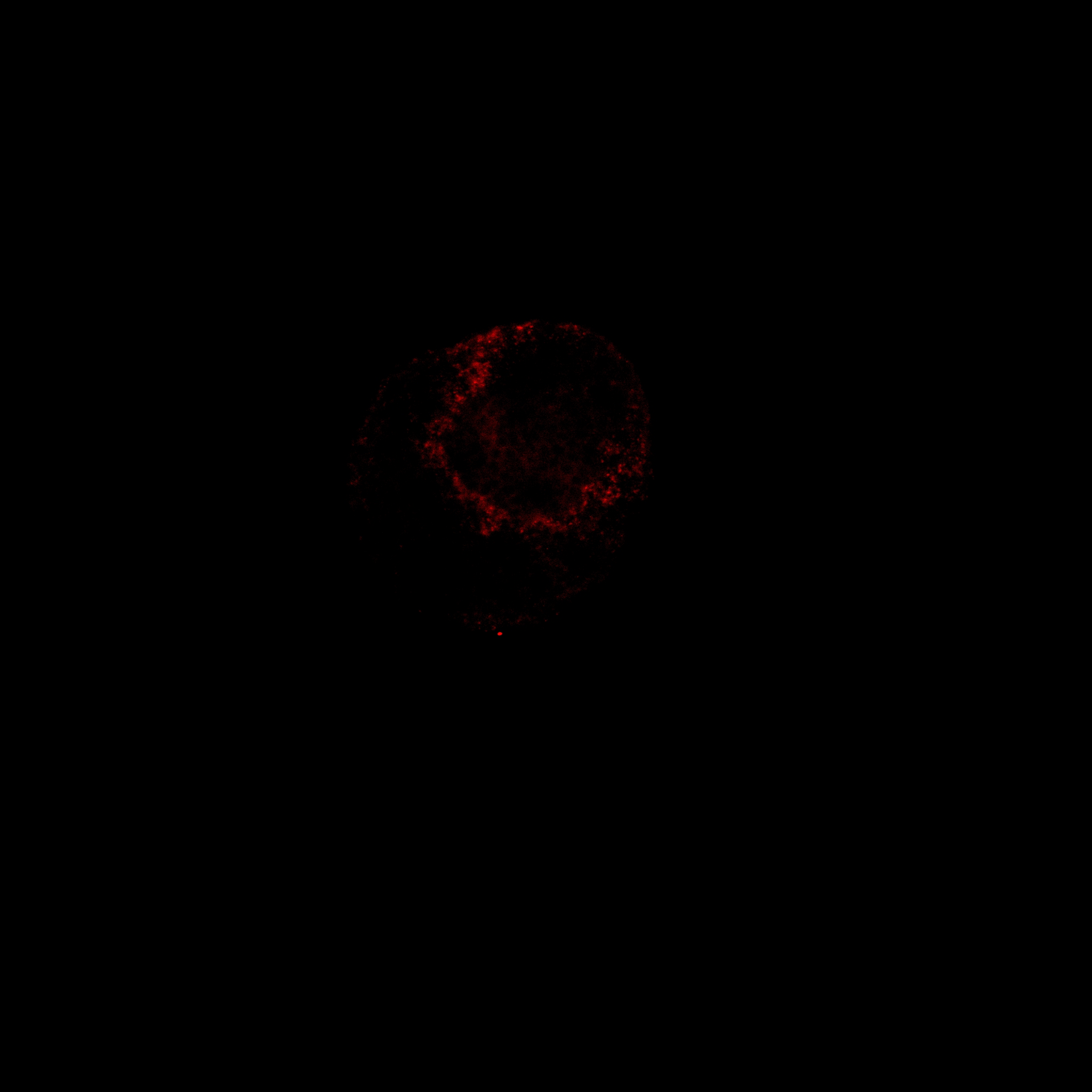

Supplement: Supplementary file 18 — Source data Fig. 2 [file 44318_2025_643_MOESM18_ESM.zip › Figure 2/2F/bmp4 explant_HCR_sox2_transverse plane.jpg]

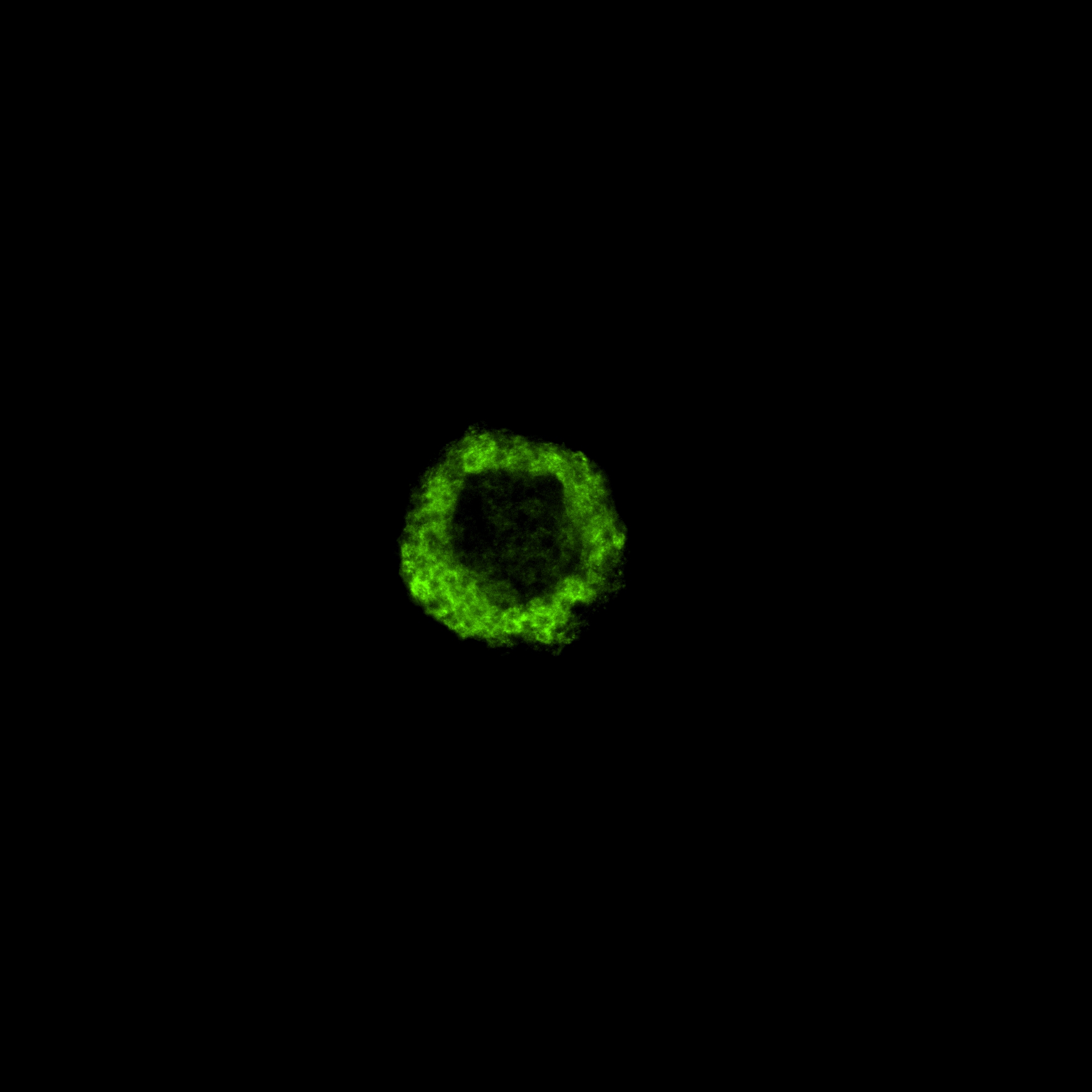

Supplement: Supplementary file 18 — Source data Fig. 2 [file 44318_2025_643_MOESM18_ESM.zip › Figure 2/2F/bmp4 explant_HCR_tbxta_sagittal plane.jpg]

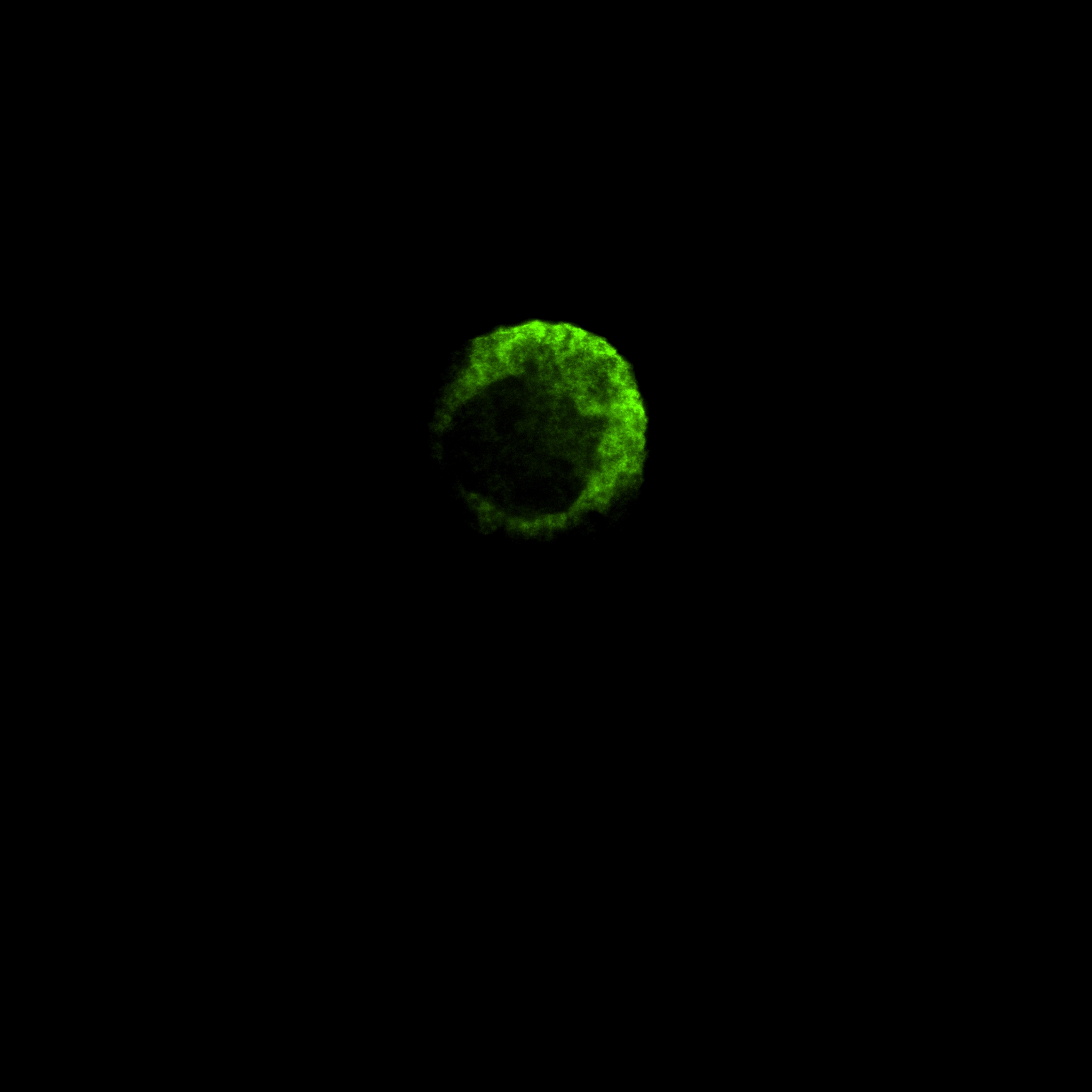

Supplement: Supplementary file 18 — Source data Fig. 2 [file 44318_2025_643_MOESM18_ESM.zip › Figure 2/2F/bmp4 explant_HCR_tbxta_transverse plane.jpg]

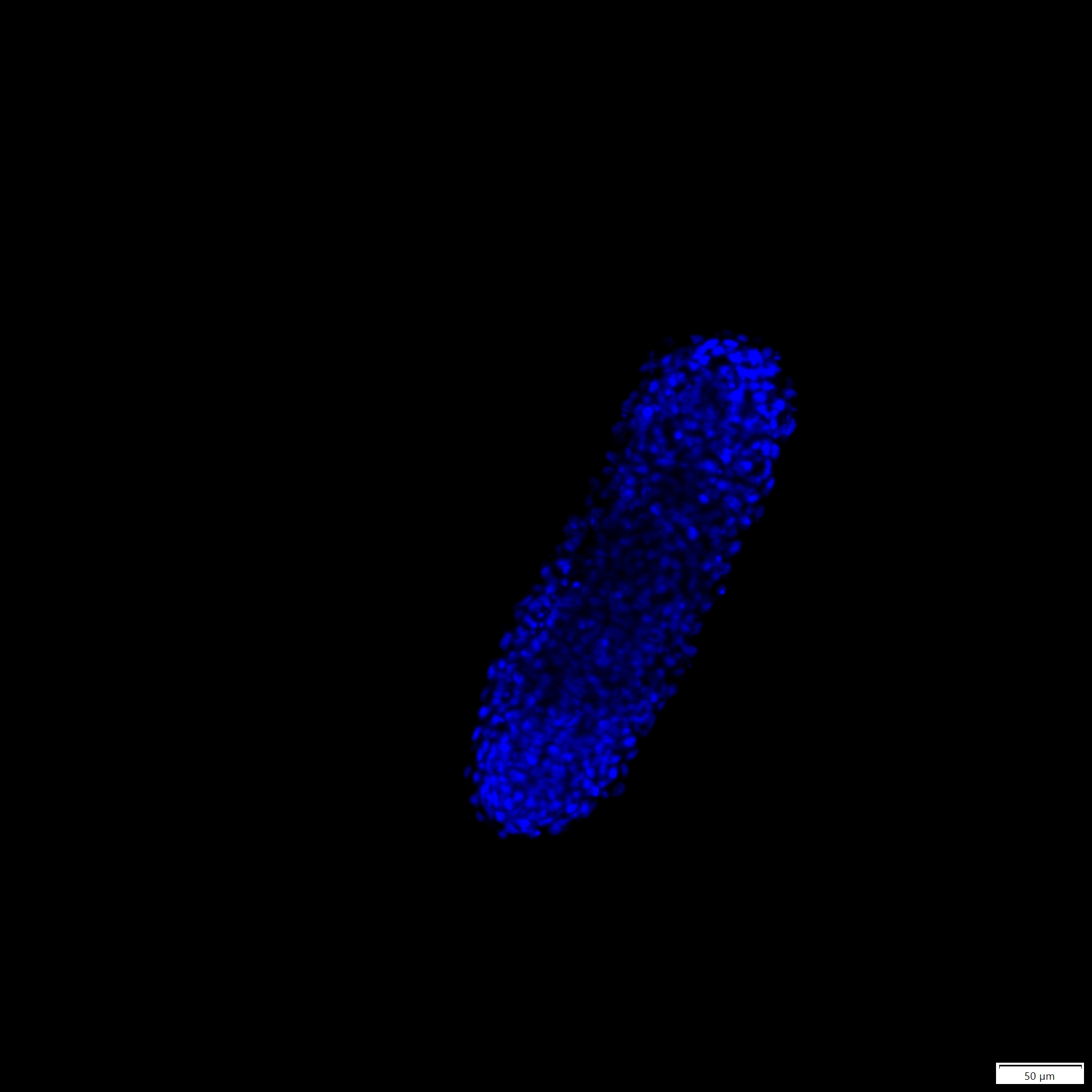

Supplement: Supplementary file 18 — Source data Fig. 2 [file 44318_2025_643_MOESM18_ESM.zip › Figure 2/2H/bmp4 explant_DAPI.jpg]

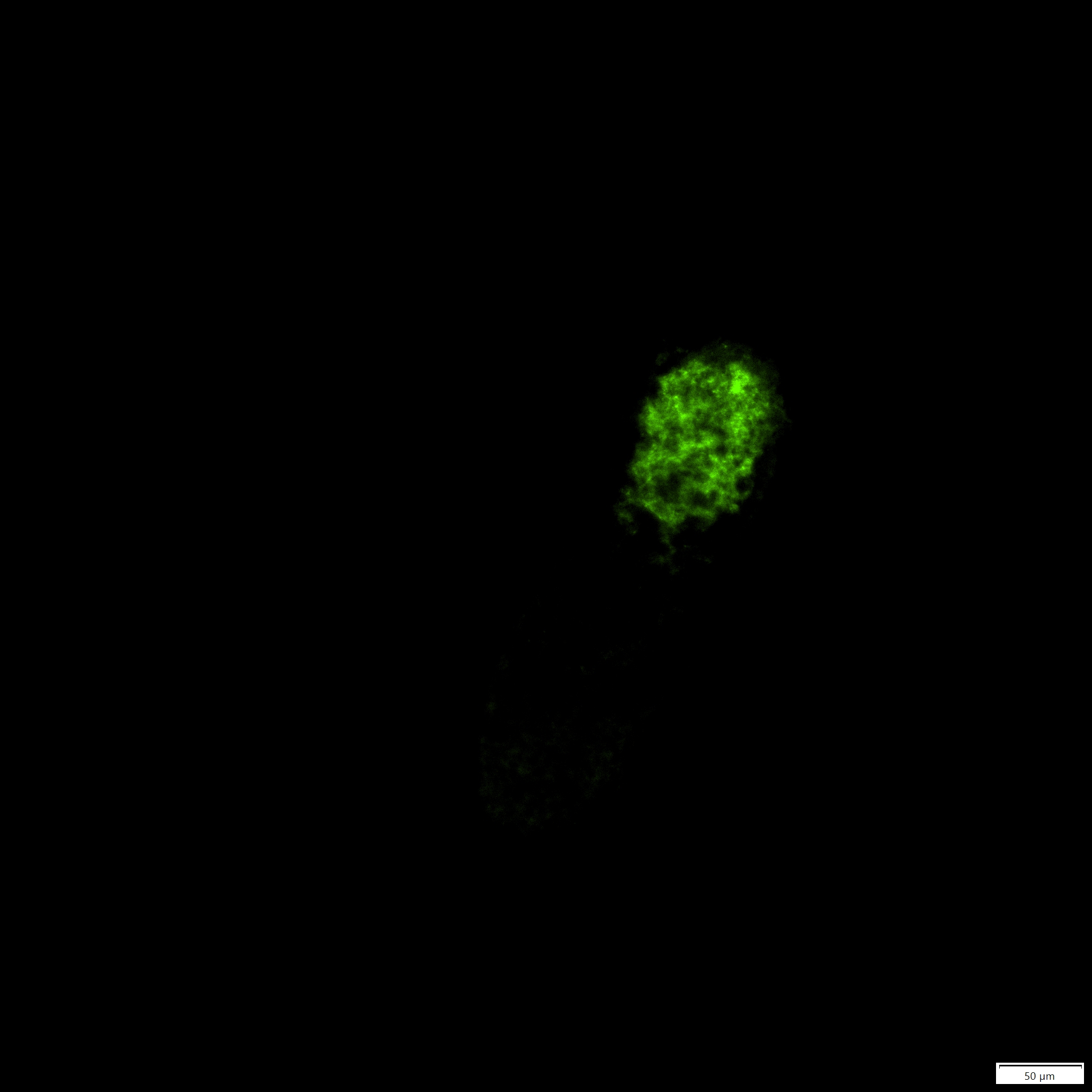

Supplement: Supplementary file 18 — Source data Fig. 2 [file 44318_2025_643_MOESM18_ESM.zip › Figure 2/2H/bmp4 explant_HCR_ripply1.jpg]

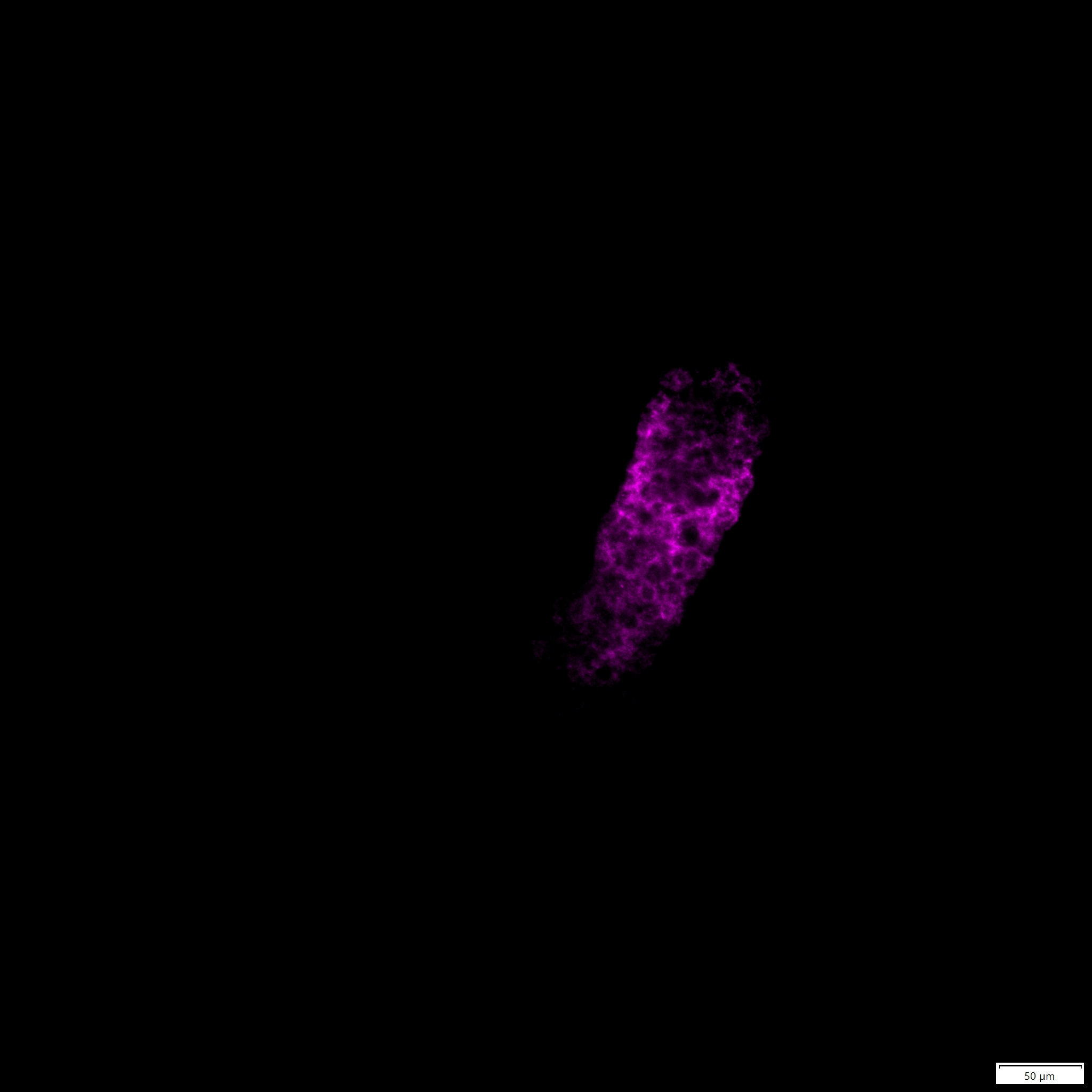

Supplement: Supplementary file 18 — Source data Fig. 2 [file 44318_2025_643_MOESM18_ESM.zip › Figure 2/2H/bmp4 explant_HCR_tbx6.jpg]

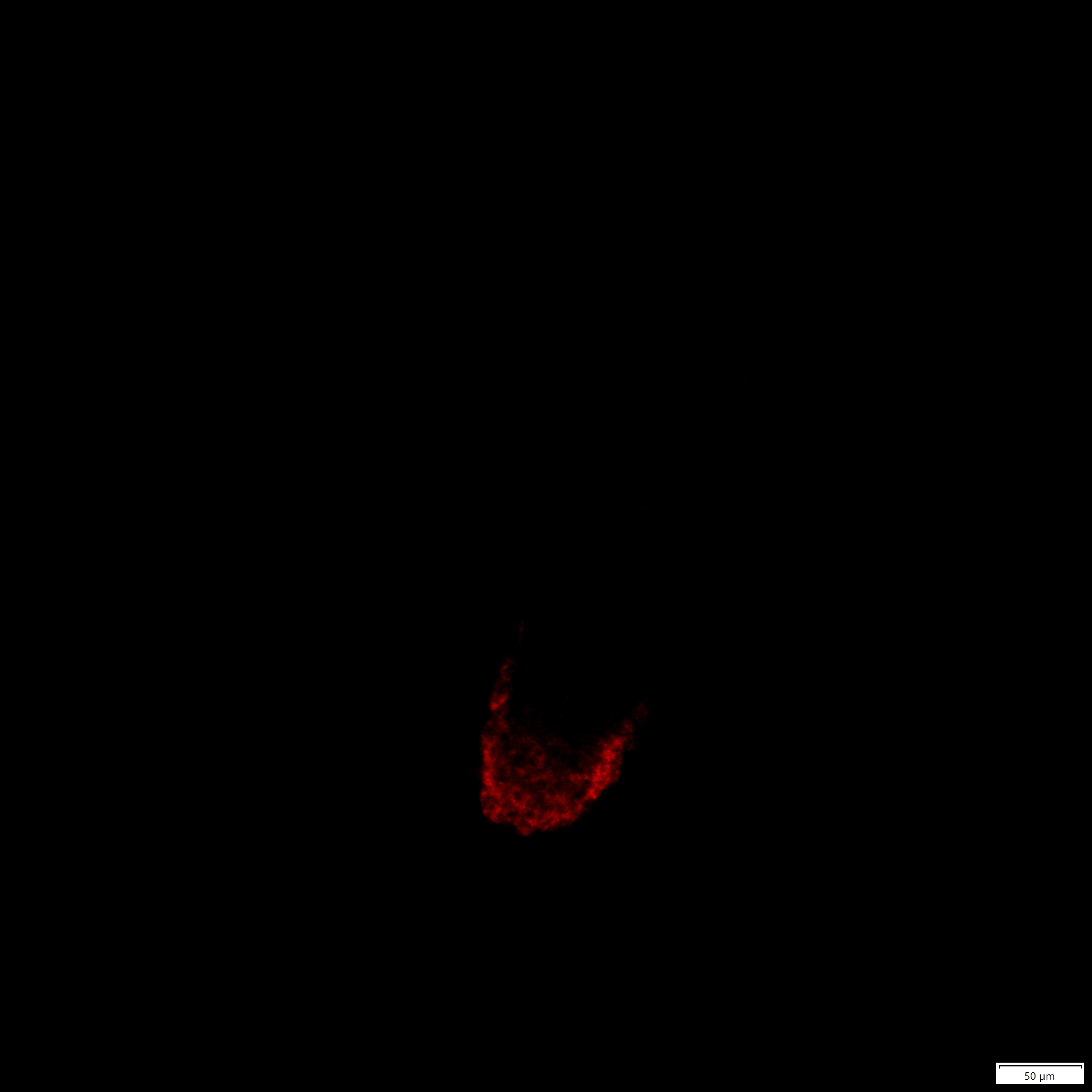

Supplement: Supplementary file 18 — Source data Fig. 2 [file 44318_2025_643_MOESM18_ESM.zip › Figure 2/2H/bmp4 explant_HCR_tbxta.jpg]

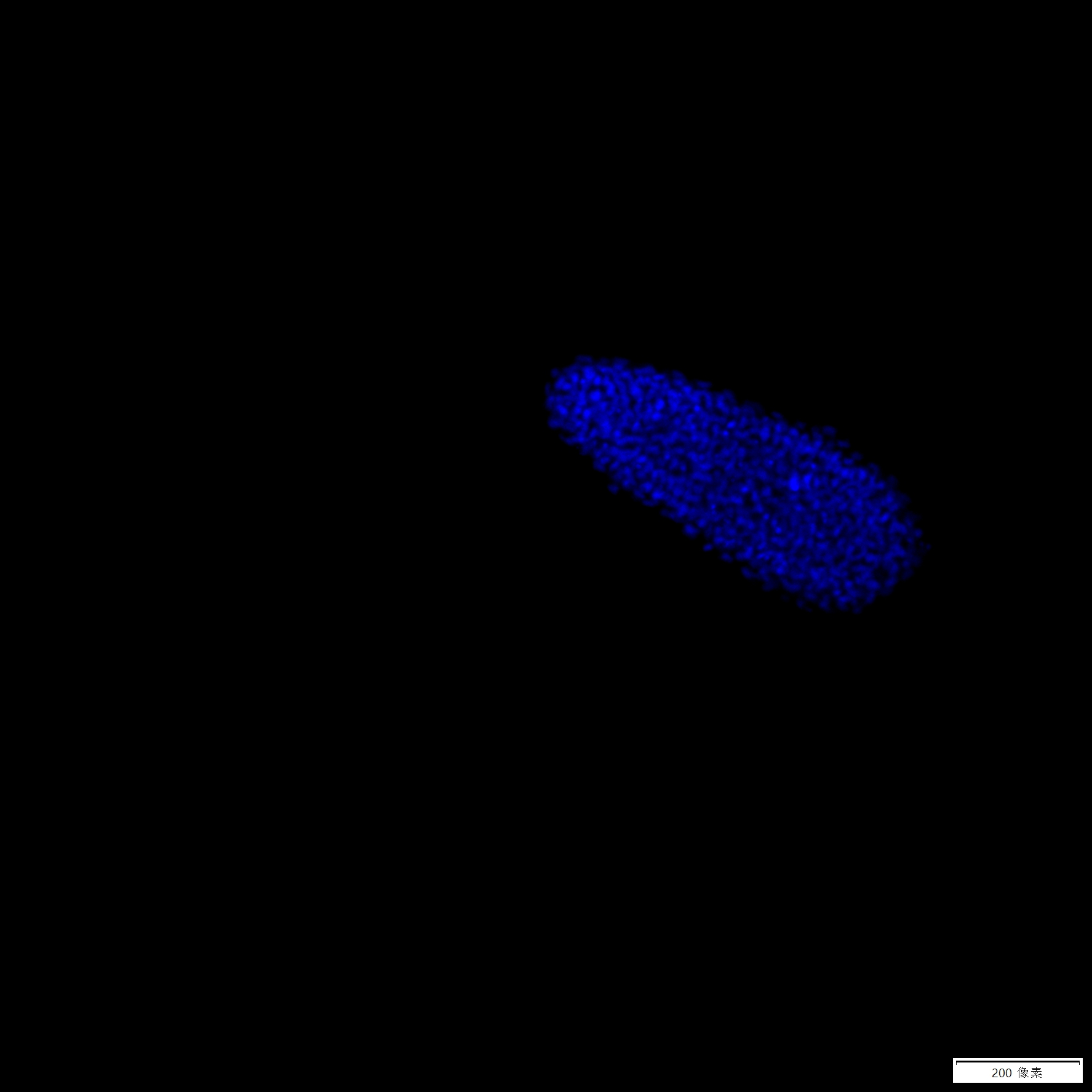

Supplement: Supplementary file 18 — Source data Fig. 2 [file 44318_2025_643_MOESM18_ESM.zip › Figure 2/2I/bmp4_explant_DAPI.jpg]

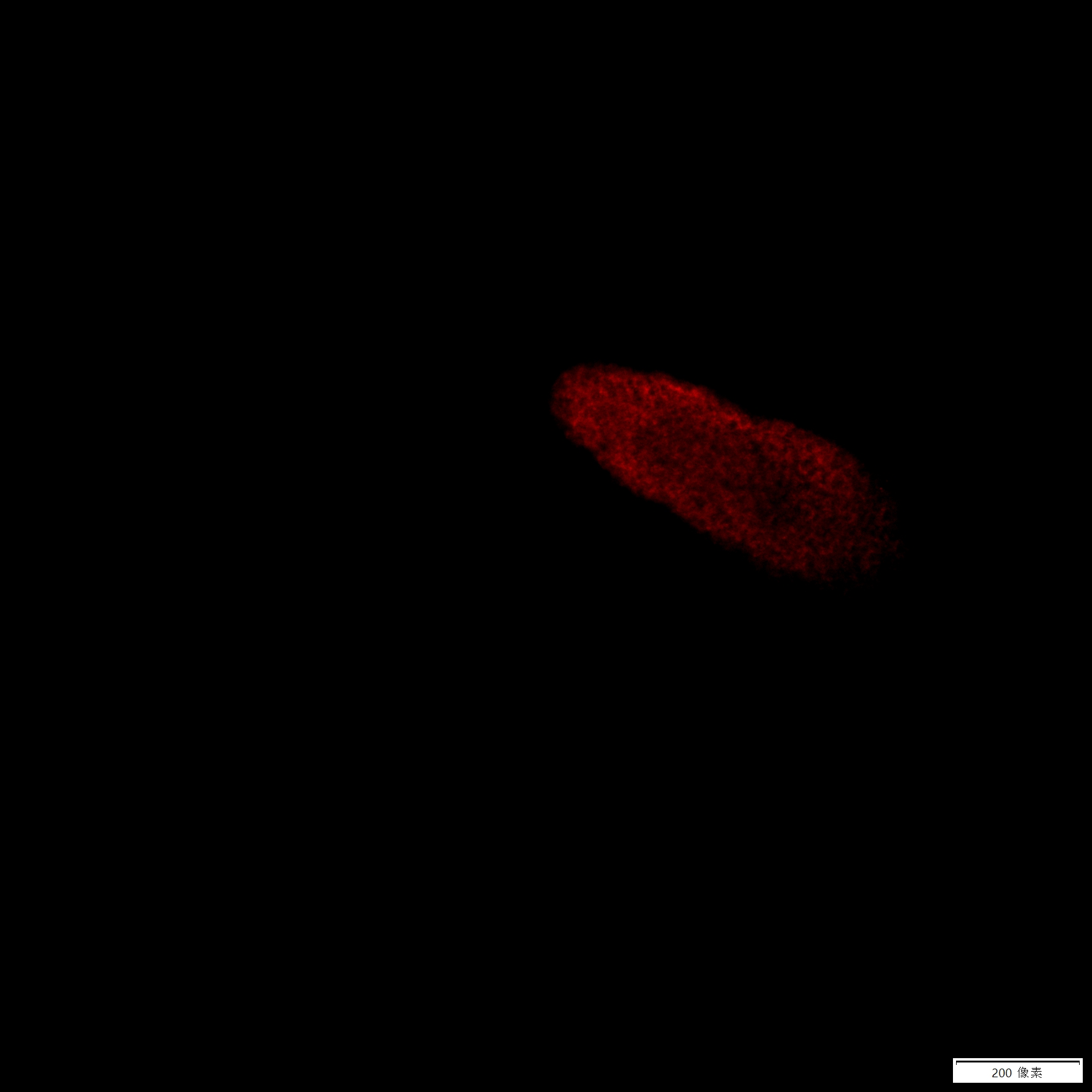

Supplement: Supplementary file 18 — Source data Fig. 2 [file 44318_2025_643_MOESM18_ESM.zip › Figure 2/2I/bmp4_explant_HCR_cdx4.jpg]

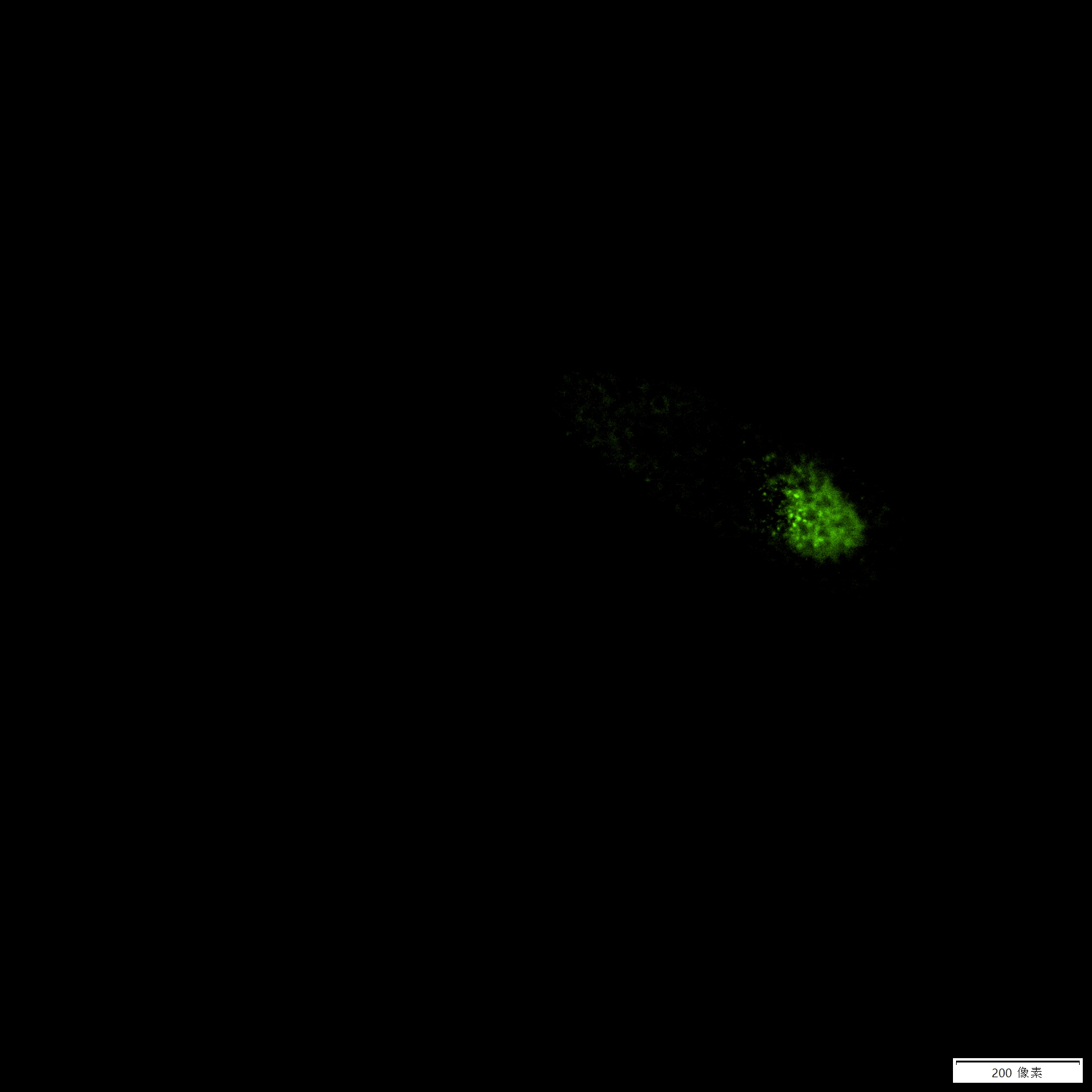

Supplement: Supplementary file 18 — Source data Fig. 2 [file 44318_2025_643_MOESM18_ESM.zip › Figure 2/2I/bmp4_explant_HCR_gata5.jpg]

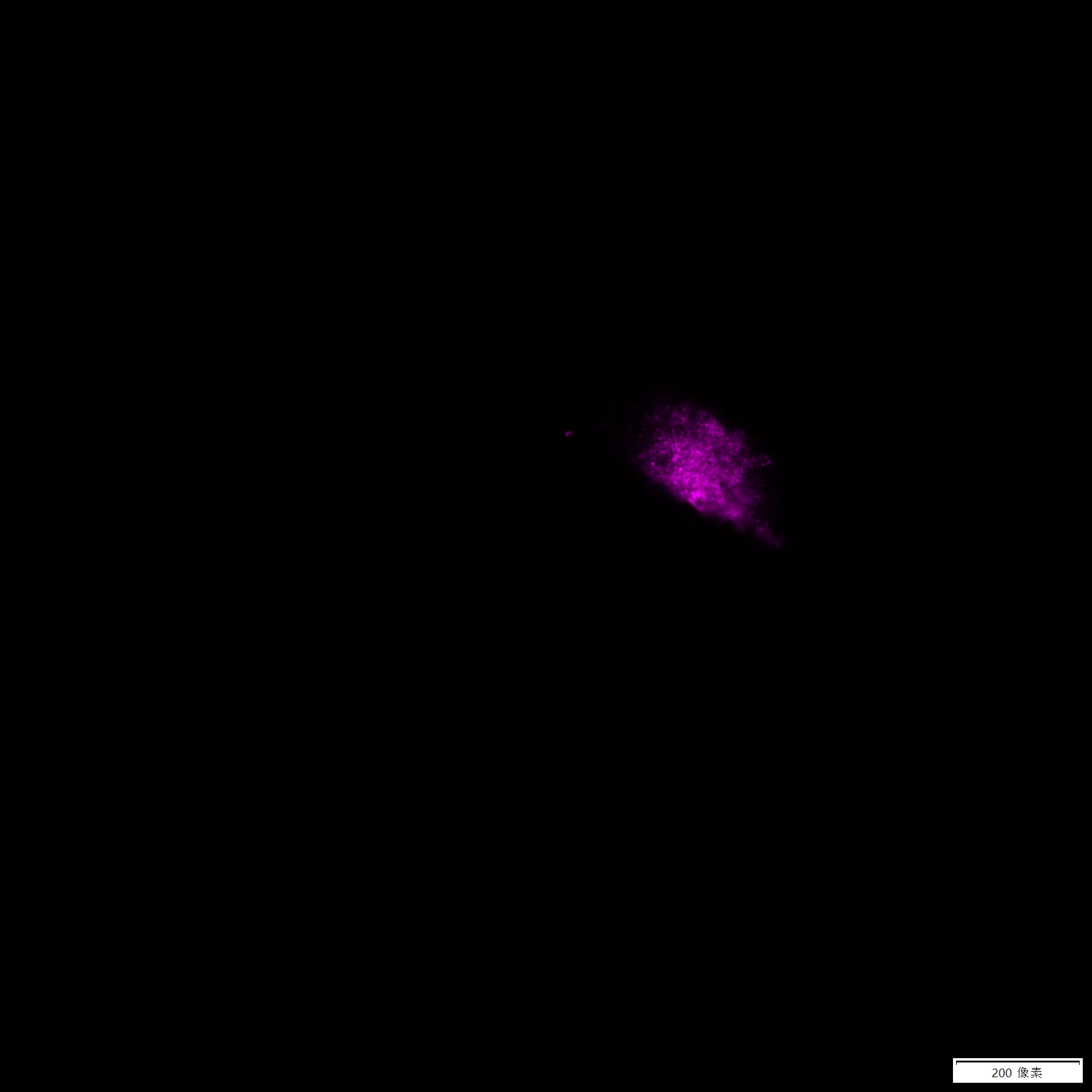

Supplement: Supplementary file 18 — Source data Fig. 2 [file 44318_2025_643_MOESM18_ESM.zip › Figure 2/2I/bmp4_explant_HCR_tbx6.jpg]

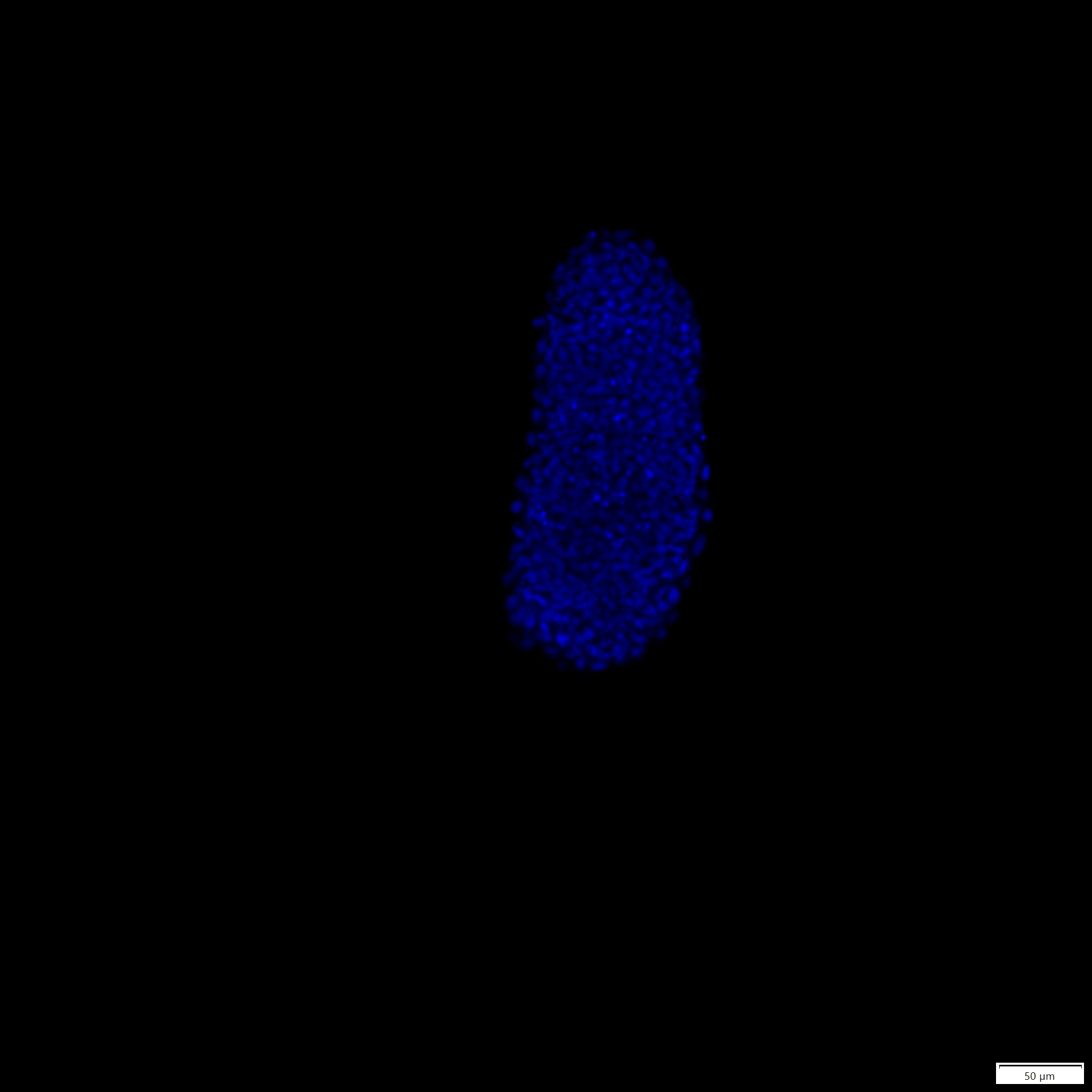

Supplement: Supplementary file 18 — Source data Fig. 2 [file 44318_2025_643_MOESM18_ESM.zip › Figure 2/2J/bmp4 explant_DAPI-2.jpg]

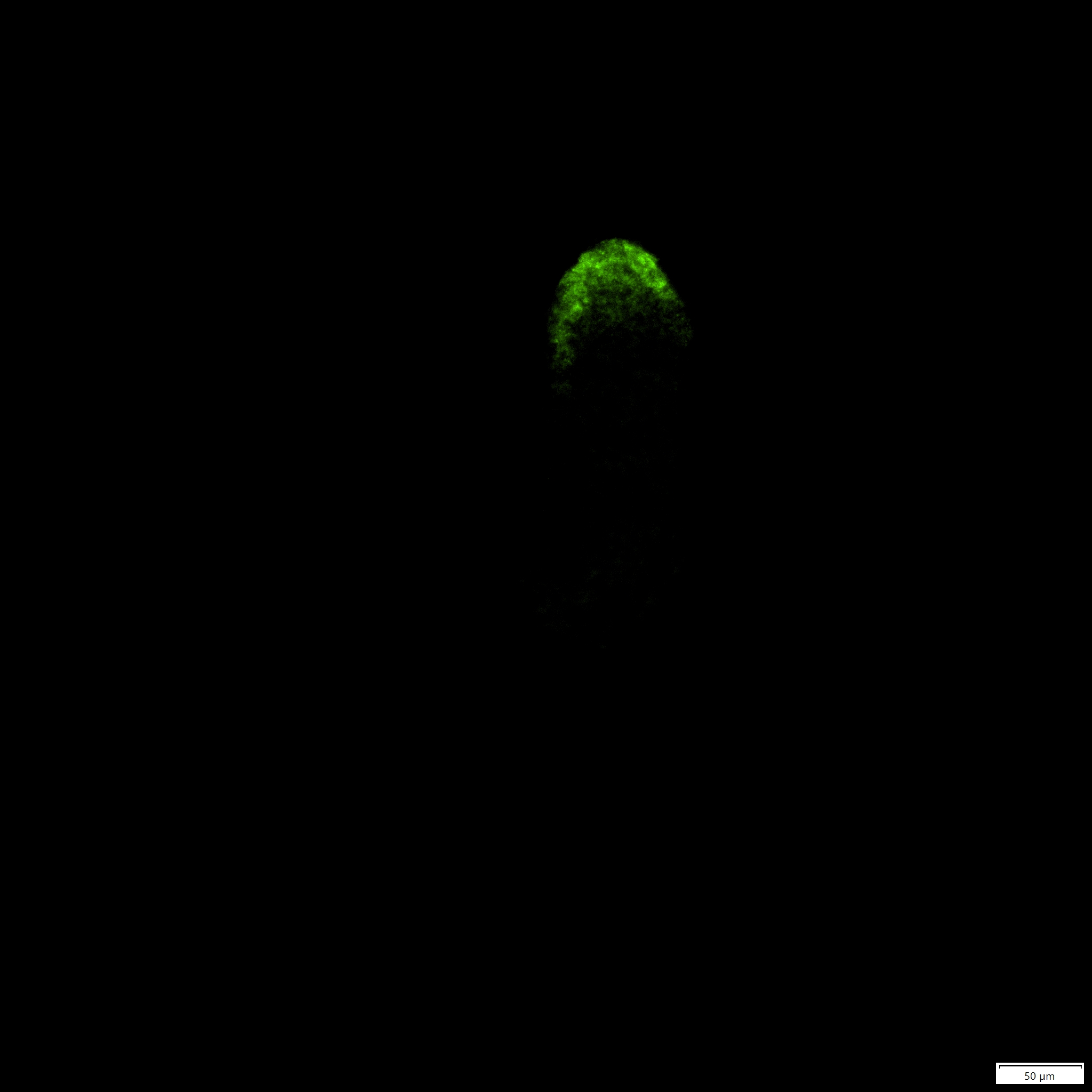

Supplement: Supplementary file 18 — Source data Fig. 2 [file 44318_2025_643_MOESM18_ESM.zip › Figure 2/2J/bmp4 explant_HCR_tbxta-2.jpg]

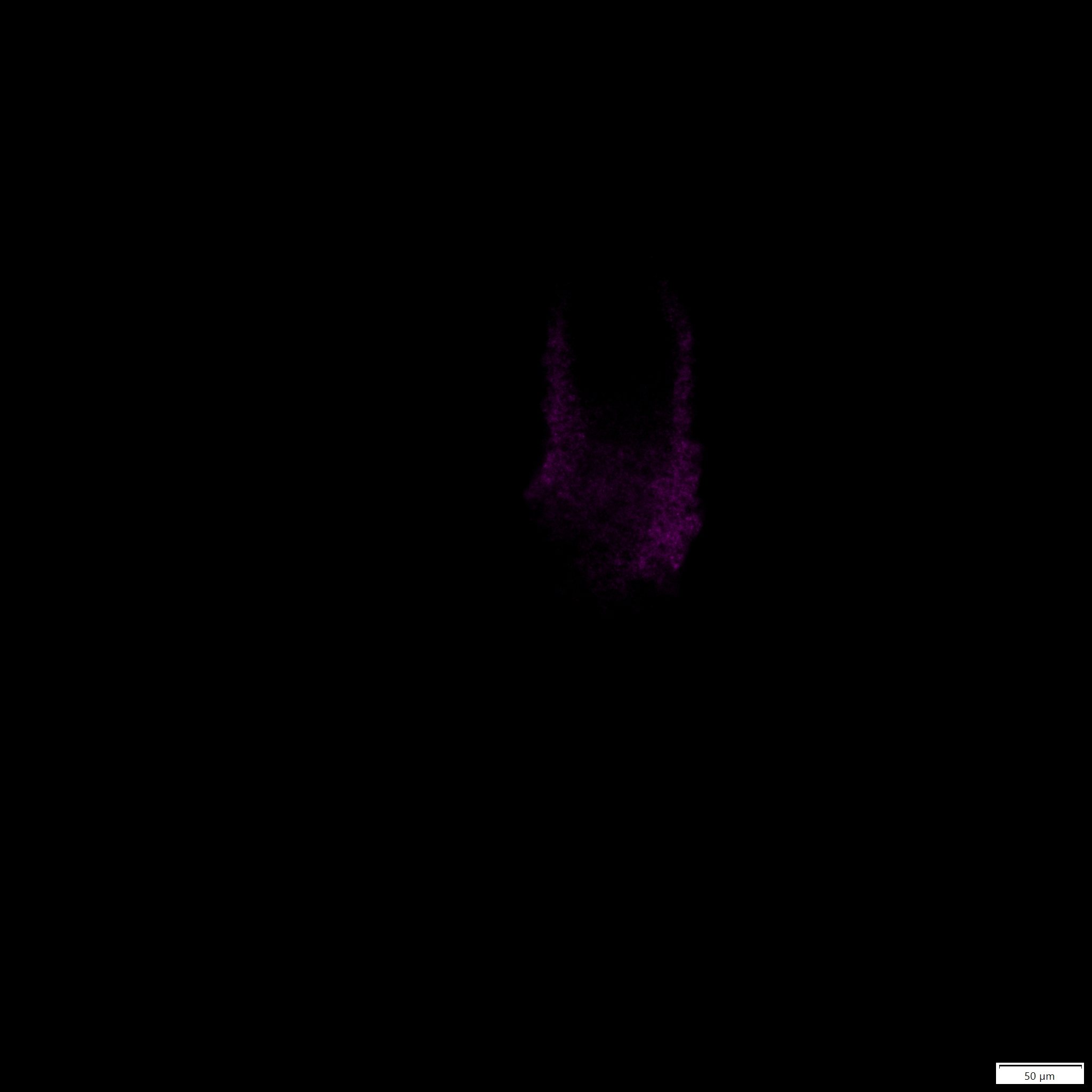

Supplement: Supplementary file 18 — Source data Fig. 2 [file 44318_2025_643_MOESM18_ESM.zip › Figure 2/2J/bmp4 explant_HCR_zic2b.jpg]

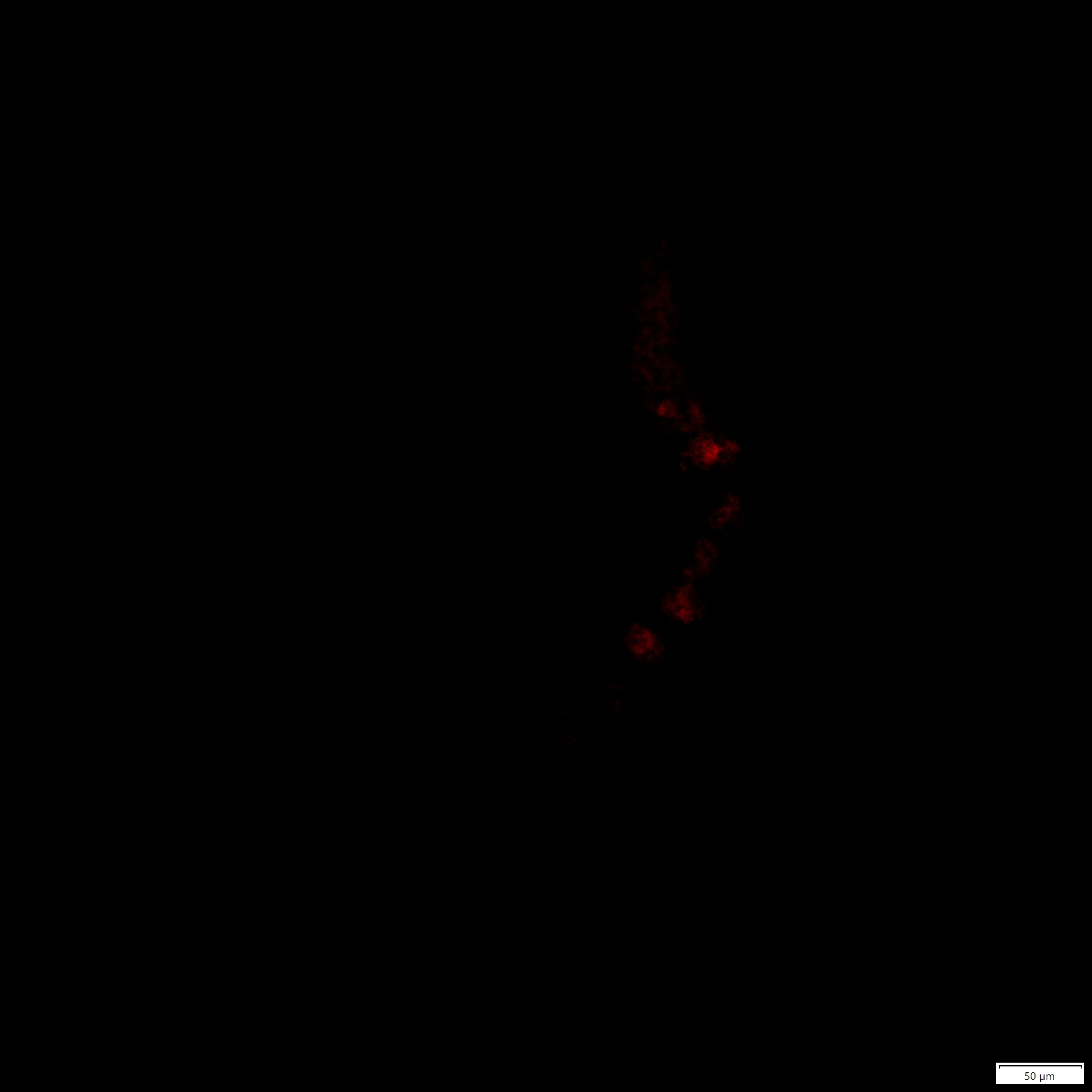

Supplement: Supplementary file 18 — Source data Fig. 2 [file 44318_2025_643_MOESM18_ESM.zip › Figure 2/2L/bmp4 explant_18hpf_HCR_myod1.jpg]

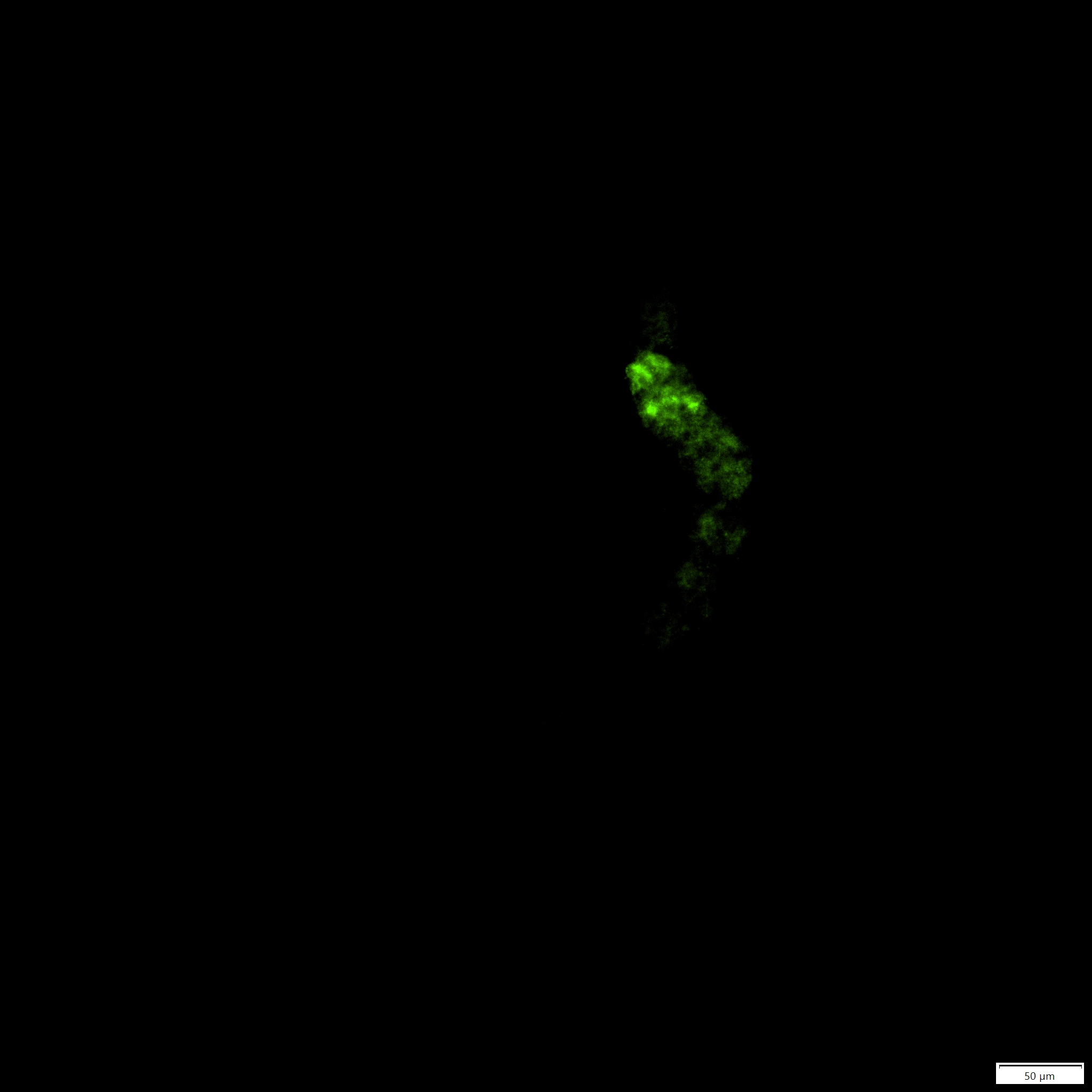

Supplement: Supplementary file 18 — Source data Fig. 2 [file 44318_2025_643_MOESM18_ESM.zip › Figure 2/2L/bmp4 explant_18hpf_HCR_ripply1.jpg]

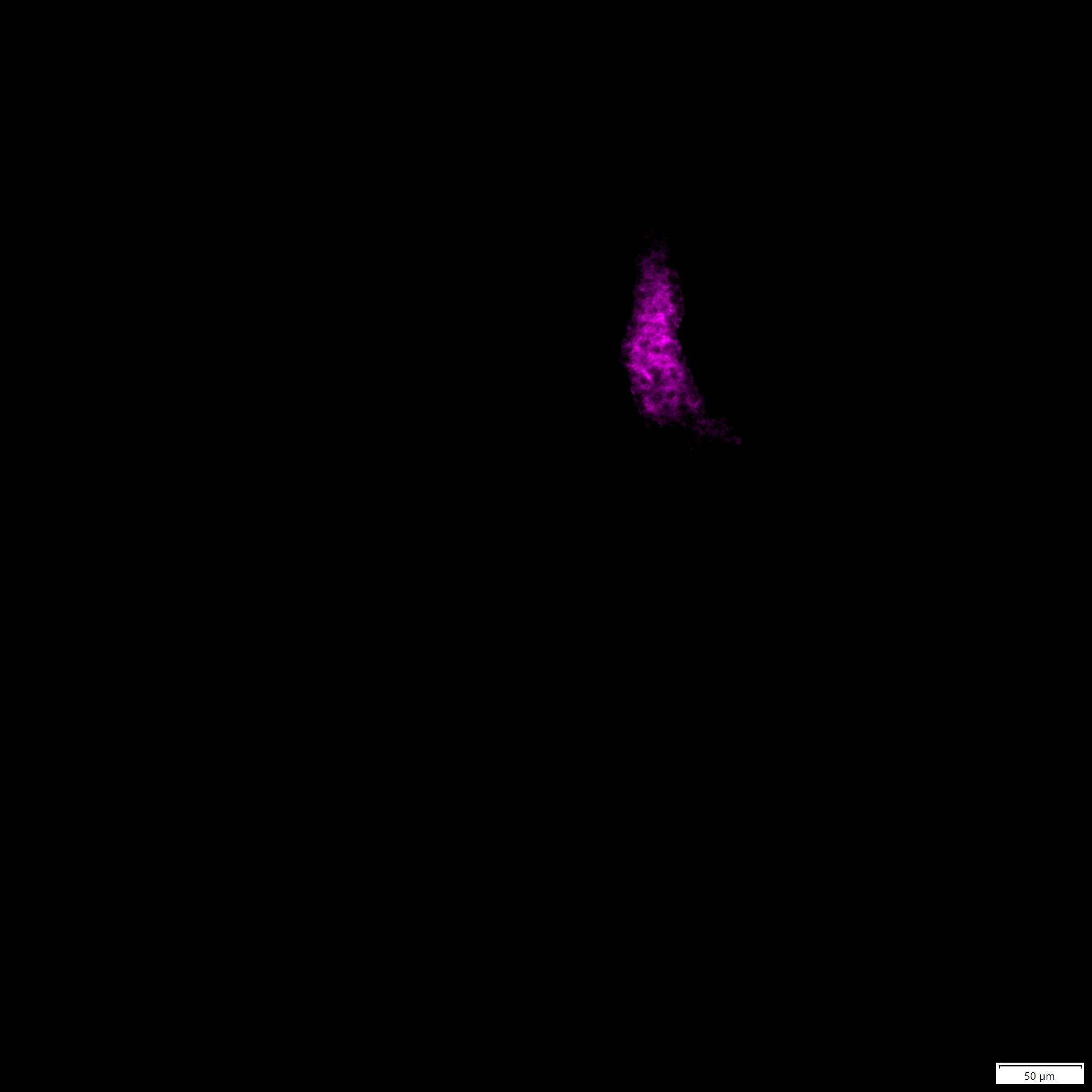

Supplement: Supplementary file 18 — Source data Fig. 2 [file 44318_2025_643_MOESM18_ESM.zip › Figure 2/2L/bmp4 explant_18hpf_HCR_tbx6.jpg]

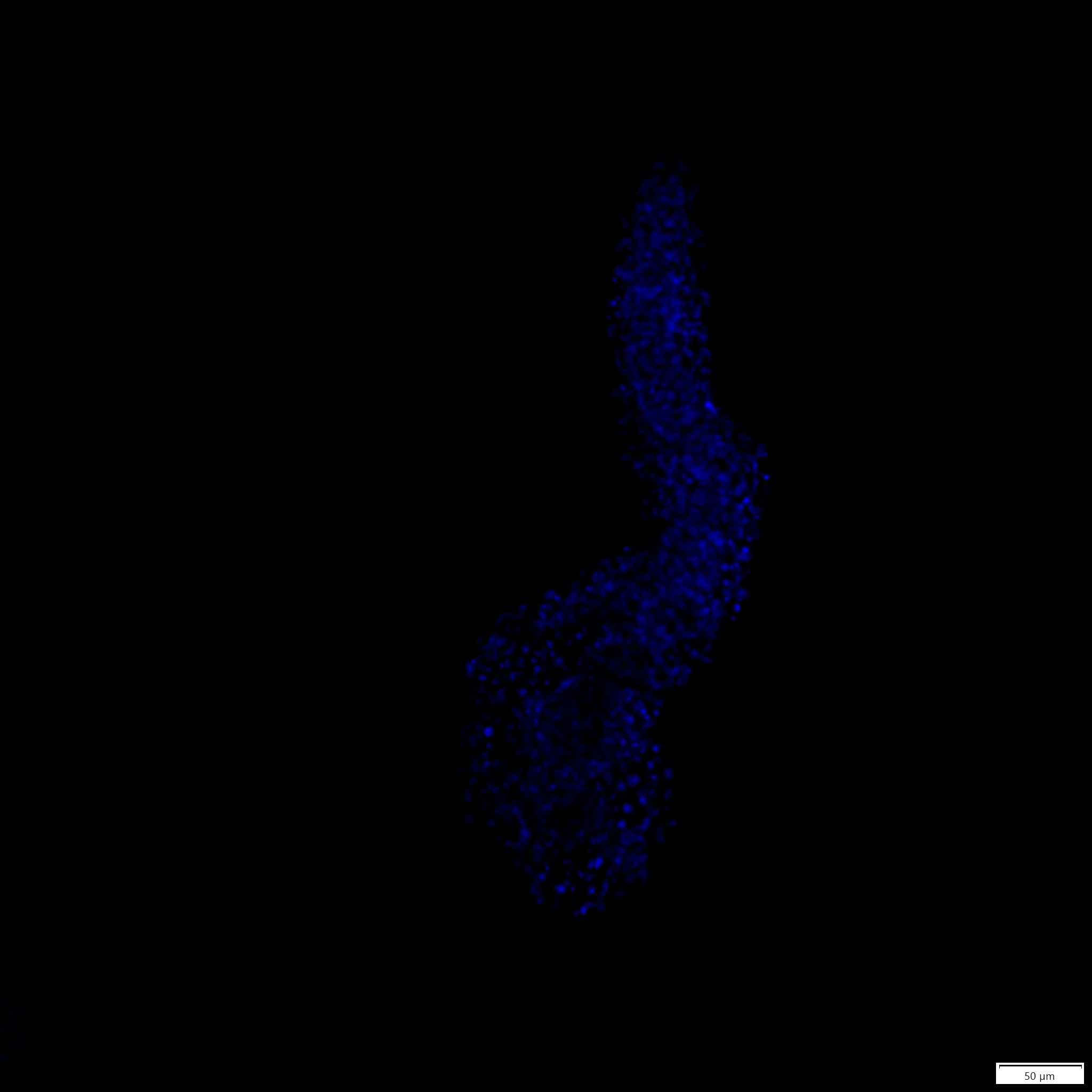

Supplement: Supplementary file 18 — Source data Fig. 2 [file 44318_2025_643_MOESM18_ESM.zip › Figure 2/2L/bmp4 explant__18hpf_DAPI.jpg]

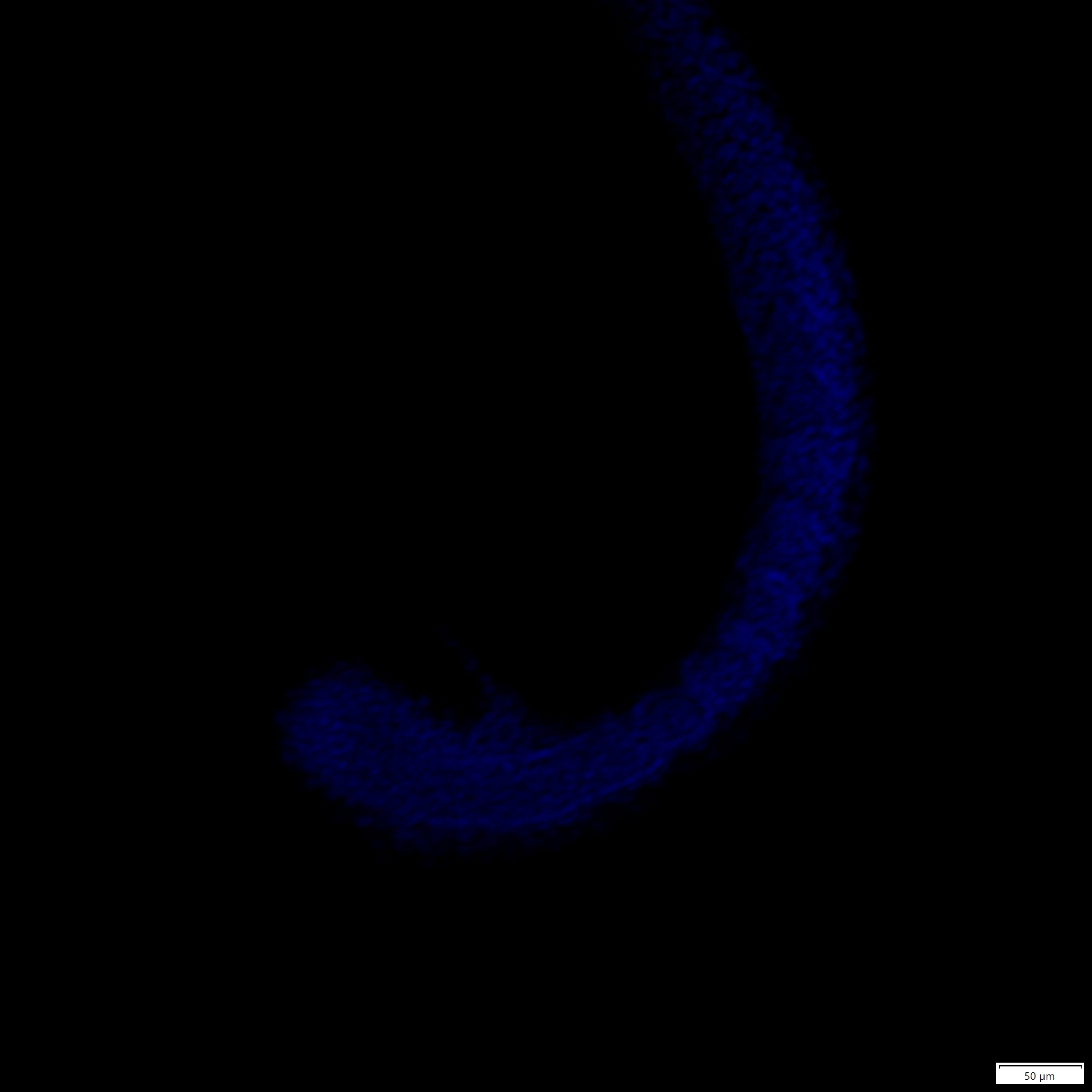

Supplement: Supplementary file 18 — Source data Fig. 2 [file 44318_2025_643_MOESM18_ESM.zip › Figure 2/2M/embryo_18hpf_DAPI.jpg]

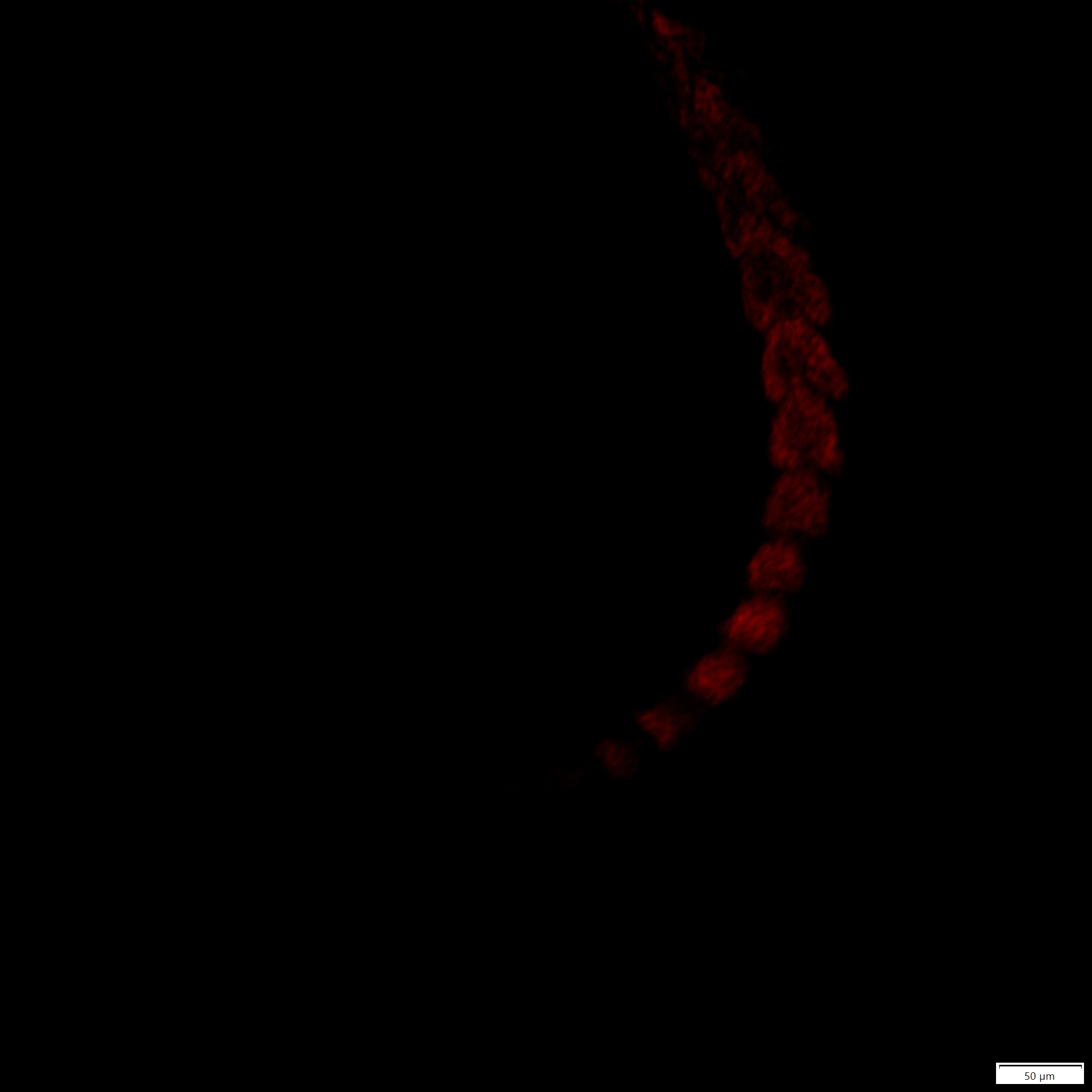

Supplement: Supplementary file 18 — Source data Fig. 2 [file 44318_2025_643_MOESM18_ESM.zip › Figure 2/2M/embryo_18hpf_HCR_myod1.jpg]

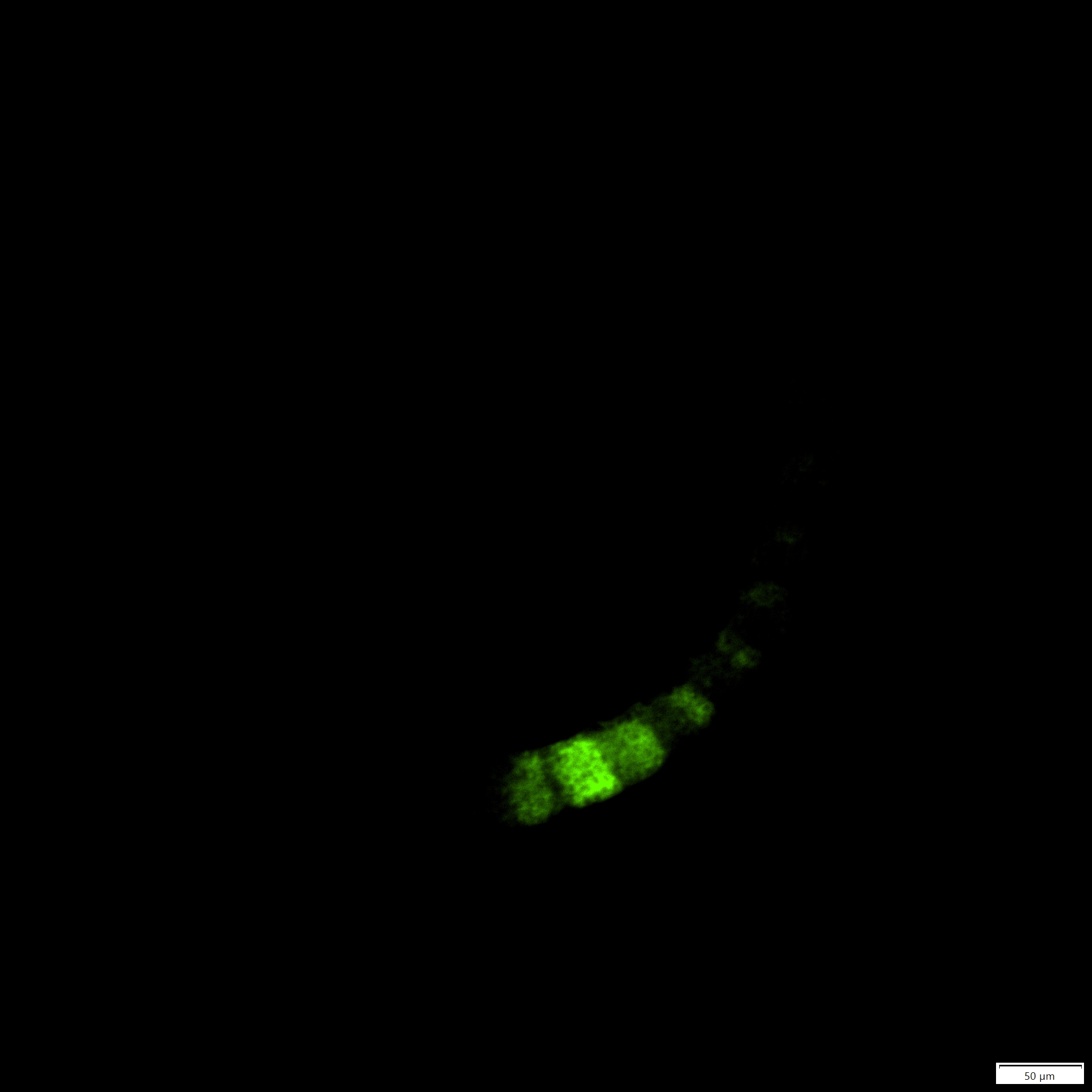

Supplement: Supplementary file 18 — Source data Fig. 2 [file 44318_2025_643_MOESM18_ESM.zip › Figure 2/2M/embryo_18hpf_HCR_ripply1.jpg]

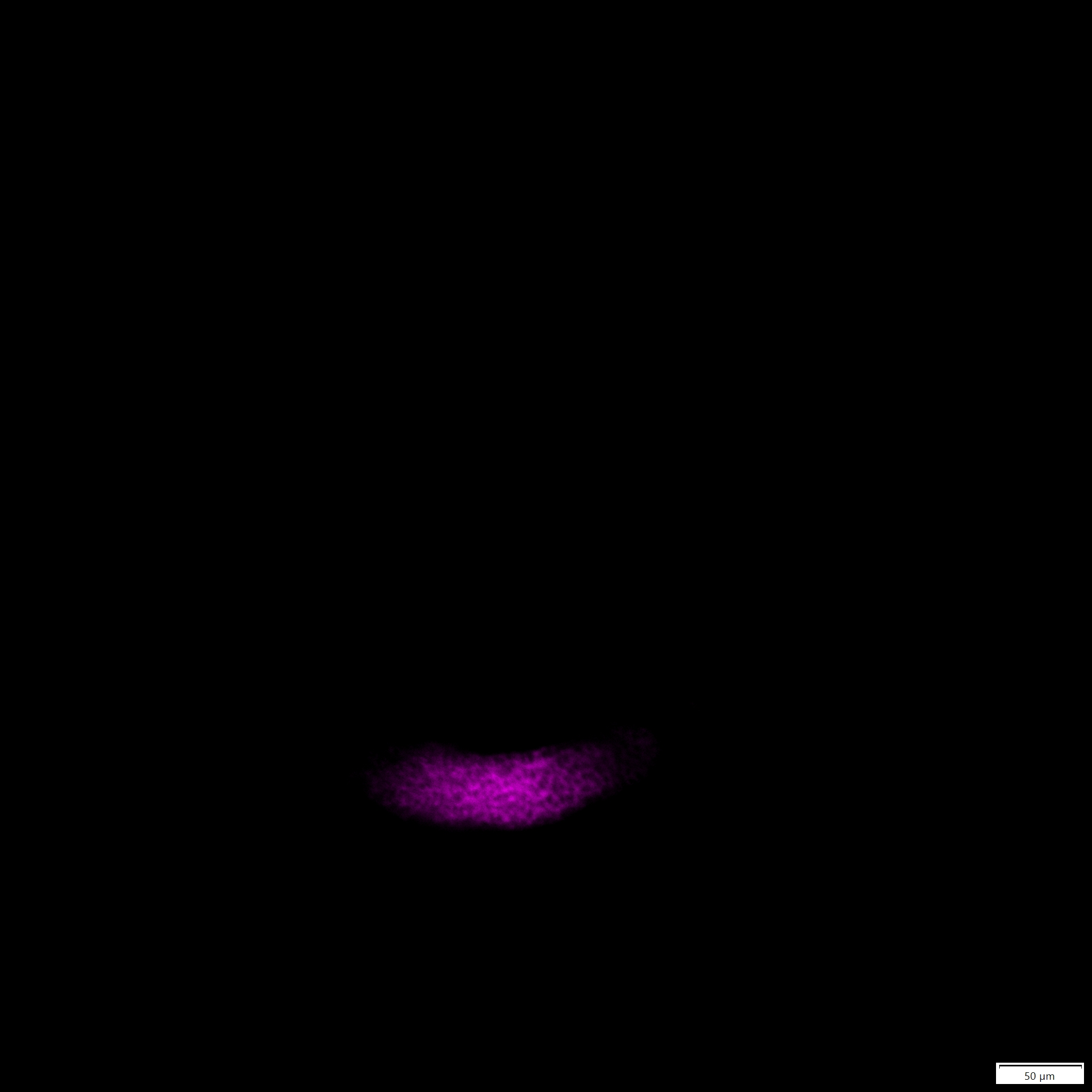

Supplement: Supplementary file 18 — Source data Fig. 2 [file 44318_2025_643_MOESM18_ESM.zip › Figure 2/2M/embryo_18hpf_HCR_tbx6.jpg]

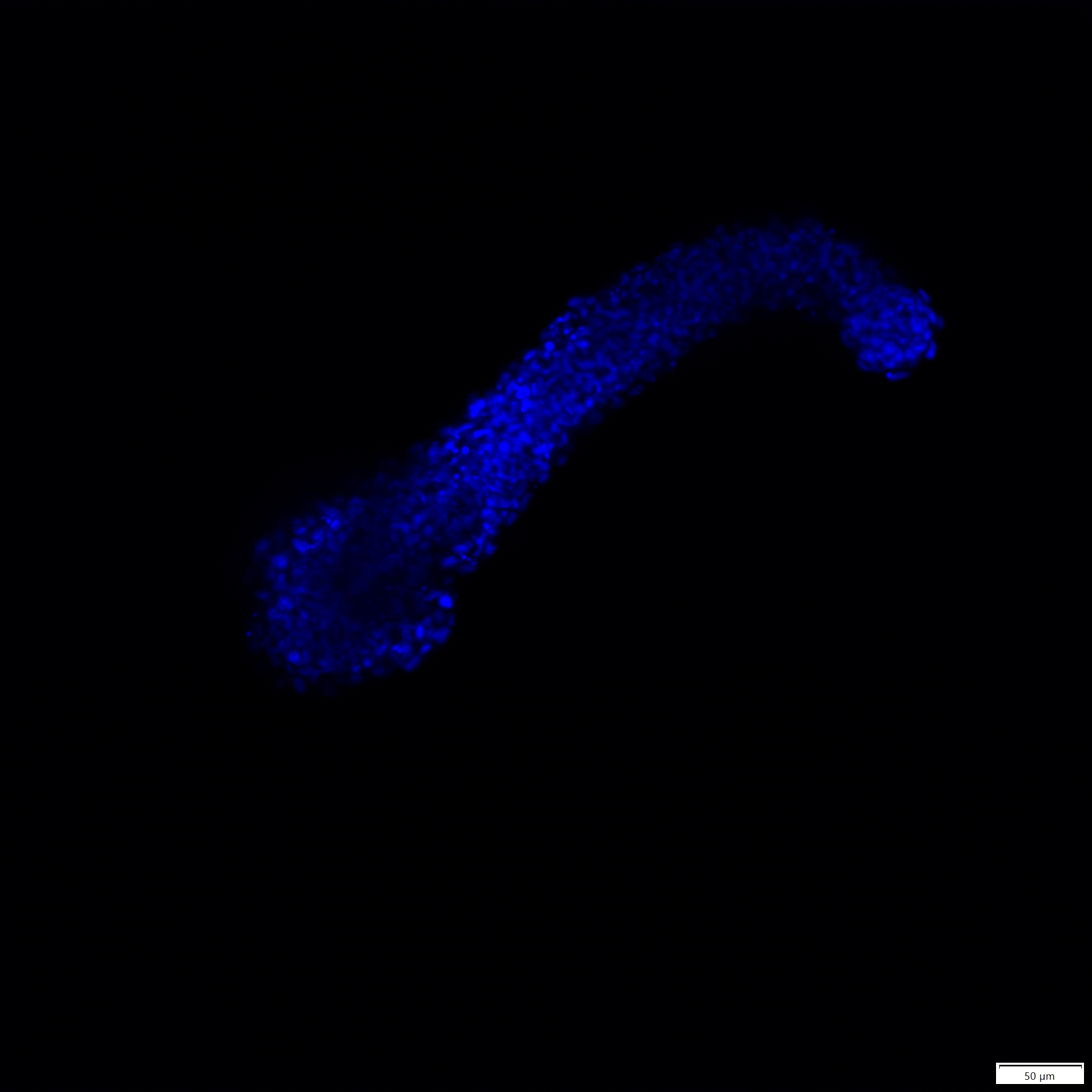

Supplement: Supplementary file 18 — Source data Fig. 2 [file 44318_2025_643_MOESM18_ESM.zip › Figure 2/2N/bmp4 explant_18hpf_DAPI.jpg]

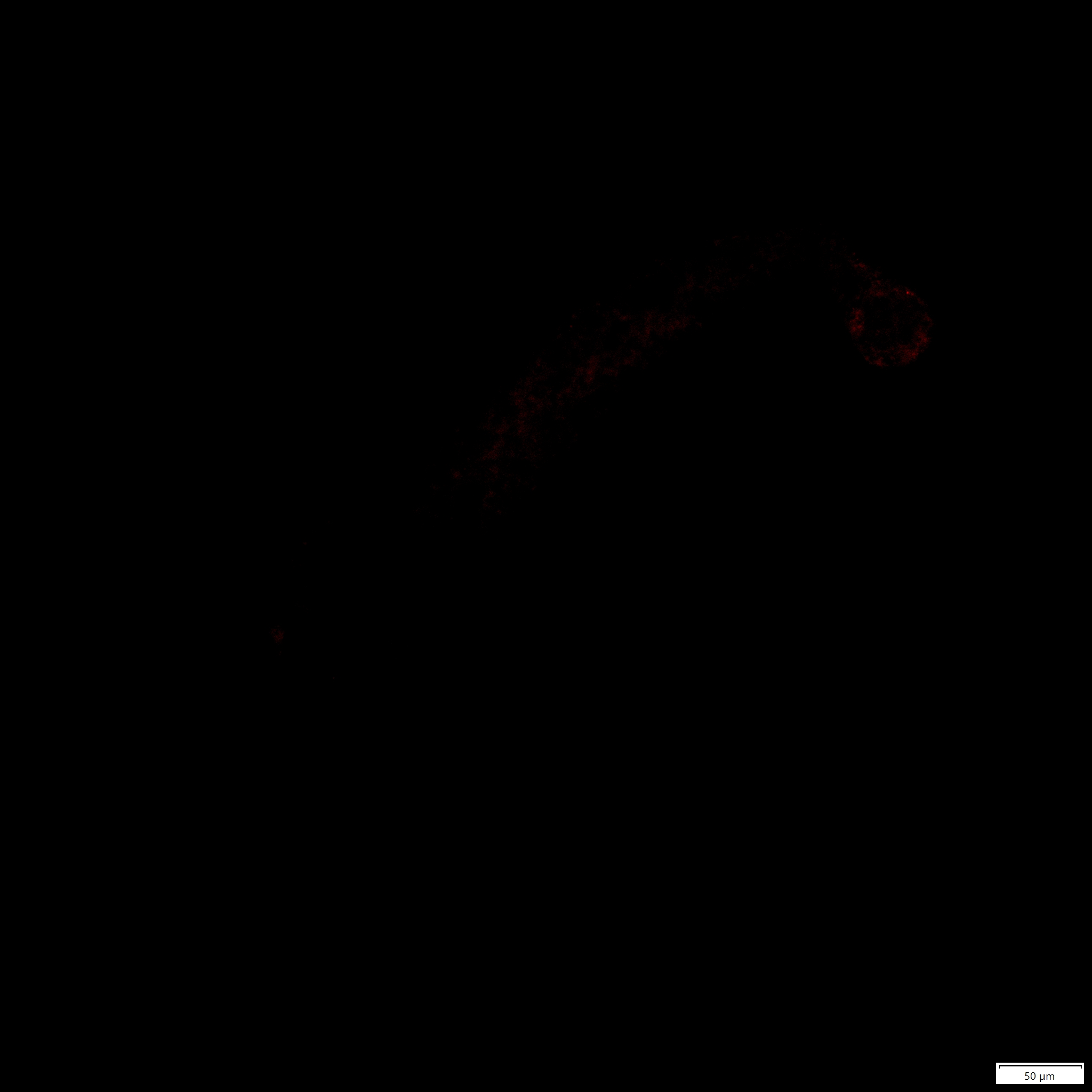

Supplement: Supplementary file 18 — Source data Fig. 2 [file 44318_2025_643_MOESM18_ESM.zip › Figure 2/2N/bmp4 explant_18hpf_HCR_sox2.jpg]

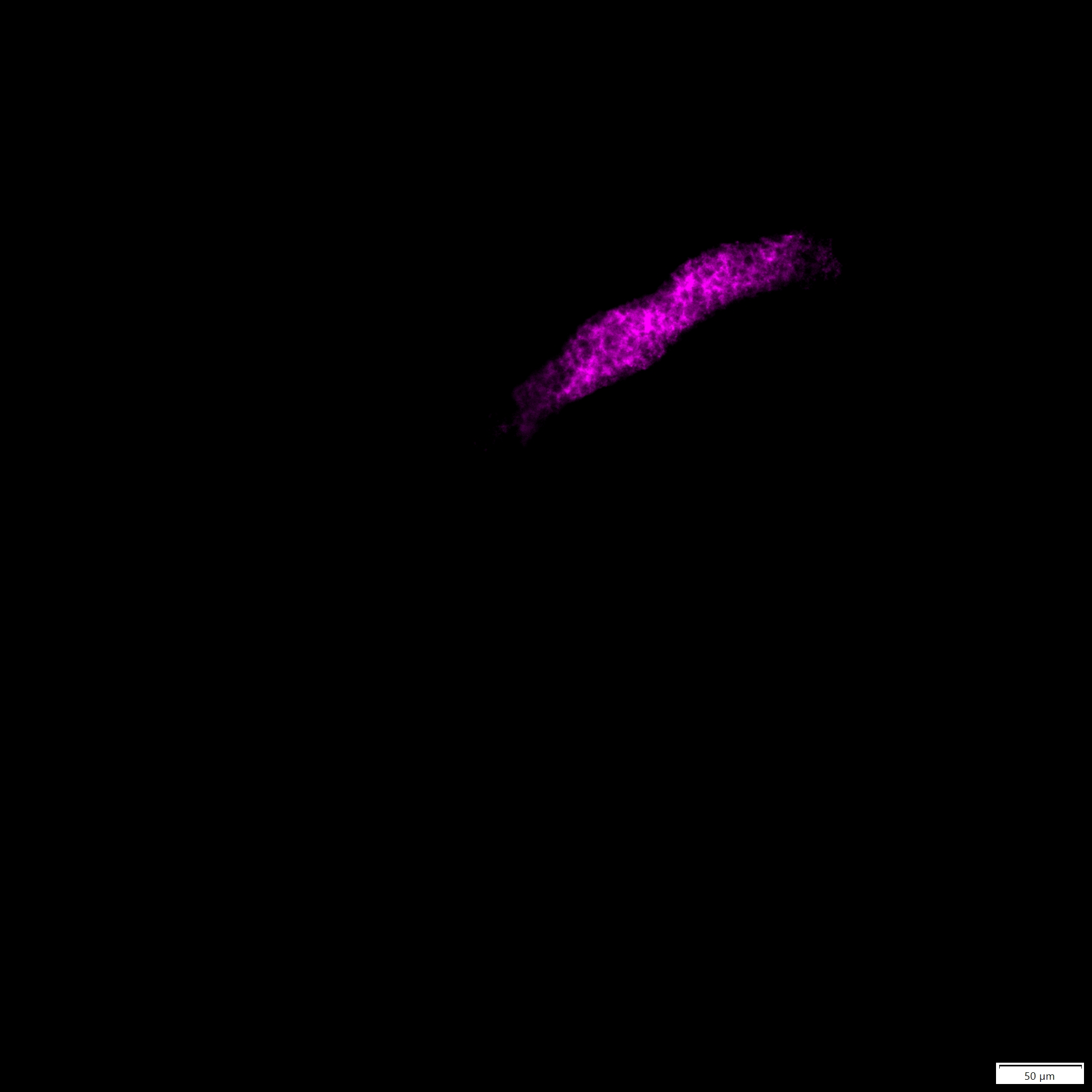

Supplement: Supplementary file 18 — Source data Fig. 2 [file 44318_2025_643_MOESM18_ESM.zip › Figure 2/2N/bmp4 explant_18hpf_HCR_tbx6.jpg]

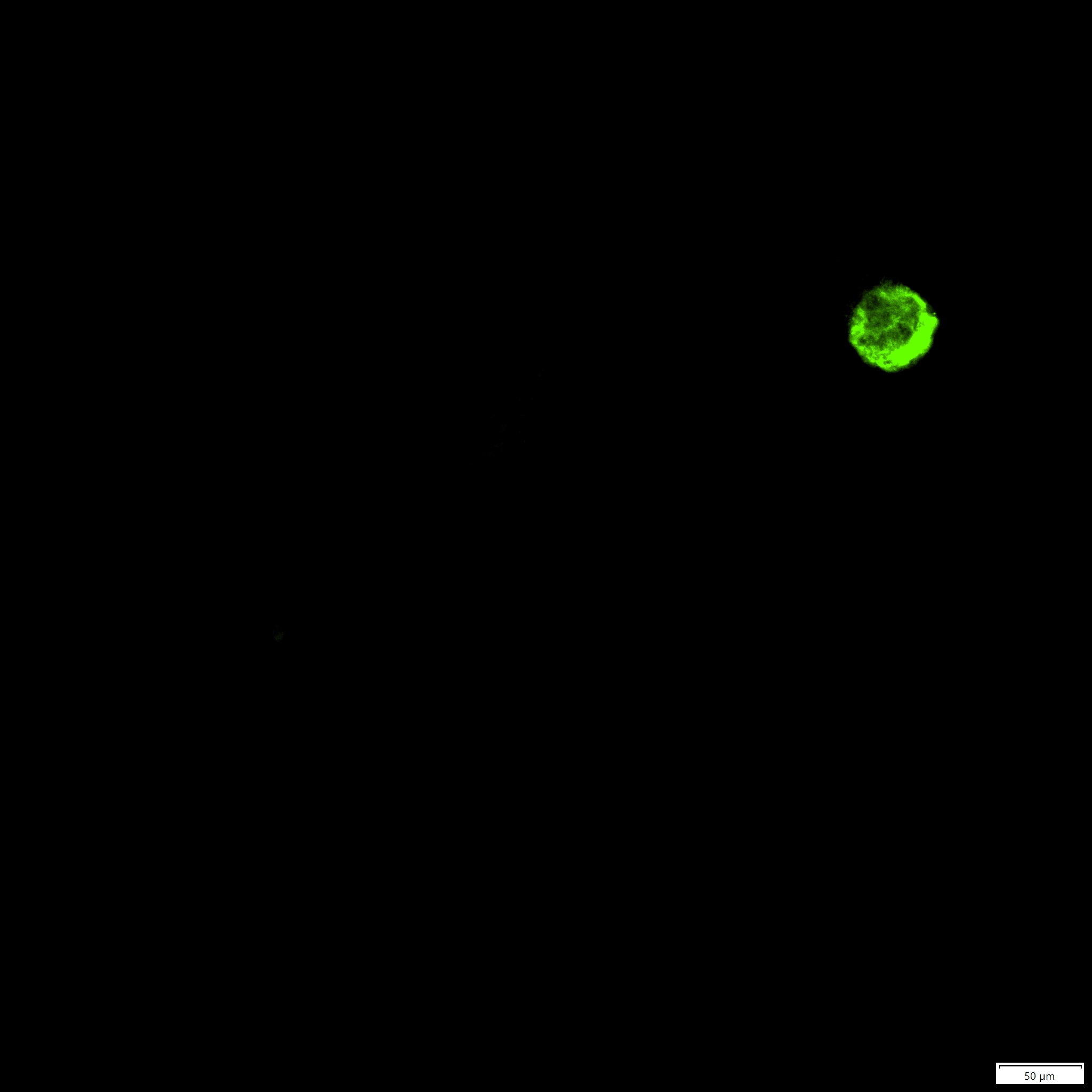

Supplement: Supplementary file 18 — Source data Fig. 2 [file 44318_2025_643_MOESM18_ESM.zip › Figure 2/2N/bmp4 explant_18hpf_HCR_tbxta.jpg]

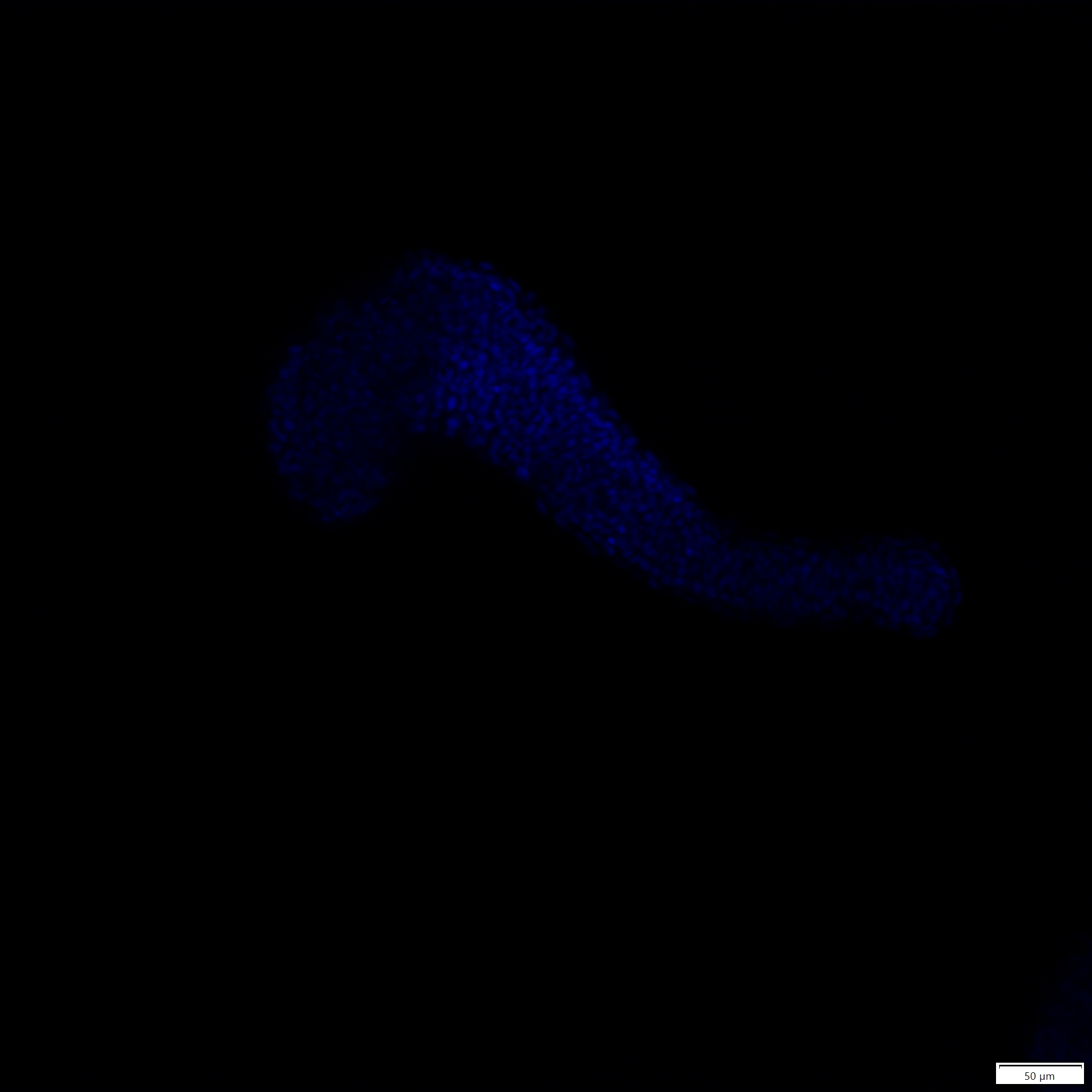

Supplement: Supplementary file 18 — Source data Fig. 2 [file 44318_2025_643_MOESM18_ESM.zip › Figure 2/2O/bmp4 explant_18hpf_DAPI.jpg]

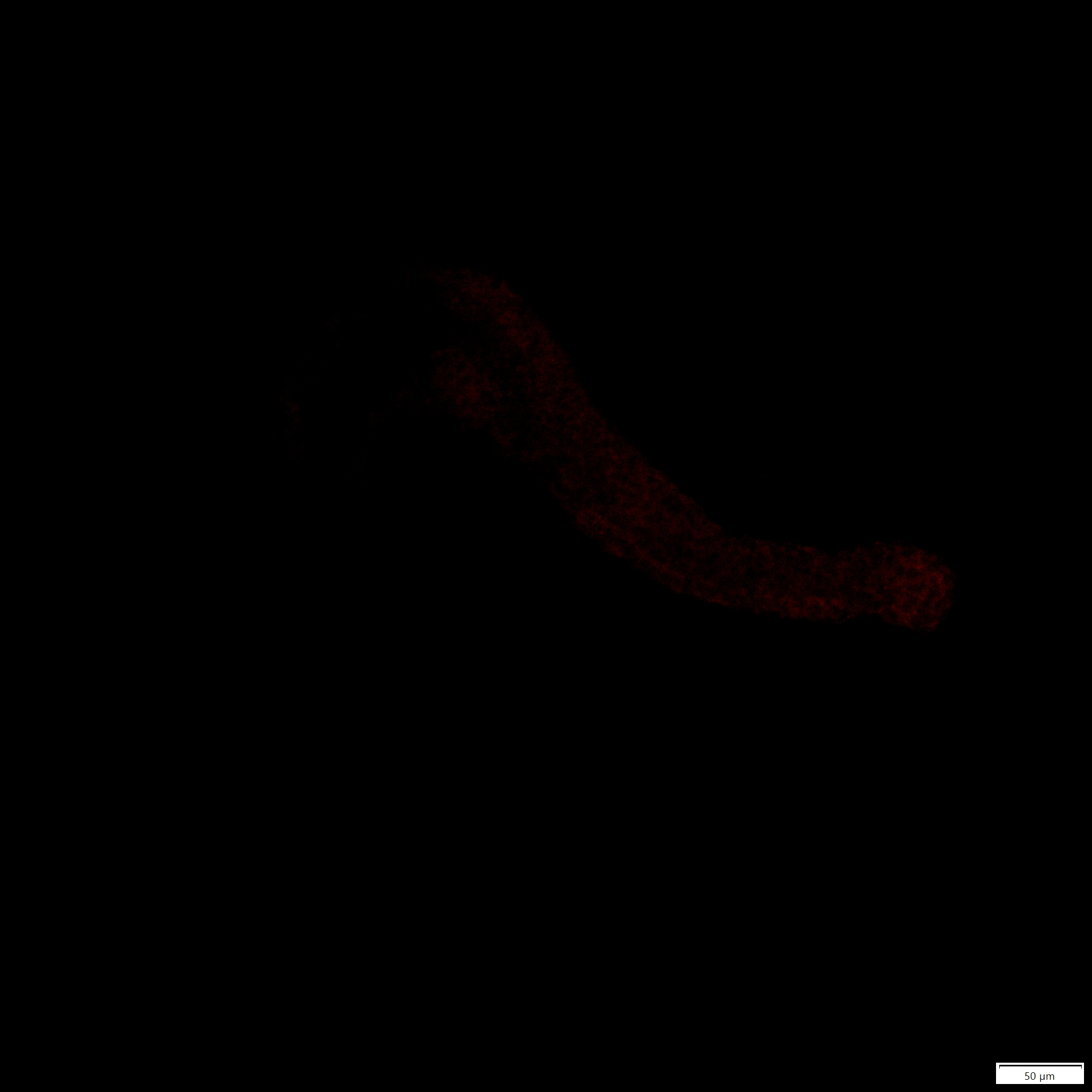

Supplement: Supplementary file 18 — Source data Fig. 2 [file 44318_2025_643_MOESM18_ESM.zip › Figure 2/2O/bmp4 explant_18hpf_HCR_cdx4.jpg]

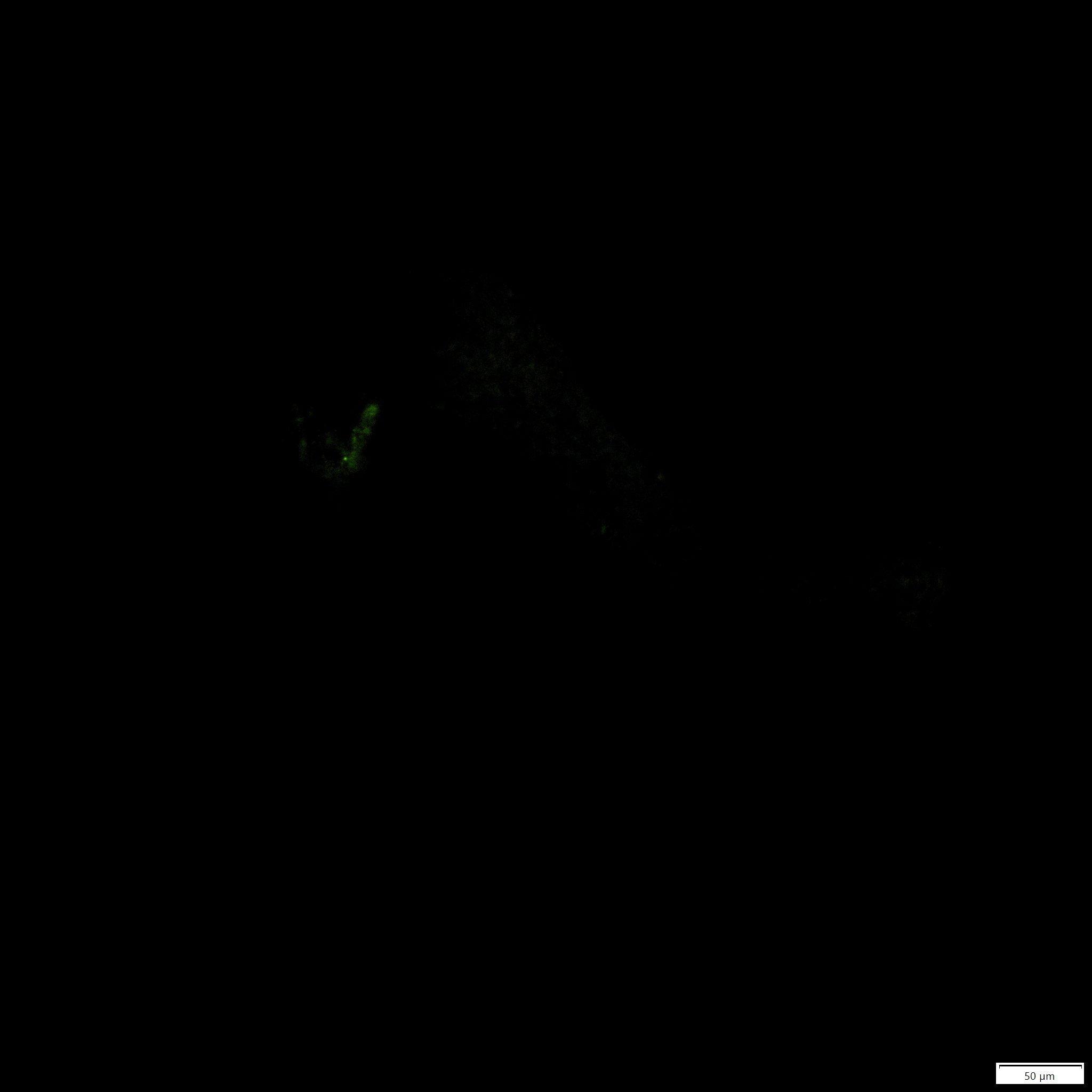

Supplement: Supplementary file 18 — Source data Fig. 2 [file 44318_2025_643_MOESM18_ESM.zip › Figure 2/2O/bmp4 explant_18hpf_HCR_gata5.jpg]

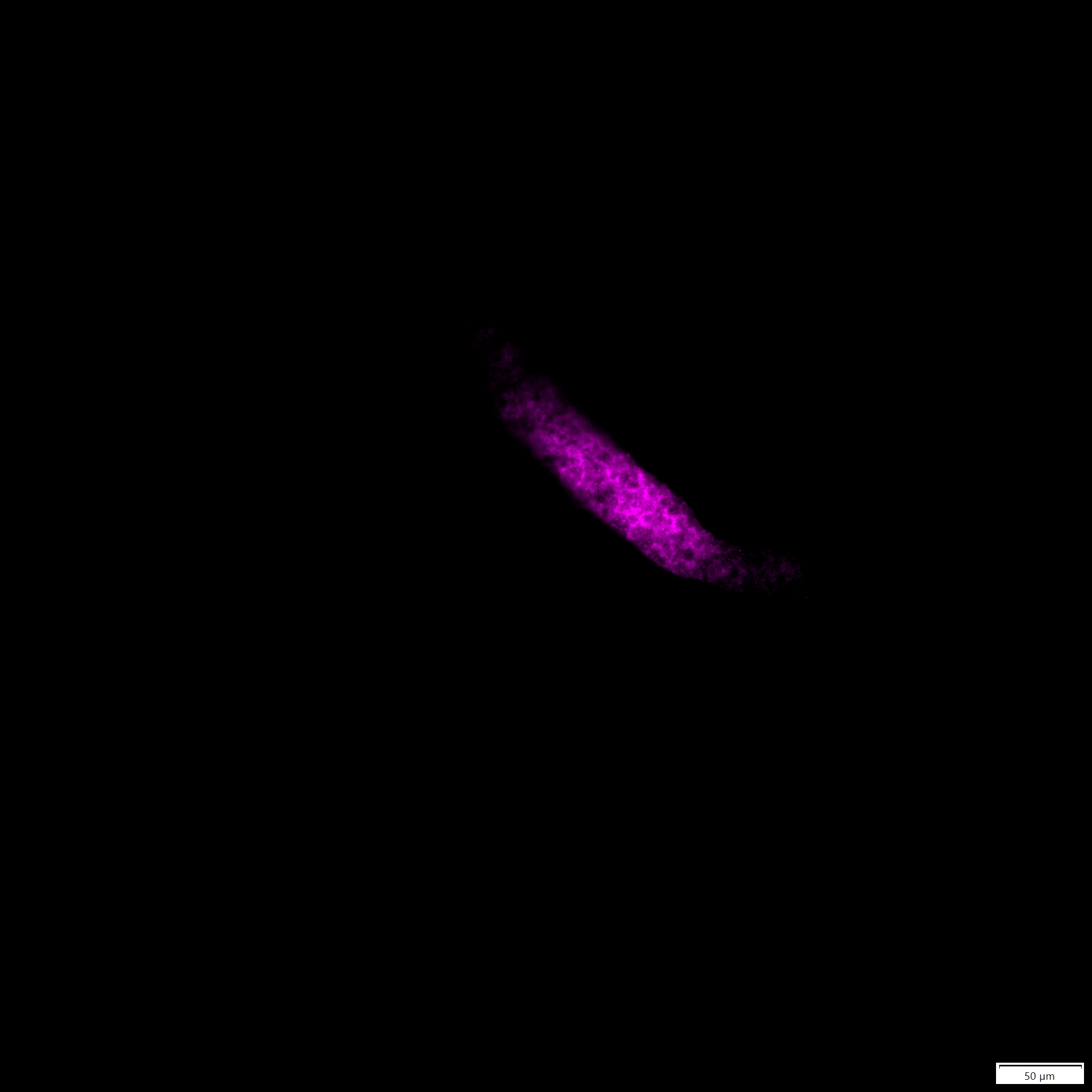

Supplement: Supplementary file 18 — Source data Fig. 2 [file 44318_2025_643_MOESM18_ESM.zip › Figure 2/2O/bmp4 explant_18hpf_HCR_tbx6.jpg]

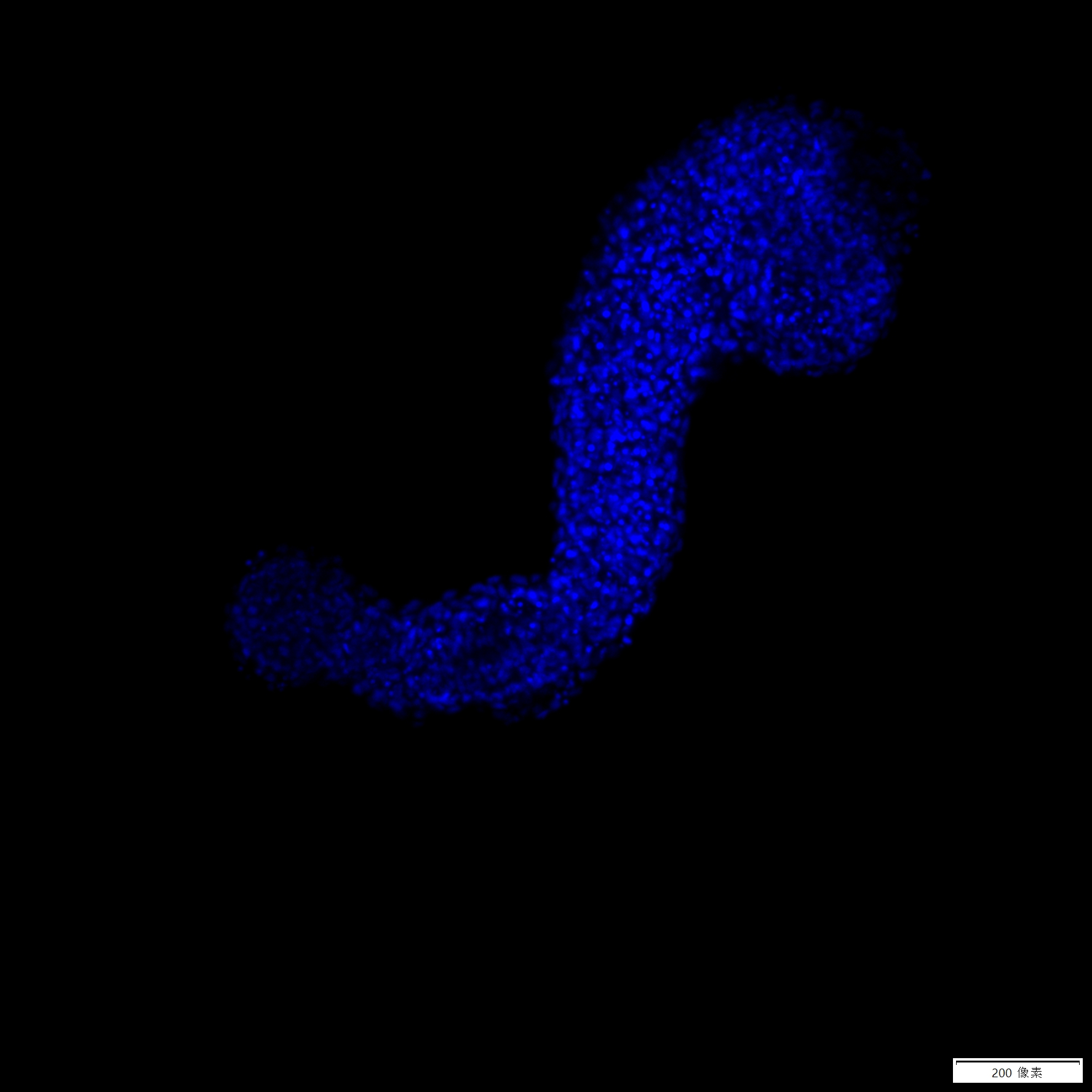

Supplement: Supplementary file 19 — Source data Fig. 3 [file 44318_2025_643_MOESM19_ESM.zip › Figure 3/3G/bmp4 explant_24hpf_DAPI.jpg]

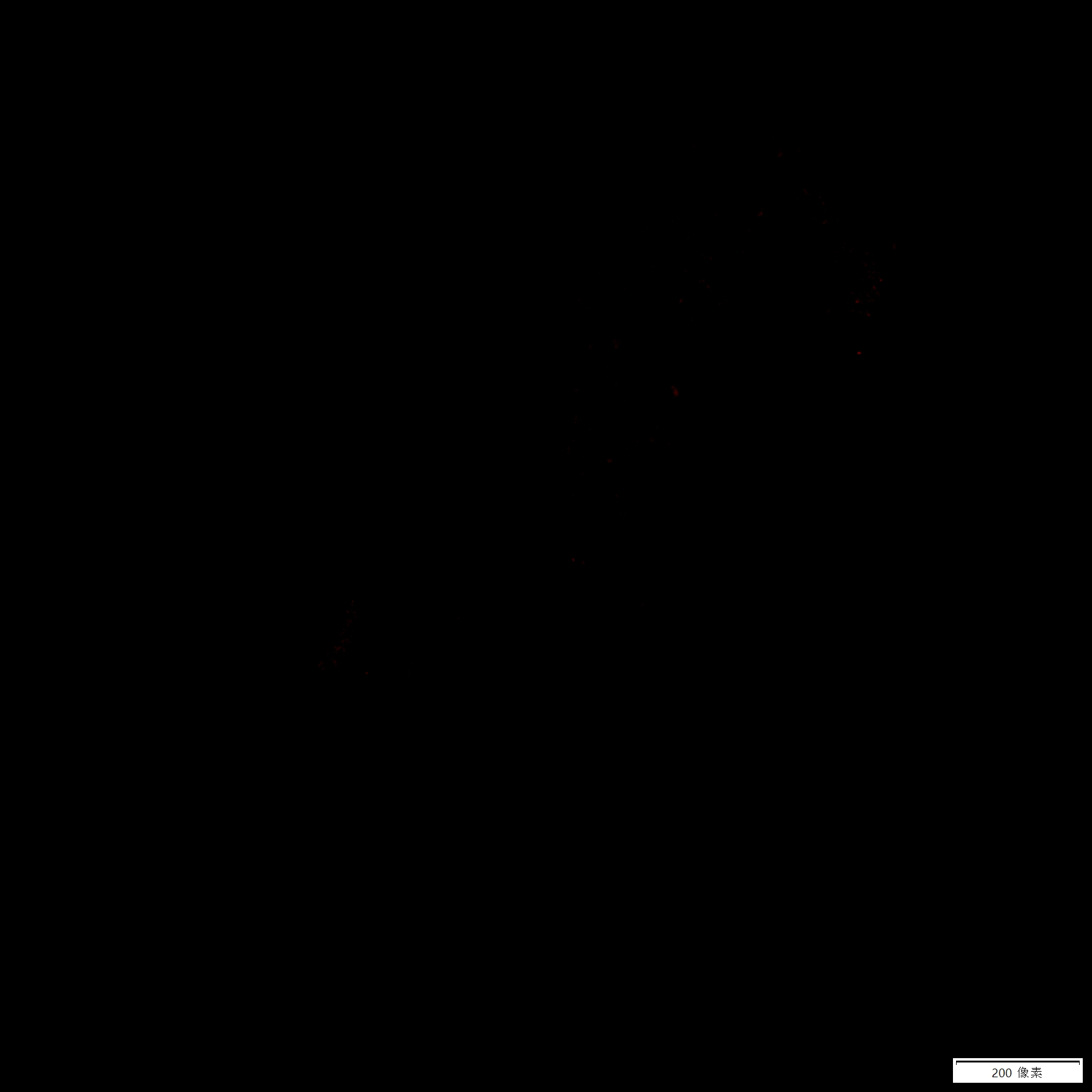

Supplement: Supplementary file 19 — Source data Fig. 3 [file 44318_2025_643_MOESM19_ESM.zip › Figure 3/3G/bmp4 explant_24hpf_HCR_sox2.jpg]

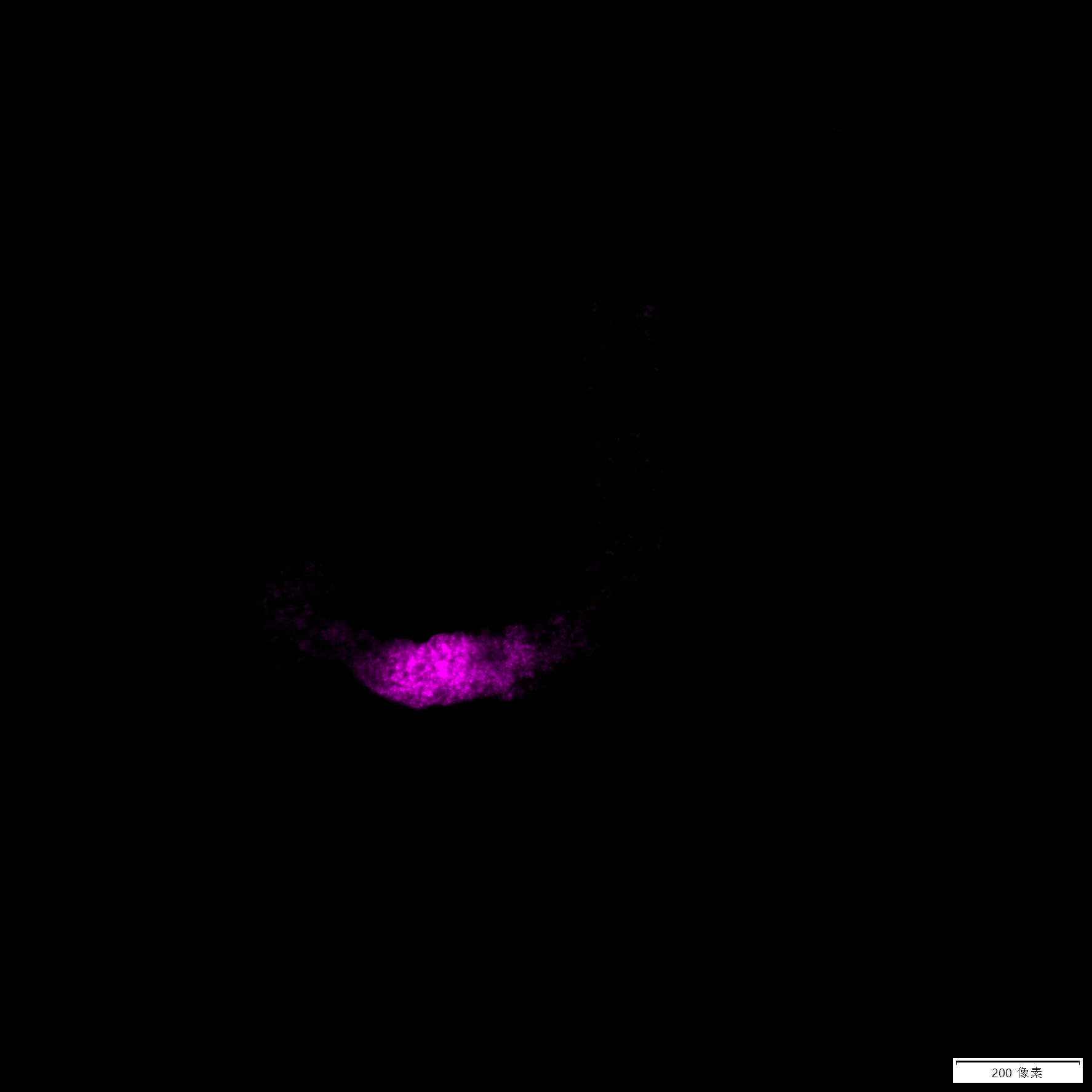

Supplement: Supplementary file 19 — Source data Fig. 3 [file 44318_2025_643_MOESM19_ESM.zip › Figure 3/3G/bmp4 explant_24hpf_HCR_tbx6.jpg]

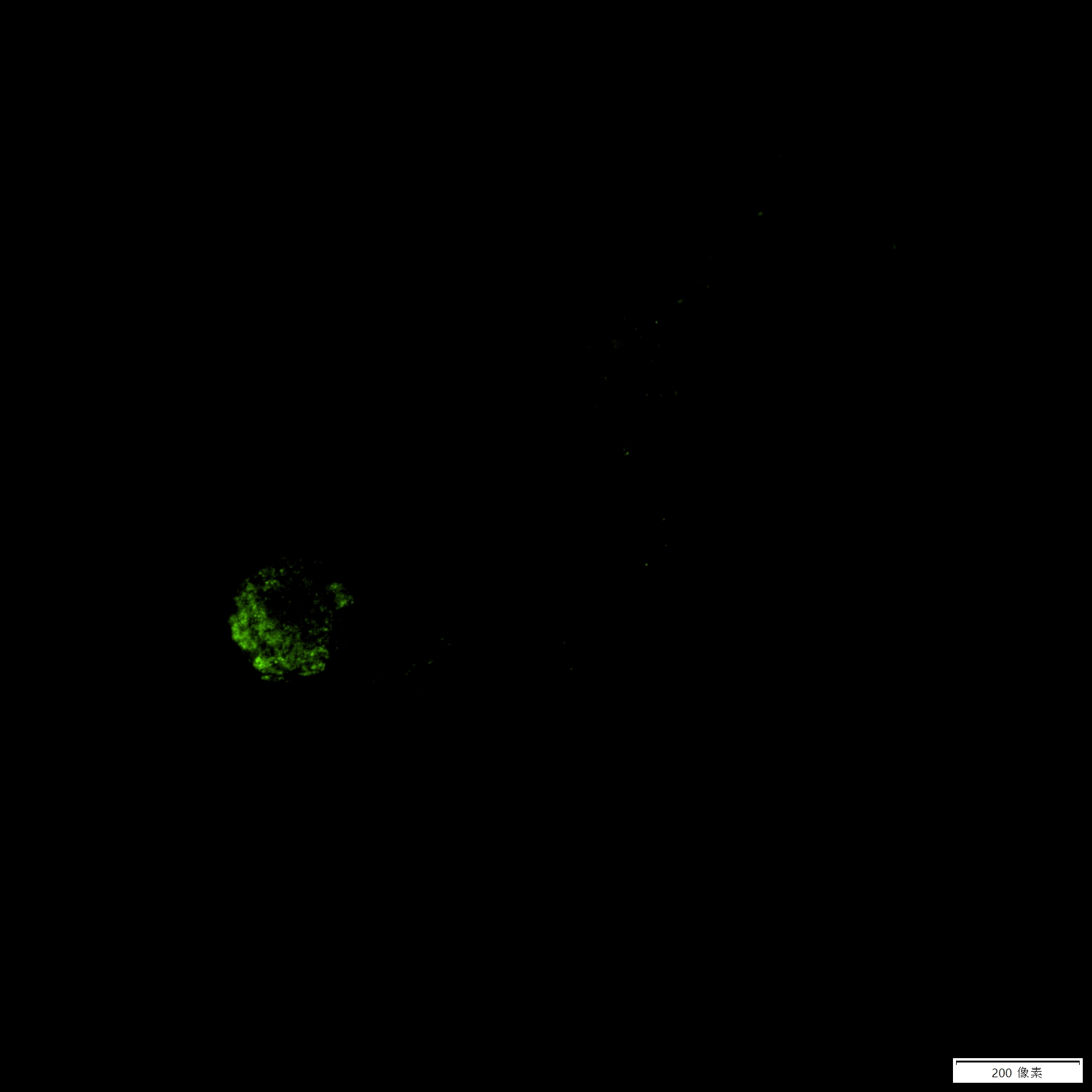

Supplement: Supplementary file 19 — Source data Fig. 3 [file 44318_2025_643_MOESM19_ESM.zip › Figure 3/3G/bmp4 explant_24hpf_HCR_tbxta.jpg]

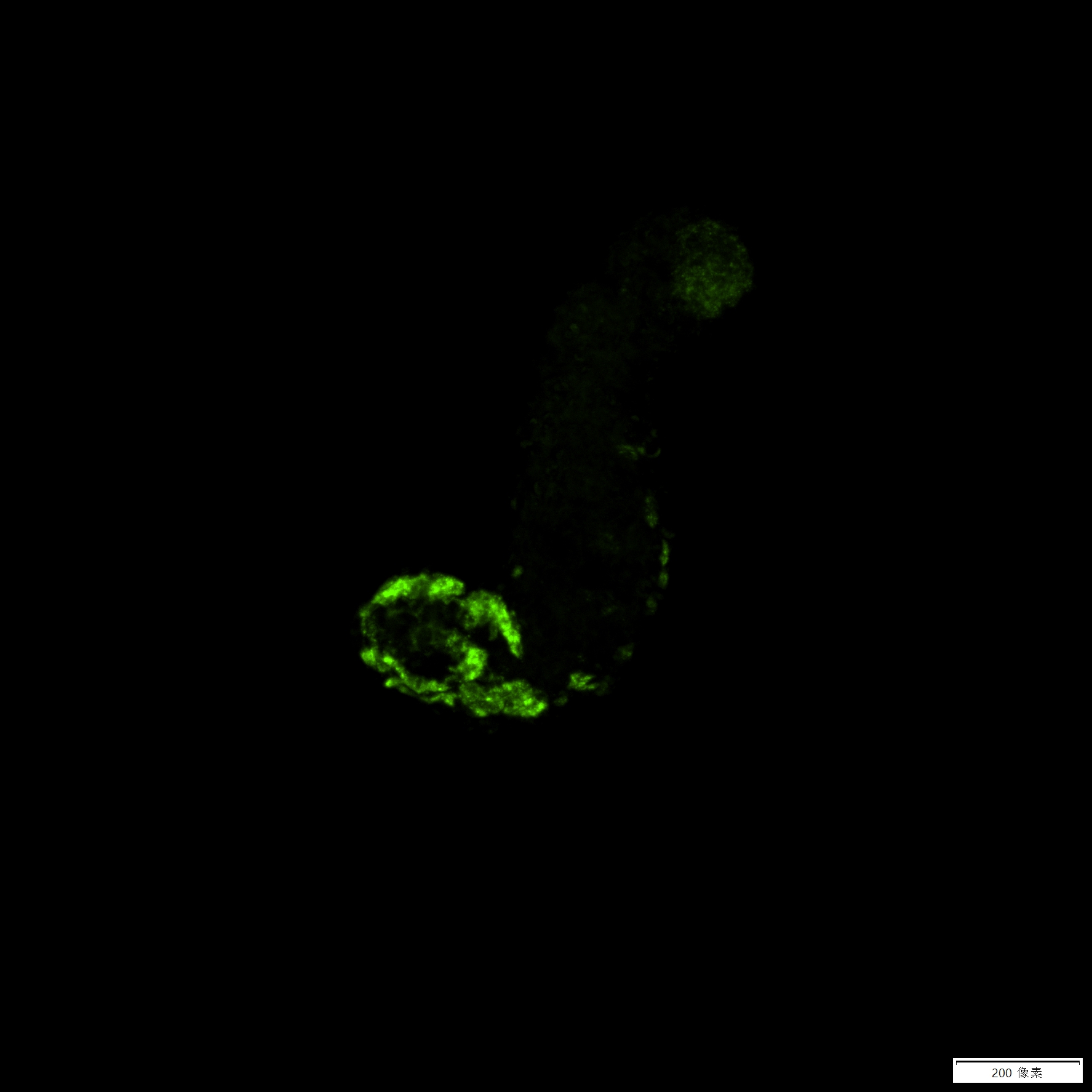

Supplement: Supplementary file 19 — Source data Fig. 3 [file 44318_2025_643_MOESM19_ESM.zip › Figure 3/3H/bmp4 explant_18hpf_HCR_foxd3.jpg]

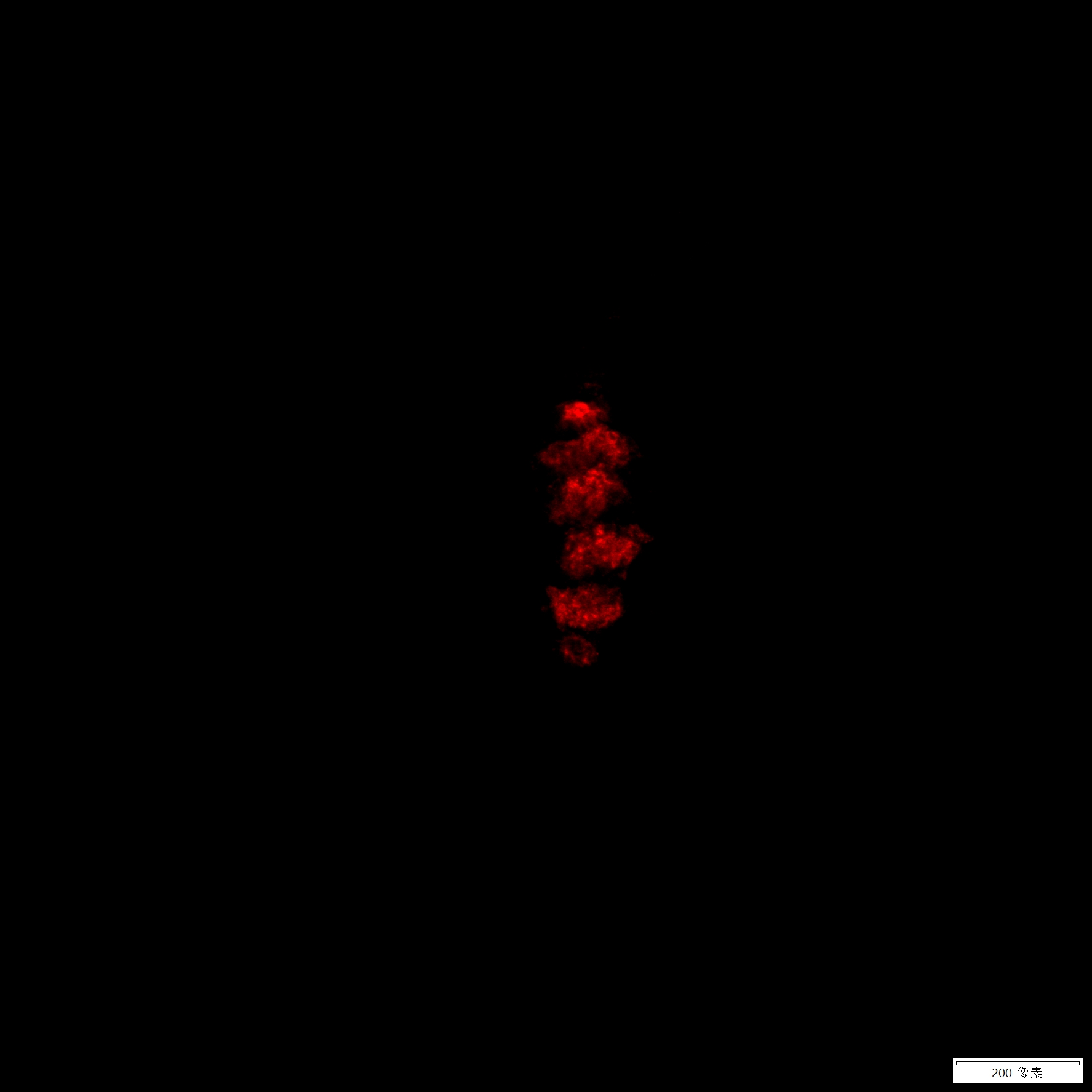

Supplement: Supplementary file 19 — Source data Fig. 3 [file 44318_2025_643_MOESM19_ESM.zip › Figure 3/3H/bmp4 explant_18hpf_HCR_myod1.jpg]

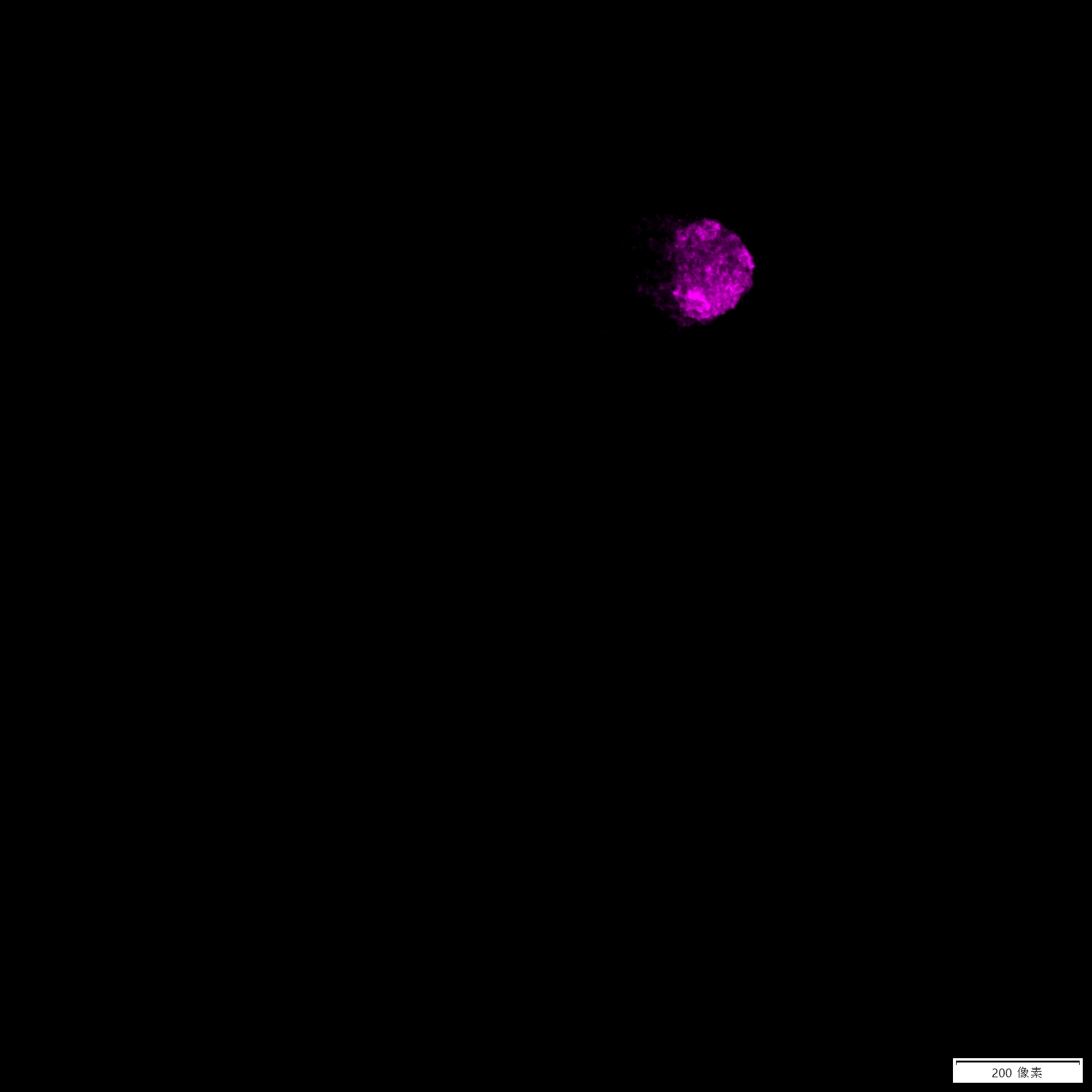

Supplement: Supplementary file 19 — Source data Fig. 3 [file 44318_2025_643_MOESM19_ESM.zip › Figure 3/3H/bmp4 explant_18hpf_HCR_tbxta.jpg]

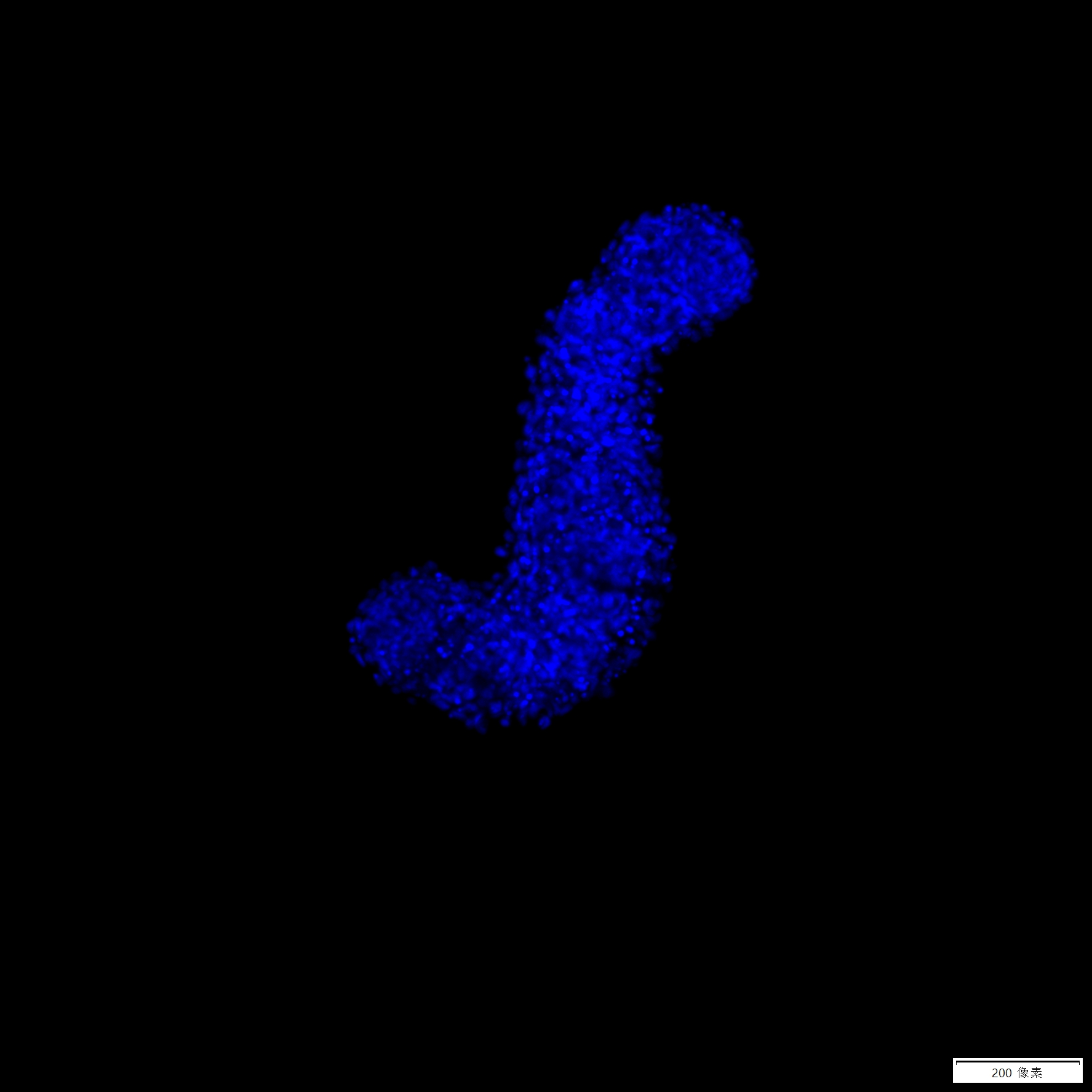

Supplement: Supplementary file 19 — Source data Fig. 3 [file 44318_2025_643_MOESM19_ESM.zip › Figure 3/3H/bmp4 explant_24hpf_DAPI.jpg]

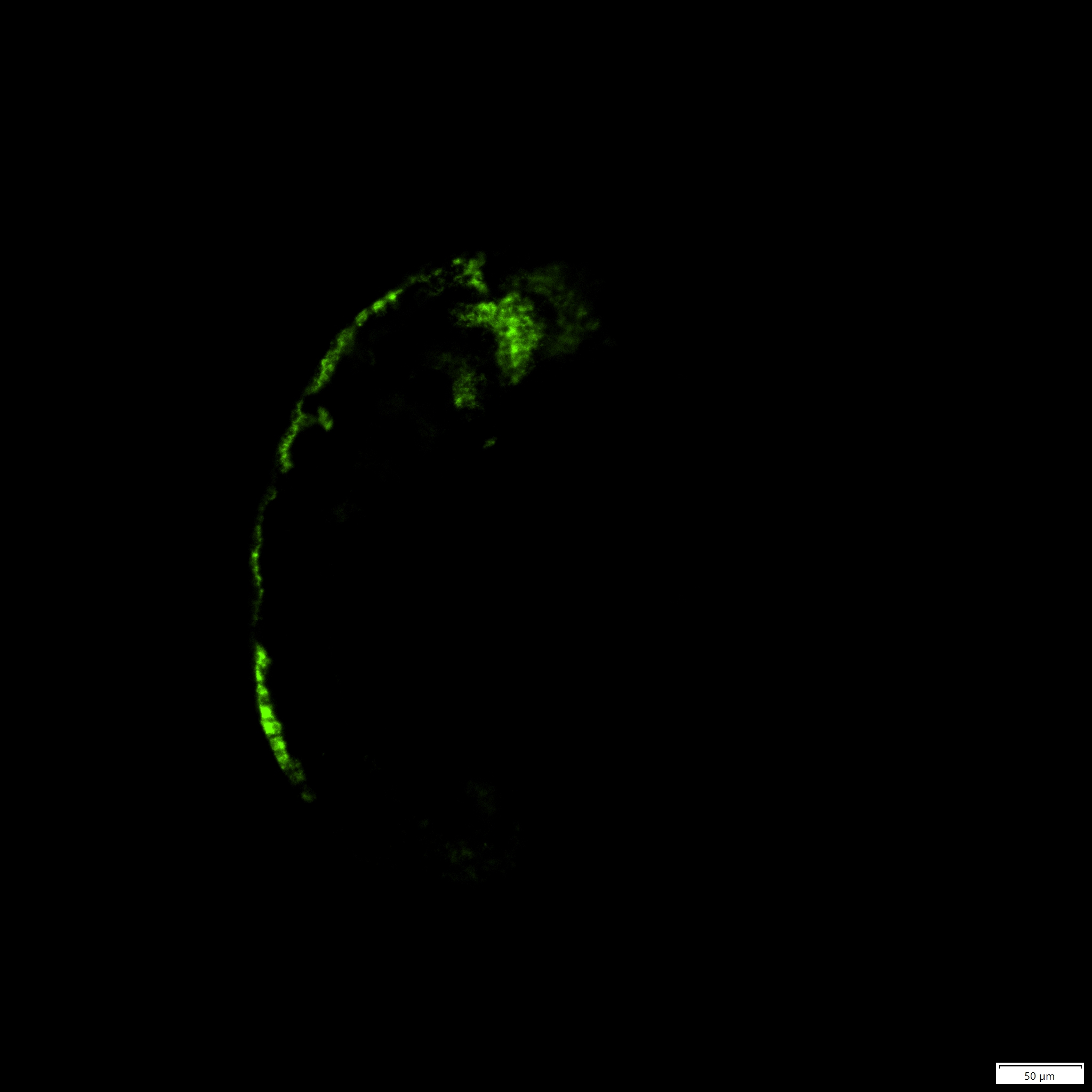

Supplement: Supplementary file 19 — Source data Fig. 3 [file 44318_2025_643_MOESM19_ESM.zip › Figure 3/3H/embryo_18hpf_HCR_foxd3.jpg]

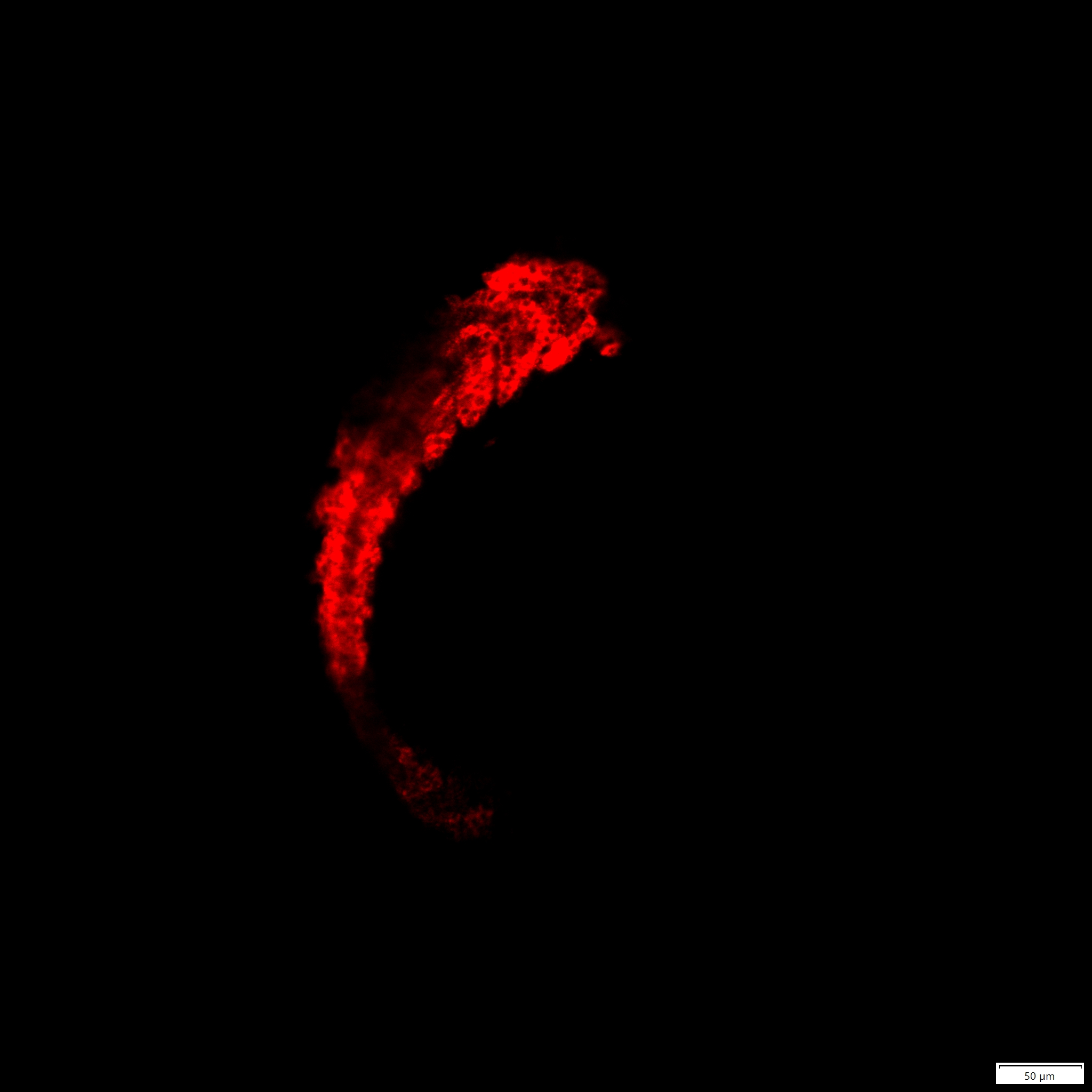

Supplement: Supplementary file 19 — Source data Fig. 3 [file 44318_2025_643_MOESM19_ESM.zip › Figure 3/3H/embryo_18hpf_HCR_myod1.jpg]
