# Supplementary figures and images for: BMP4 initiates and patterns ventral-caudal structures in zebrafish and human pluripotent stem cell aggregates (part 2 of 2)
Source: EMBO J. 2025 Nov 24;45(1):210–42. doi: 10.1038/s44318-025-00643-6 (PMC12759085; doi:10.1038/s44318-025-00643-6)

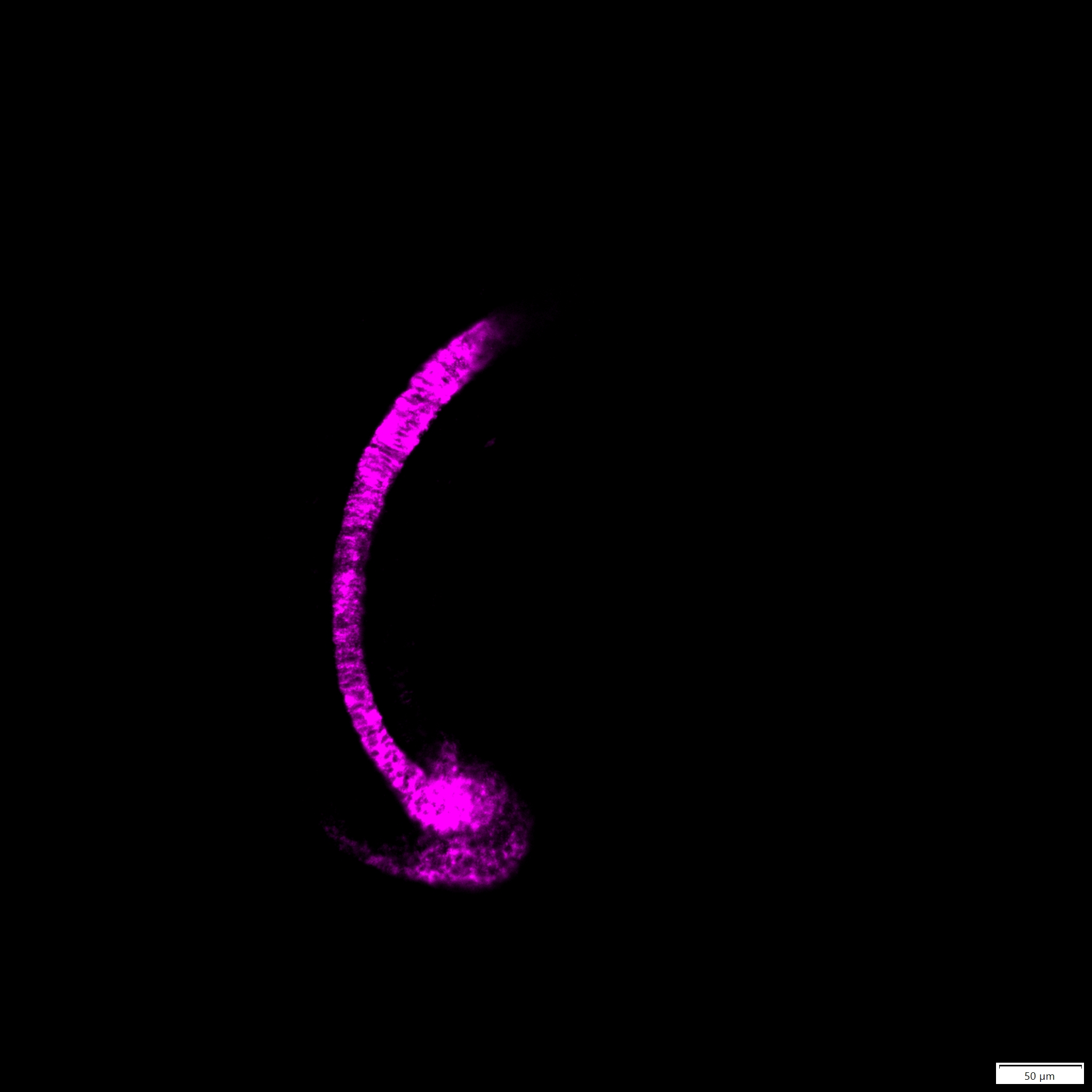

Supplement: Supplementary file 19 — Source data Fig. 3 [file 44318_2025_643_MOESM19_ESM.zip › Figure 3/3H/embryo_18hpf_HCR_tbxta.jpg]

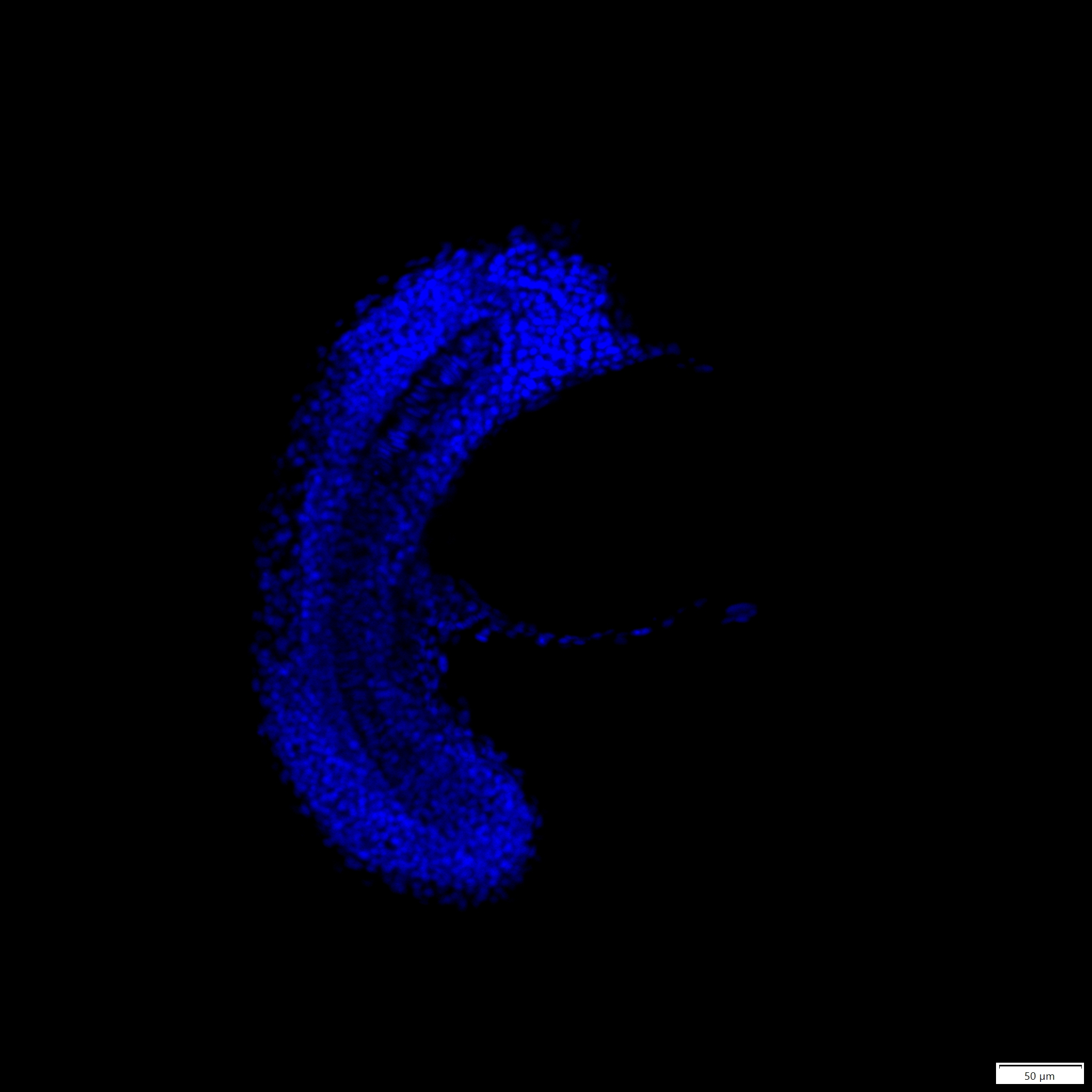

Supplement: Supplementary file 19 — Source data Fig. 3 [file 44318_2025_643_MOESM19_ESM.zip › Figure 3/3H/embryo_24hpf_DAPI.jpg]

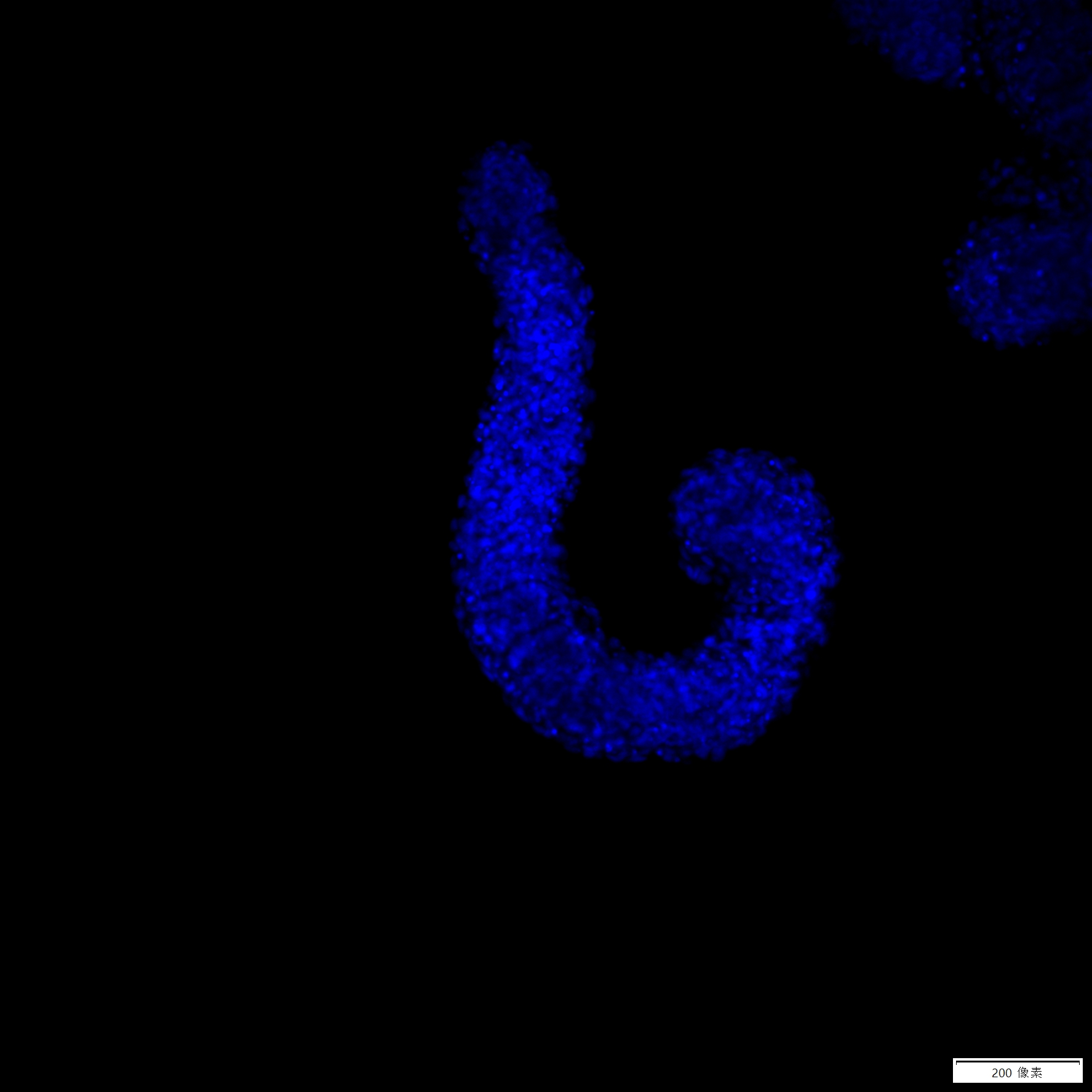

Supplement: Supplementary file 19 — Source data Fig. 3 [file 44318_2025_643_MOESM19_ESM.zip › Figure 3/3I/bmp4 explant_24hpf_DAPI.jpg]

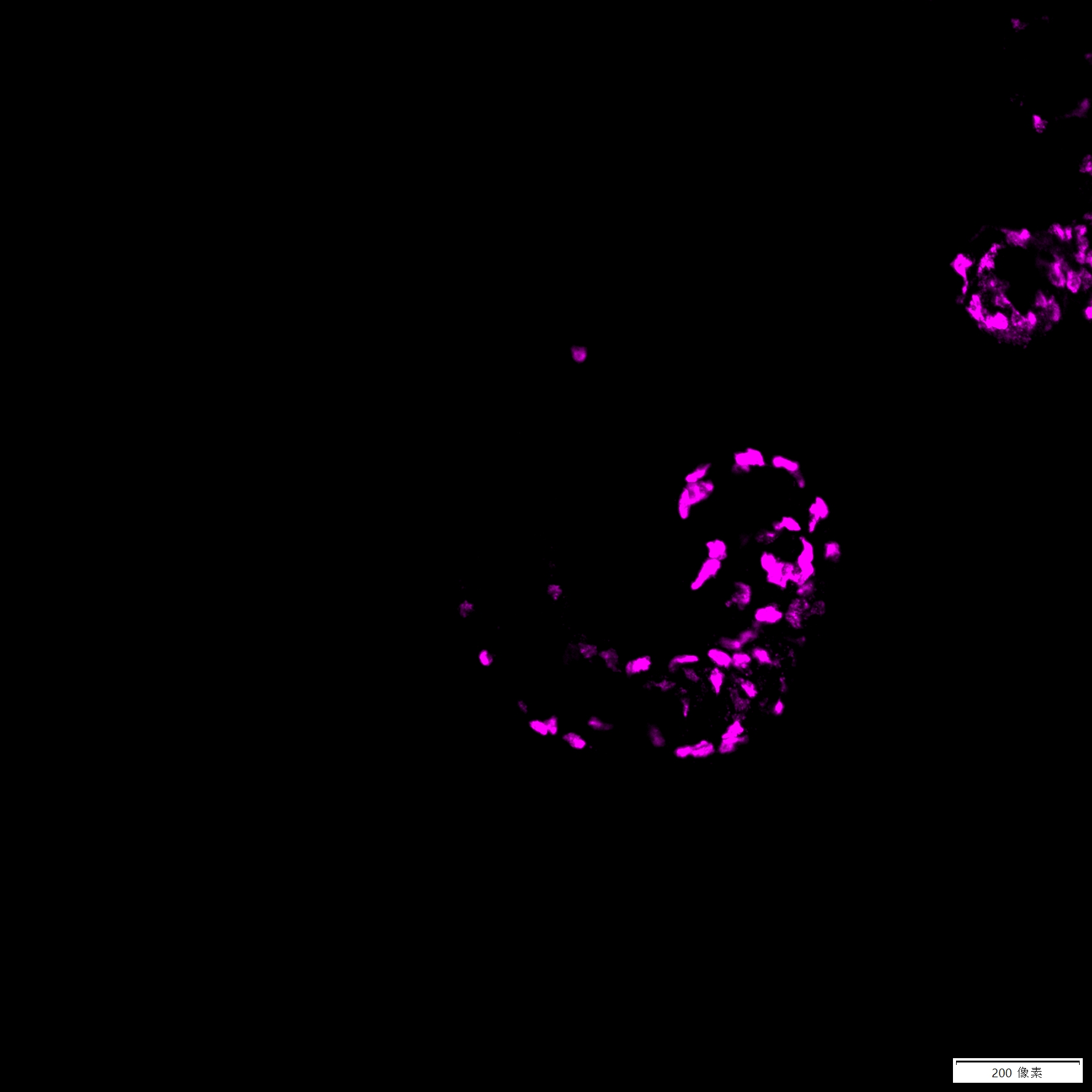

Supplement: Supplementary file 19 — Source data Fig. 3 [file 44318_2025_643_MOESM19_ESM.zip › Figure 3/3I/bmp4 explant_24hpf_HCR_elavl3.jpg]

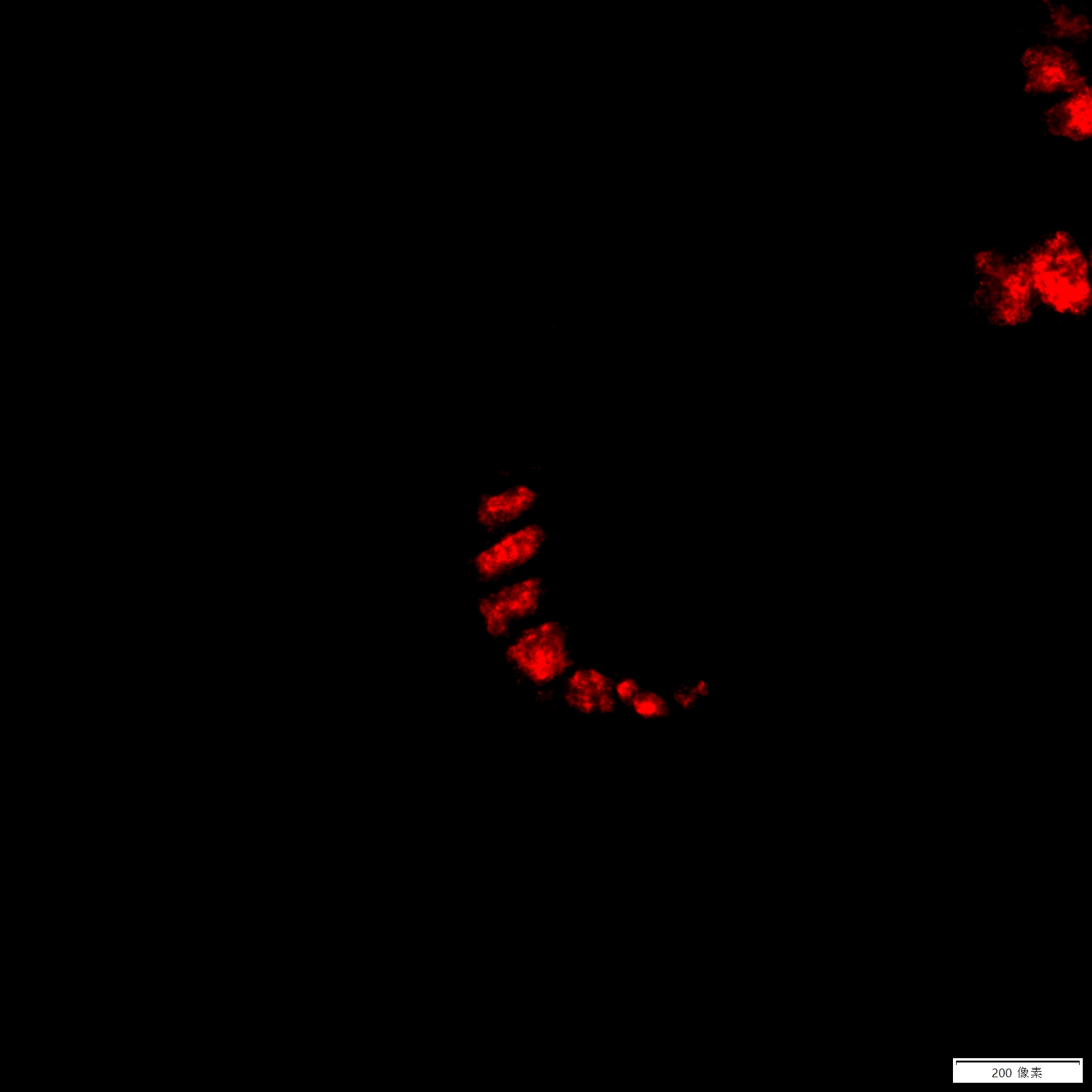

Supplement: Supplementary file 19 — Source data Fig. 3 [file 44318_2025_643_MOESM19_ESM.zip › Figure 3/3I/bmp4 explant_24hpf_HCR_myod1.jpg]

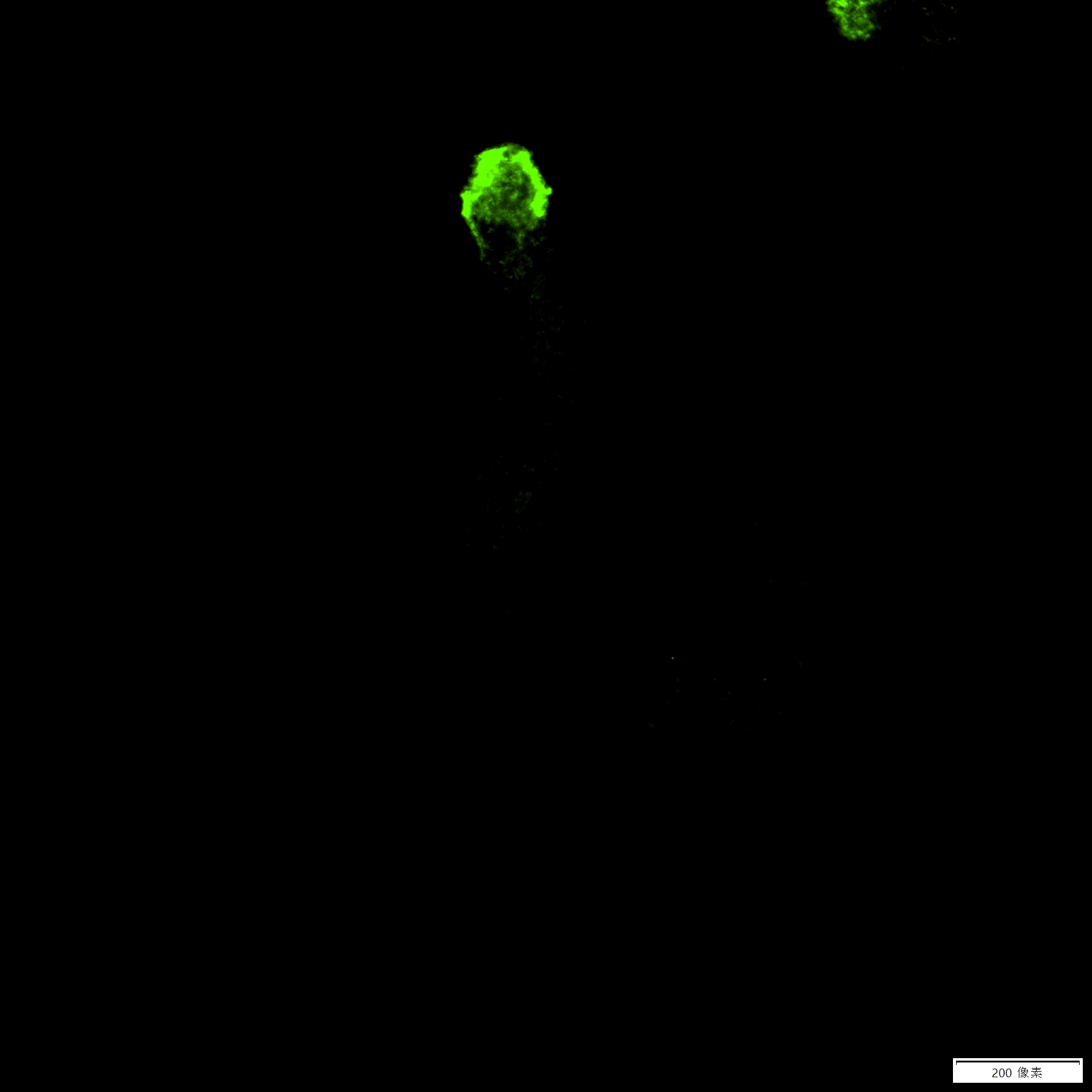

Supplement: Supplementary file 19 — Source data Fig. 3 [file 44318_2025_643_MOESM19_ESM.zip › Figure 3/3I/bmp4 explant_24hpf_HCR_tbxta.jpg]

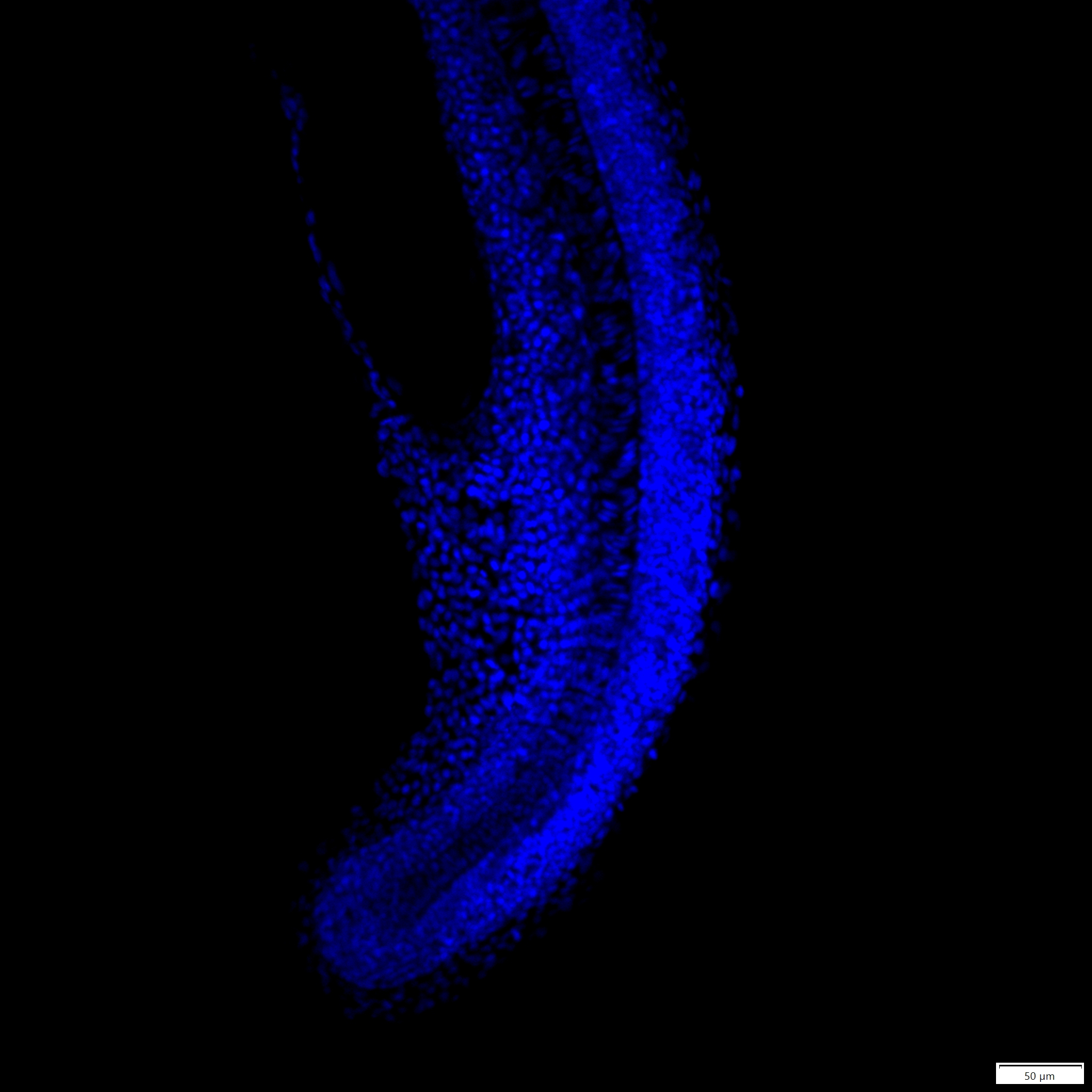

Supplement: Supplementary file 19 — Source data Fig. 3 [file 44318_2025_643_MOESM19_ESM.zip › Figure 3/3I/embryo_24hpf_DAPI.jpg]

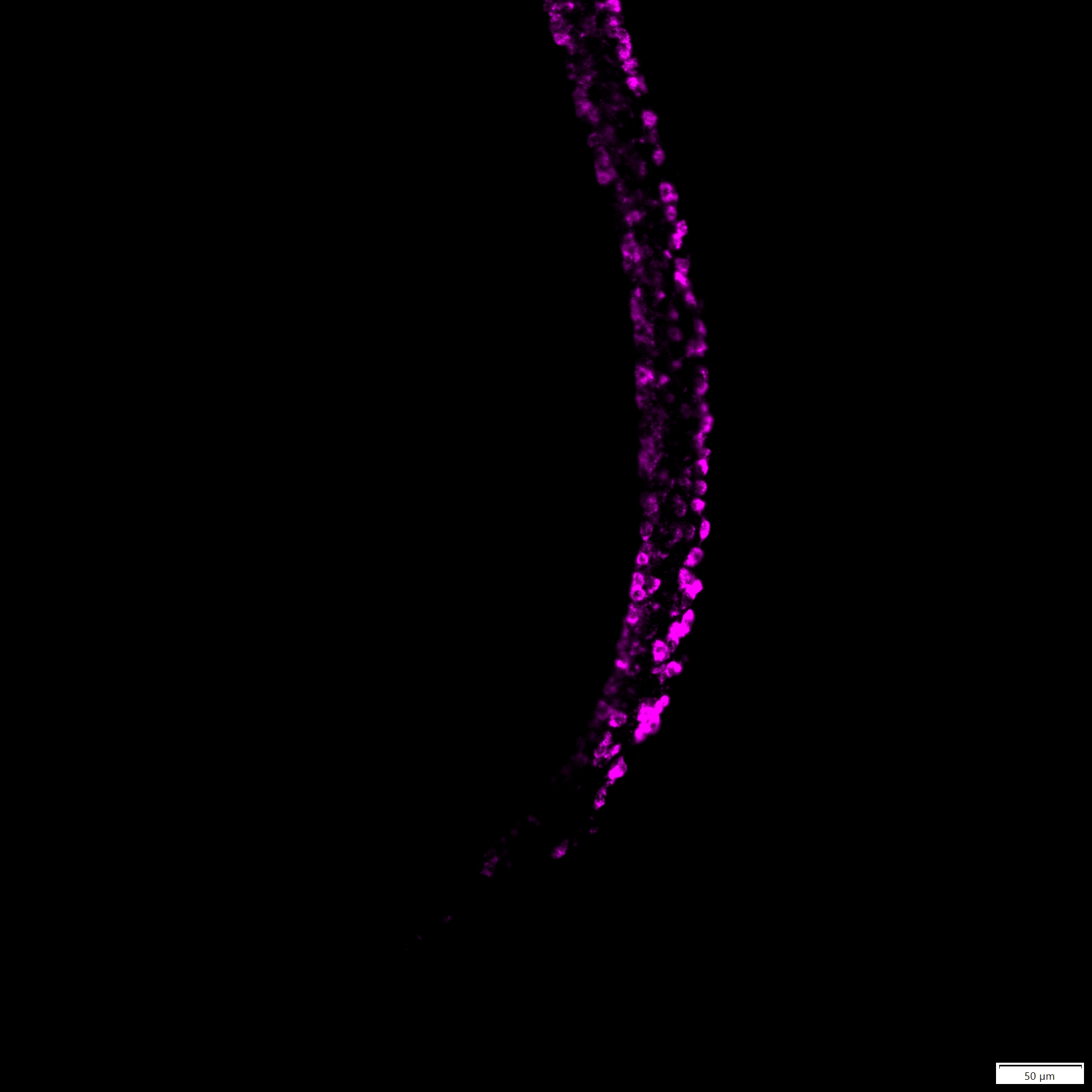

Supplement: Supplementary file 19 — Source data Fig. 3 [file 44318_2025_643_MOESM19_ESM.zip › Figure 3/3I/embryo_24hpf_HCR_elavl3.jpg]

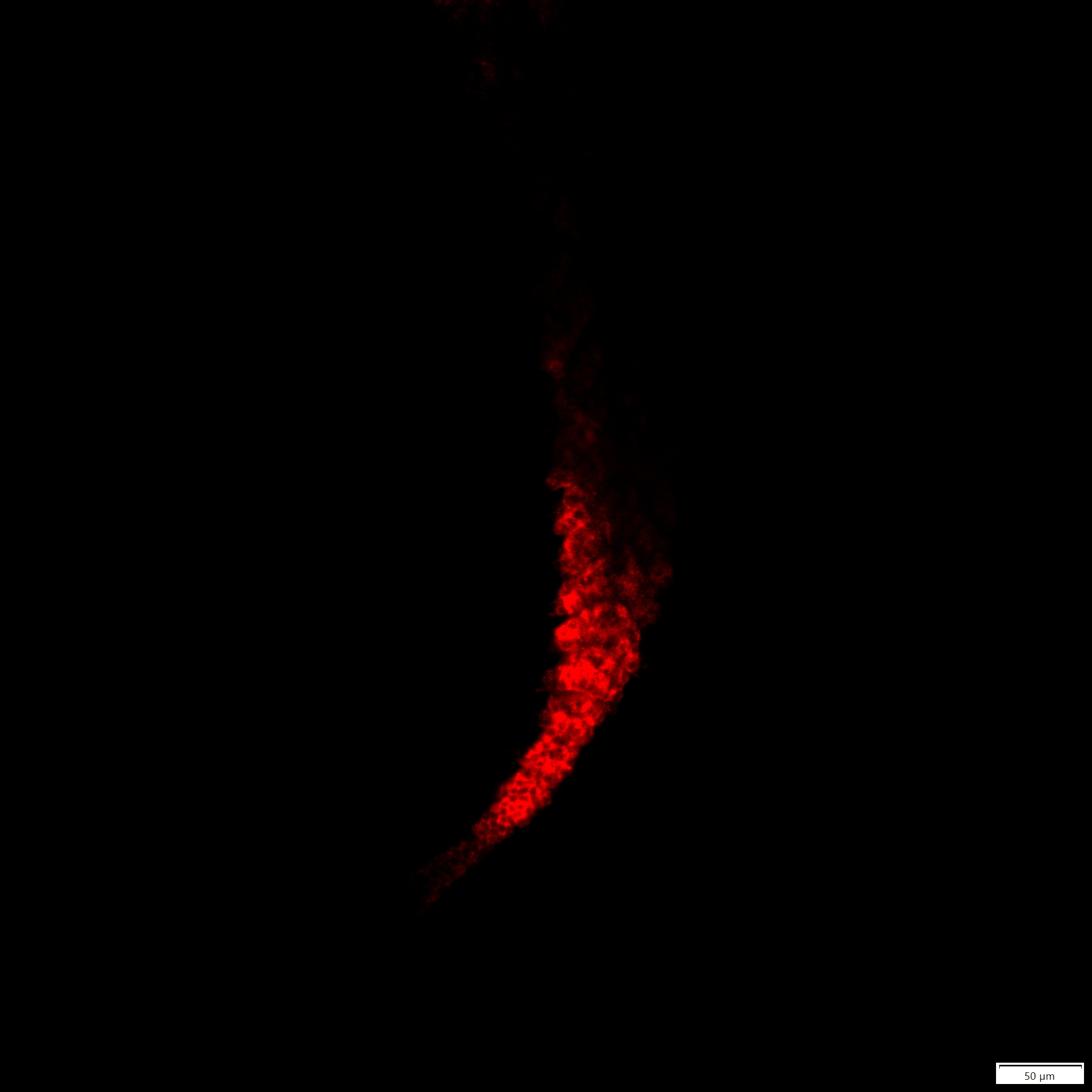

Supplement: Supplementary file 19 — Source data Fig. 3 [file 44318_2025_643_MOESM19_ESM.zip › Figure 3/3I/embryo_24hpf_HCR_myod1.jpg]

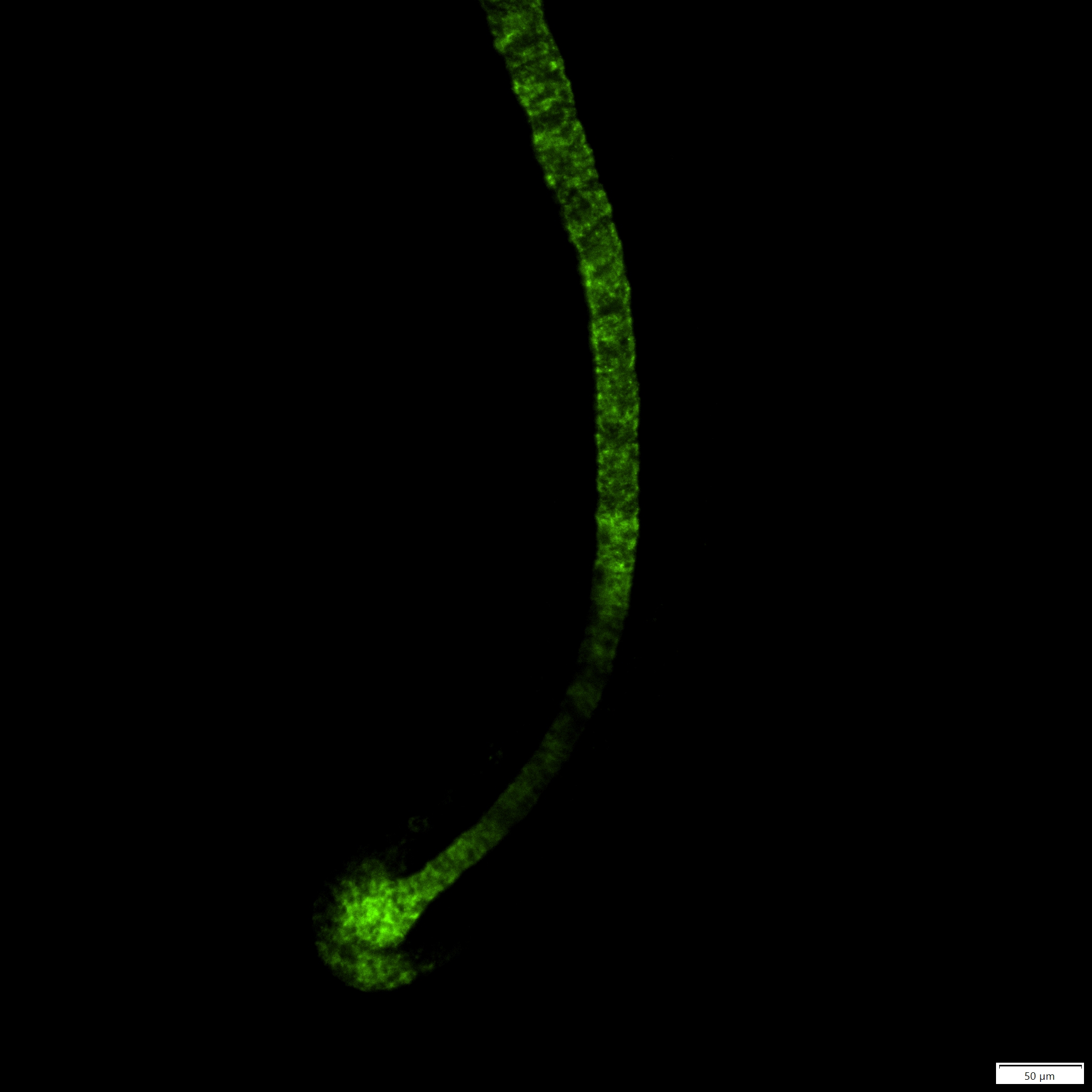

Supplement: Supplementary file 19 — Source data Fig. 3 [file 44318_2025_643_MOESM19_ESM.zip › Figure 3/3I/embryo_24hpf_HCR_tbxta.jpg]

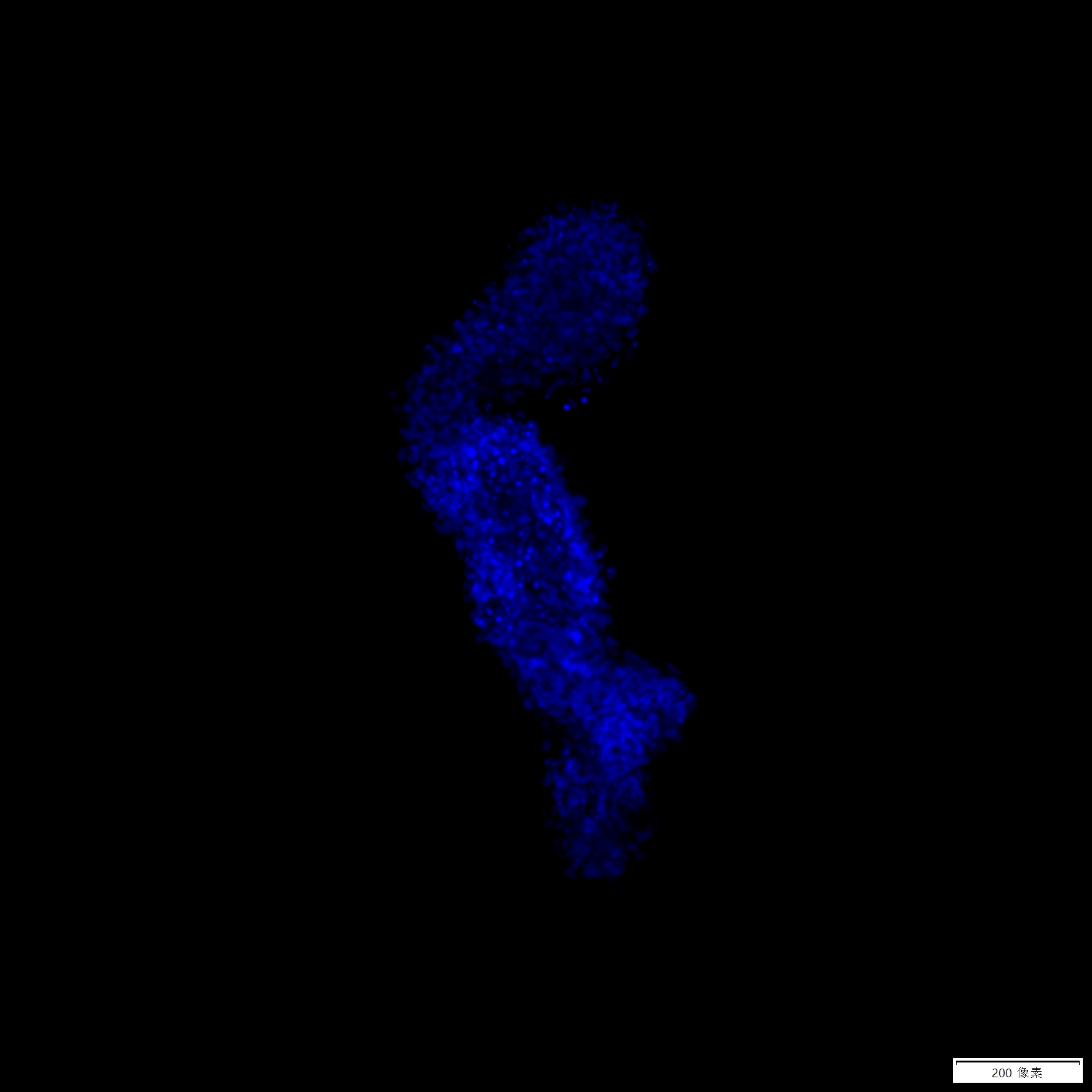

Supplement: Supplementary file 19 — Source data Fig. 3 [file 44318_2025_643_MOESM19_ESM.zip › Figure 3/3J/bmp4 explant_24hpf_DAPI.jpg]

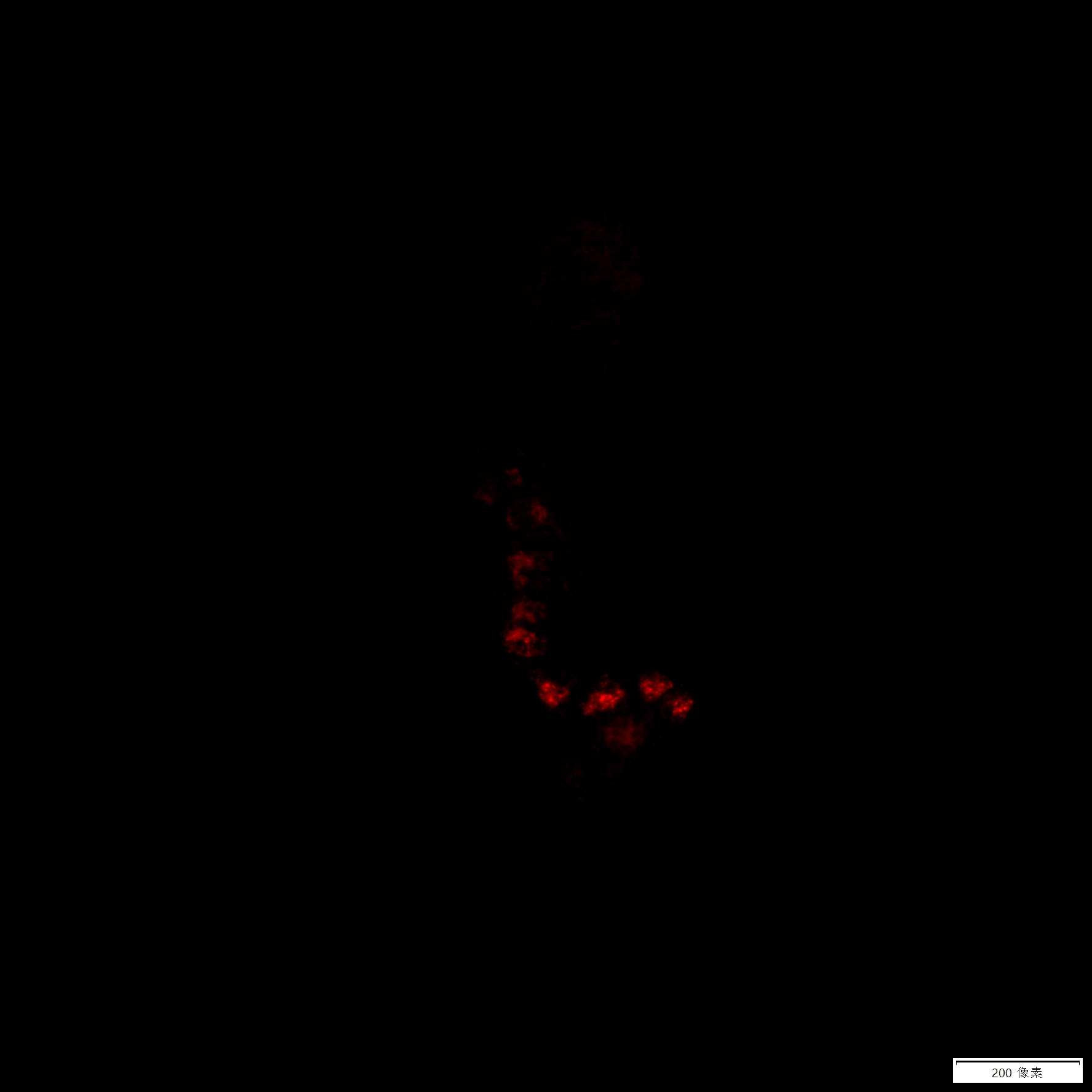

Supplement: Supplementary file 19 — Source data Fig. 3 [file 44318_2025_643_MOESM19_ESM.zip › Figure 3/3J/bmp4 explant_24hpf_HCR_myod1.jpg]

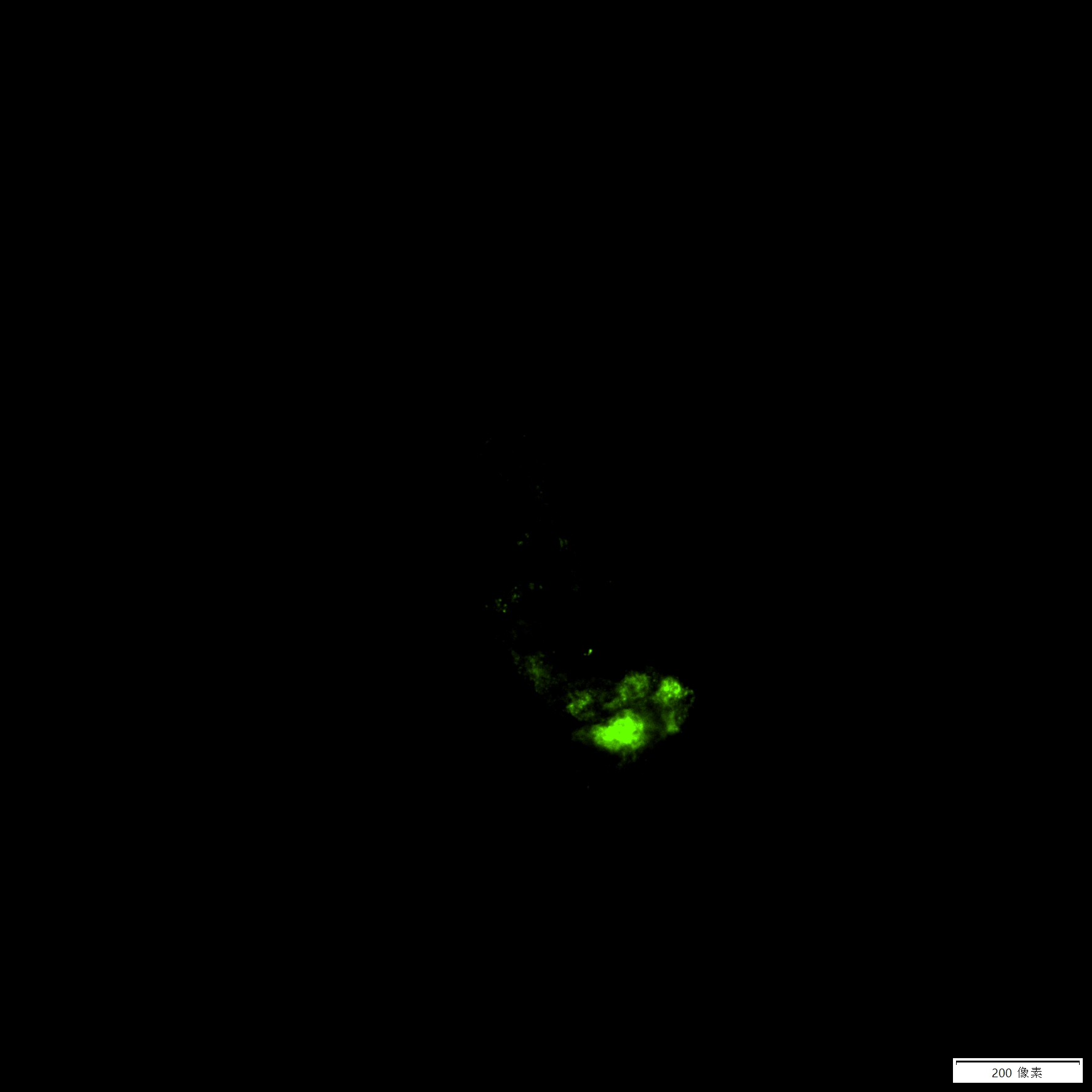

Supplement: Supplementary file 19 — Source data Fig. 3 [file 44318_2025_643_MOESM19_ESM.zip › Figure 3/3J/bmp4 explant_24hpf_HCR_ripply1.jpg]

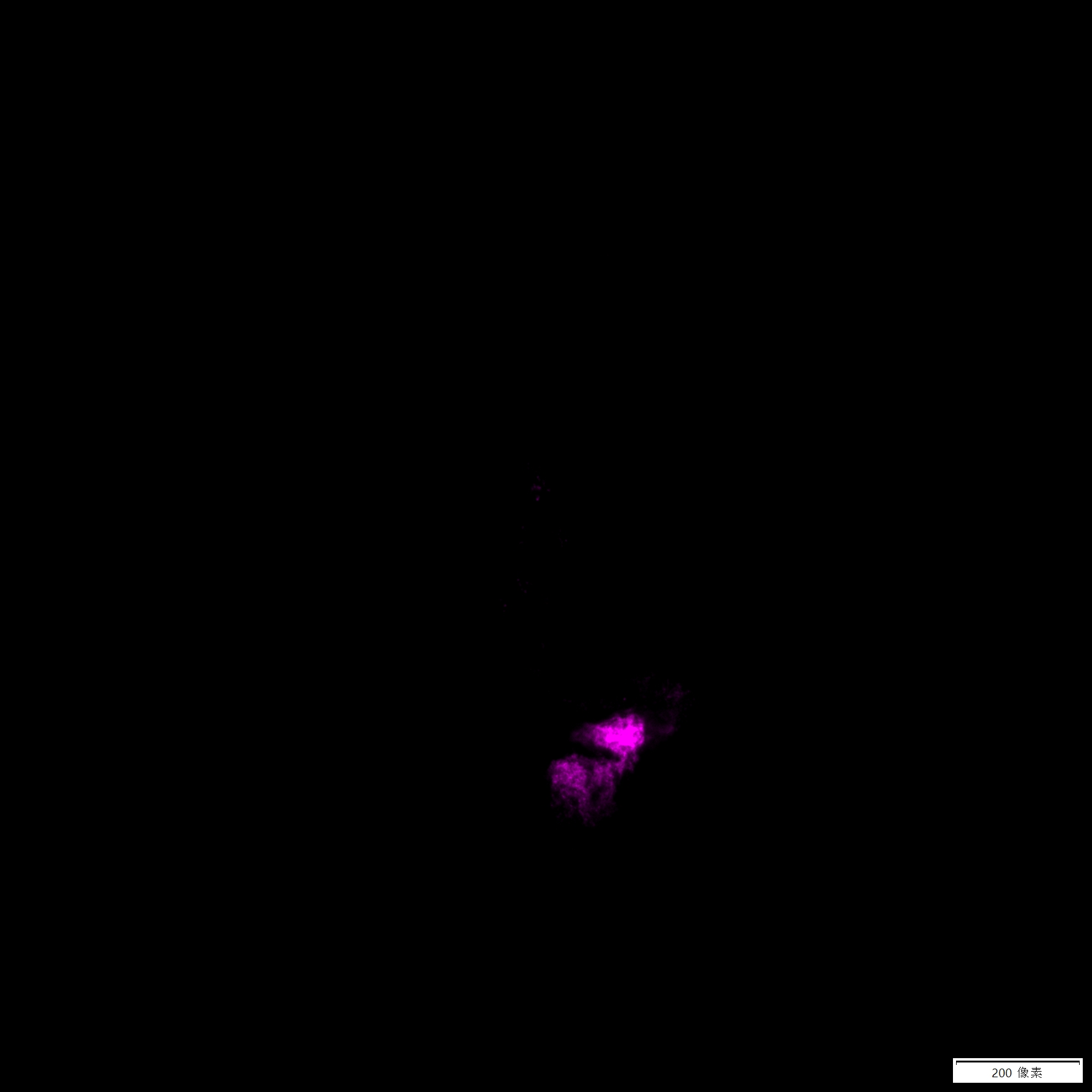

Supplement: Supplementary file 19 — Source data Fig. 3 [file 44318_2025_643_MOESM19_ESM.zip › Figure 3/3J/bmp4 explant_24hpf_HCR_tbx6.jpg]

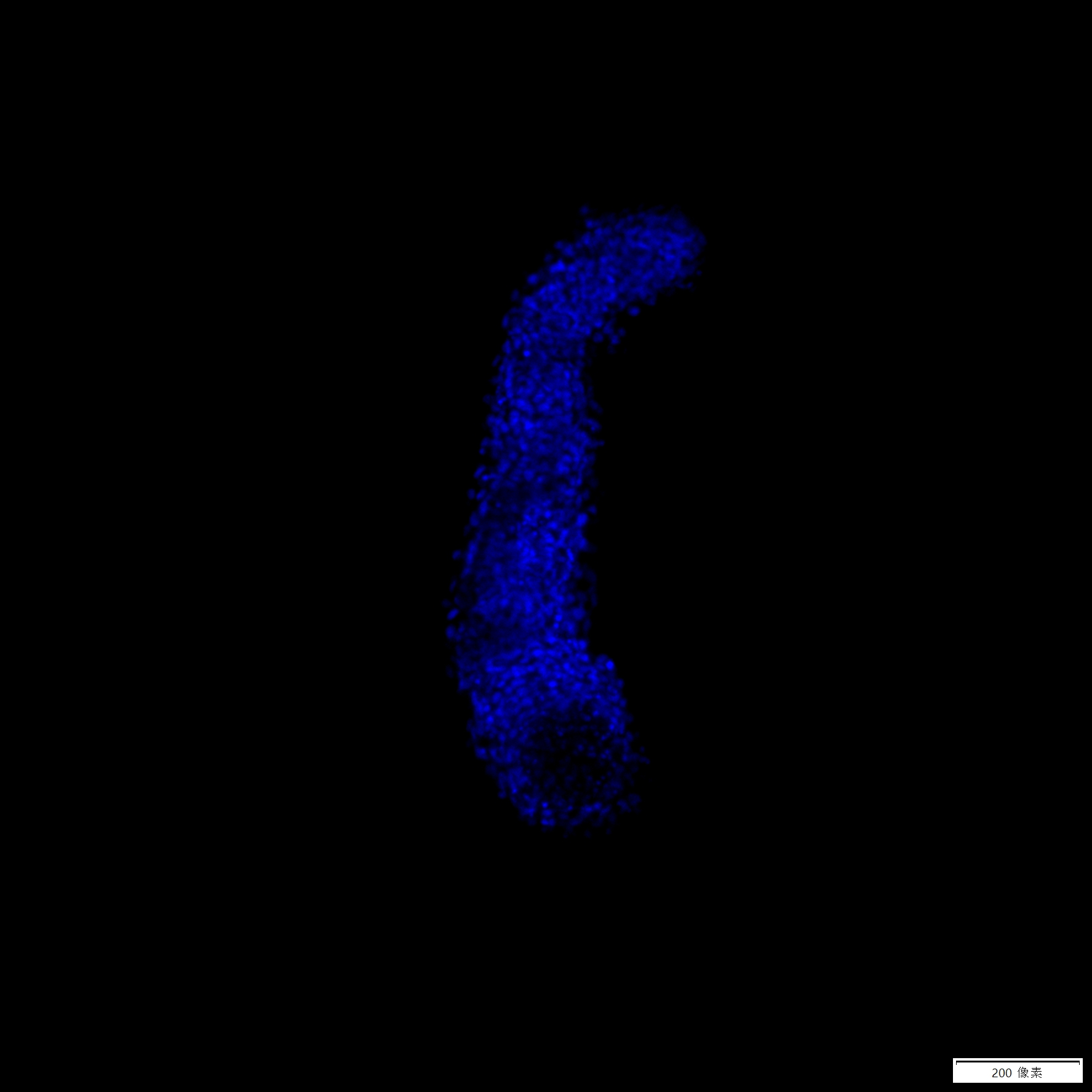

Supplement: Supplementary file 19 — Source data Fig. 3 [file 44318_2025_643_MOESM19_ESM.zip › Figure 3/3K/bmp4 explant_24hpf_DAPI.jpg]

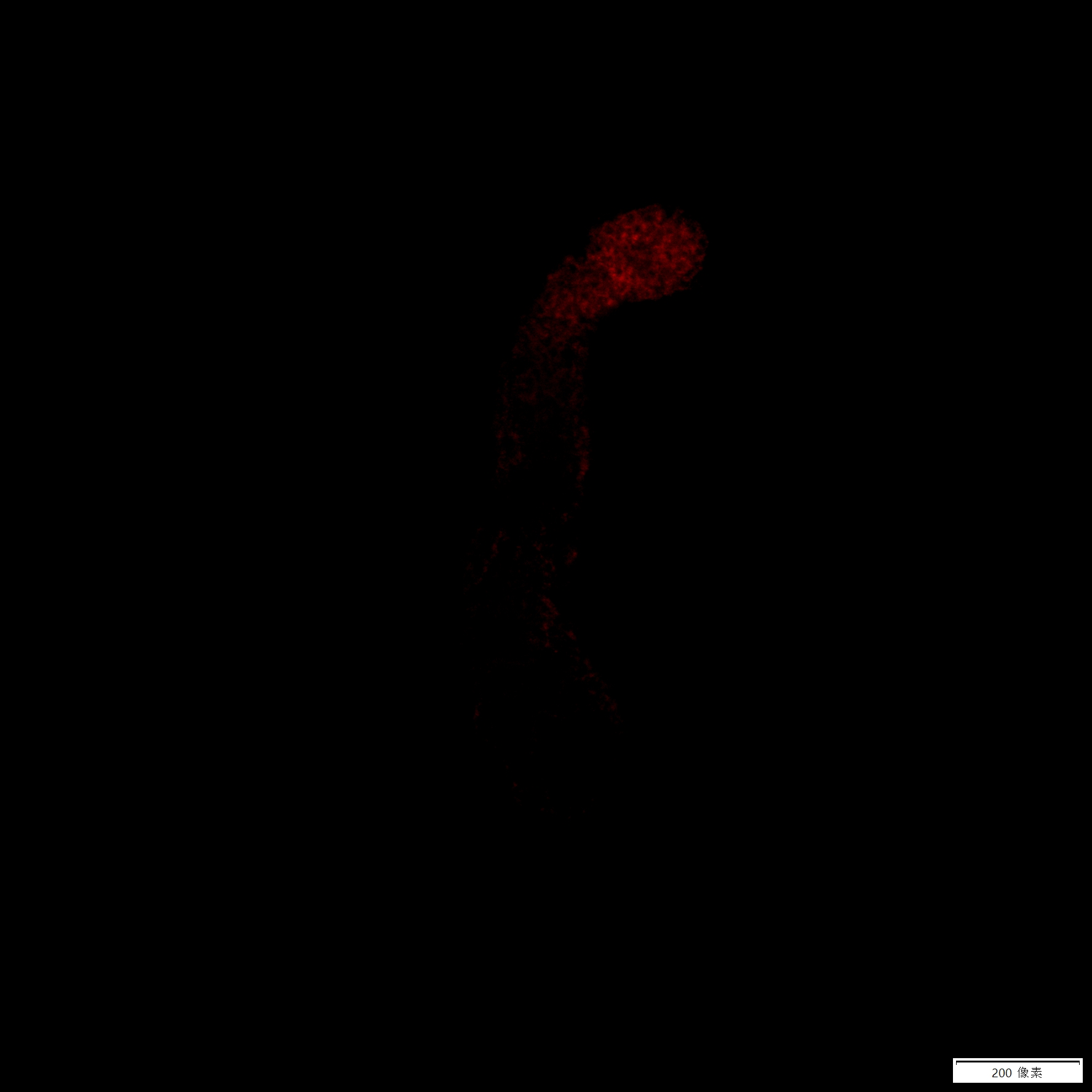

Supplement: Supplementary file 19 — Source data Fig. 3 [file 44318_2025_643_MOESM19_ESM.zip › Figure 3/3K/bmp4 explant_24hpf_HCR_cdx4.jpg]

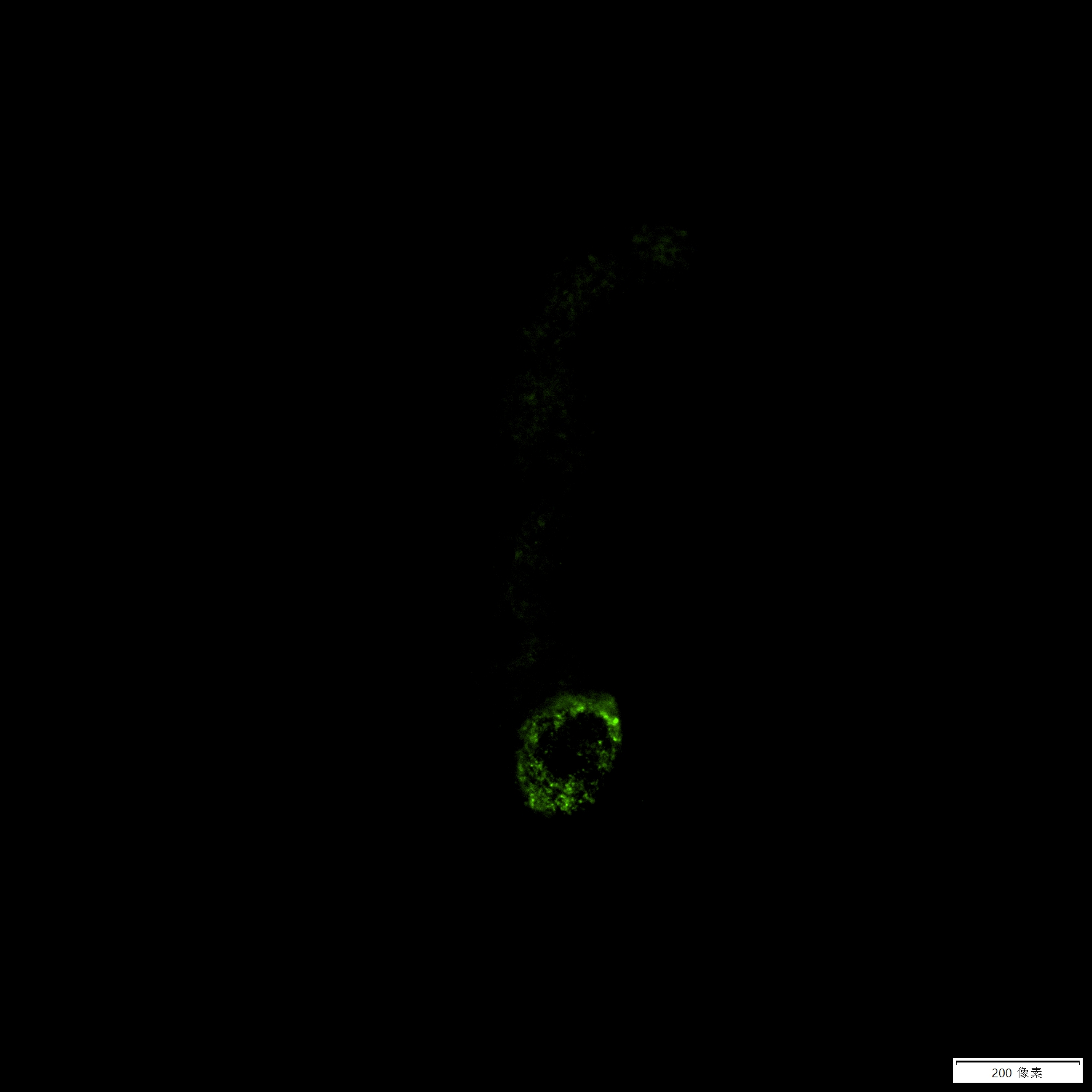

Supplement: Supplementary file 19 — Source data Fig. 3 [file 44318_2025_643_MOESM19_ESM.zip › Figure 3/3K/bmp4 explant_24hpf_HCR_gata5.jpg]

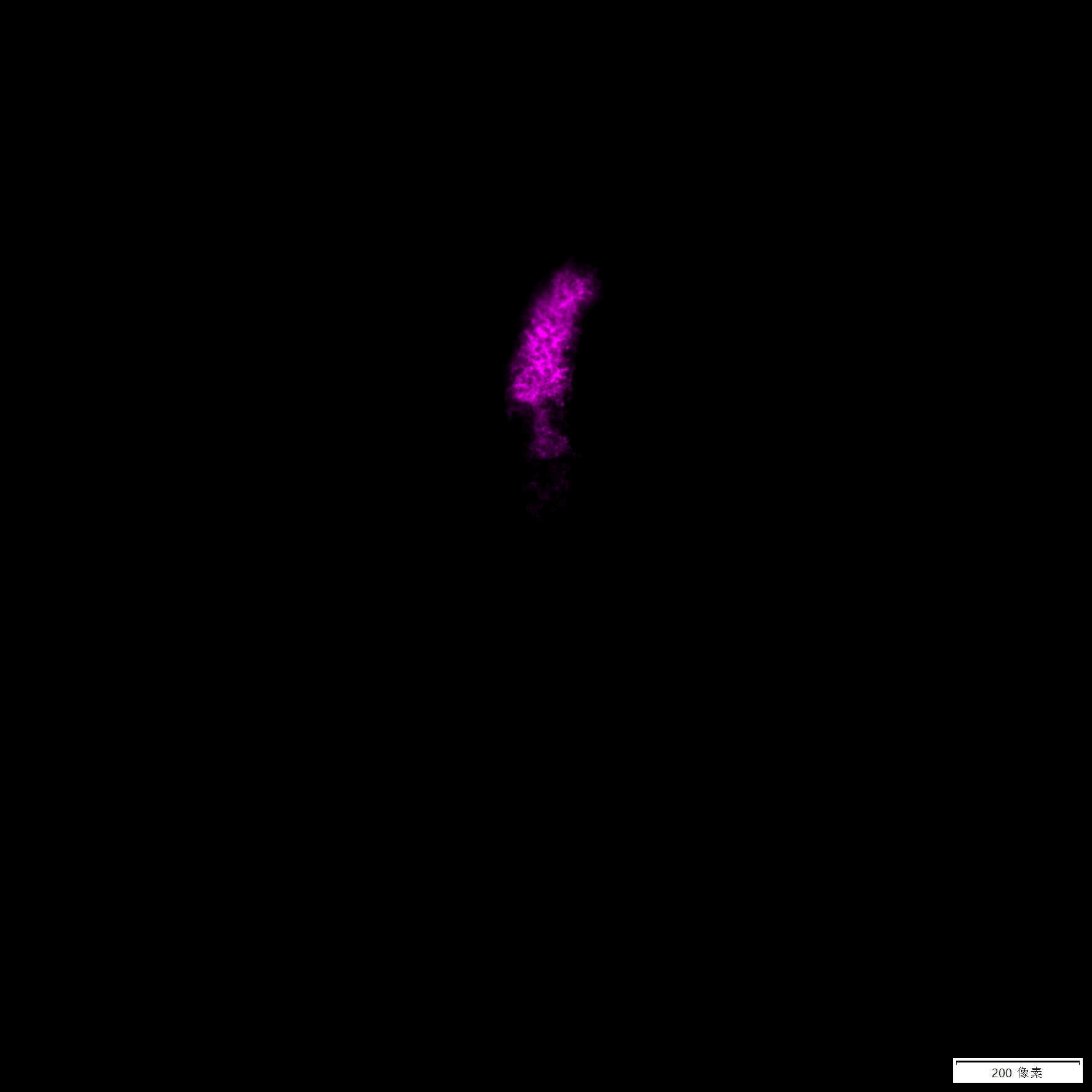

Supplement: Supplementary file 19 — Source data Fig. 3 [file 44318_2025_643_MOESM19_ESM.zip › Figure 3/3K/bmp4 explant_24hpf_HCR_tbx6.jpg]

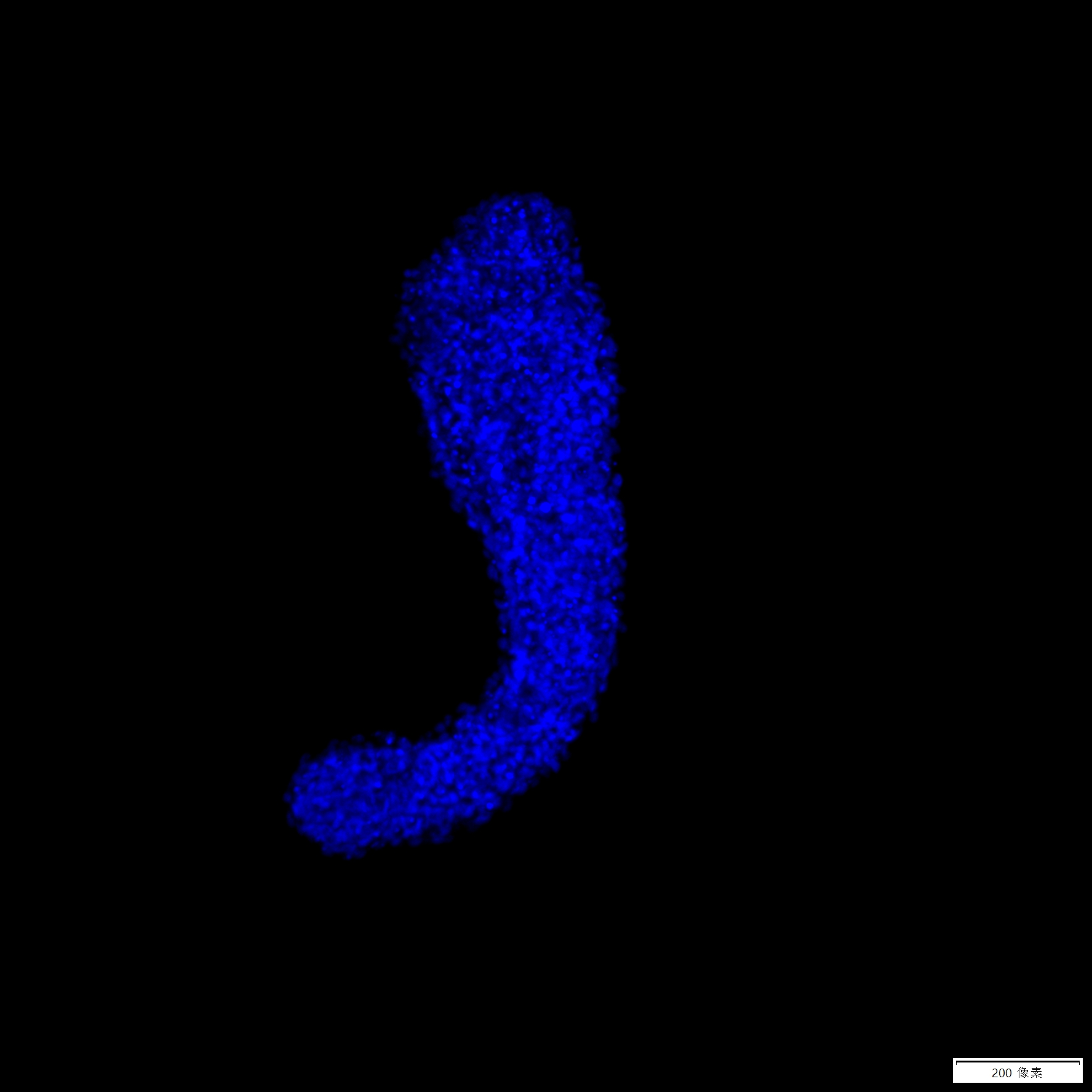

Supplement: Supplementary file 19 — Source data Fig. 3 [file 44318_2025_643_MOESM19_ESM.zip › Figure 3/3L/Bmp4 explant_24hpf_DAPI.jpg]

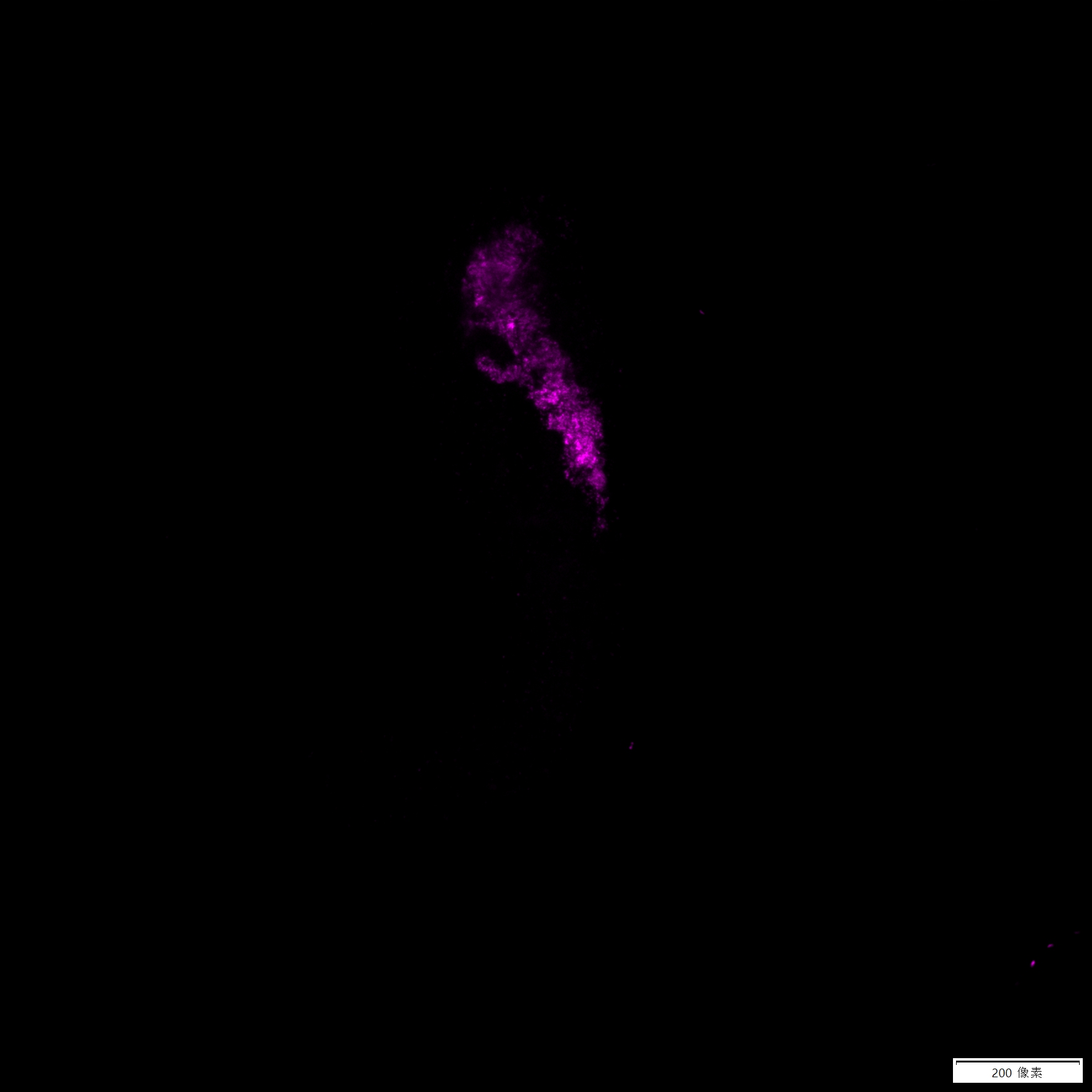

Supplement: Supplementary file 19 — Source data Fig. 3 [file 44318_2025_643_MOESM19_ESM.zip › Figure 3/3L/Bmp4 explant_24hpf_HCR_cldn3d.jpg]

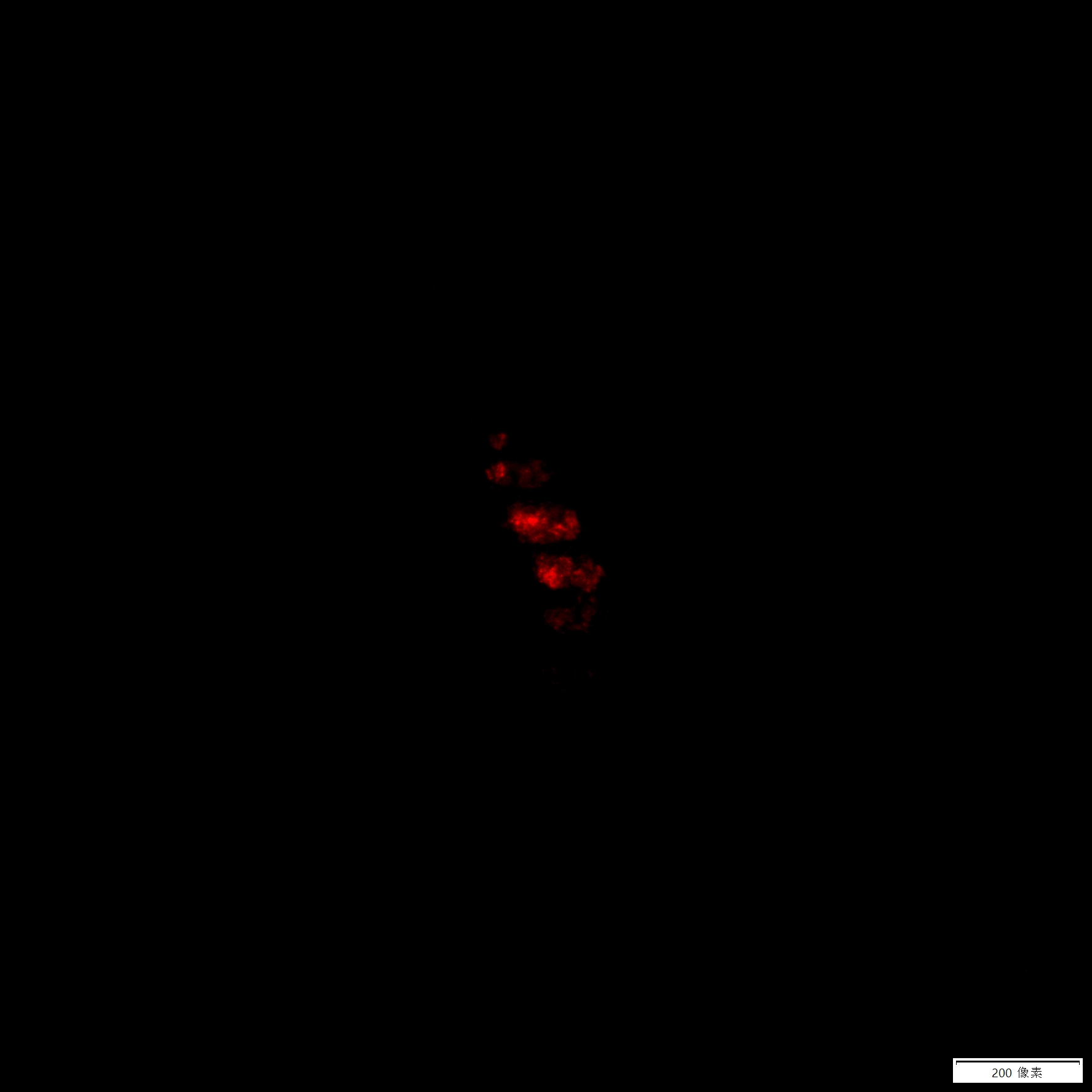

Supplement: Supplementary file 19 — Source data Fig. 3 [file 44318_2025_643_MOESM19_ESM.zip › Figure 3/3L/Bmp4 explant_24hpf_HCR_myod1.jpg]

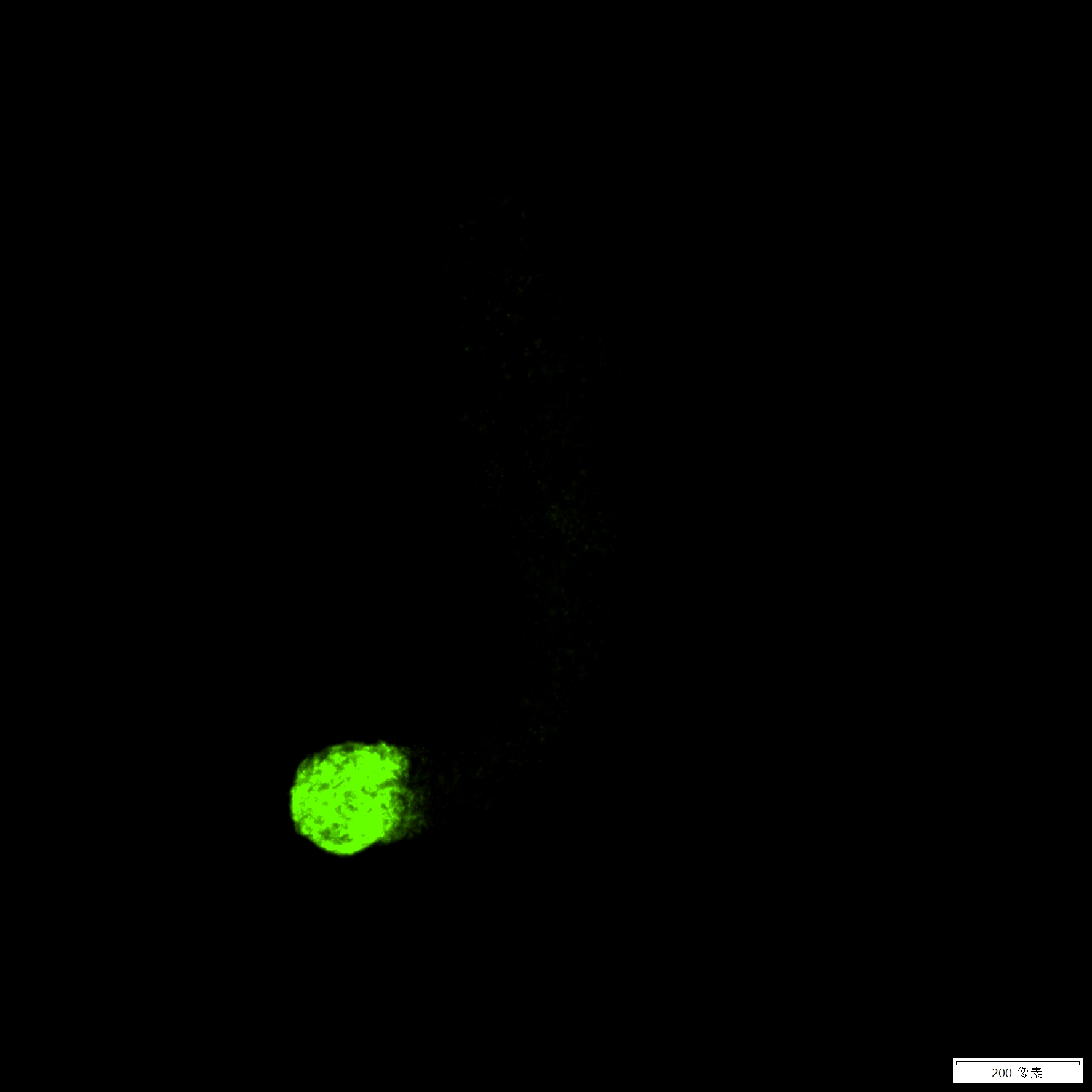

Supplement: Supplementary file 19 — Source data Fig. 3 [file 44318_2025_643_MOESM19_ESM.zip › Figure 3/3L/Bmp4 explant_24hpf_HCR_tbxta.jpg]

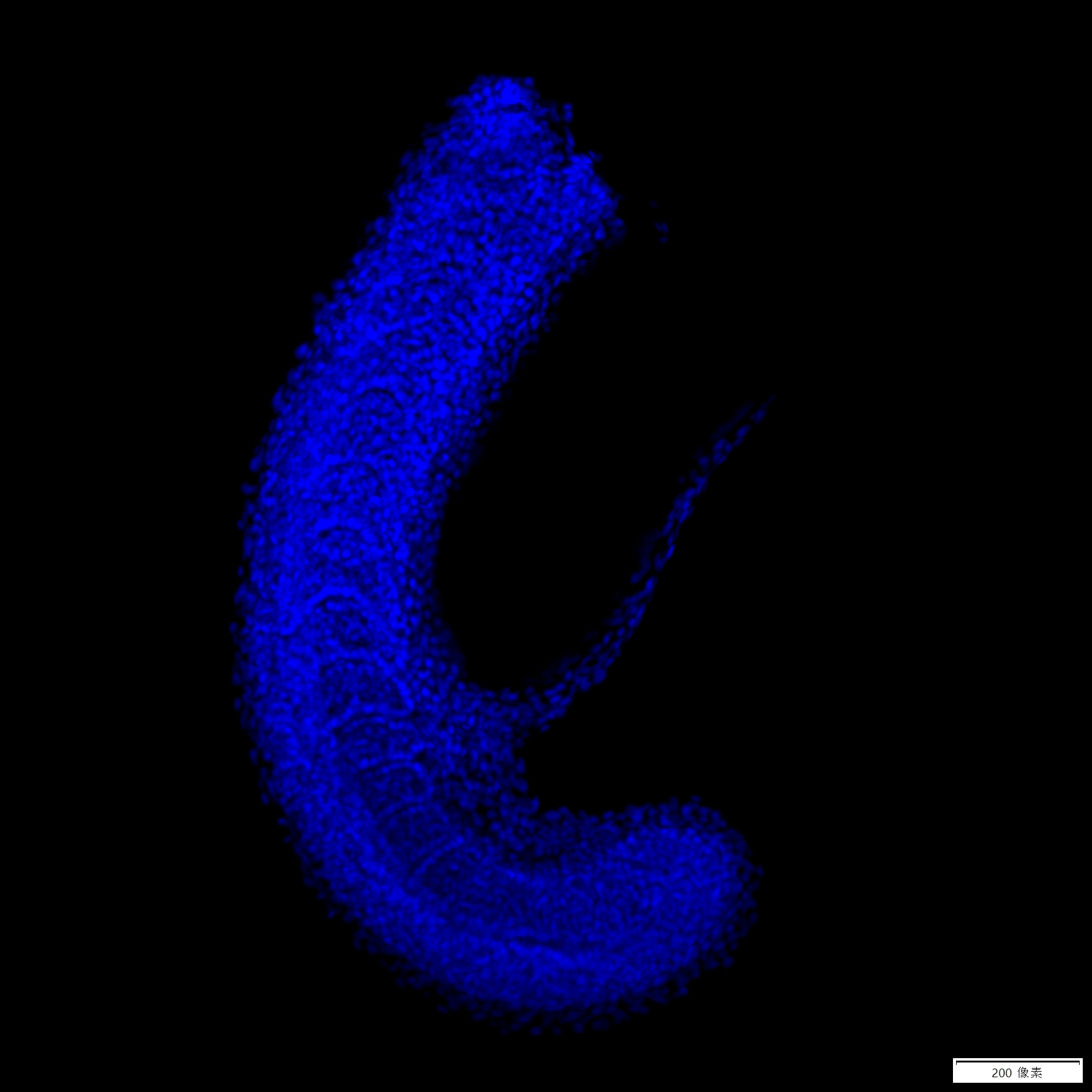

Supplement: Supplementary file 19 — Source data Fig. 3 [file 44318_2025_643_MOESM19_ESM.zip › Figure 3/3L/embryo_24hpf_DAPI.jpg]

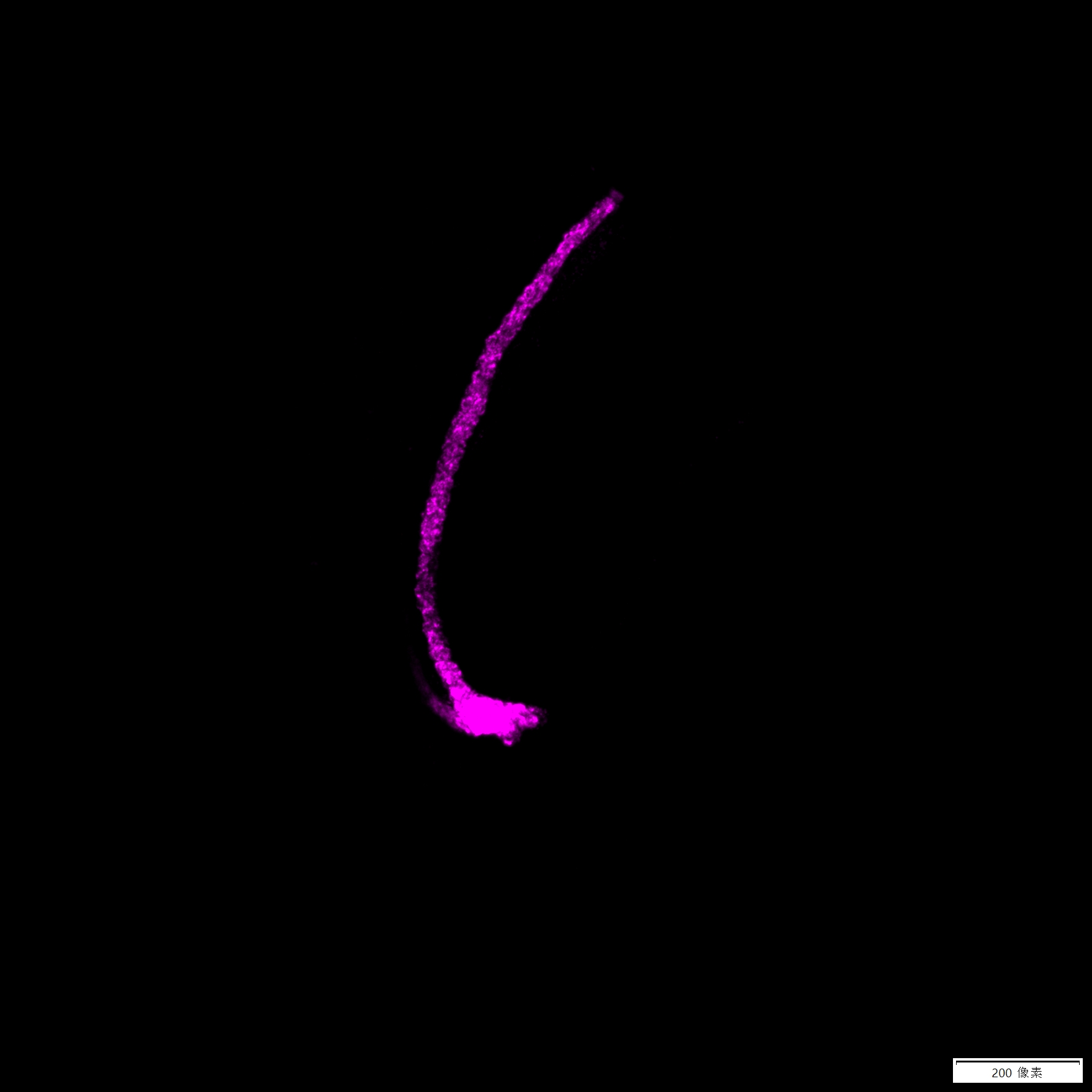

Supplement: Supplementary file 19 — Source data Fig. 3 [file 44318_2025_643_MOESM19_ESM.zip › Figure 3/3L/embryo_24hpf_HCR_cldn3d.jpg]

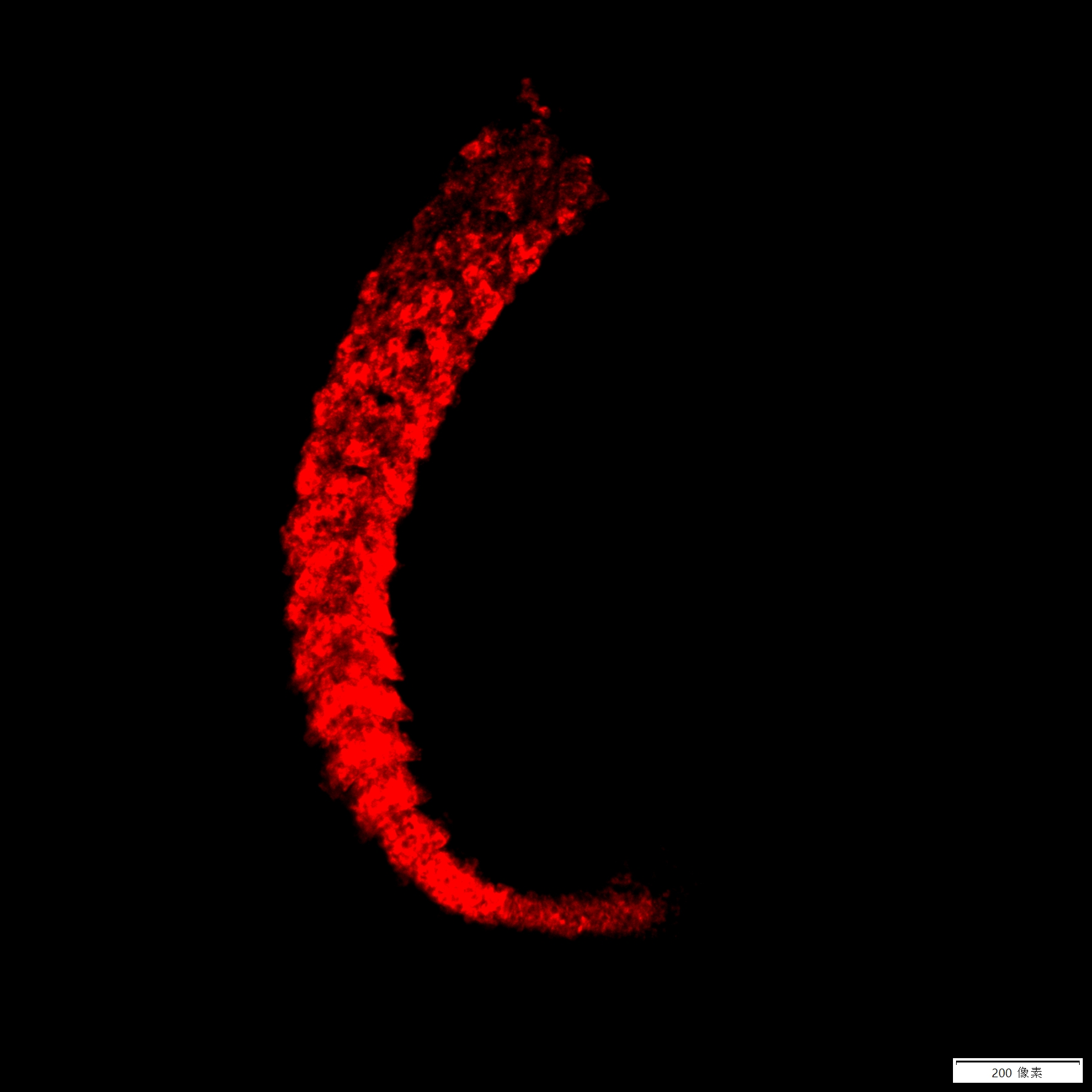

Supplement: Supplementary file 19 — Source data Fig. 3 [file 44318_2025_643_MOESM19_ESM.zip › Figure 3/3L/embryo_24hpf_HCR_myod1.jpg]

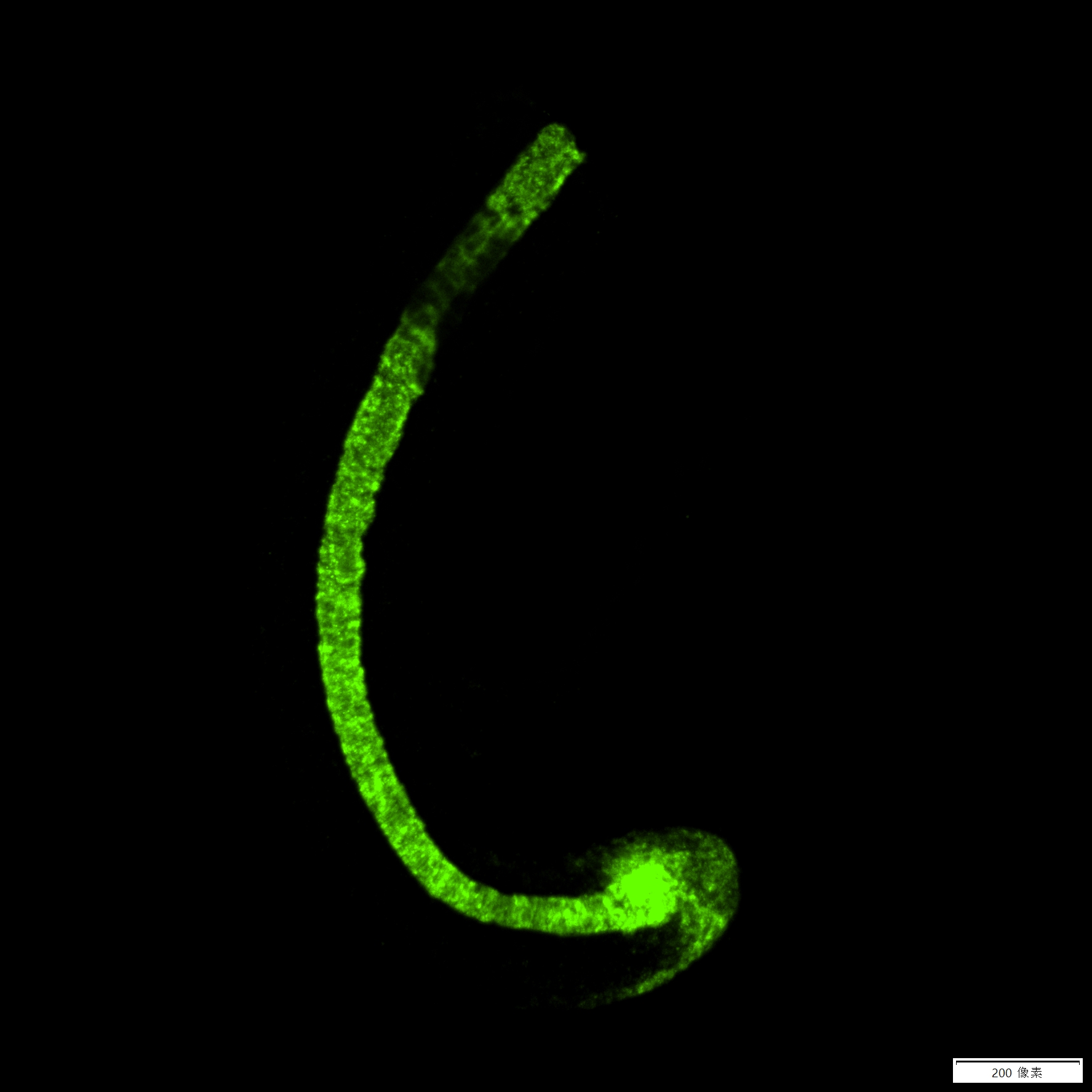

Supplement: Supplementary file 19 — Source data Fig. 3 [file 44318_2025_643_MOESM19_ESM.zip › Figure 3/3L/embryo_24hpf_HCR_tbxta.jpg]

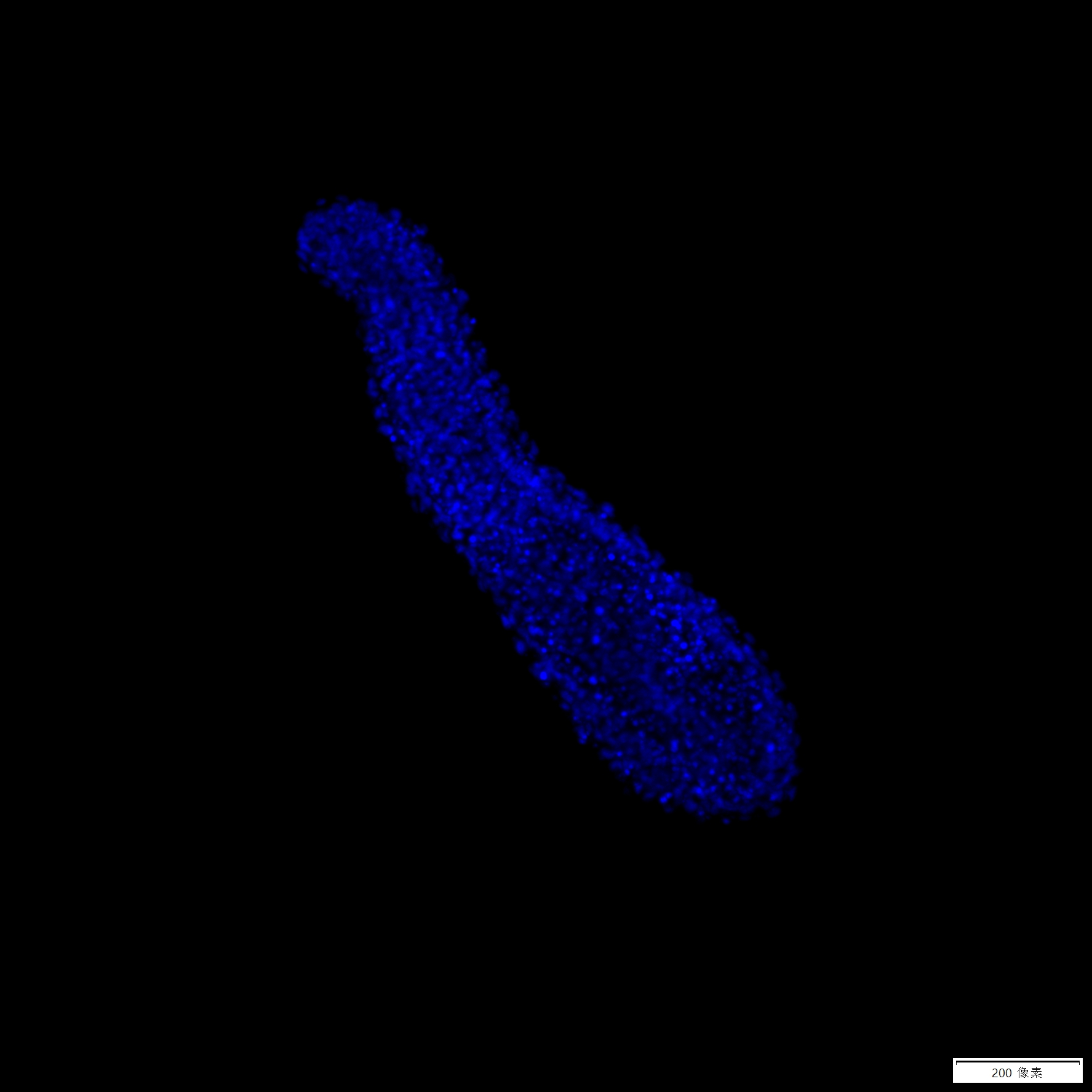

Supplement: Supplementary file 19 — Source data Fig. 3 [file 44318_2025_643_MOESM19_ESM.zip › Figure 3/3M/bmp4 explant_24hpf_DAPI.jpg]

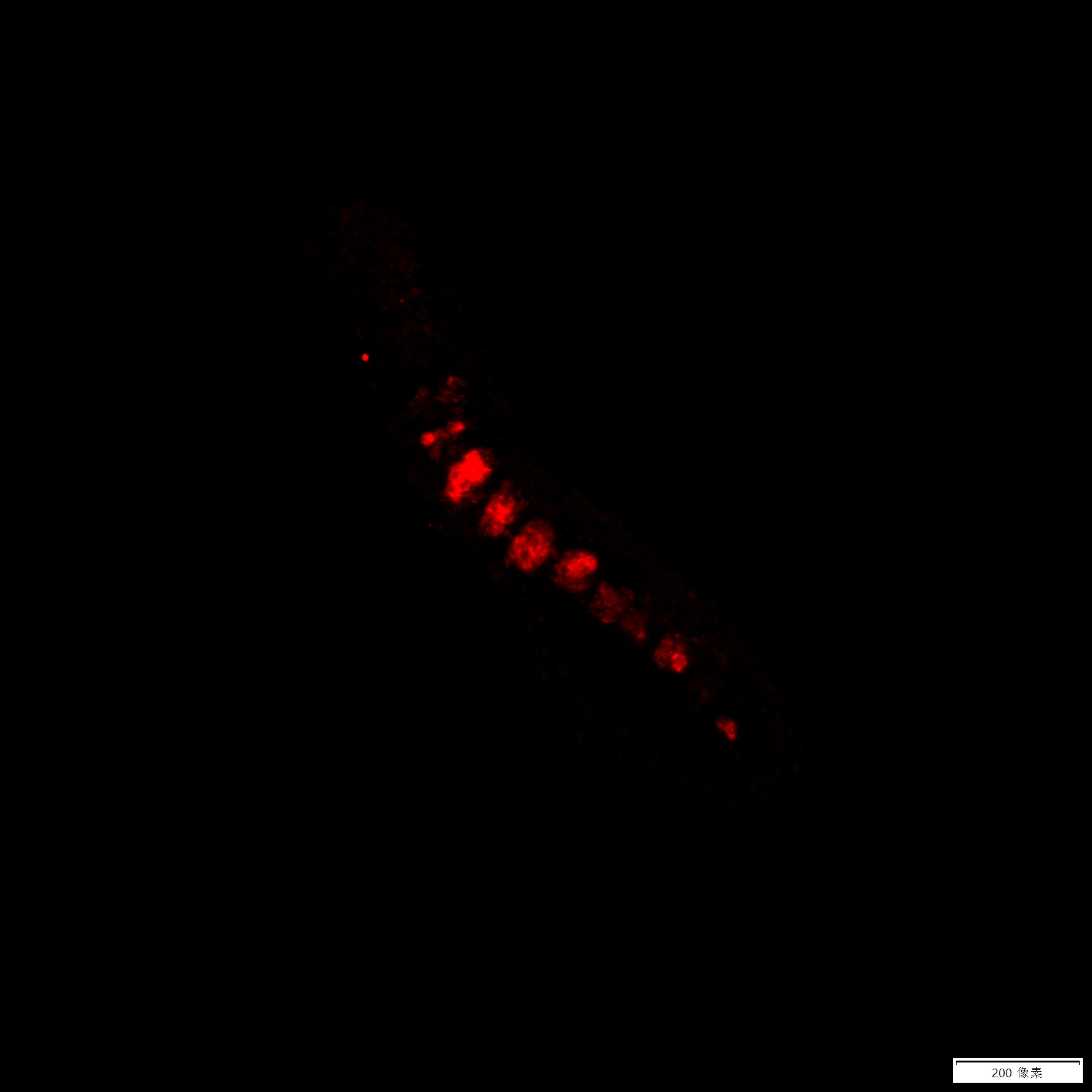

Supplement: Supplementary file 19 — Source data Fig. 3 [file 44318_2025_643_MOESM19_ESM.zip › Figure 3/3M/bmp4 explant_24hpf_HCR_myod1.jpg]

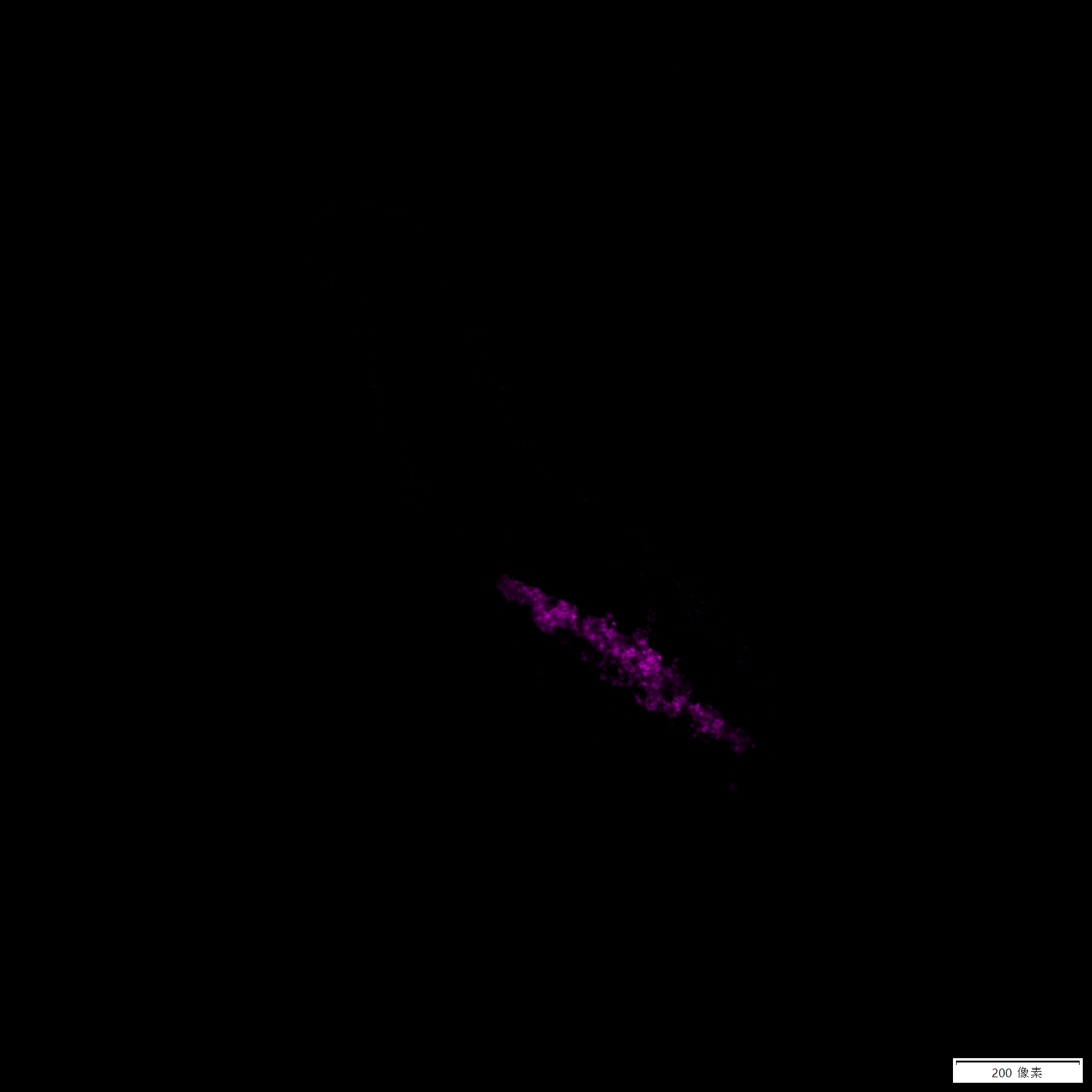

Supplement: Supplementary file 19 — Source data Fig. 3 [file 44318_2025_643_MOESM19_ESM.zip › Figure 3/3M/bmp4 explant_24hpf_HCR_sox7.jpg]

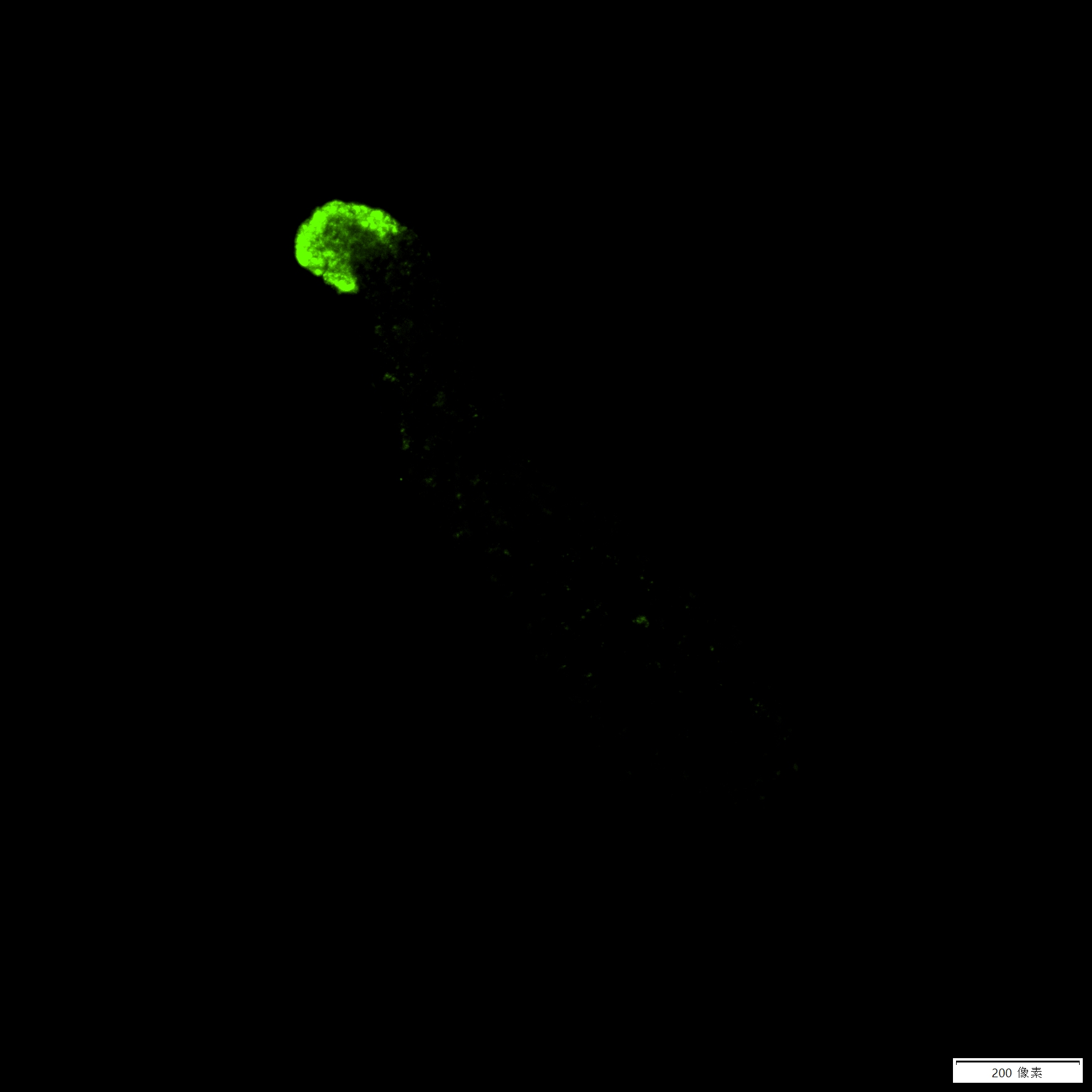

Supplement: Supplementary file 19 — Source data Fig. 3 [file 44318_2025_643_MOESM19_ESM.zip › Figure 3/3M/bmp4 explant_24hpf_HCR_tbxta.jpg]

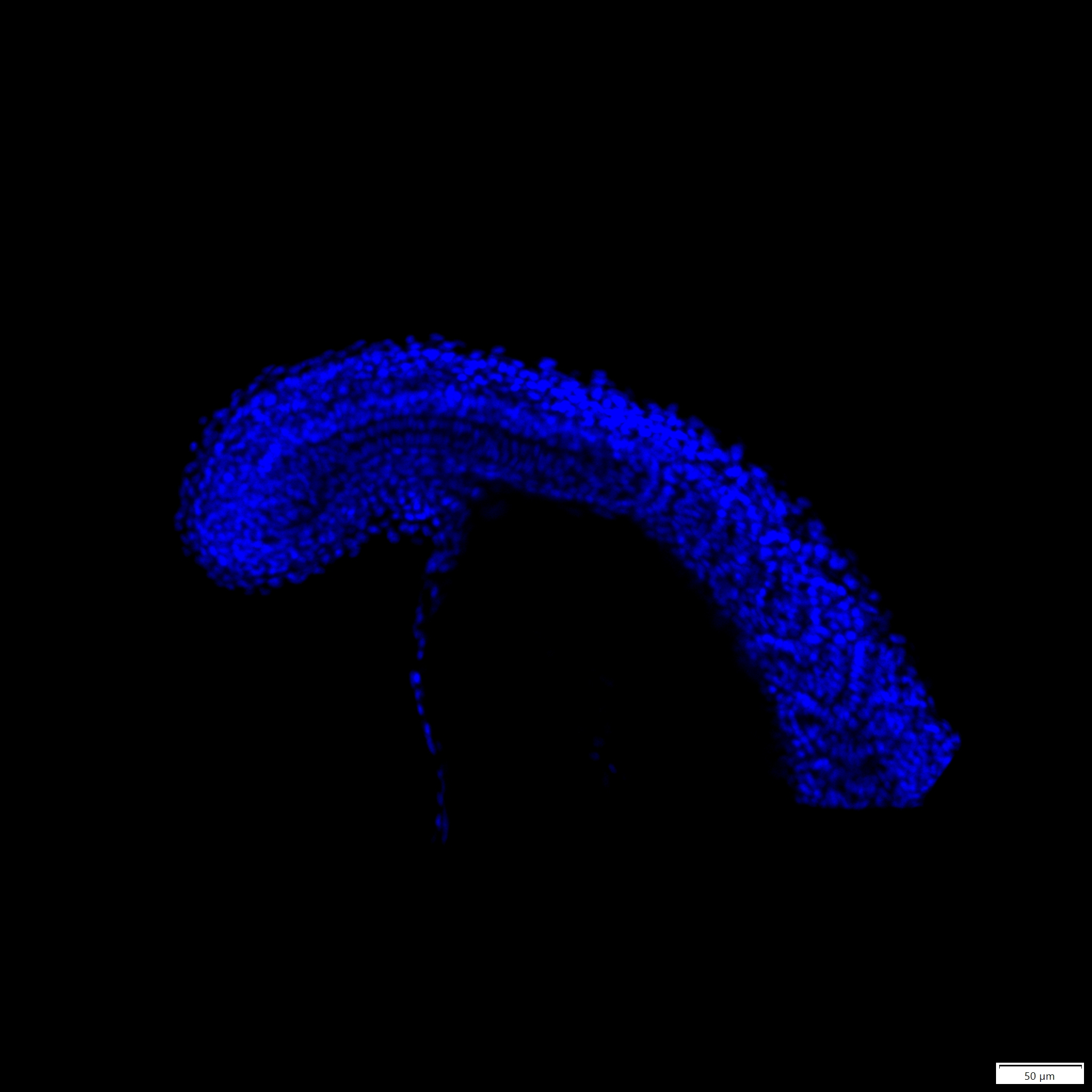

Supplement: Supplementary file 19 — Source data Fig. 3 [file 44318_2025_643_MOESM19_ESM.zip › Figure 3/3M/embryo_24hpf_DAPI.jpg]

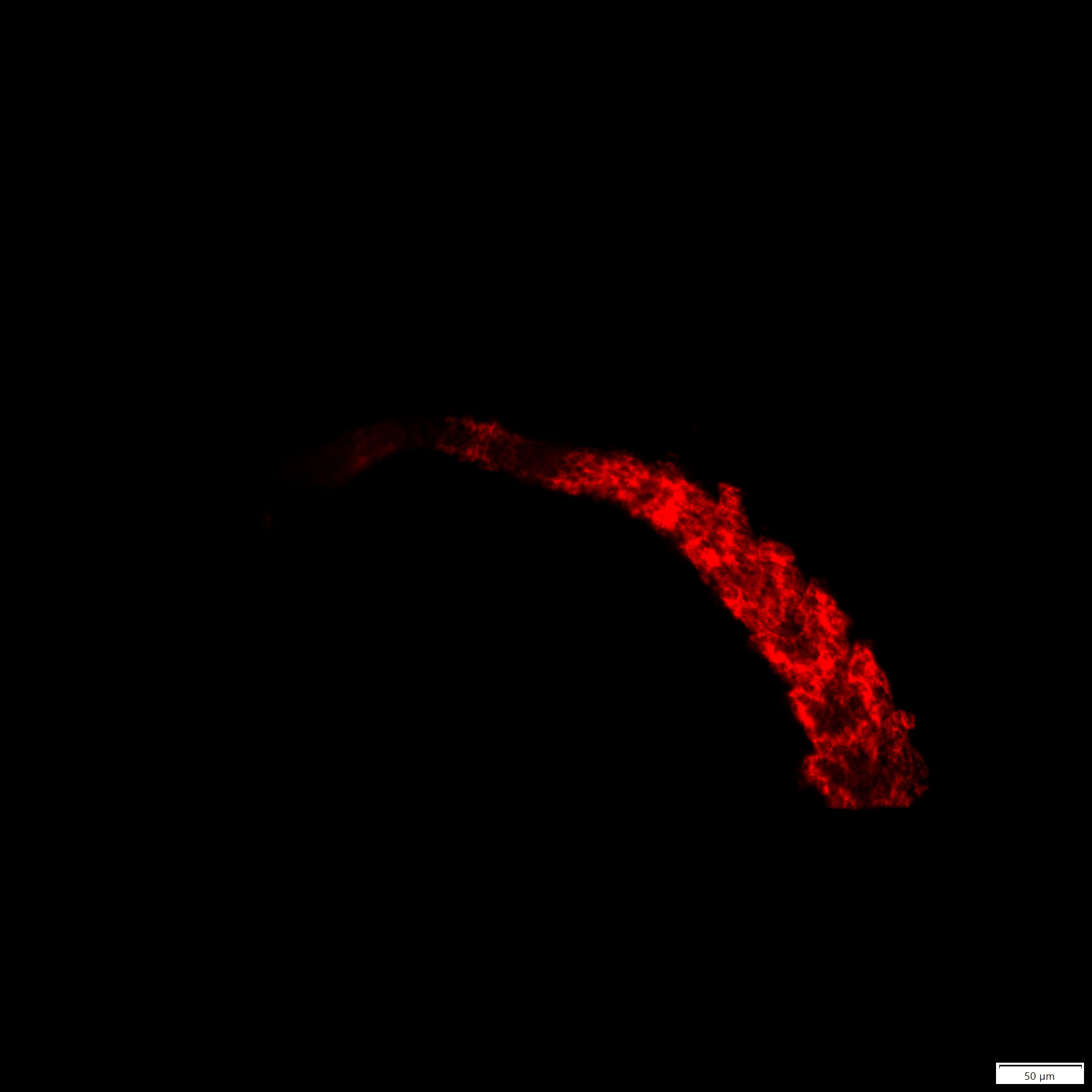

Supplement: Supplementary file 19 — Source data Fig. 3 [file 44318_2025_643_MOESM19_ESM.zip › Figure 3/3M/embryo_24hpf_HCR_myod1.jpg]

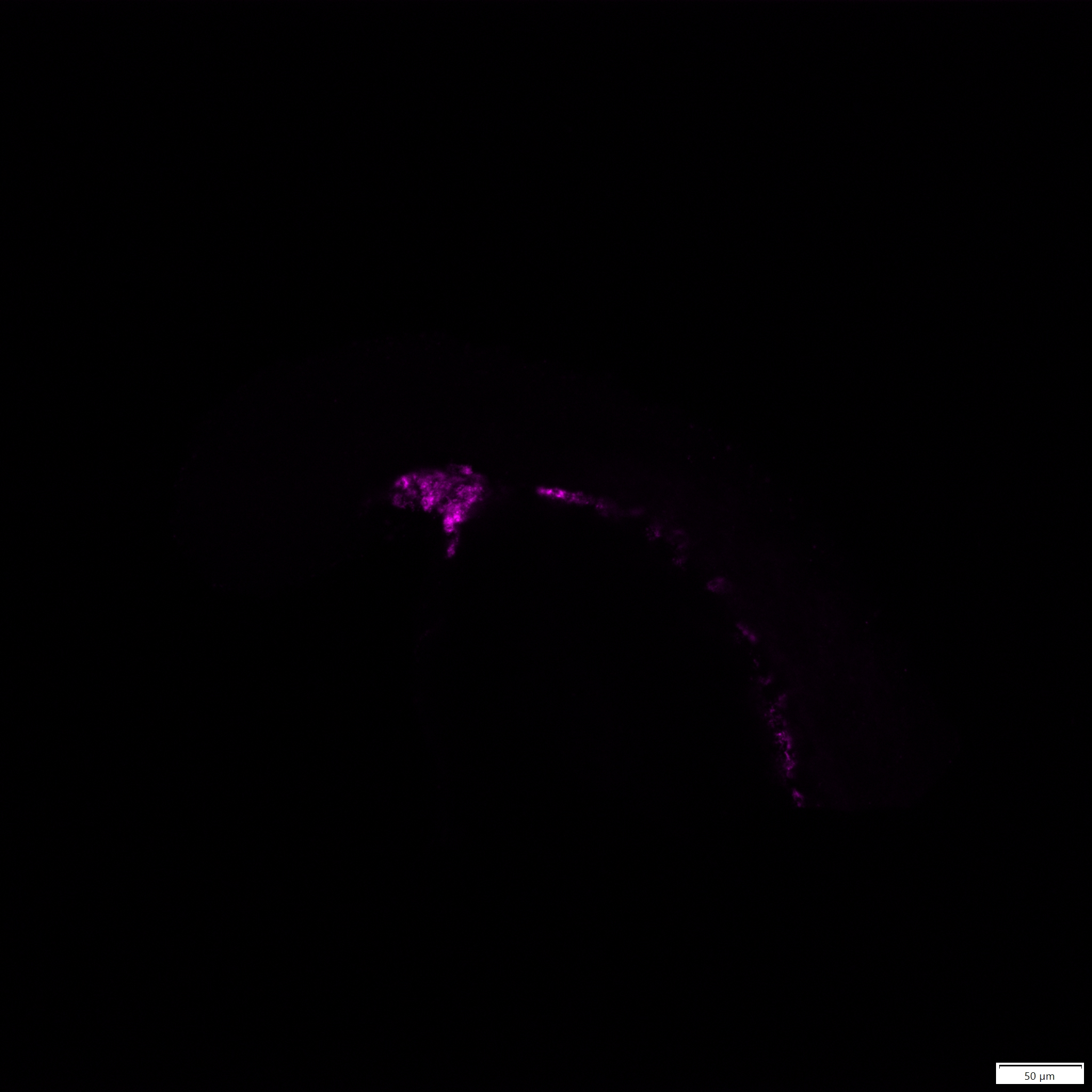

Supplement: Supplementary file 19 — Source data Fig. 3 [file 44318_2025_643_MOESM19_ESM.zip › Figure 3/3M/embryo_24hpf_HCR_sox7.jpg]

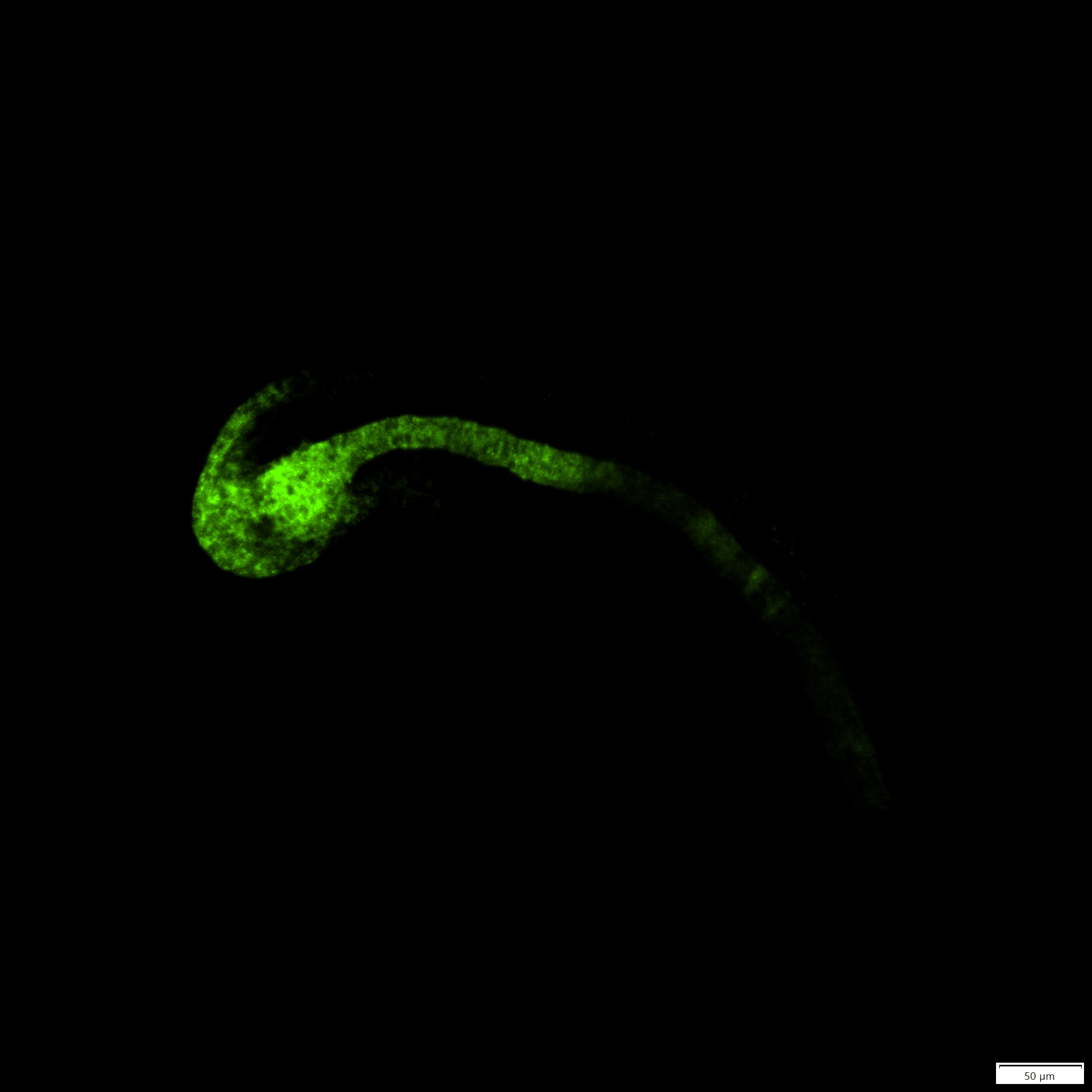

Supplement: Supplementary file 19 — Source data Fig. 3 [file 44318_2025_643_MOESM19_ESM.zip › Figure 3/3M/embryo_24hpf_HCR_tbxta.jpg]

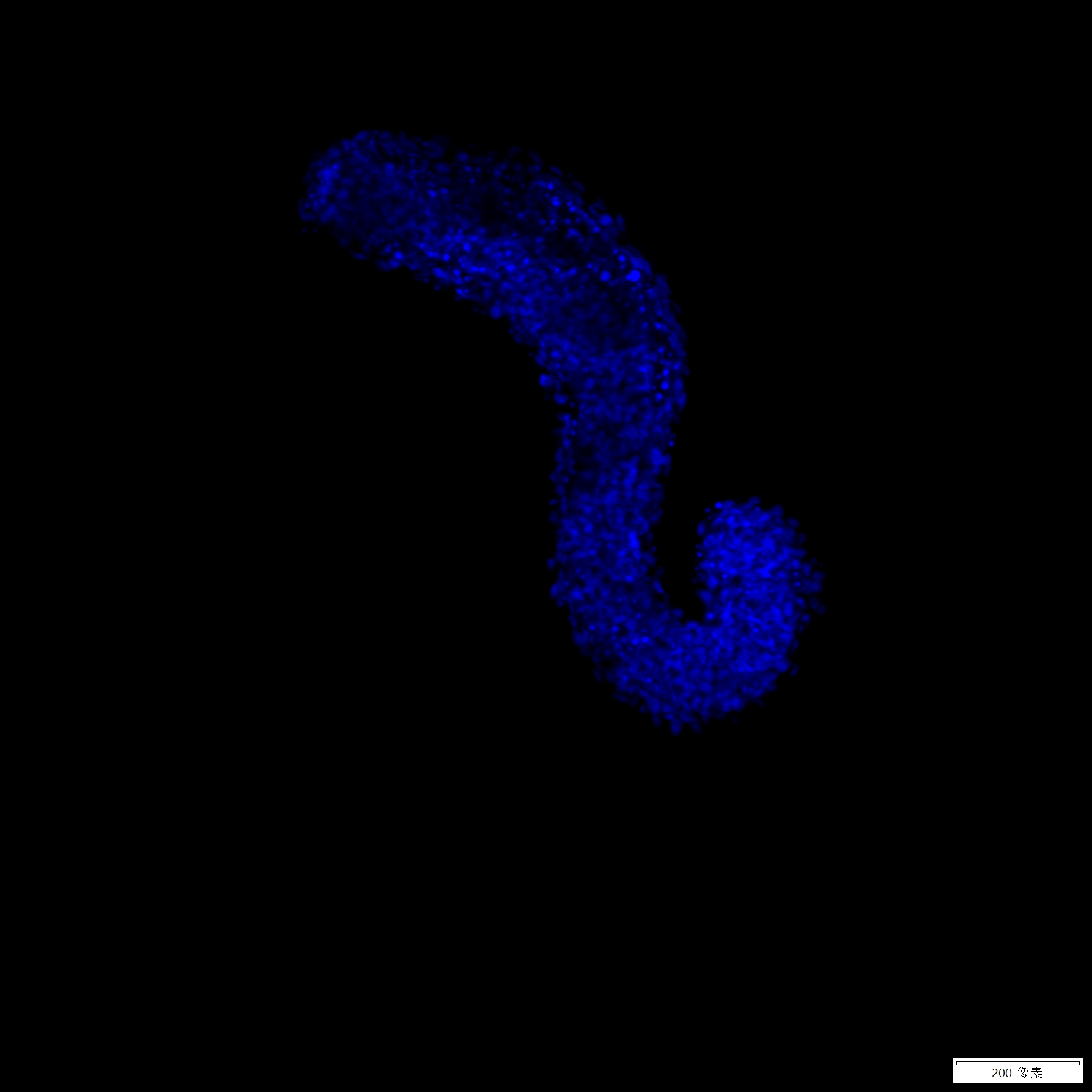

Supplement: Supplementary file 19 — Source data Fig. 3 [file 44318_2025_643_MOESM19_ESM.zip › Figure 3/3N/bmp4 explant_24hpf_DAPI.jpg]

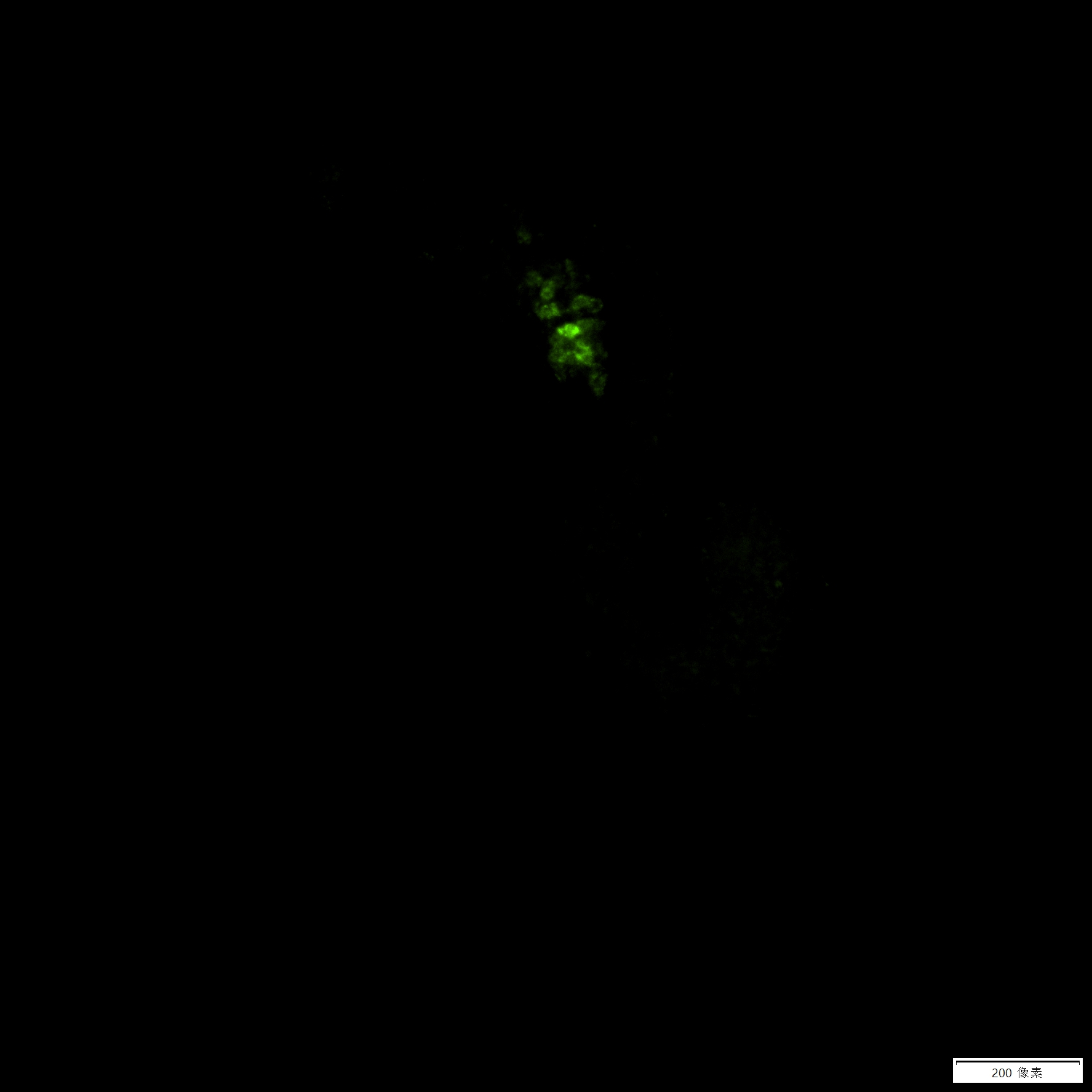

Supplement: Supplementary file 19 — Source data Fig. 3 [file 44318_2025_643_MOESM19_ESM.zip › Figure 3/3N/bmp4 explant_24hpf_HCR_hbbe1.1.jpg]

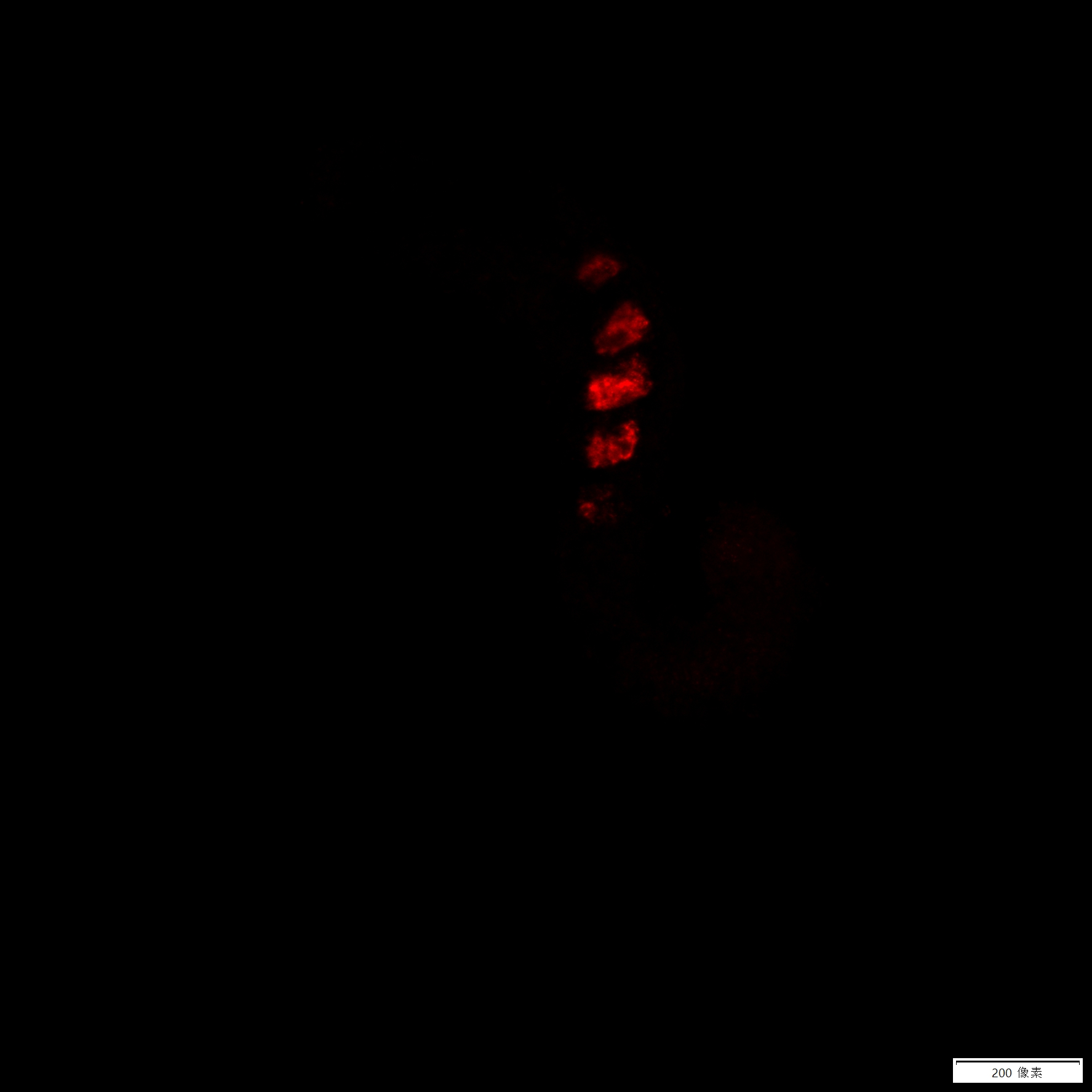

Supplement: Supplementary file 19 — Source data Fig. 3 [file 44318_2025_643_MOESM19_ESM.zip › Figure 3/3N/bmp4 explant_24hpf_HCR_myod1.jpg]

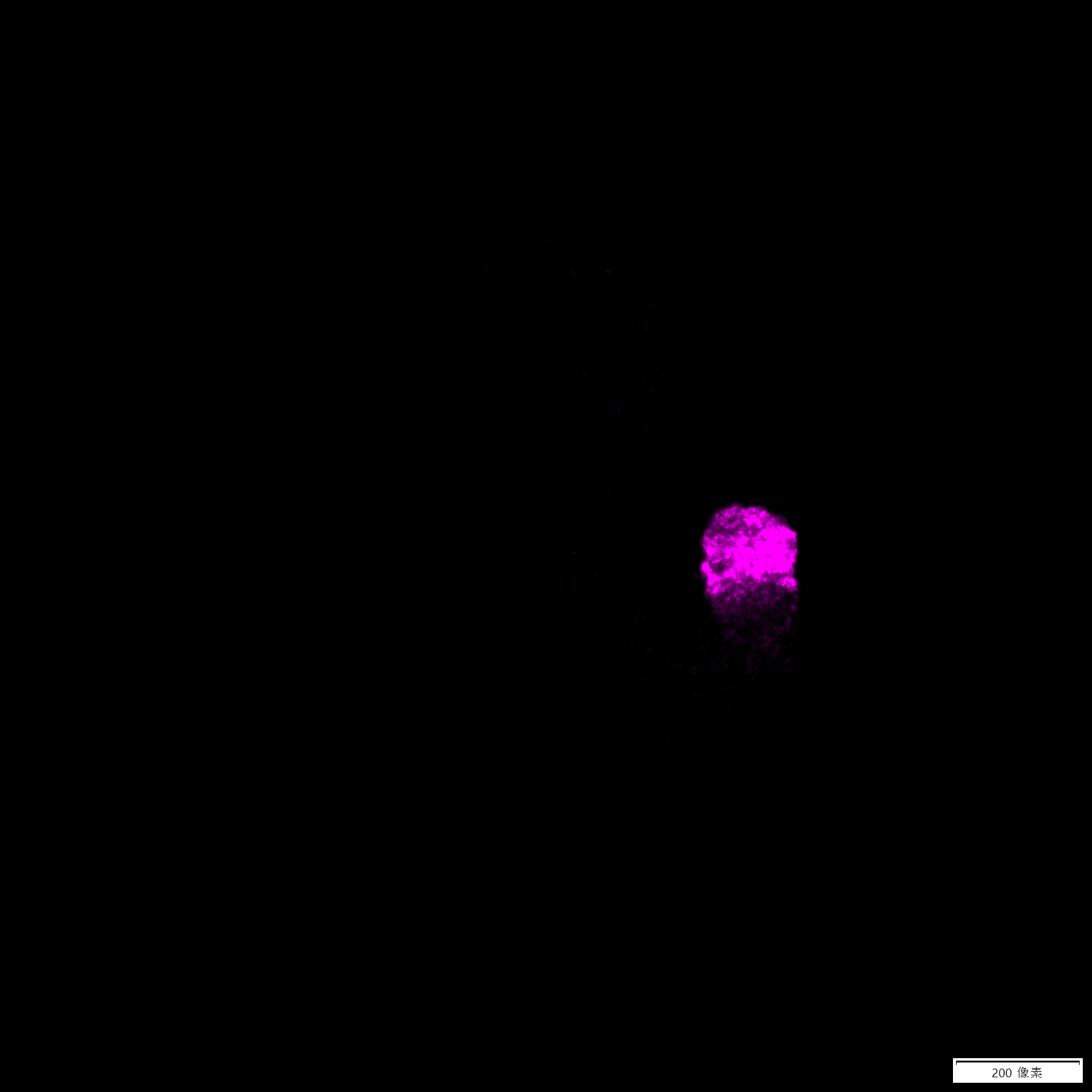

Supplement: Supplementary file 19 — Source data Fig. 3 [file 44318_2025_643_MOESM19_ESM.zip › Figure 3/3N/bmp4 explant_24hpf_HCR_tbxta.jpg]

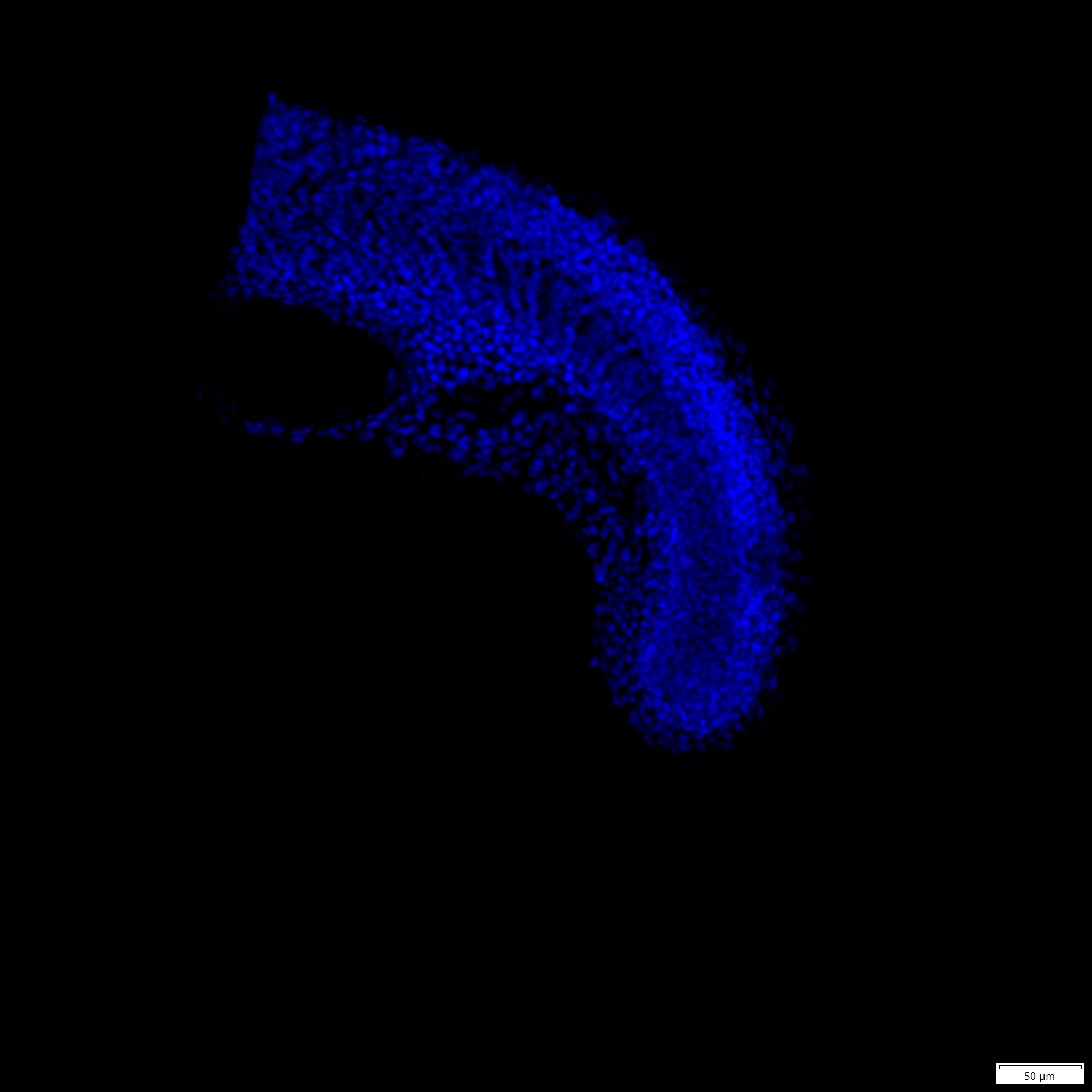

Supplement: Supplementary file 19 — Source data Fig. 3 [file 44318_2025_643_MOESM19_ESM.zip › Figure 3/3N/embryo_24hpf_DAPI.jpg]

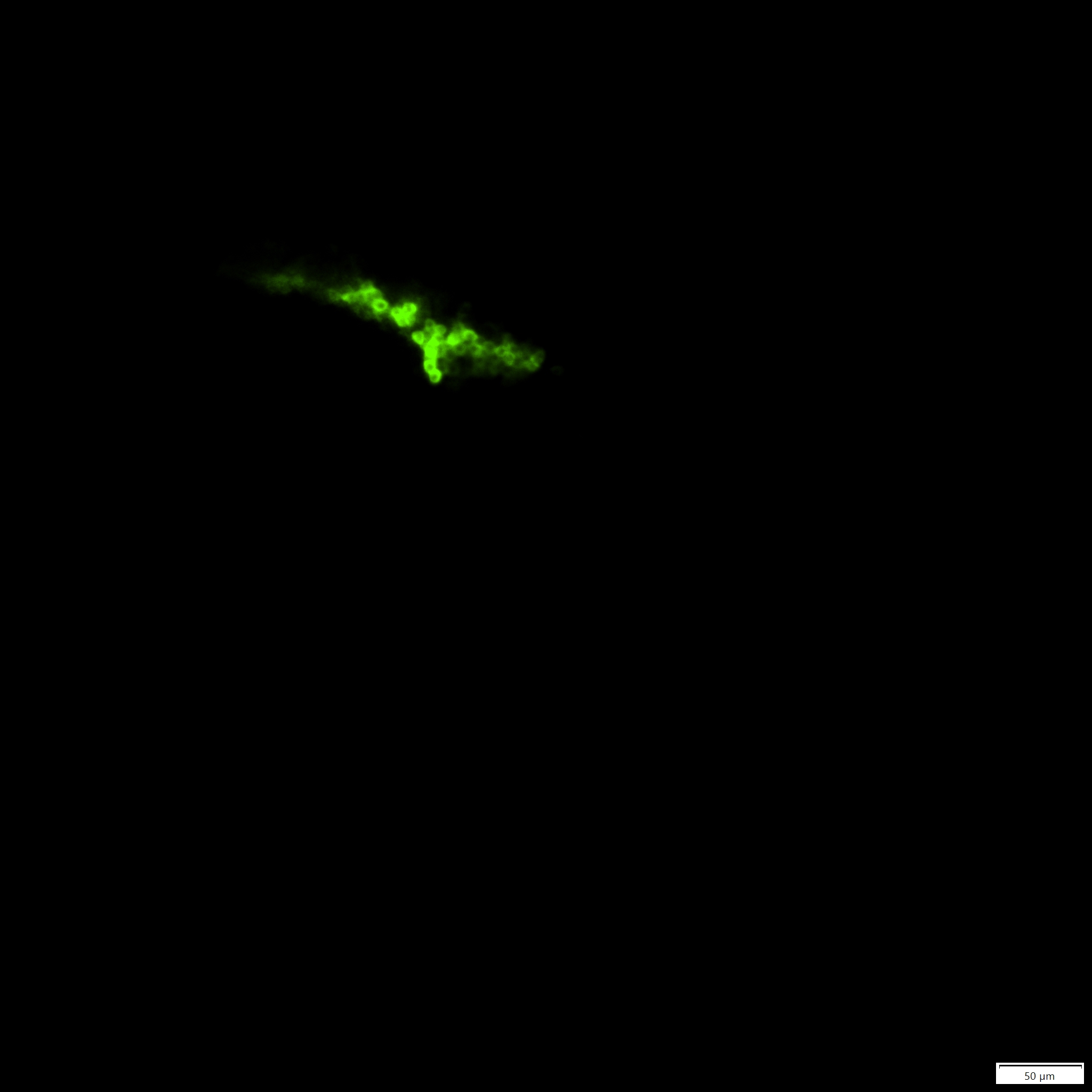

Supplement: Supplementary file 19 — Source data Fig. 3 [file 44318_2025_643_MOESM19_ESM.zip › Figure 3/3N/embryo_24hpf_HCR_hbbe1.1.jpg]

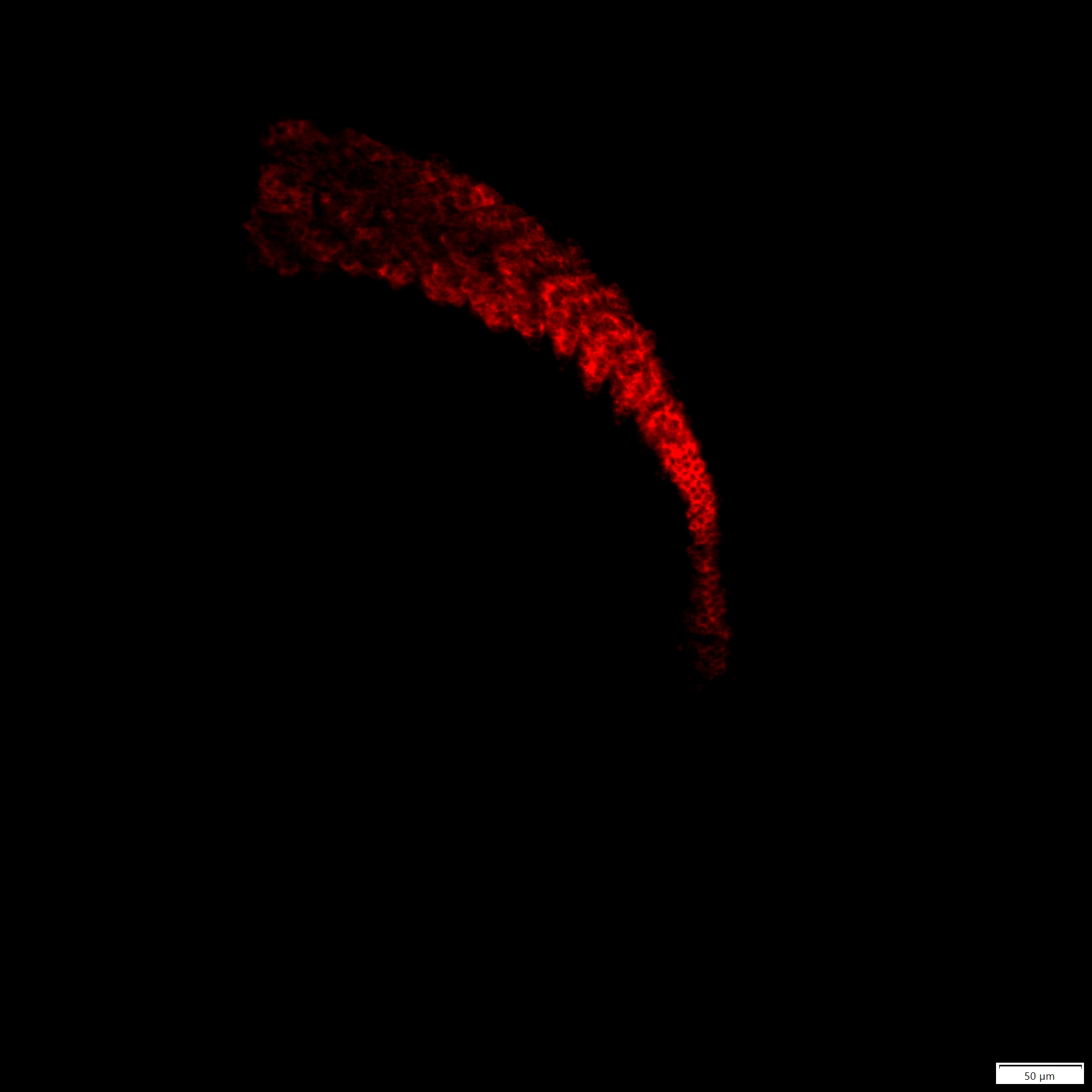

Supplement: Supplementary file 19 — Source data Fig. 3 [file 44318_2025_643_MOESM19_ESM.zip › Figure 3/3N/embryo_24hpf_HCR_myod1.jpg]

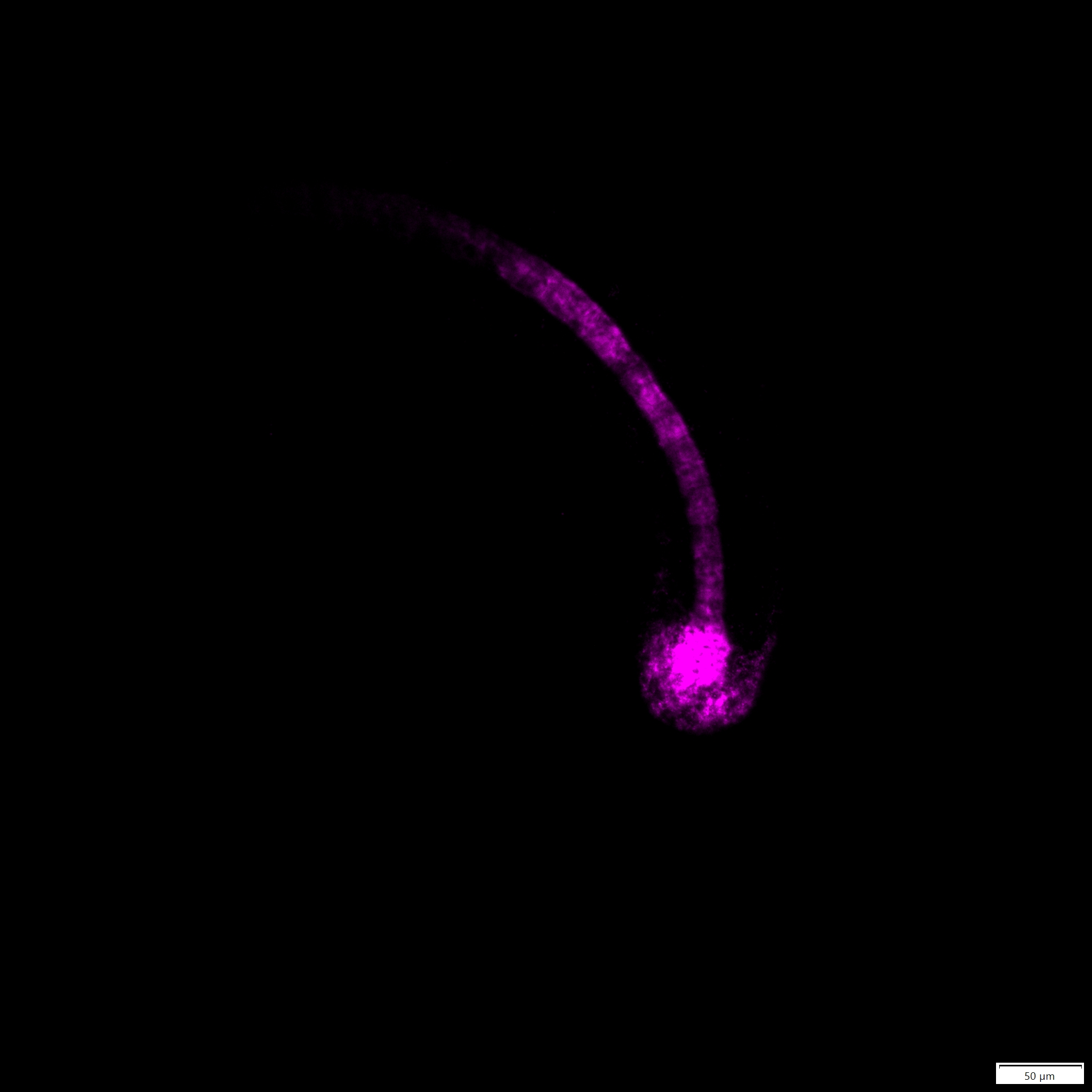

Supplement: Supplementary file 19 — Source data Fig. 3 [file 44318_2025_643_MOESM19_ESM.zip › Figure 3/3N/embryo_24hpf_HCR_tbxta.jpg]

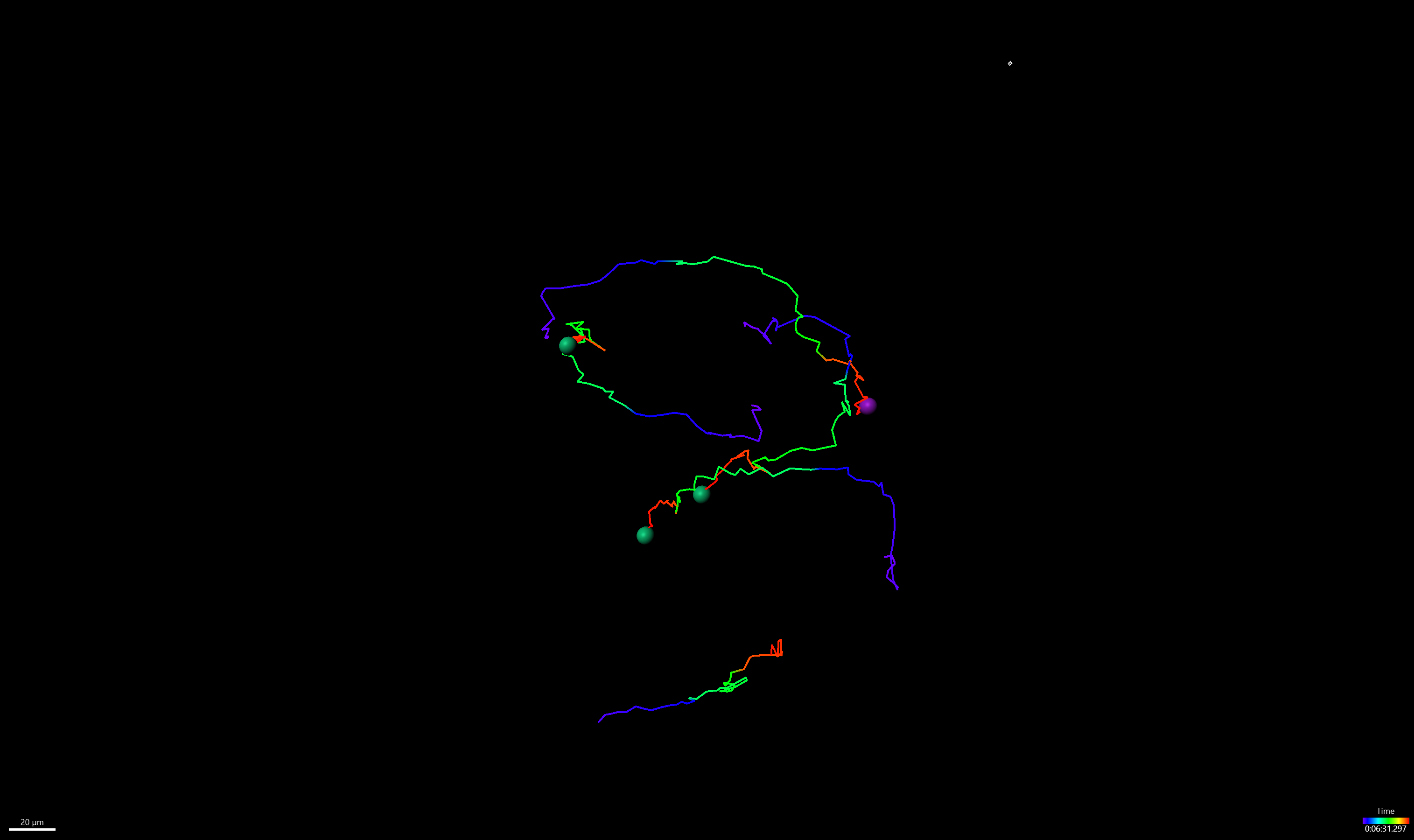

Supplement: Supplementary file 20 — Source data Fig. 4 [file 44318_2025_643_MOESM20_ESM.zip › Figure 4/4B/bottome_time 6.tif]

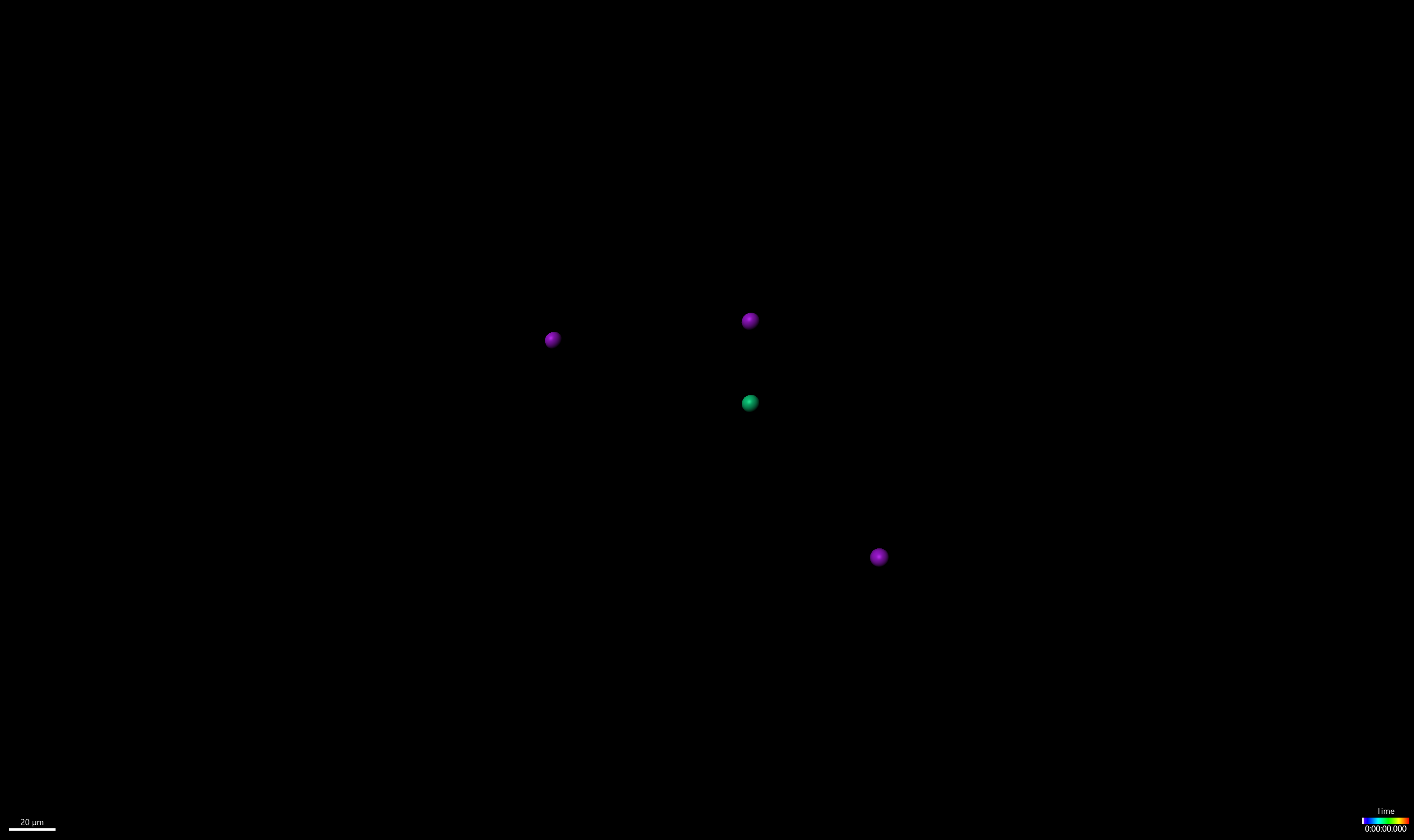

Supplement: Supplementary file 20 — Source data Fig. 4 [file 44318_2025_643_MOESM20_ESM.zip › Figure 4/4B/bottom_time 1.tif]

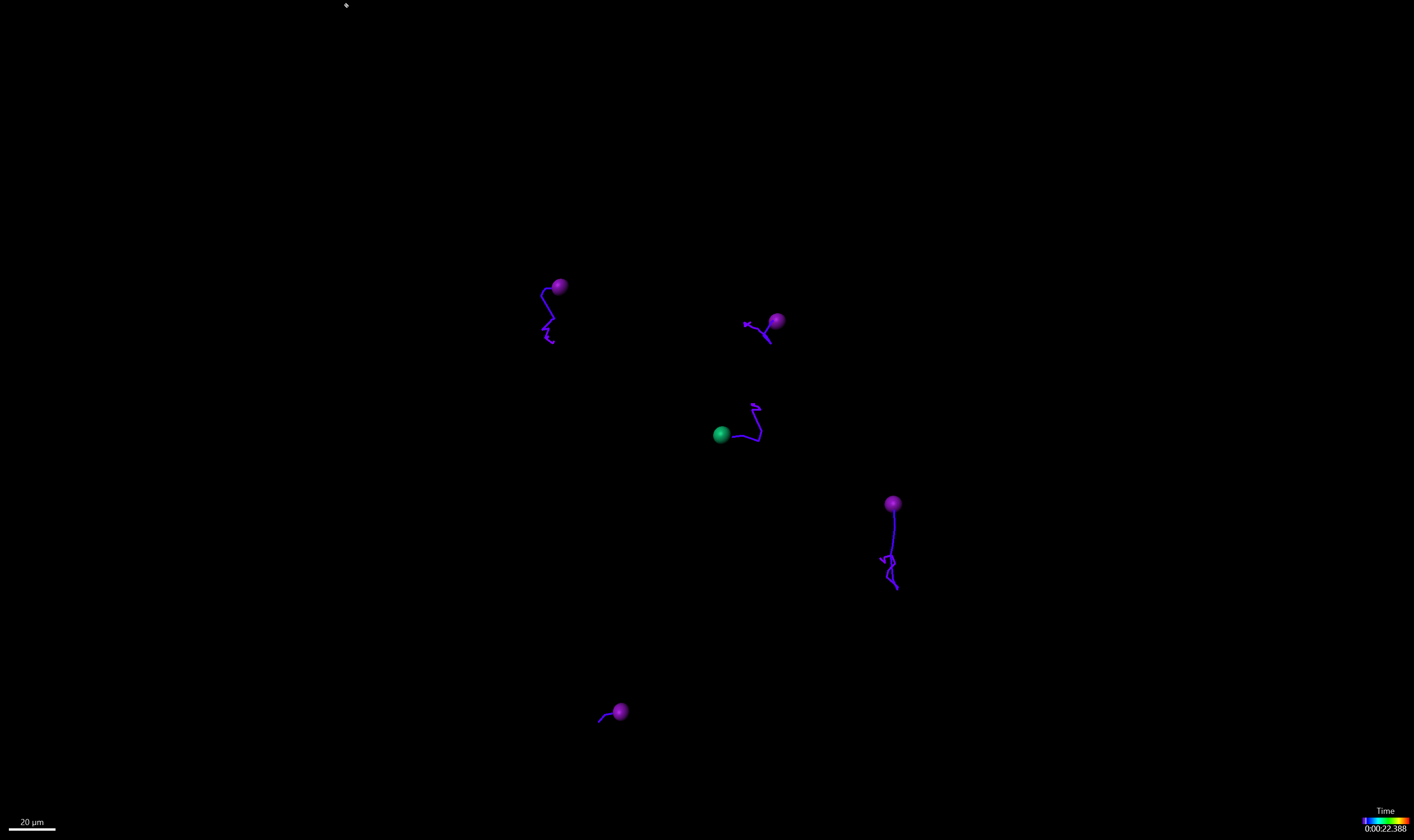

Supplement: Supplementary file 20 — Source data Fig. 4 [file 44318_2025_643_MOESM20_ESM.zip › Figure 4/4B/bottom_time 2.tif]

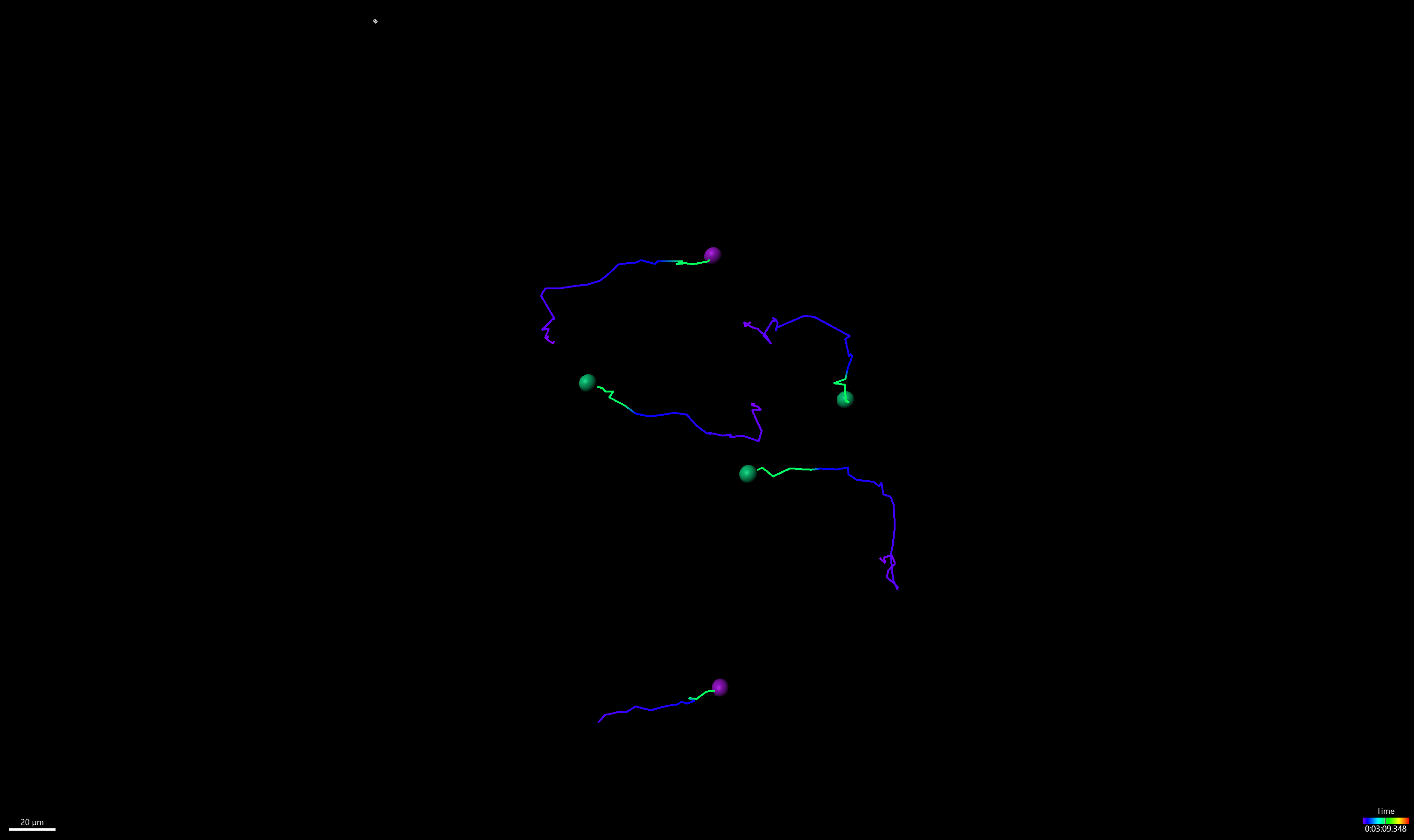

Supplement: Supplementary file 20 — Source data Fig. 4 [file 44318_2025_643_MOESM20_ESM.zip › Figure 4/4B/bottom_time 3.tif]

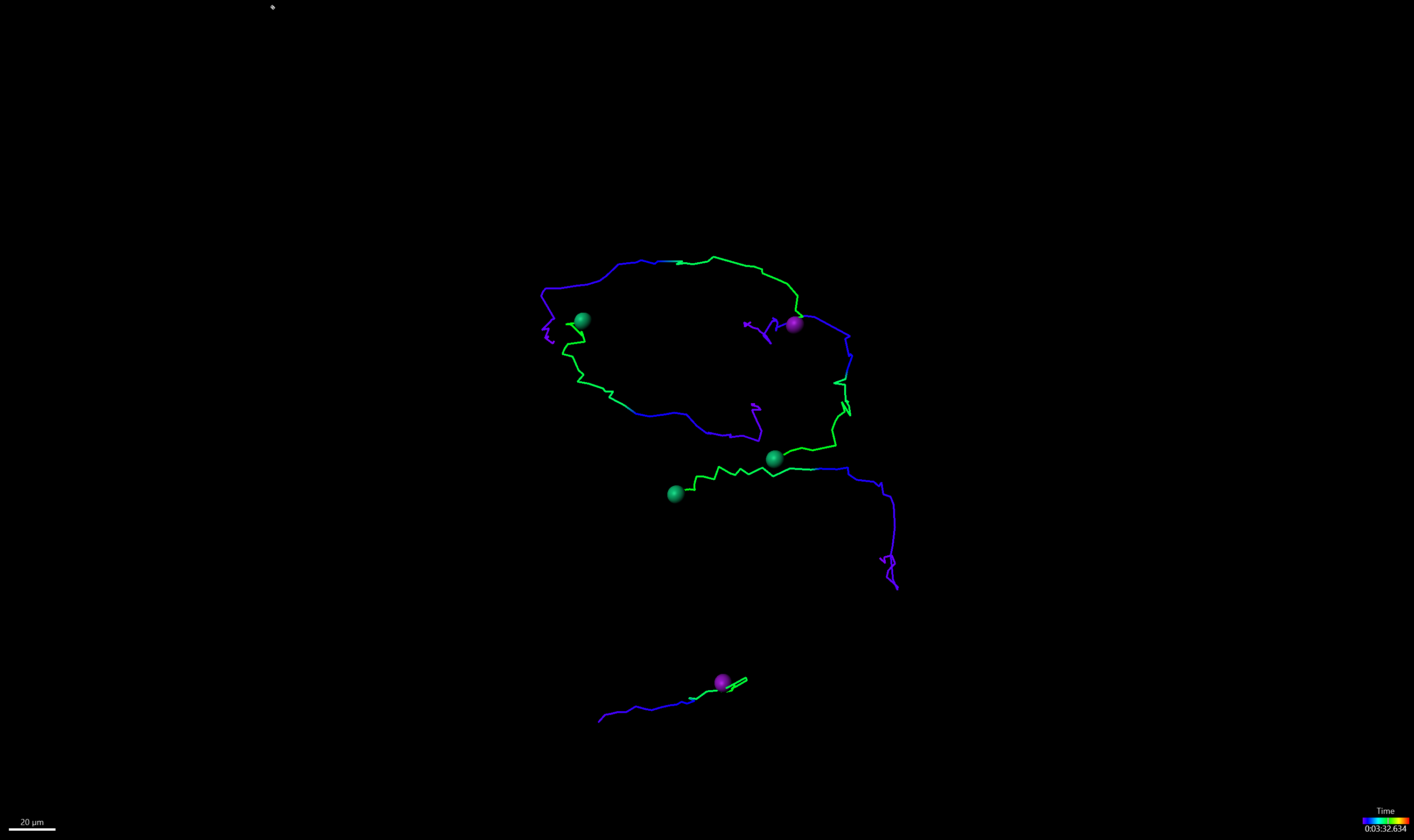

Supplement: Supplementary file 20 — Source data Fig. 4 [file 44318_2025_643_MOESM20_ESM.zip › Figure 4/4B/bottom_time 4.tif]

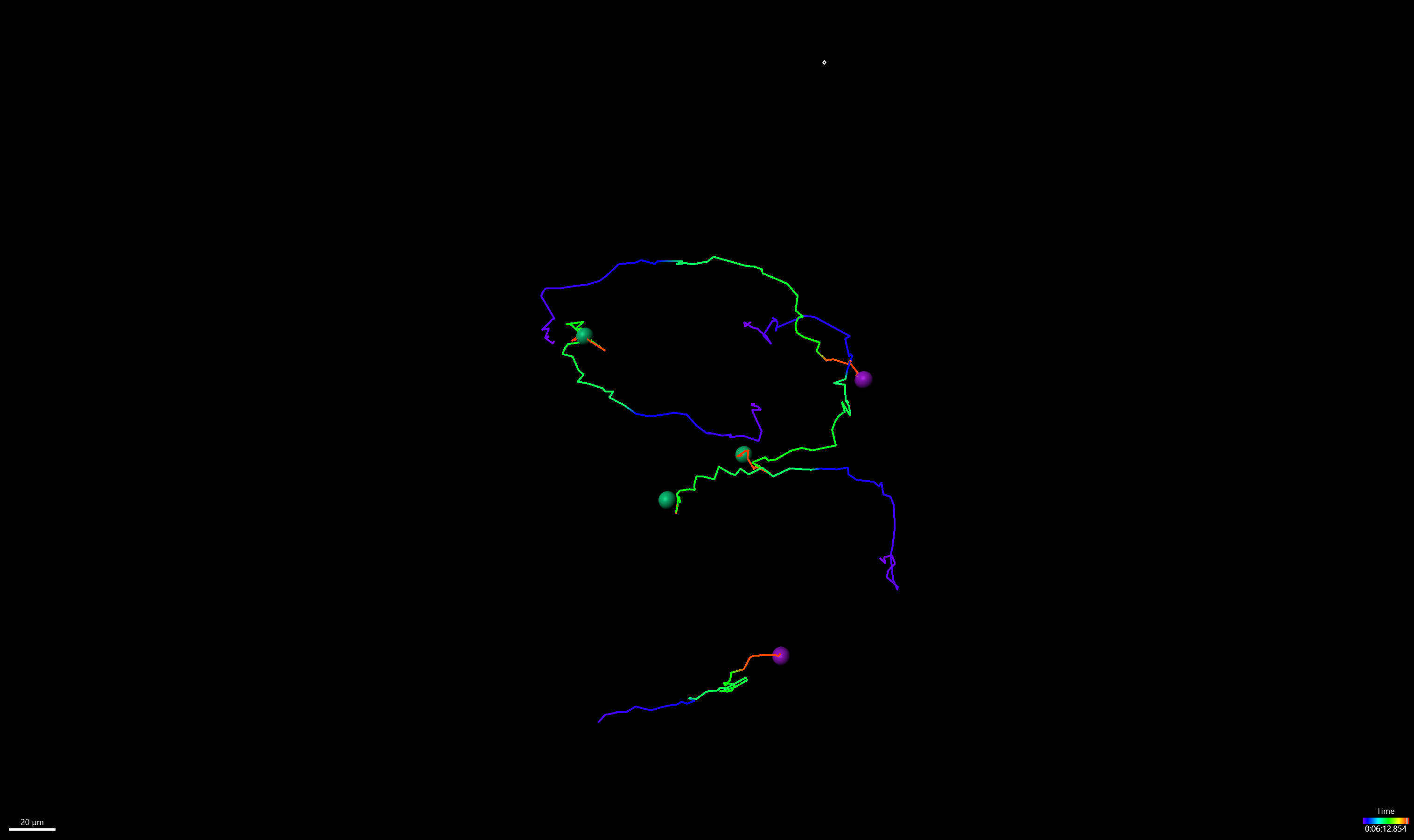

Supplement: Supplementary file 20 — Source data Fig. 4 [file 44318_2025_643_MOESM20_ESM.zip › Figure 4/4B/bottom_time 5.tif]

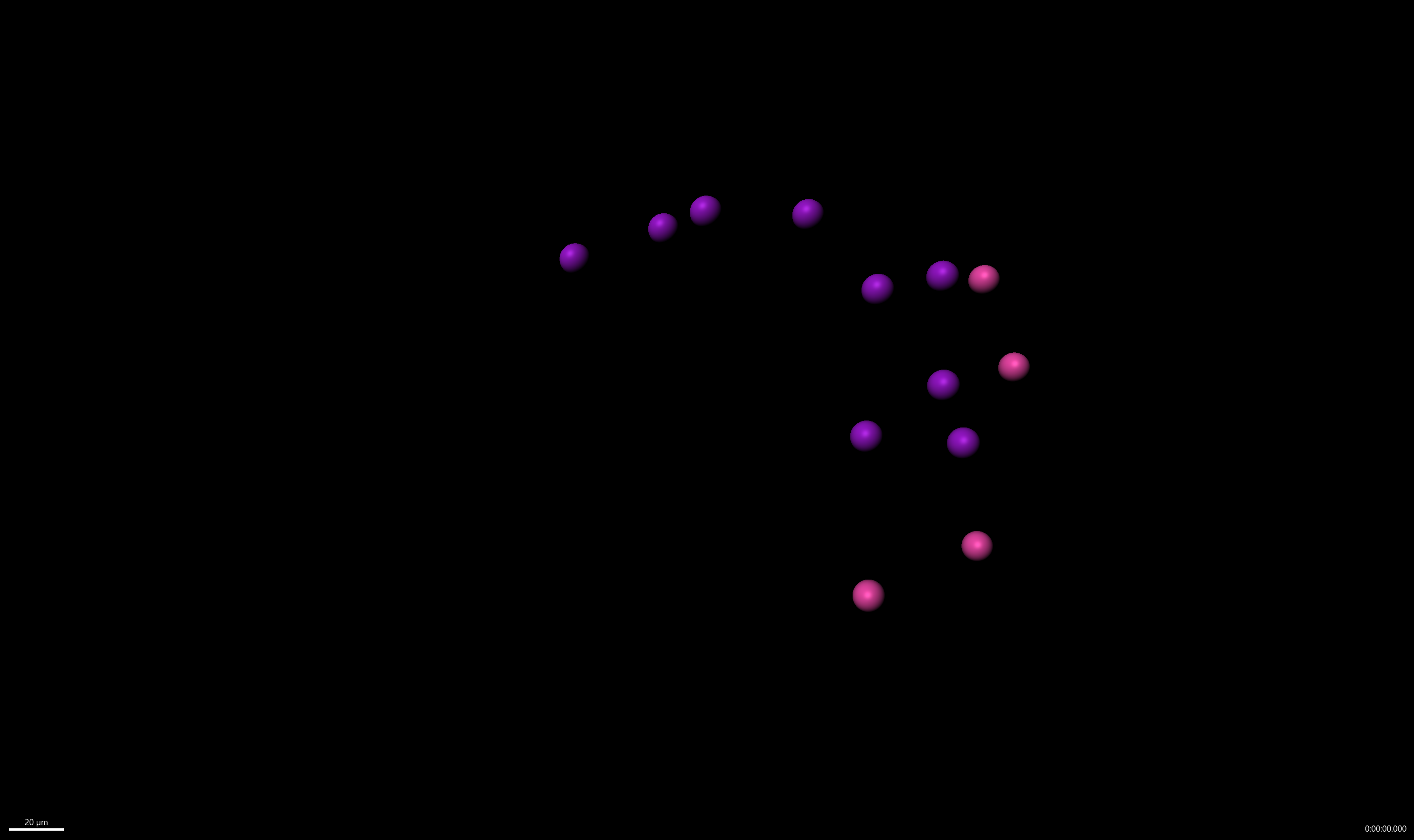

Supplement: Supplementary file 20 — Source data Fig. 4 [file 44318_2025_643_MOESM20_ESM.zip › Figure 4/4B/middle_time 1.tif]

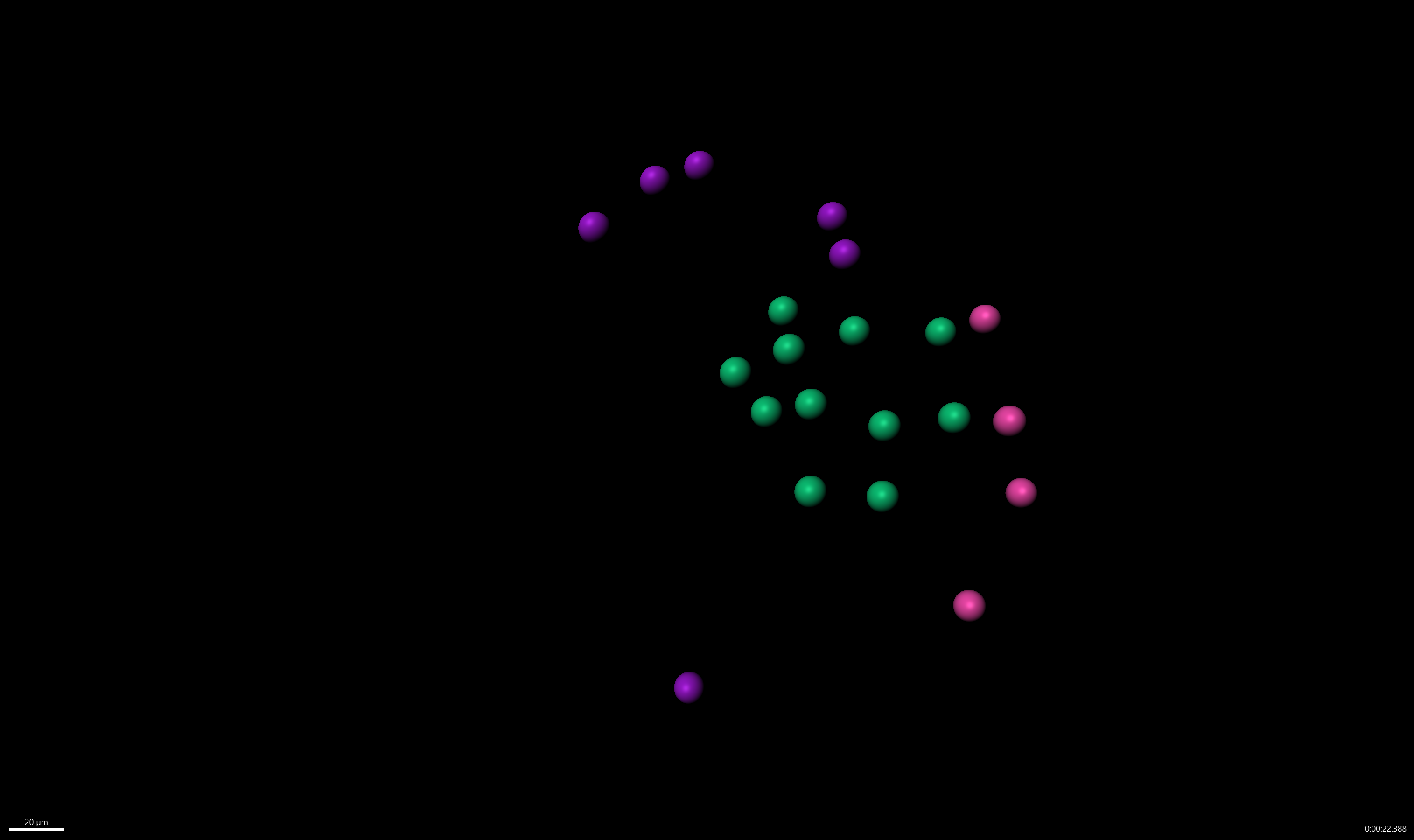

Supplement: Supplementary file 20 — Source data Fig. 4 [file 44318_2025_643_MOESM20_ESM.zip › Figure 4/4B/middle_time 2.tif]

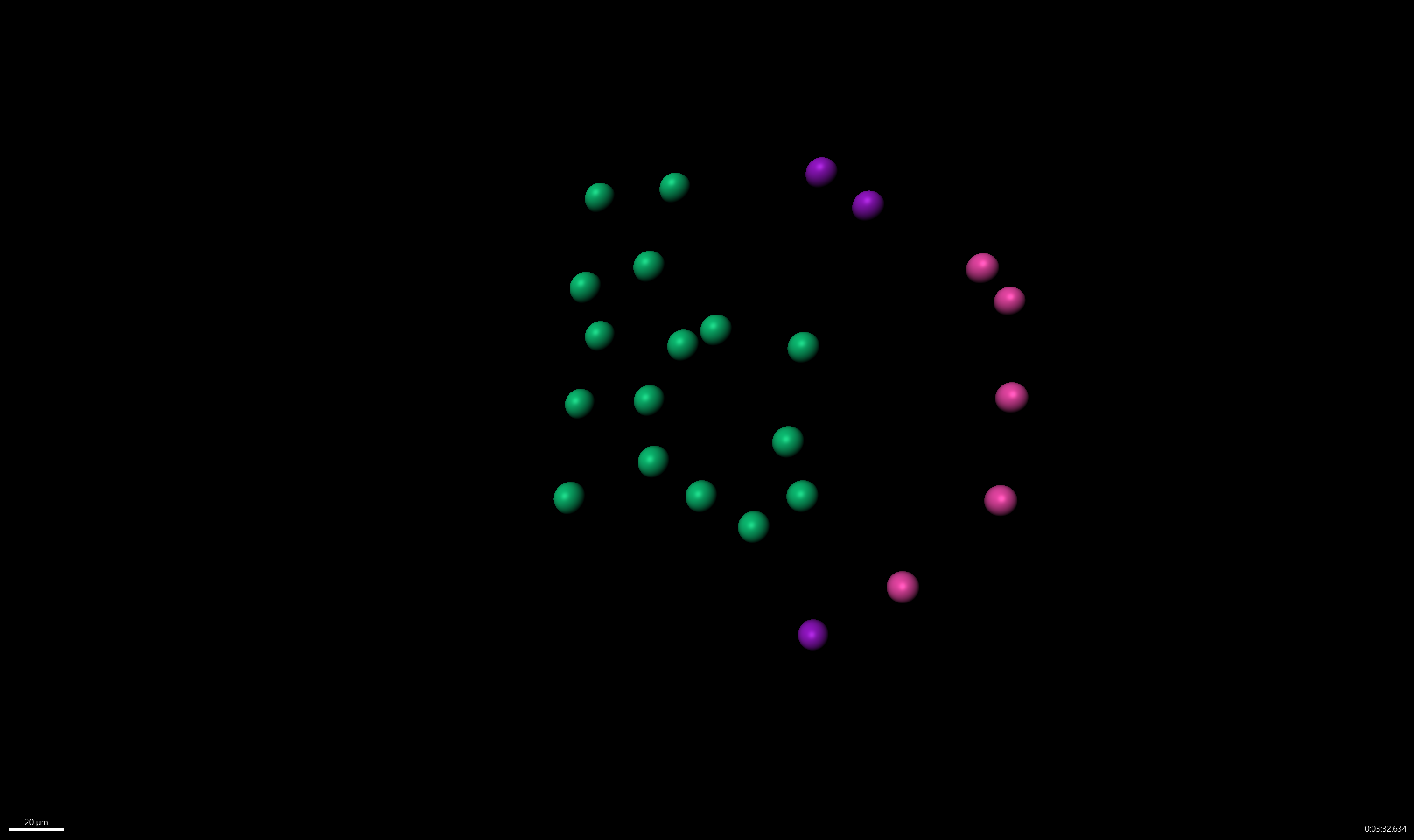

Supplement: Supplementary file 20 — Source data Fig. 4 [file 44318_2025_643_MOESM20_ESM.zip › Figure 4/4B/middle_time 4.tif]

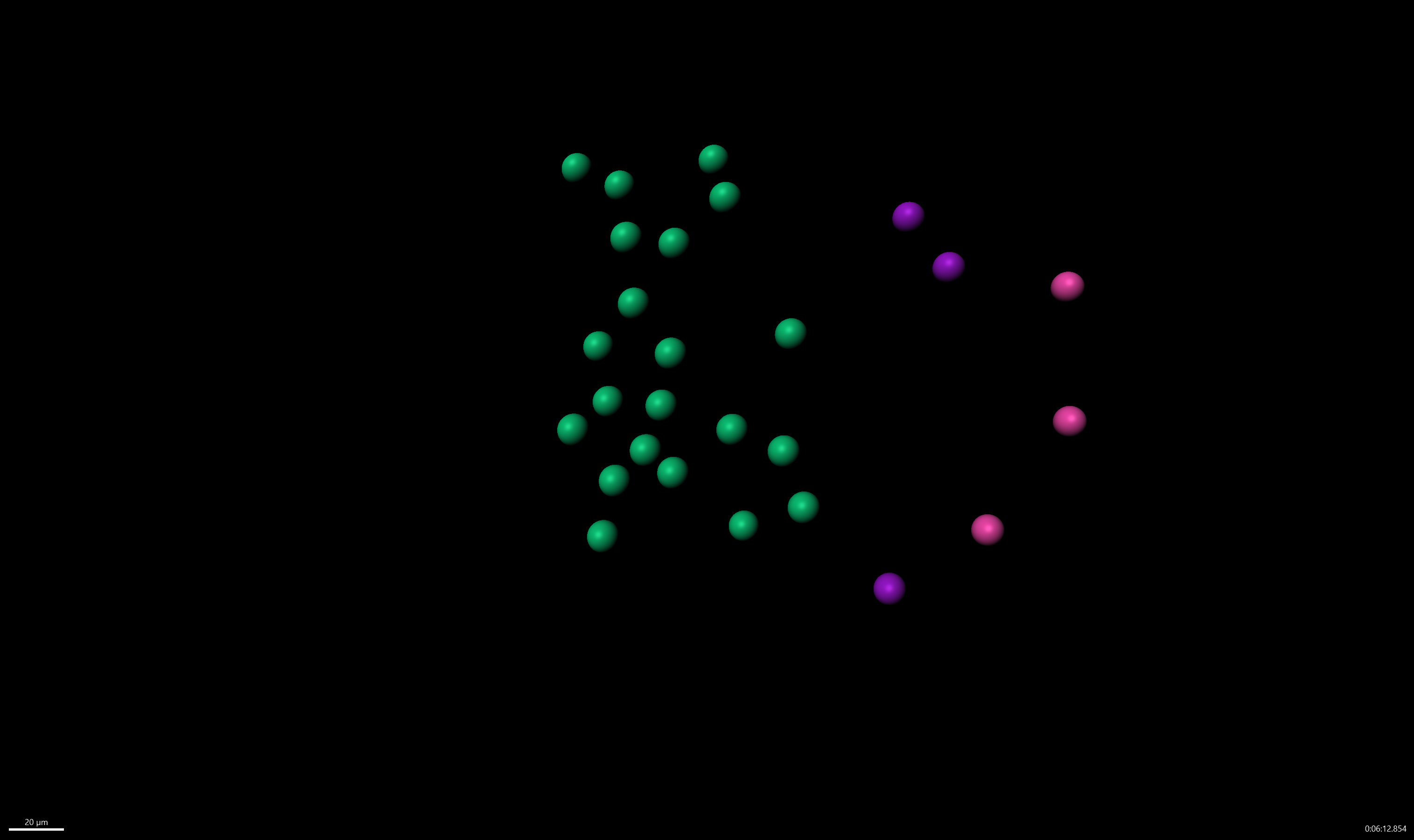

Supplement: Supplementary file 20 — Source data Fig. 4 [file 44318_2025_643_MOESM20_ESM.zip › Figure 4/4B/middle_time 5.tif]

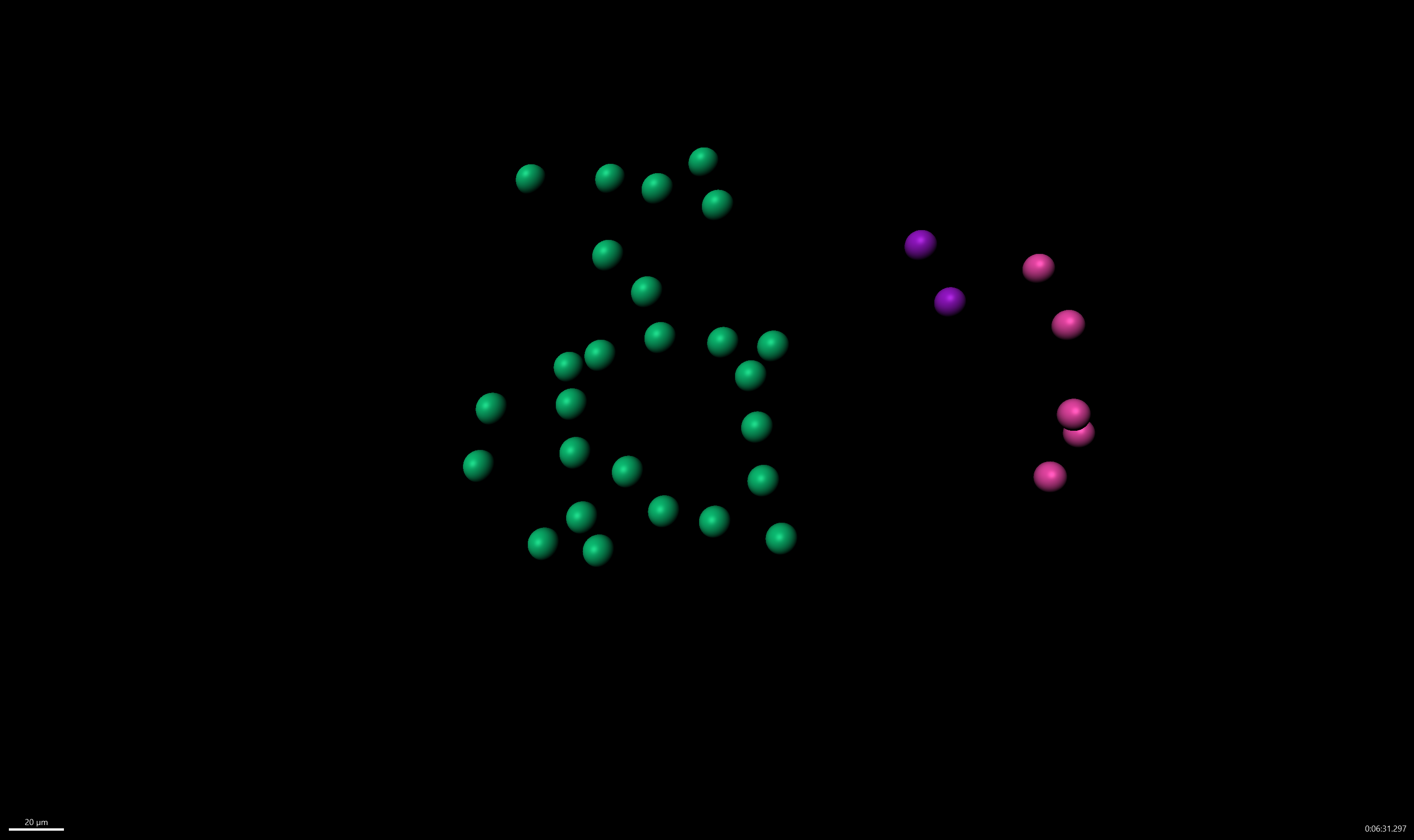

Supplement: Supplementary file 20 — Source data Fig. 4 [file 44318_2025_643_MOESM20_ESM.zip › Figure 4/4B/middle_time 6.tif]

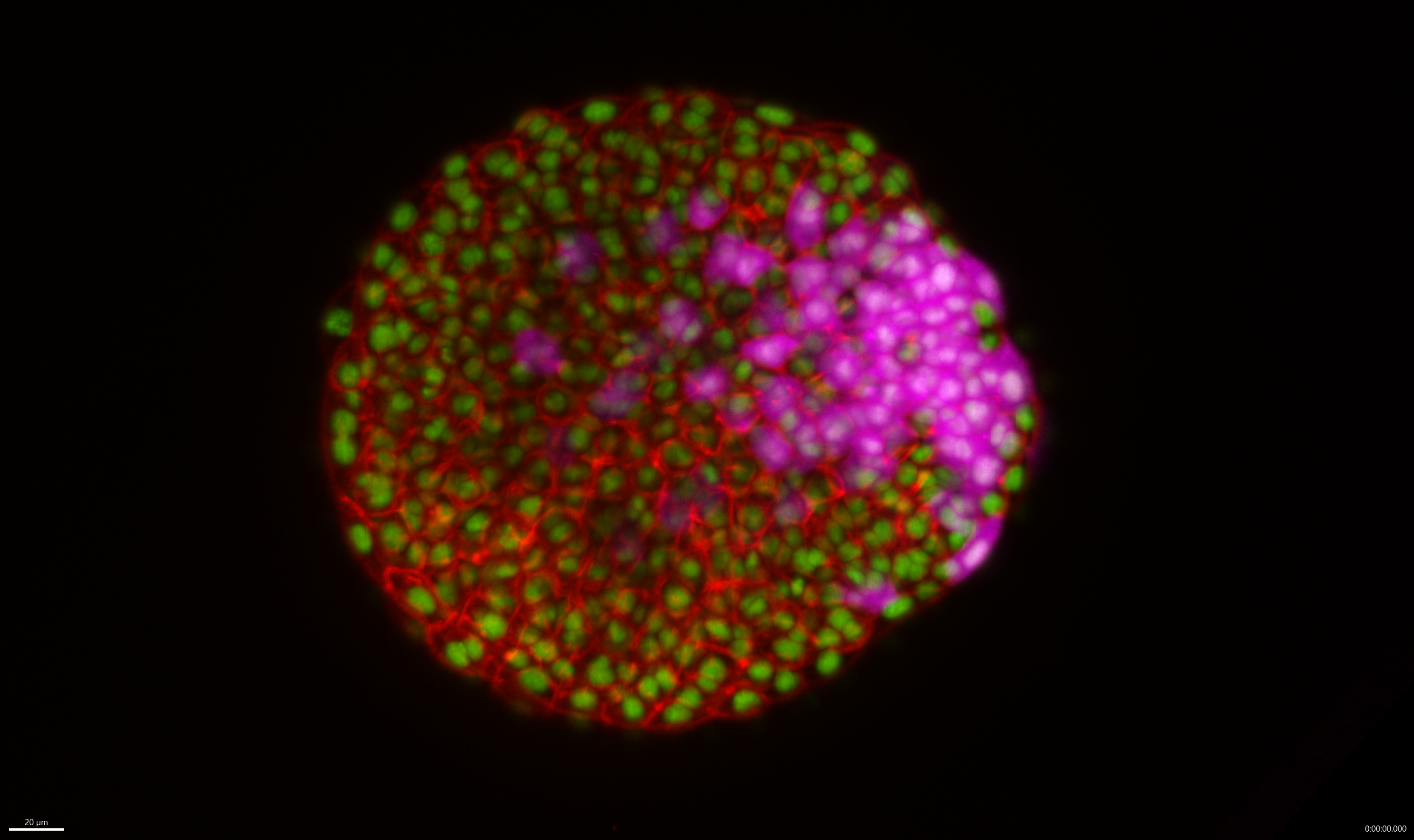

Supplement: Supplementary file 20 — Source data Fig. 4 [file 44318_2025_643_MOESM20_ESM.zip › Figure 4/4B/upper_time 1.tif]

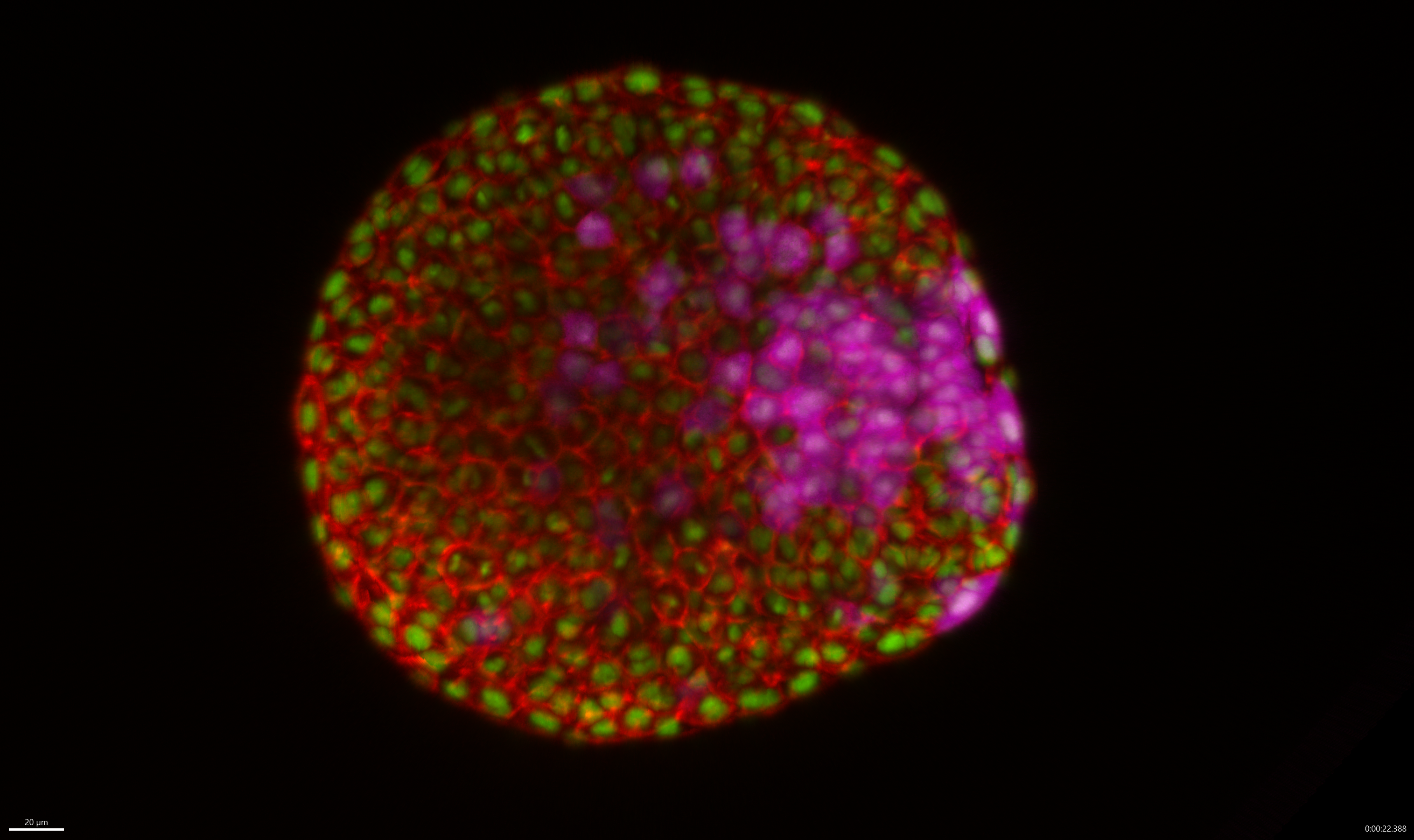

Supplement: Supplementary file 20 — Source data Fig. 4 [file 44318_2025_643_MOESM20_ESM.zip › Figure 4/4B/upper_time 2.tif]

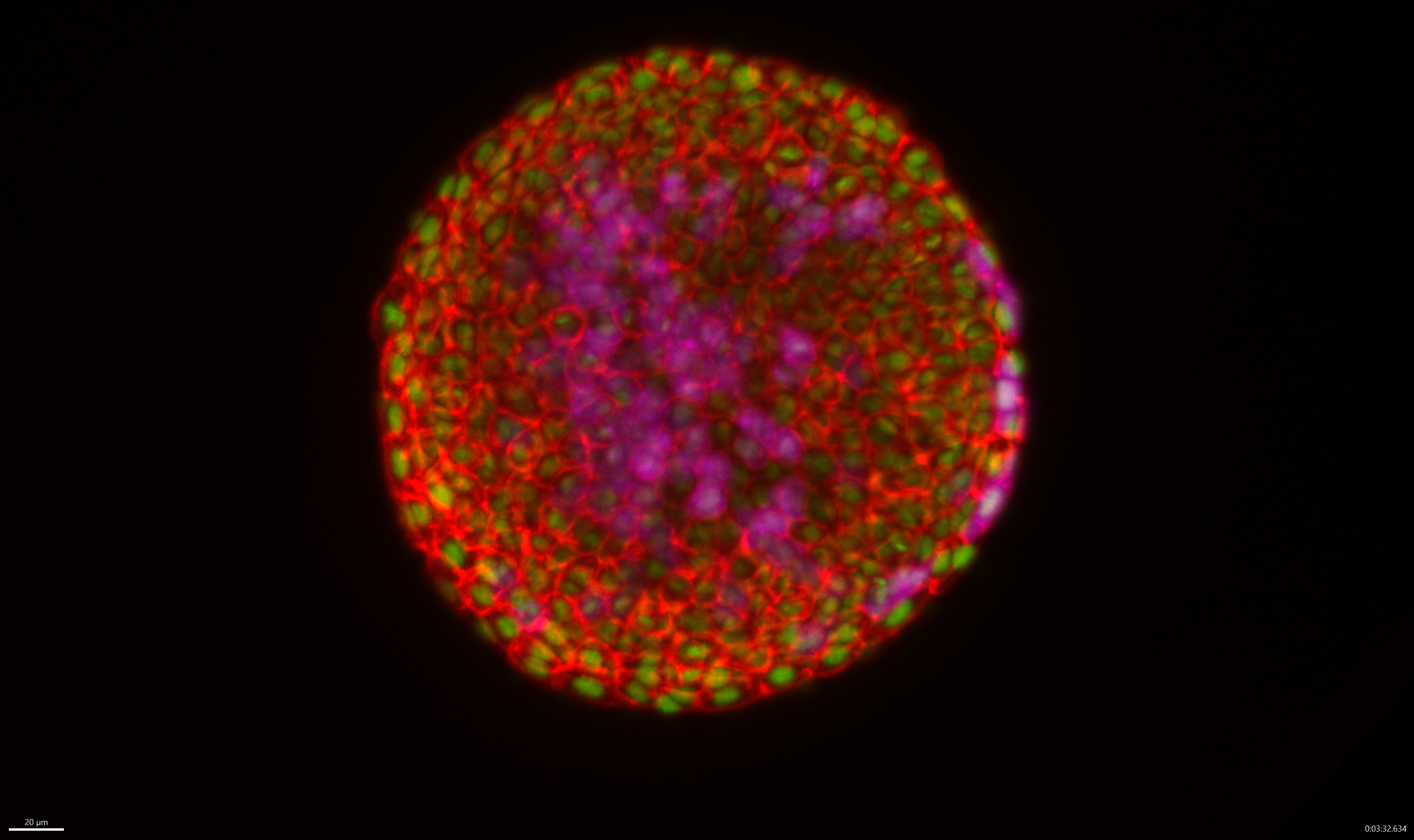

Supplement: Supplementary file 20 — Source data Fig. 4 [file 44318_2025_643_MOESM20_ESM.zip › Figure 4/4B/upper_time 4.tif]

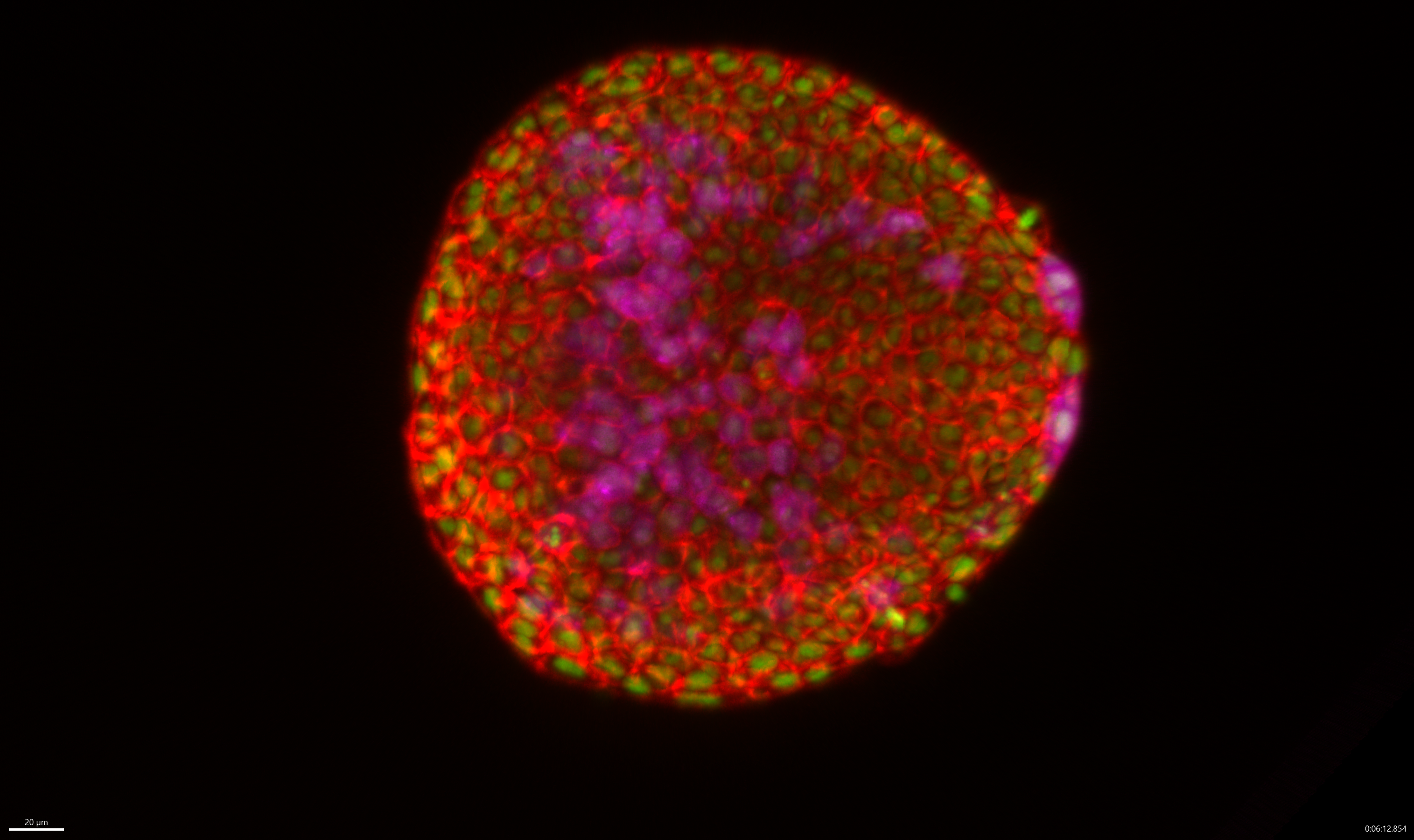

Supplement: Supplementary file 20 — Source data Fig. 4 [file 44318_2025_643_MOESM20_ESM.zip › Figure 4/4B/upper_time 5.tif]

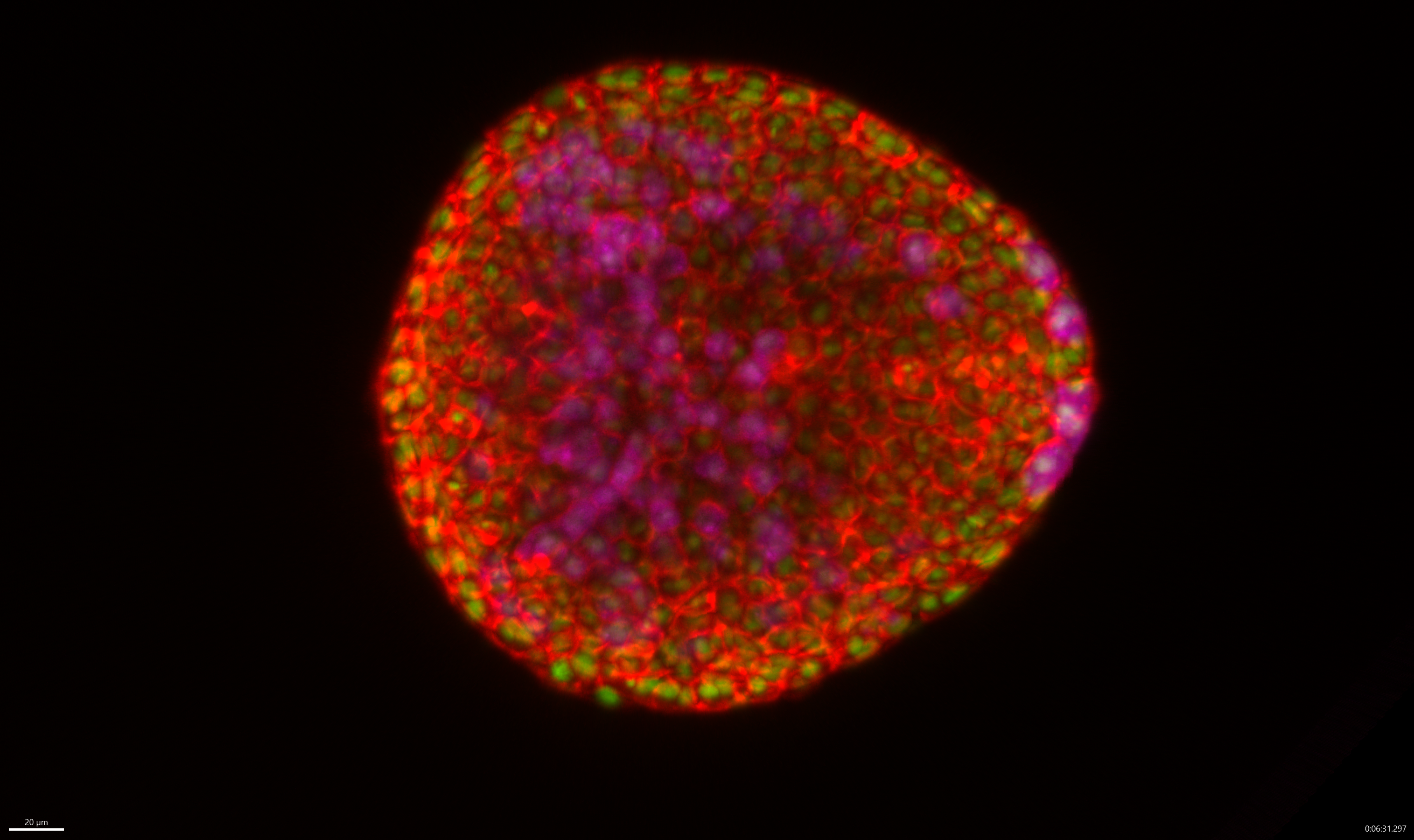

Supplement: Supplementary file 20 — Source data Fig. 4 [file 44318_2025_643_MOESM20_ESM.zip › Figure 4/4B/upper_time 6.tif]

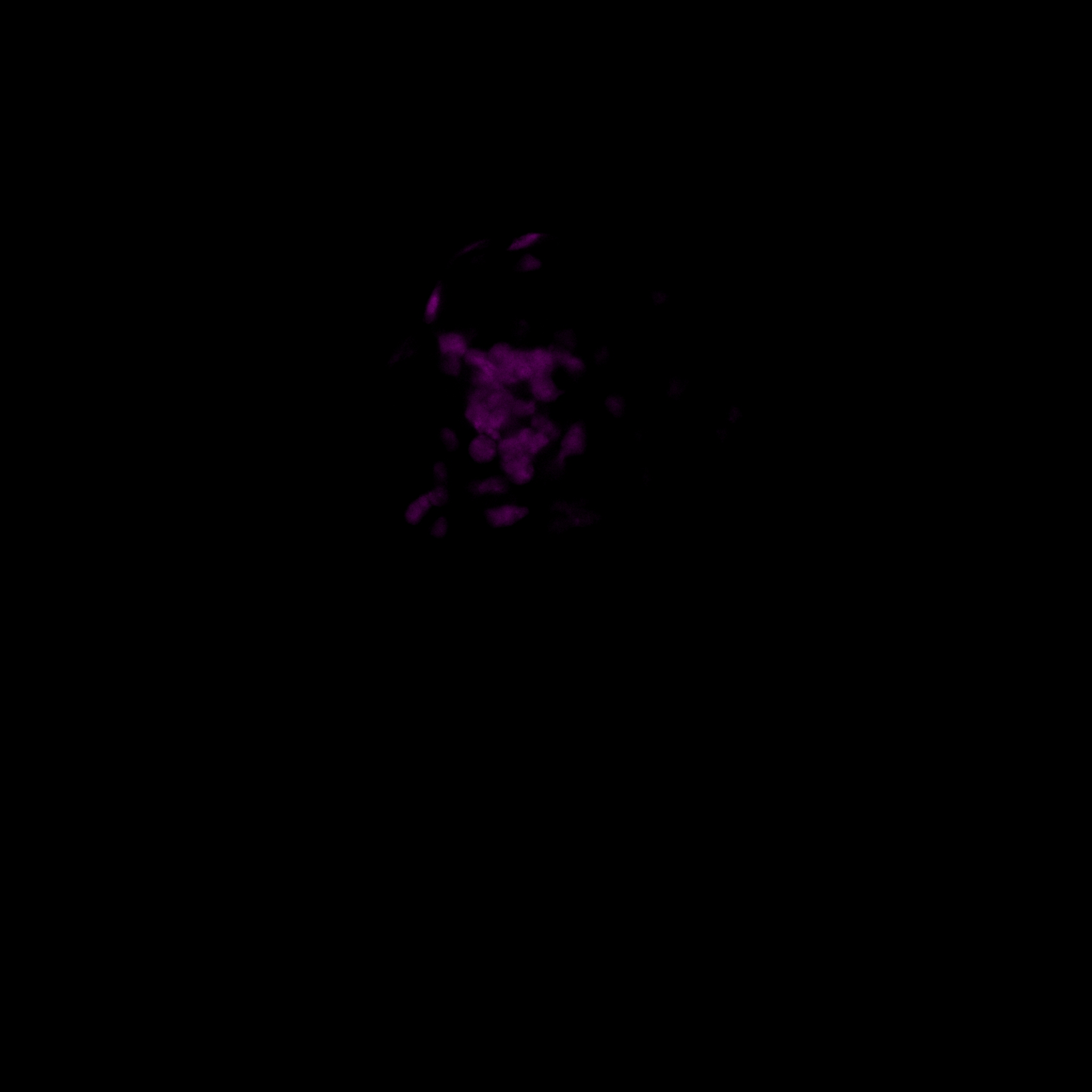

Supplement: Supplementary file 20 — Source data Fig. 4 [file 44318_2025_643_MOESM20_ESM.zip › Figure 4/4C/bmp4 explant_dextran.jpg]

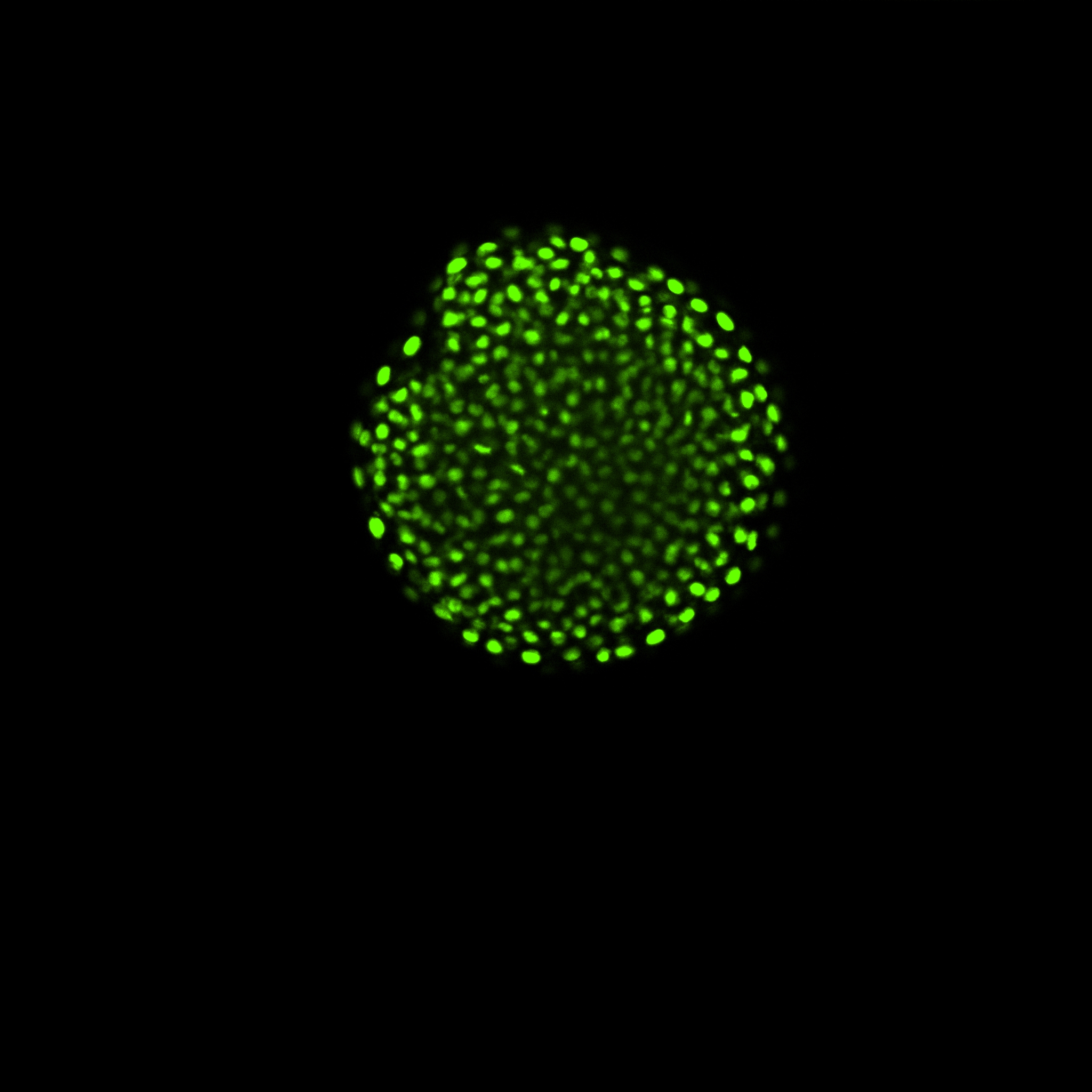

Supplement: Supplementary file 20 — Source data Fig. 4 [file 44318_2025_643_MOESM20_ESM.zip › Figure 4/4C/bmp4 explant_H2B-GFP.jpg]

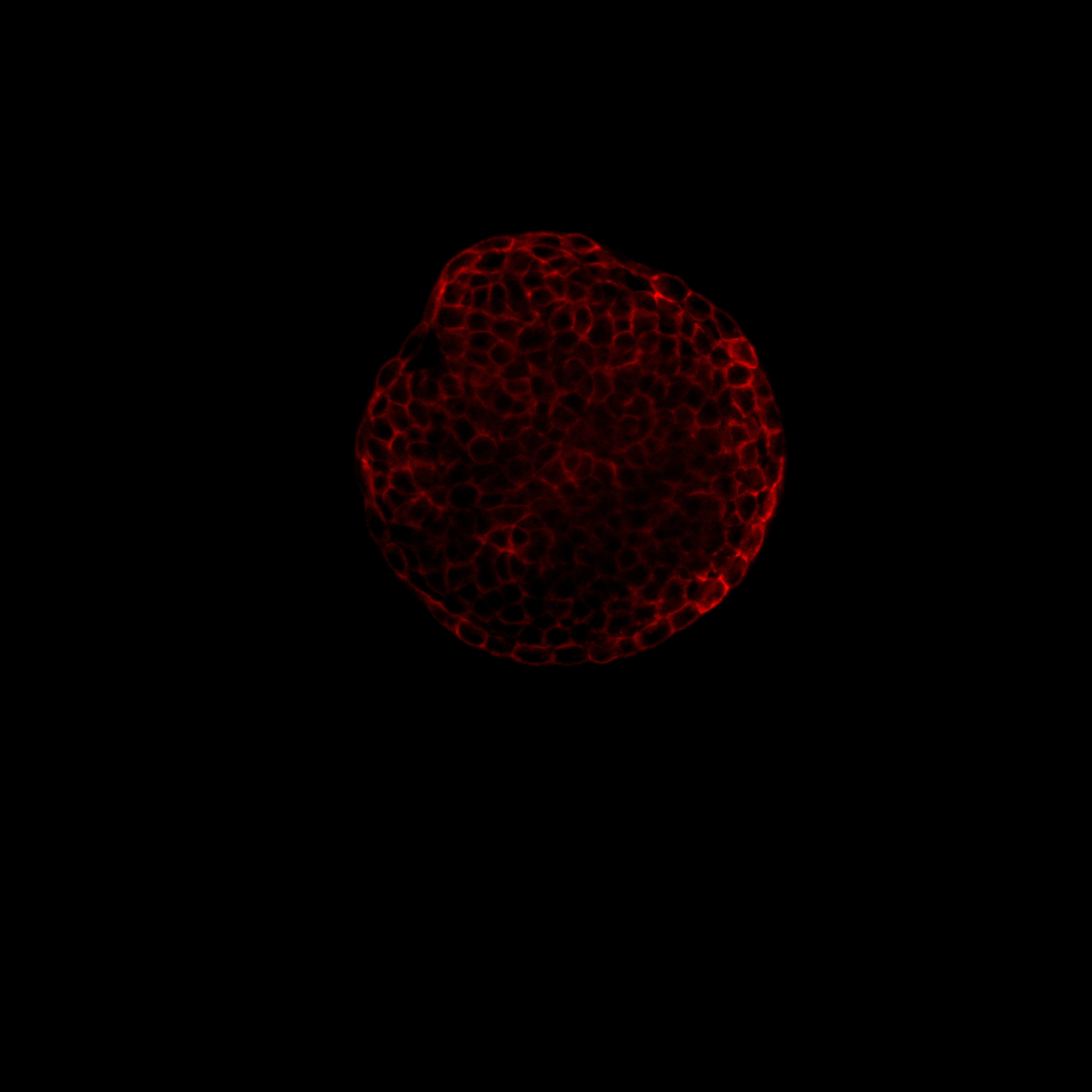

Supplement: Supplementary file 20 — Source data Fig. 4 [file 44318_2025_643_MOESM20_ESM.zip › Figure 4/4C/bmp4 explant_mcherry.jpg]

Movement Direction Distribution (XY Plane)

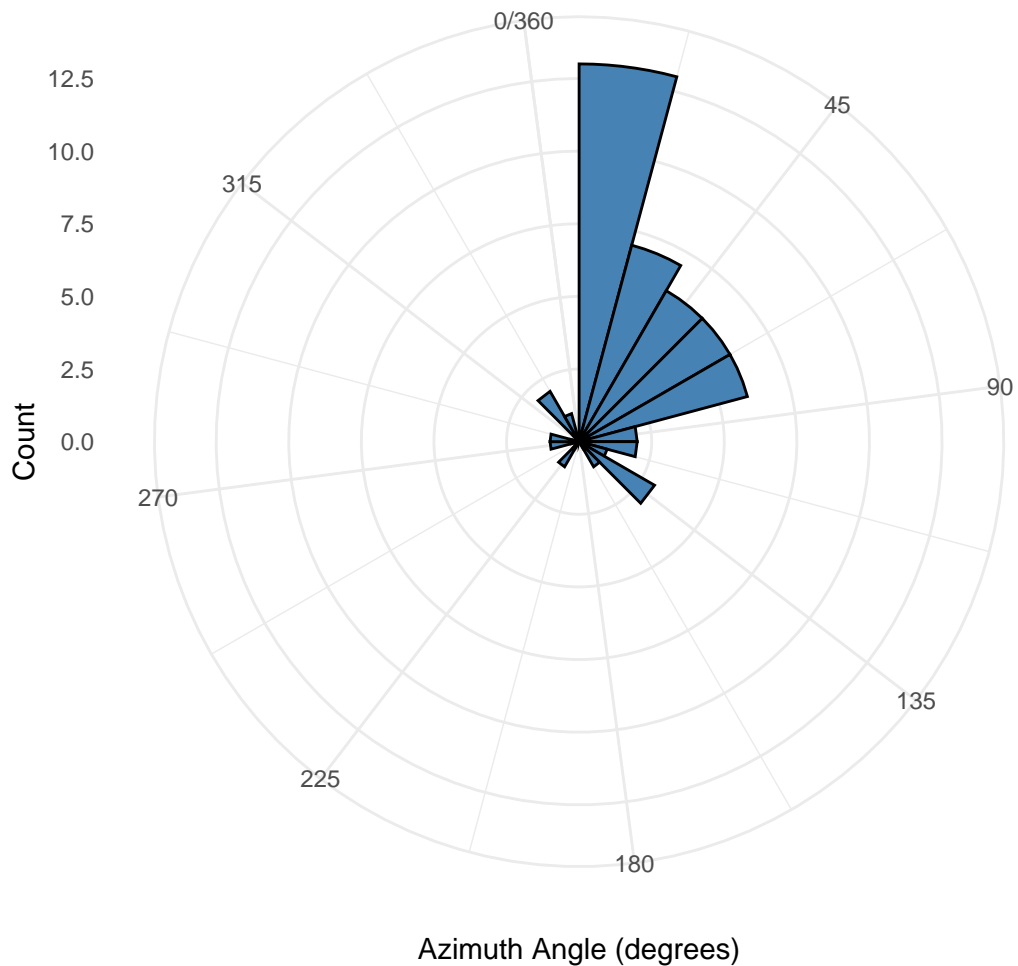

Supplement: Supplementary file 20 — Source data Fig. 4 [file 44318_2025_643_MOESM20_ESM.zip › Figure 4/4E-4F/bmp4 explants_internalization_angle_early.pdf]

Movement Direction Distribution (XY Plane)

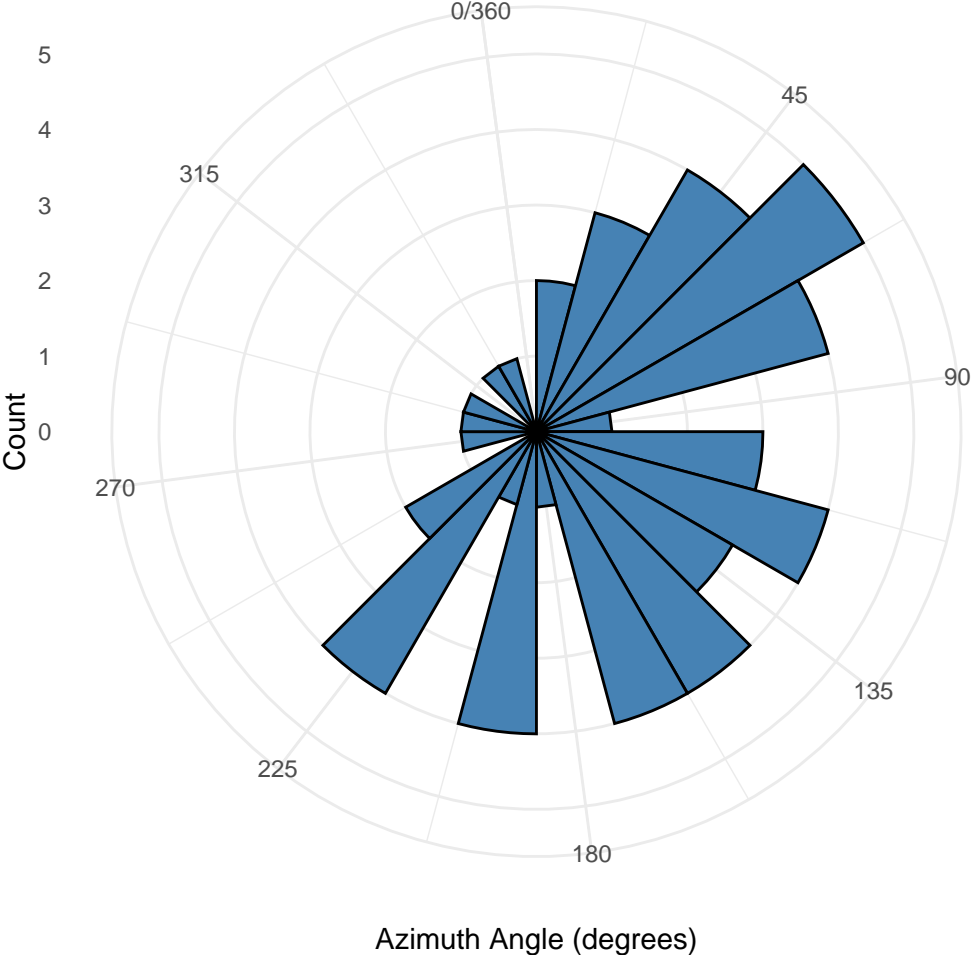

Supplement: Supplementary file 20 — Source data Fig. 4 [file 44318_2025_643_MOESM20_ESM.zip › Figure 4/4E-4F/bmp4 explants_internalization_angle_late.pdf]

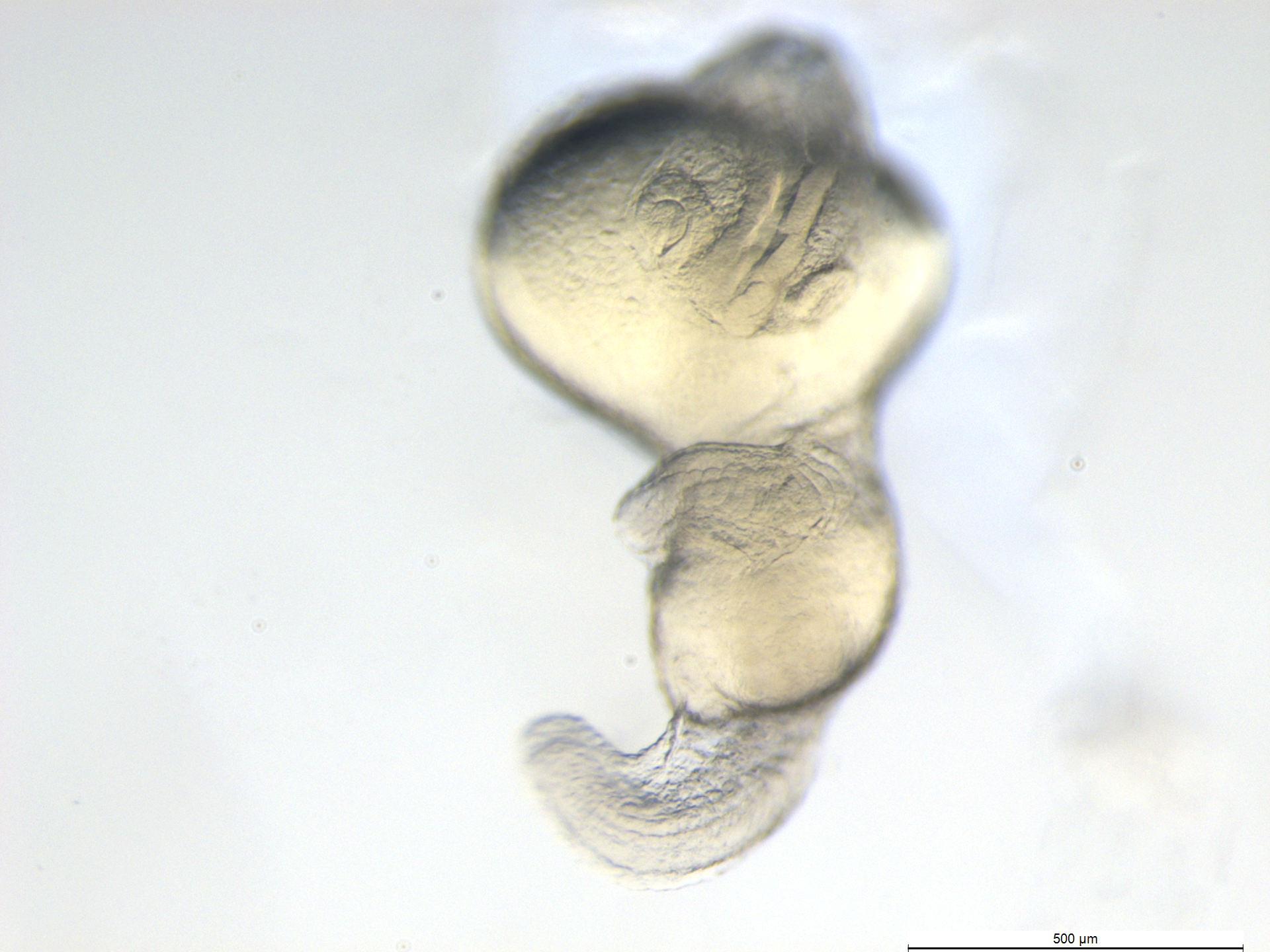

Supplement: Supplementary file 21 — Source data Fig. 5 [file 44318_2025_643_MOESM21_ESM.zip › Figure 5/5B/20230713_BMP4human_transplant_4h_zebrafish_24h_6.3x_3-B.tif]

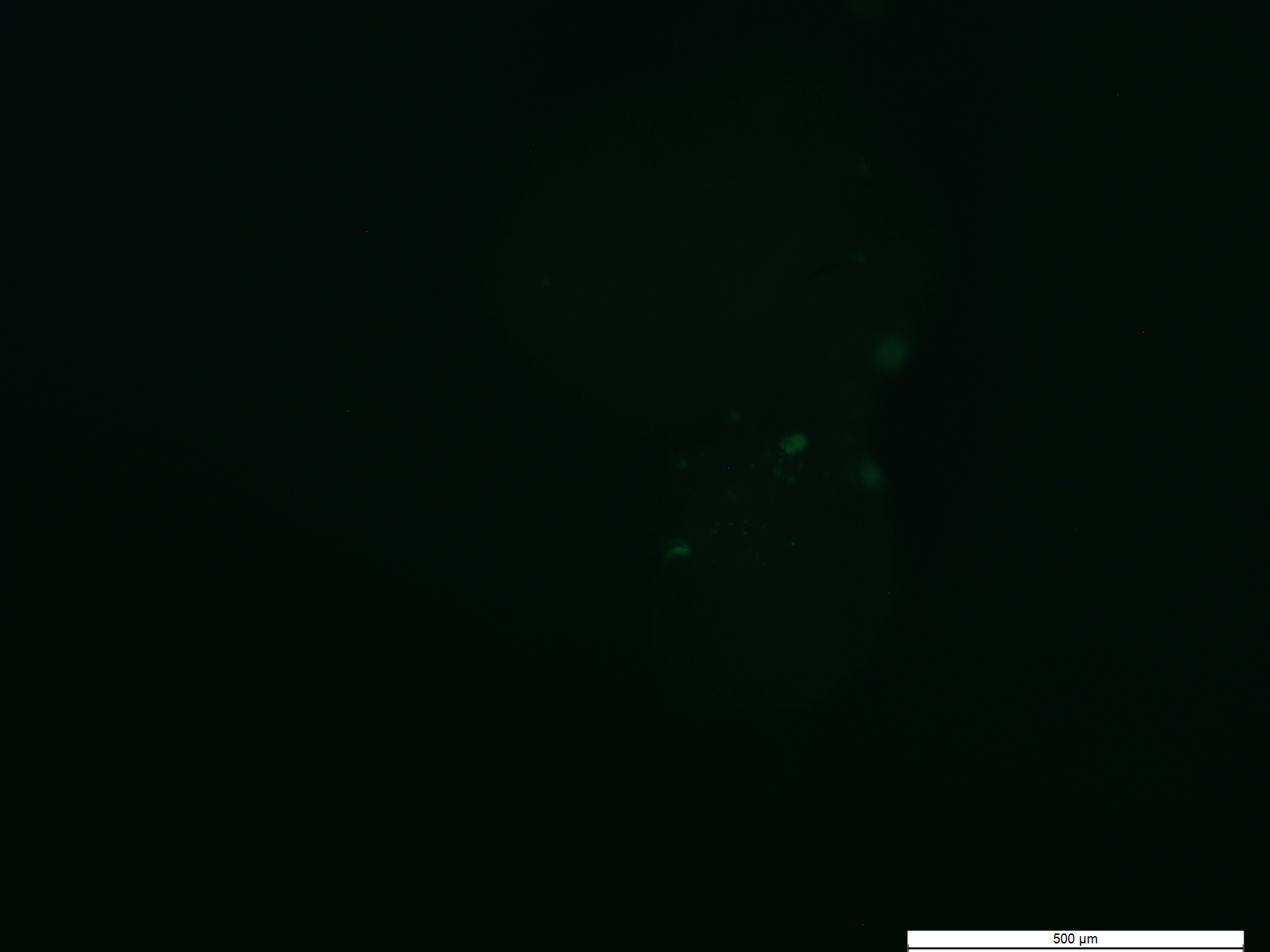

Supplement: Supplementary file 21 — Source data Fig. 5 [file 44318_2025_643_MOESM21_ESM.zip › Figure 5/5B/20230713_BMP4human_transplant_4h_zebrafish_24h_6.3x_3-G.tif]

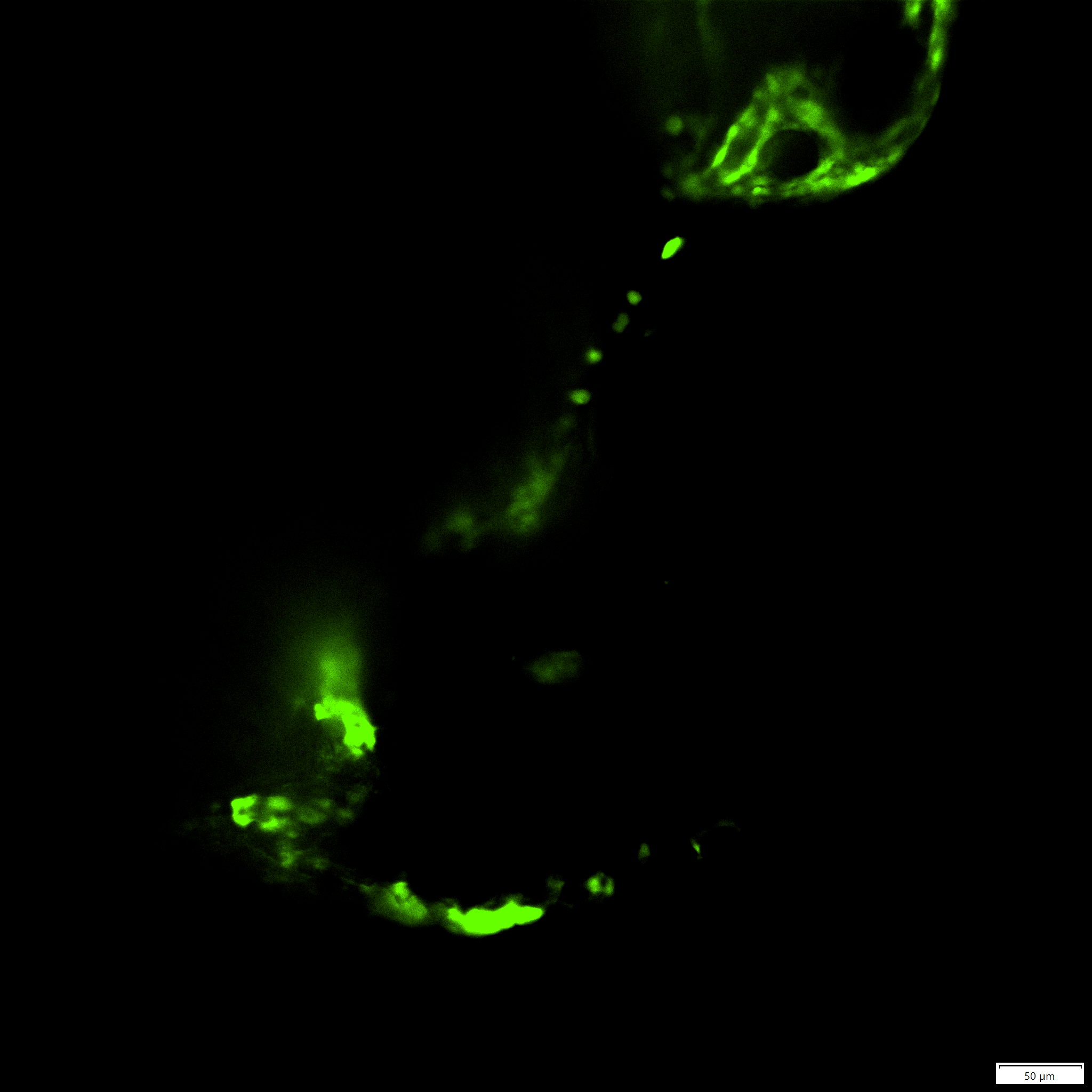

Supplement: Supplementary file 21 — Source data Fig. 5 [file 44318_2025_643_MOESM21_ESM.zip › Figure 5/5C/human_BMP4cell_transplant_zebrafish_24h-2_01-Z51-g.png]

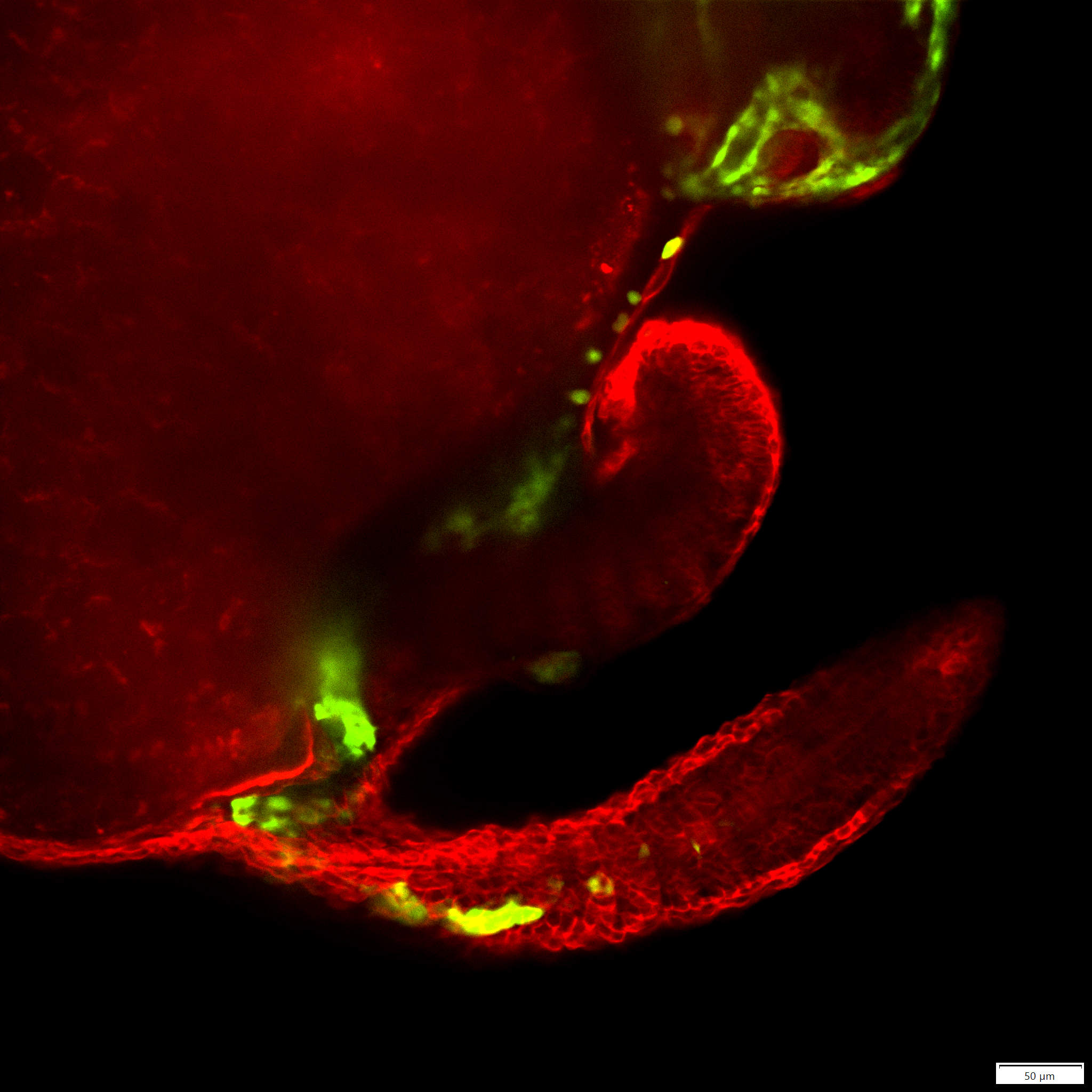

Supplement: Supplementary file 21 — Source data Fig. 5 [file 44318_2025_643_MOESM21_ESM.zip › Figure 5/5C/human_BMP4cell_transplant_zebrafish_24h-2_01-Z51-merge.png]

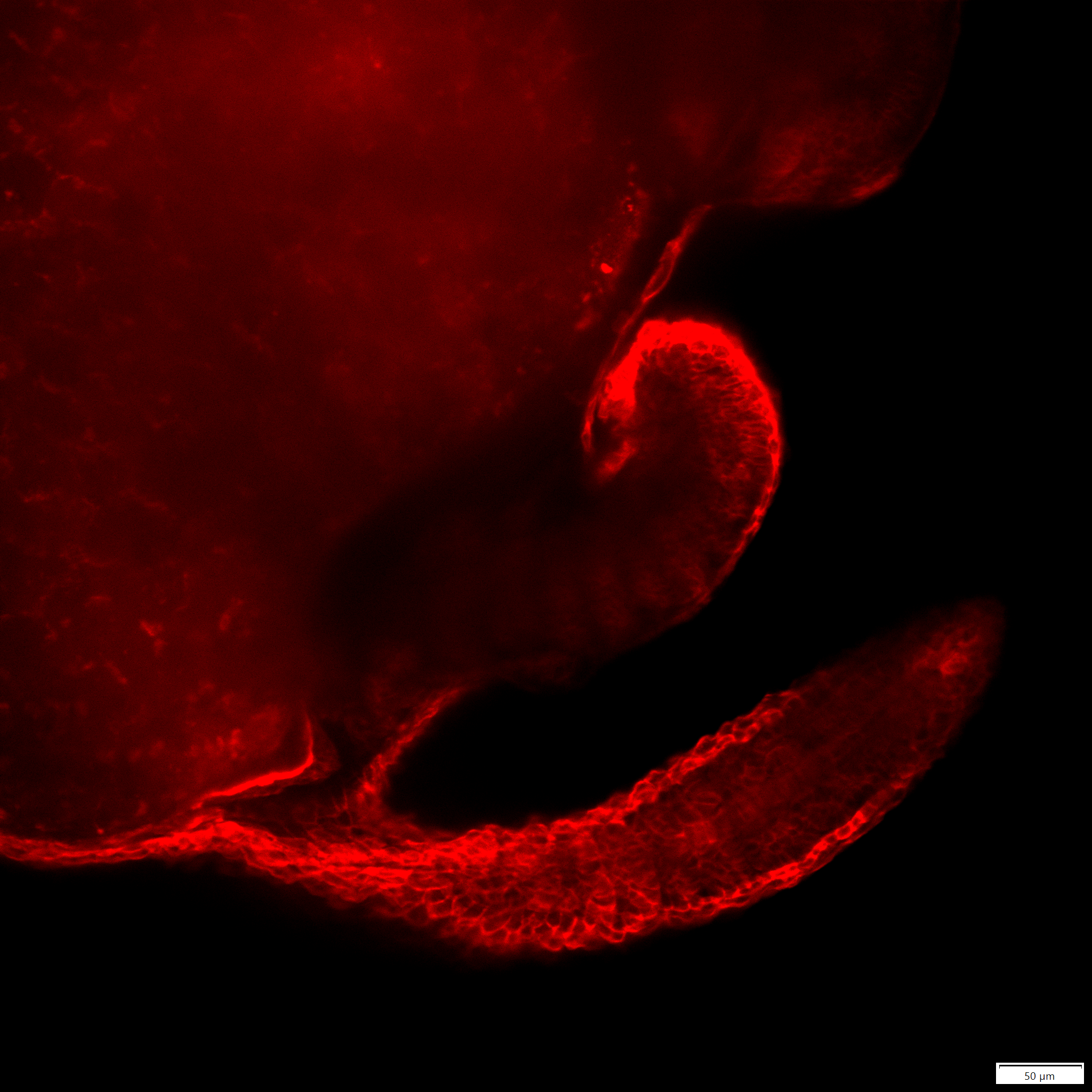

Supplement: Supplementary file 21 — Source data Fig. 5 [file 44318_2025_643_MOESM21_ESM.zip › Figure 5/5C/human_BMP4cell_transplant_zebrafish_24h-2_01-Z51-r.png]

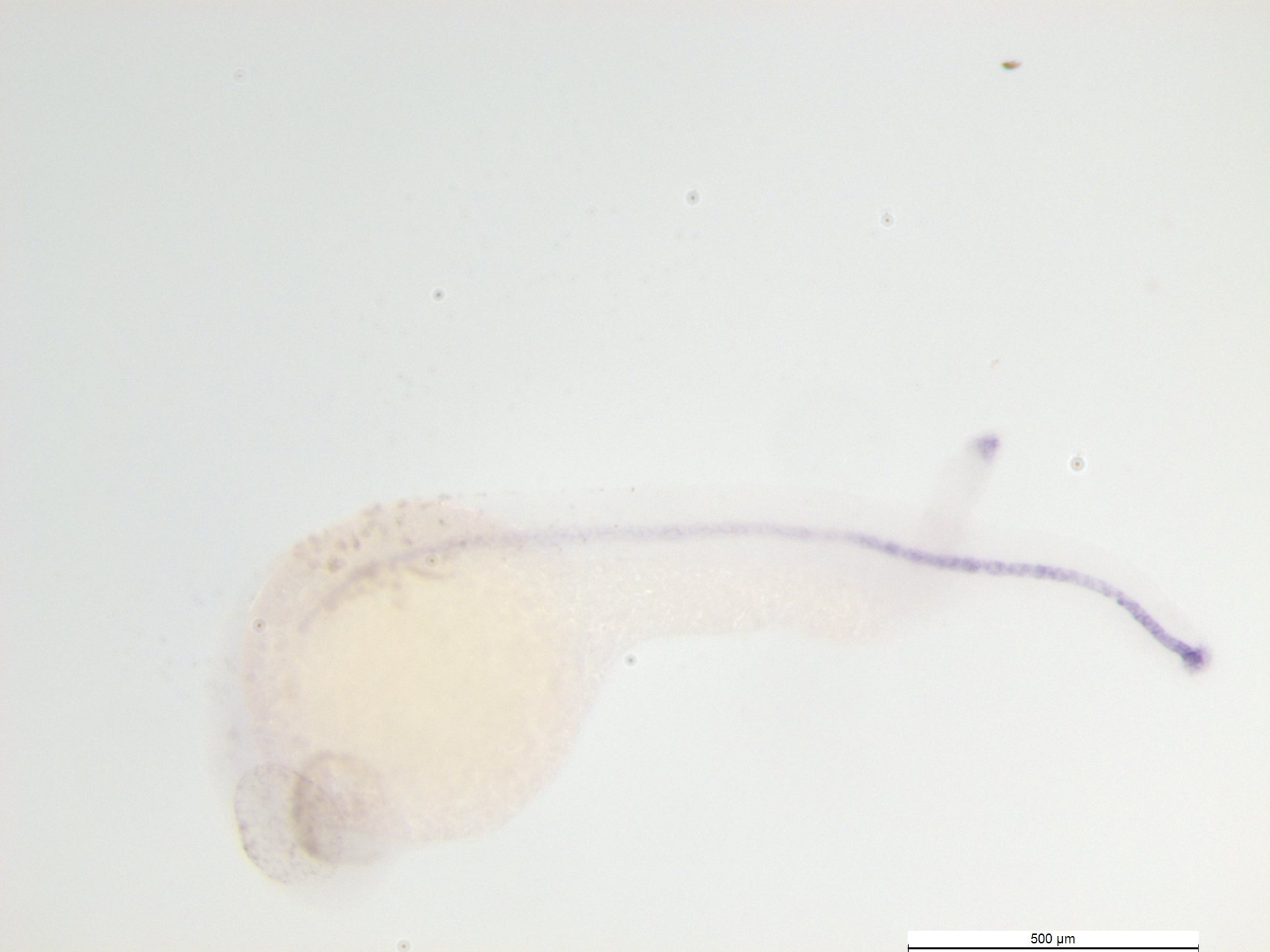

Supplement: Supplementary file 21 — Source data Fig. 5 [file 44318_2025_643_MOESM21_ESM.zip › Figure 5/5D/xenograft_24hpf_tbxta.tif]

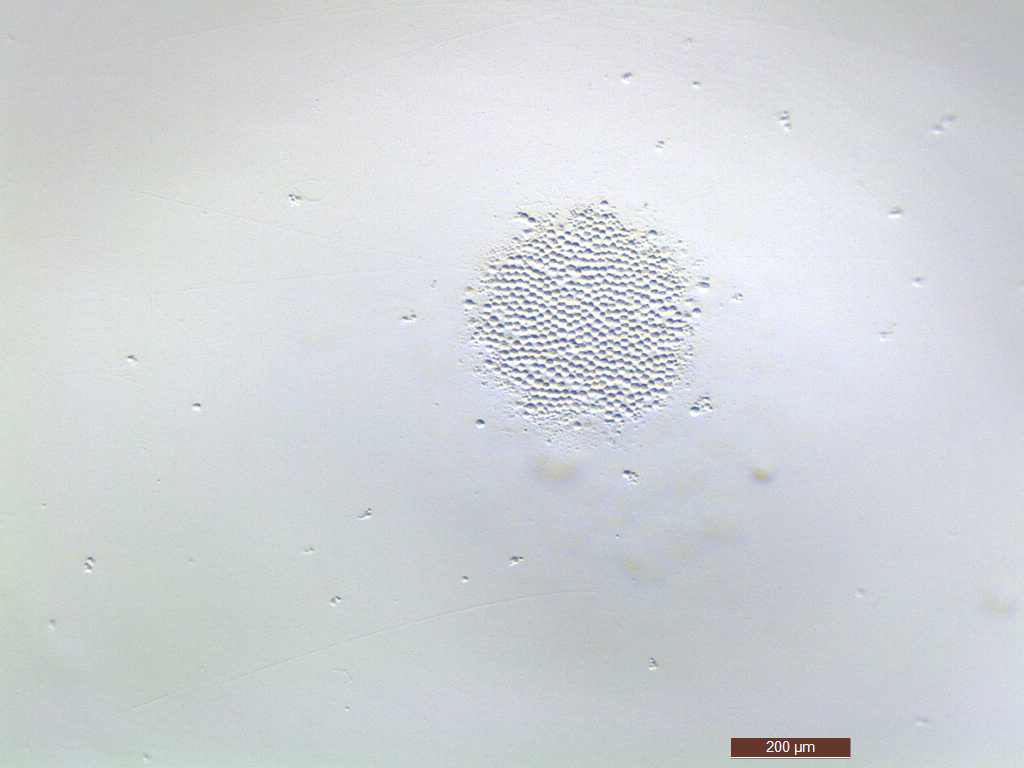

Supplement: Supplementary file 22 — Source data Fig. 6 [file 44318_2025_643_MOESM22_ESM.zip › Figure 6/6A/D0.tif]

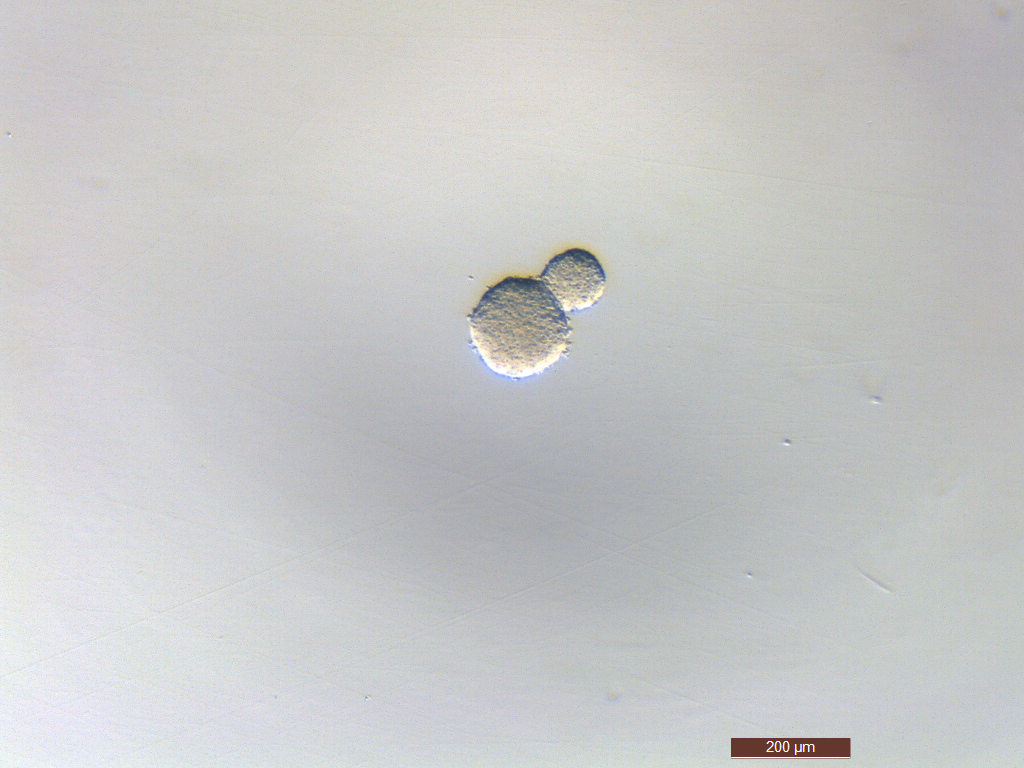

Supplement: Supplementary file 22 — Source data Fig. 6 [file 44318_2025_643_MOESM22_ESM.zip › Figure 6/6A/D1.tif]

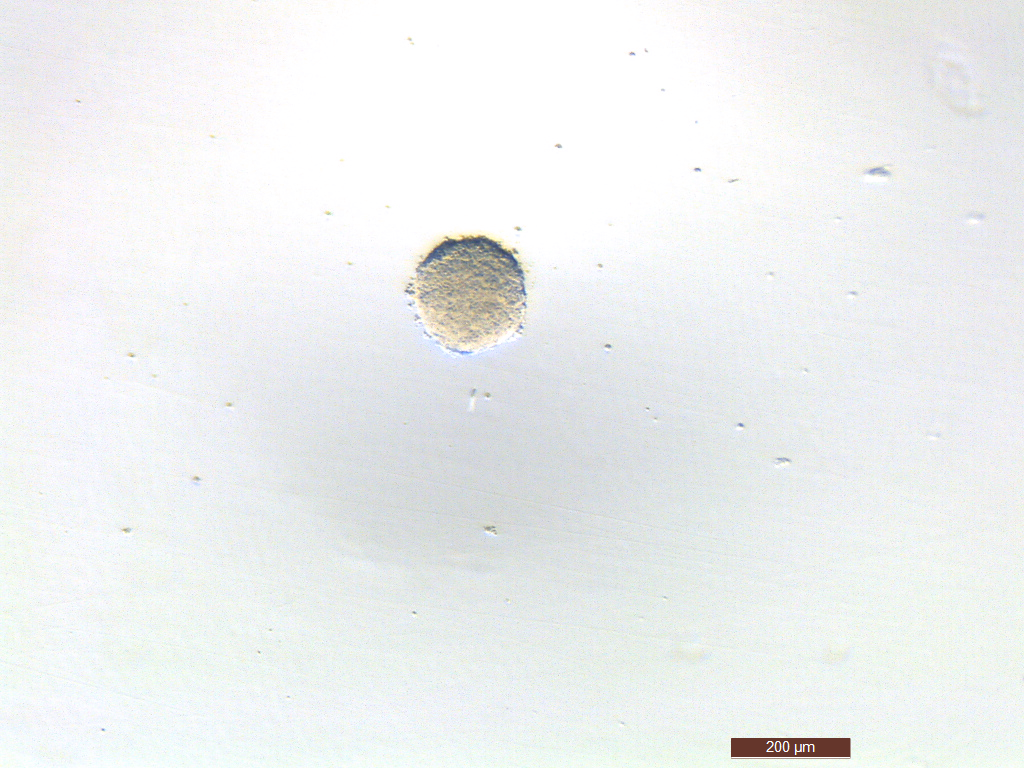

Supplement: Supplementary file 22 — Source data Fig. 6 [file 44318_2025_643_MOESM22_ESM.zip › Figure 6/6A/D2.tif]

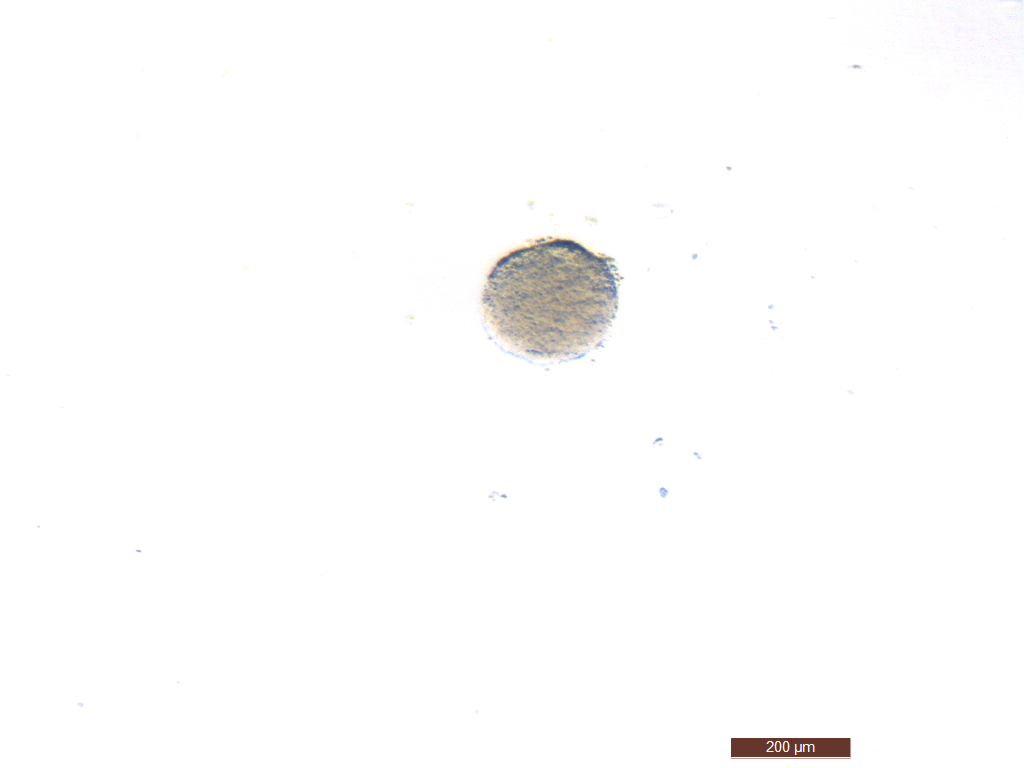

Supplement: Supplementary file 22 — Source data Fig. 6 [file 44318_2025_643_MOESM22_ESM.zip › Figure 6/6A/D3.tif]

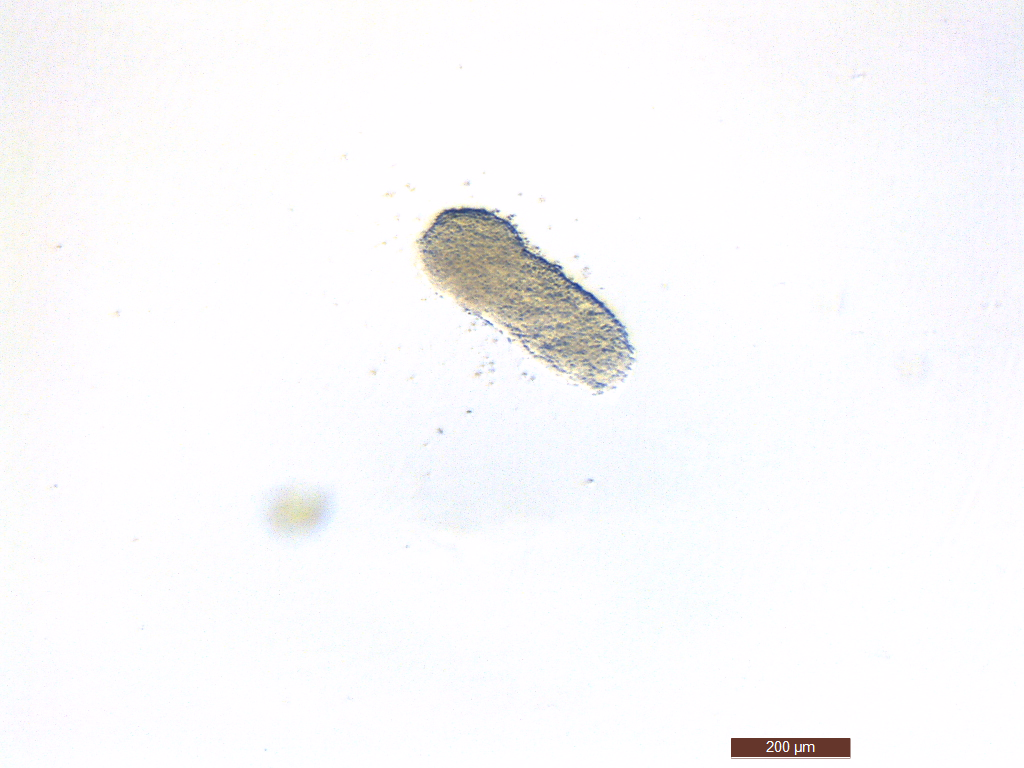

Supplement: Supplementary file 22 — Source data Fig. 6 [file 44318_2025_643_MOESM22_ESM.zip › Figure 6/6A/D4.tif]

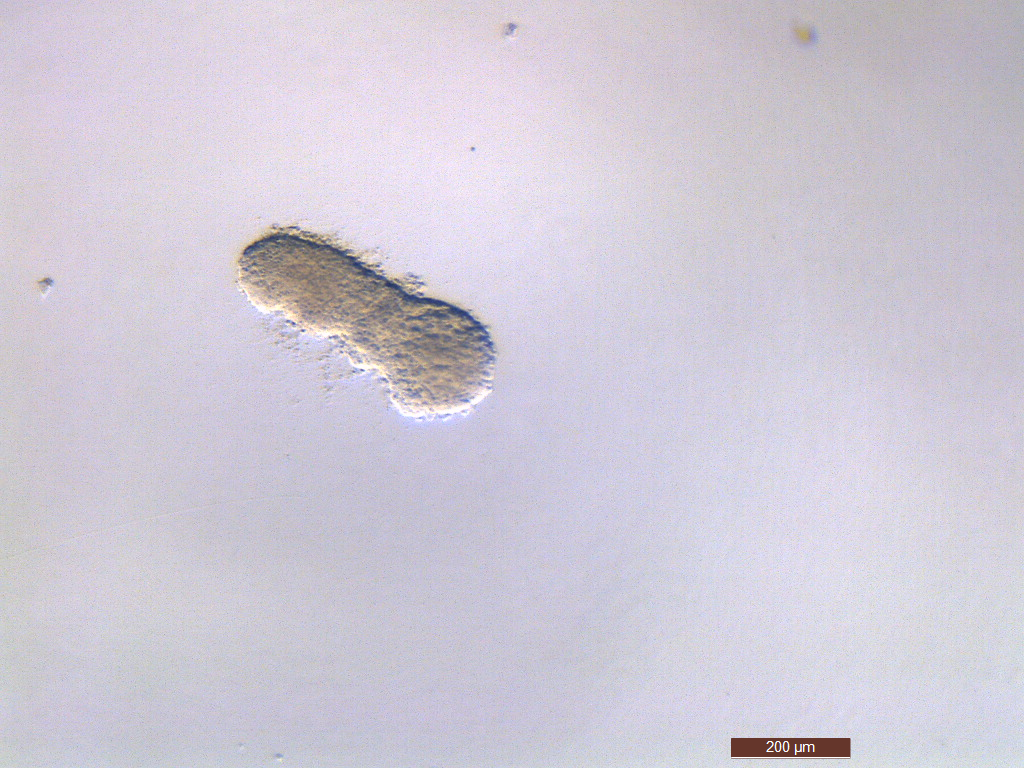

Supplement: Supplementary file 22 — Source data Fig. 6 [file 44318_2025_643_MOESM22_ESM.zip › Figure 6/6A/D5.tif]

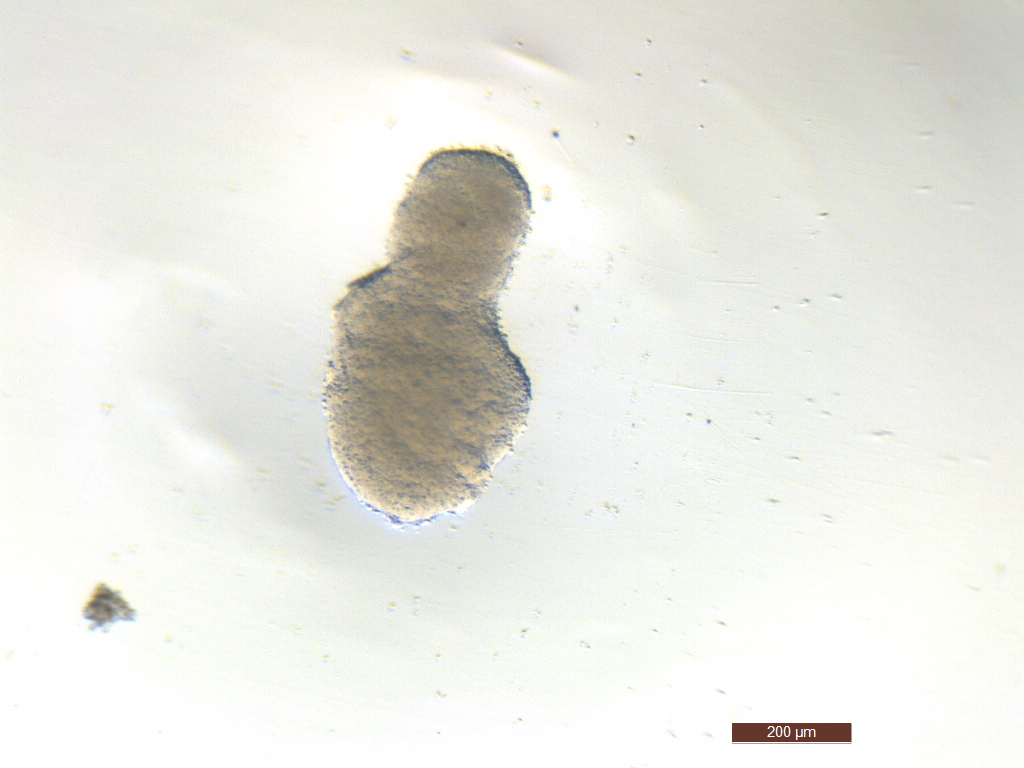

Supplement: Supplementary file 22 — Source data Fig. 6 [file 44318_2025_643_MOESM22_ESM.zip › Figure 6/6A/D6.tif]

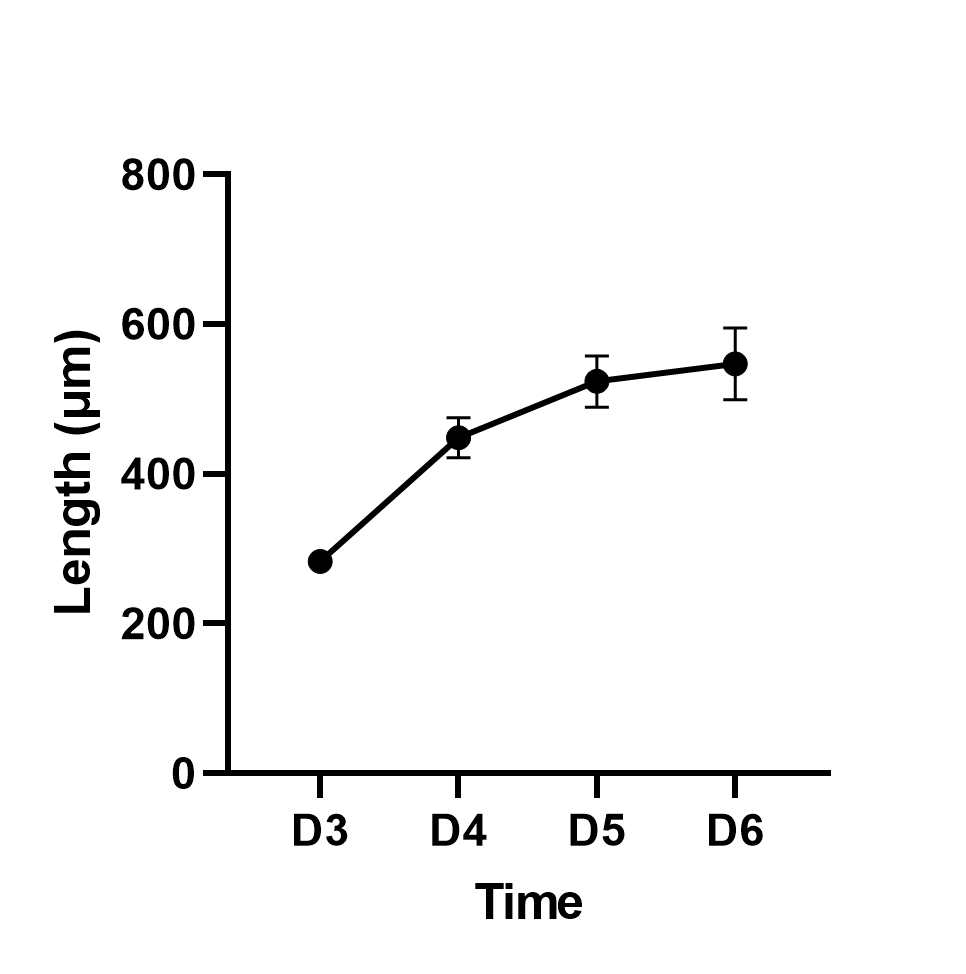

Supplement: Supplementary file 22 — Source data Fig. 6 [file 44318_2025_643_MOESM22_ESM.zip › Figure 6/6B/Length.tif]

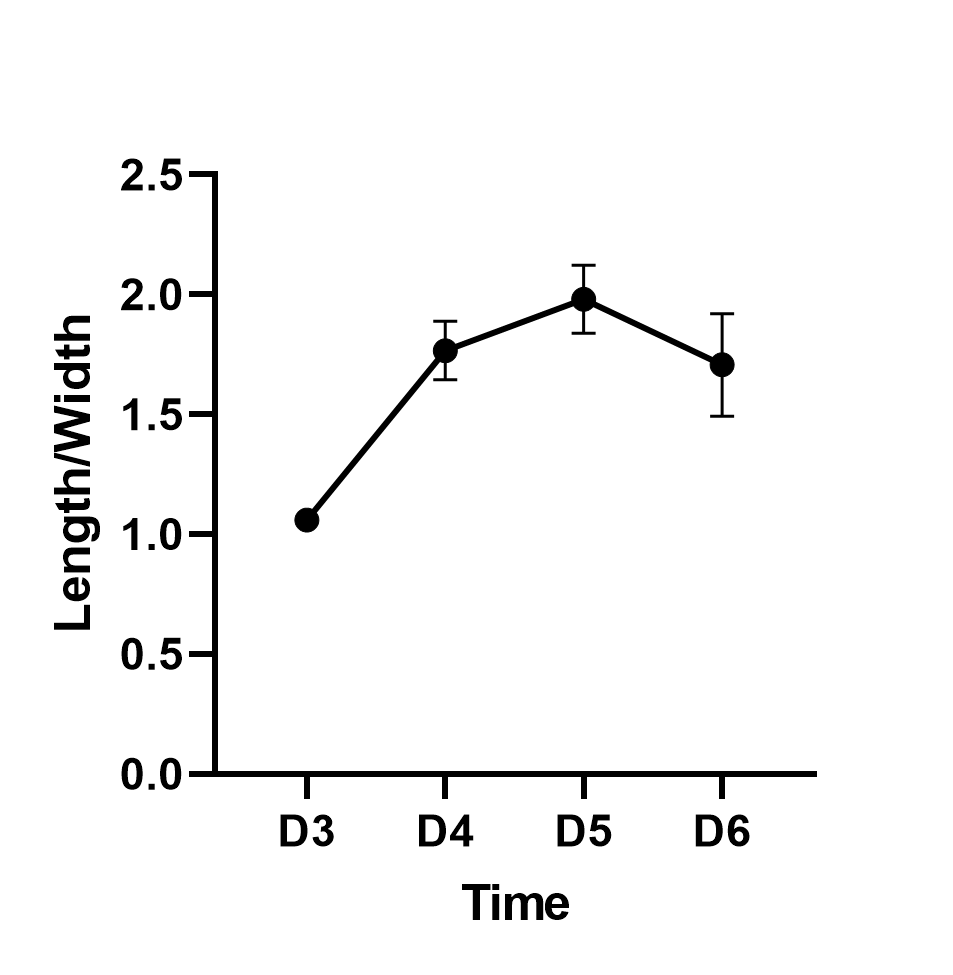

Supplement: Supplementary file 22 — Source data Fig. 6 [file 44318_2025_643_MOESM22_ESM.zip › Figure 6/6B/Ration.tif]

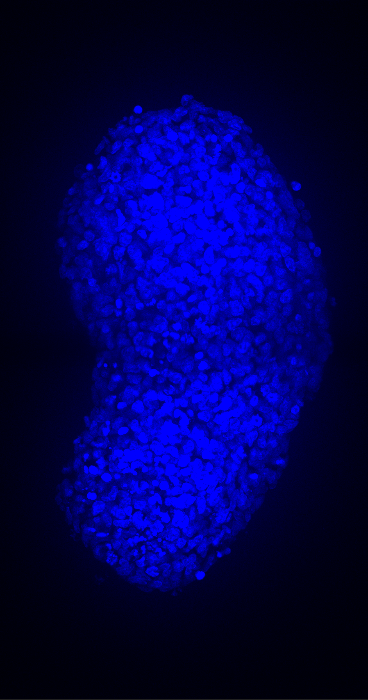

Supplement: Supplementary file 22 — Source data Fig. 6 [file 44318_2025_643_MOESM22_ESM.zip › Figure 6/6C/EB_DAPI.tif]

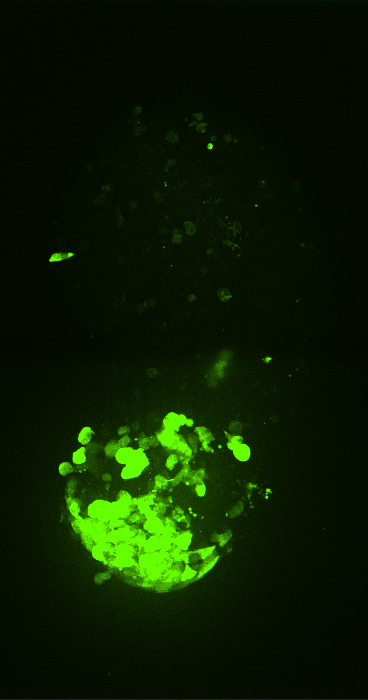

Supplement: Supplementary file 22 — Source data Fig. 6 [file 44318_2025_643_MOESM22_ESM.zip › Figure 6/6C/EB_GFP.tif]

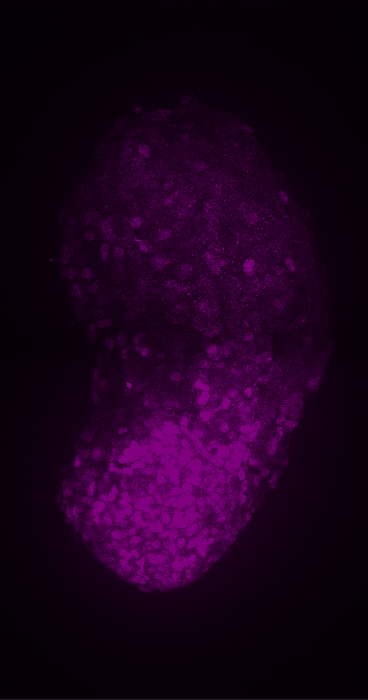

Supplement: Supplementary file 22 — Source data Fig. 6 [file 44318_2025_643_MOESM22_ESM.zip › Figure 6/6C/EB_ISH_pSmad159.tif]

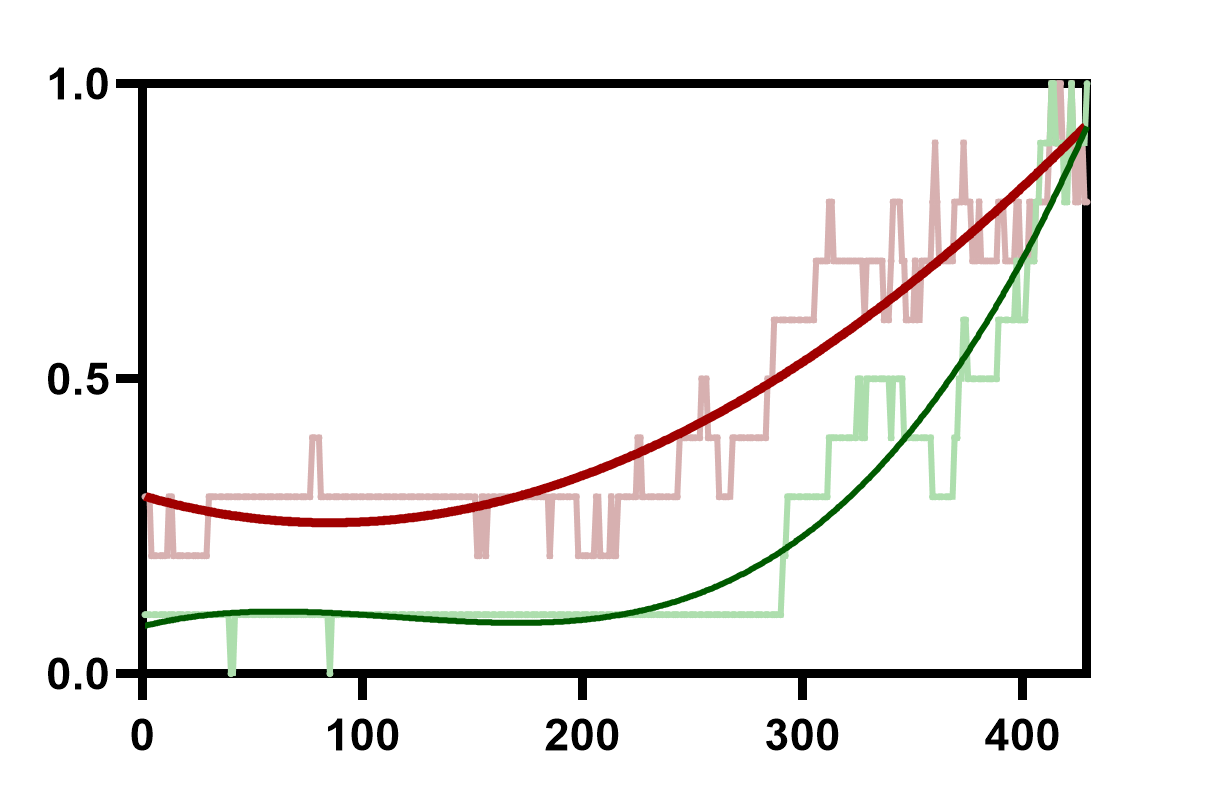

Supplement: Supplementary file 22 — Source data Fig. 6 [file 44318_2025_643_MOESM22_ESM.zip › Figure 6/6D/Data 1.tif]

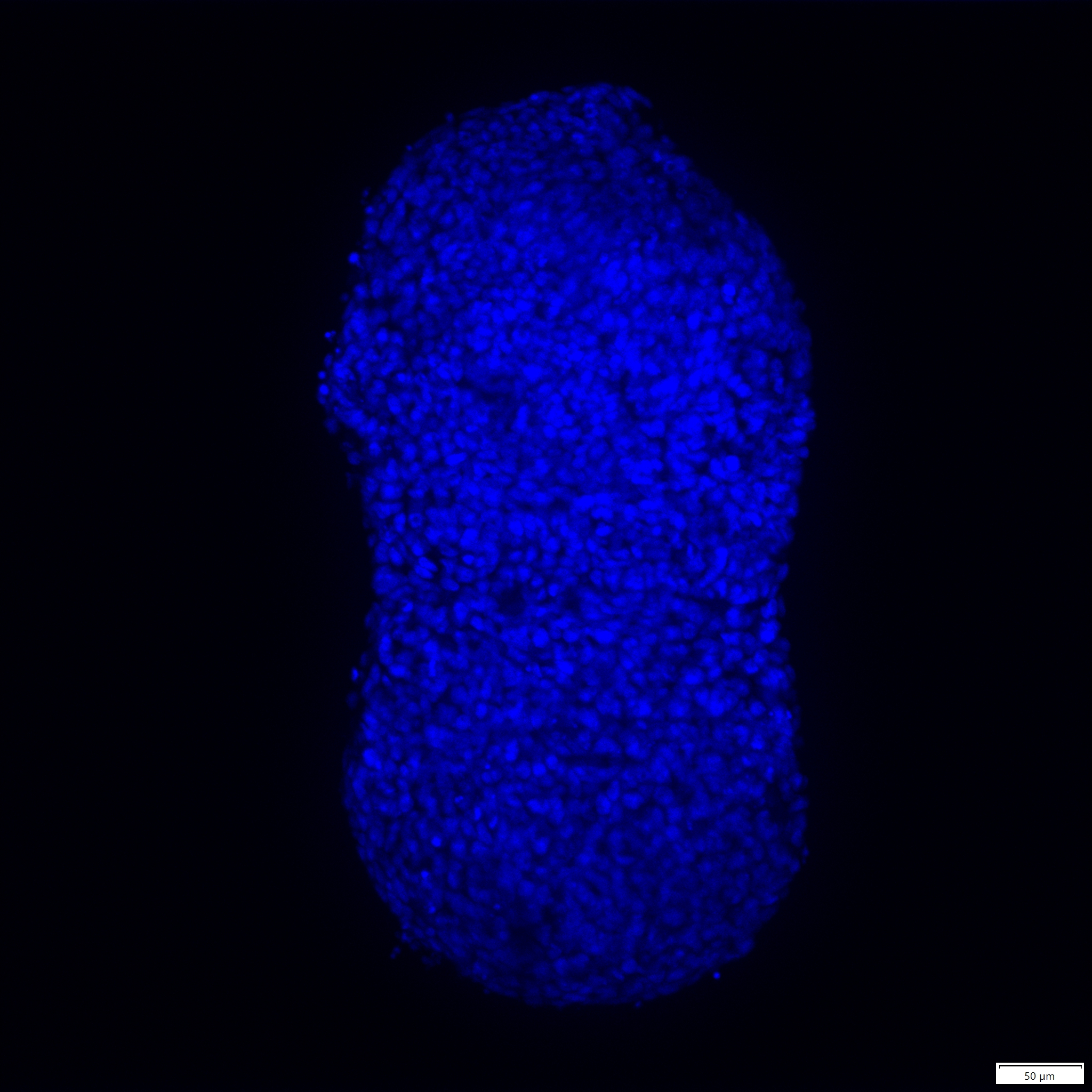

Supplement: Supplementary file 22 — Source data Fig. 6 [file 44318_2025_643_MOESM22_ESM.zip › Figure 6/6E/BMP4 EB_D3_DAPI.jpg]

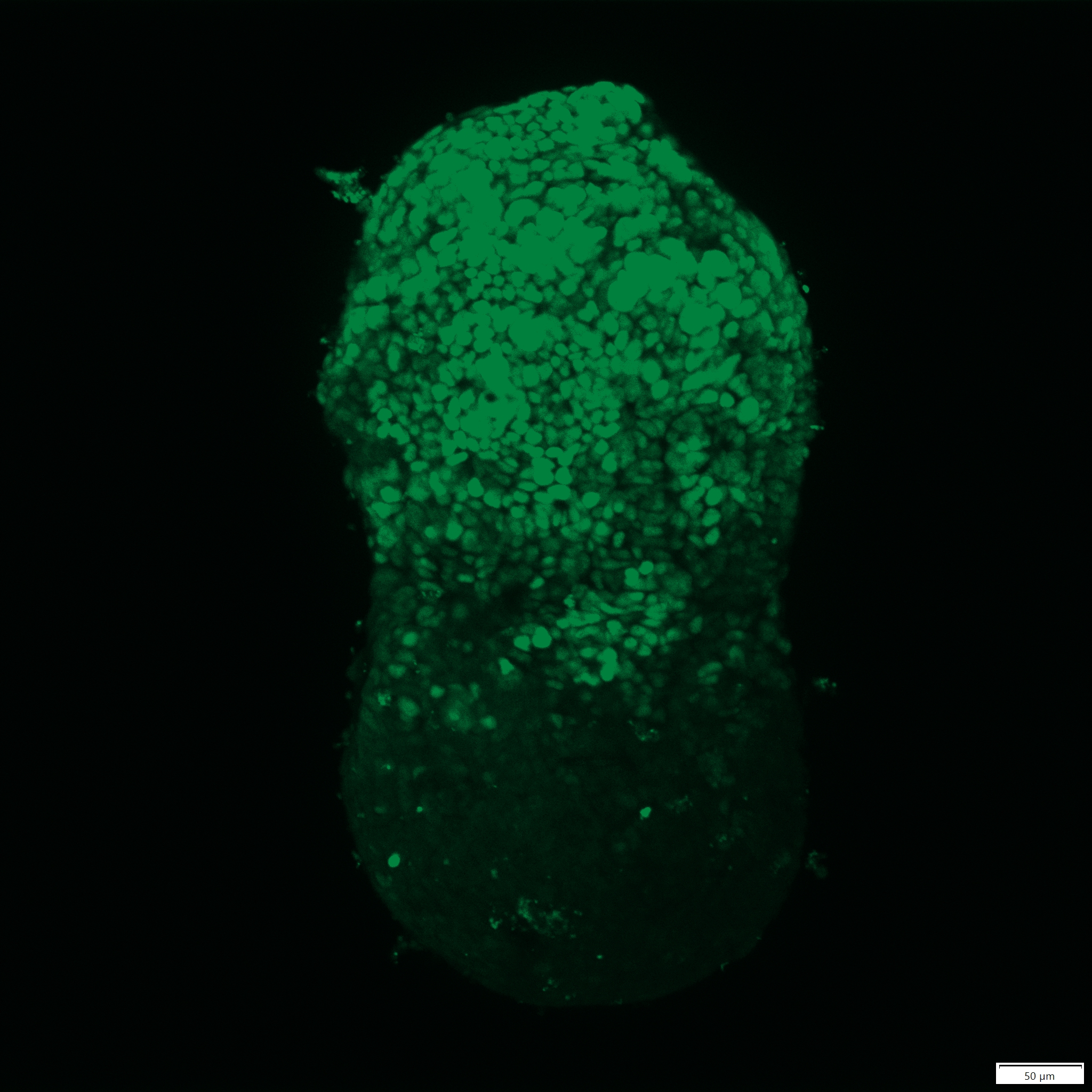

Supplement: Supplementary file 22 — Source data Fig. 6 [file 44318_2025_643_MOESM22_ESM.zip › Figure 6/6E/BMP4 EB_D3_ISH_SOX2.jpg]

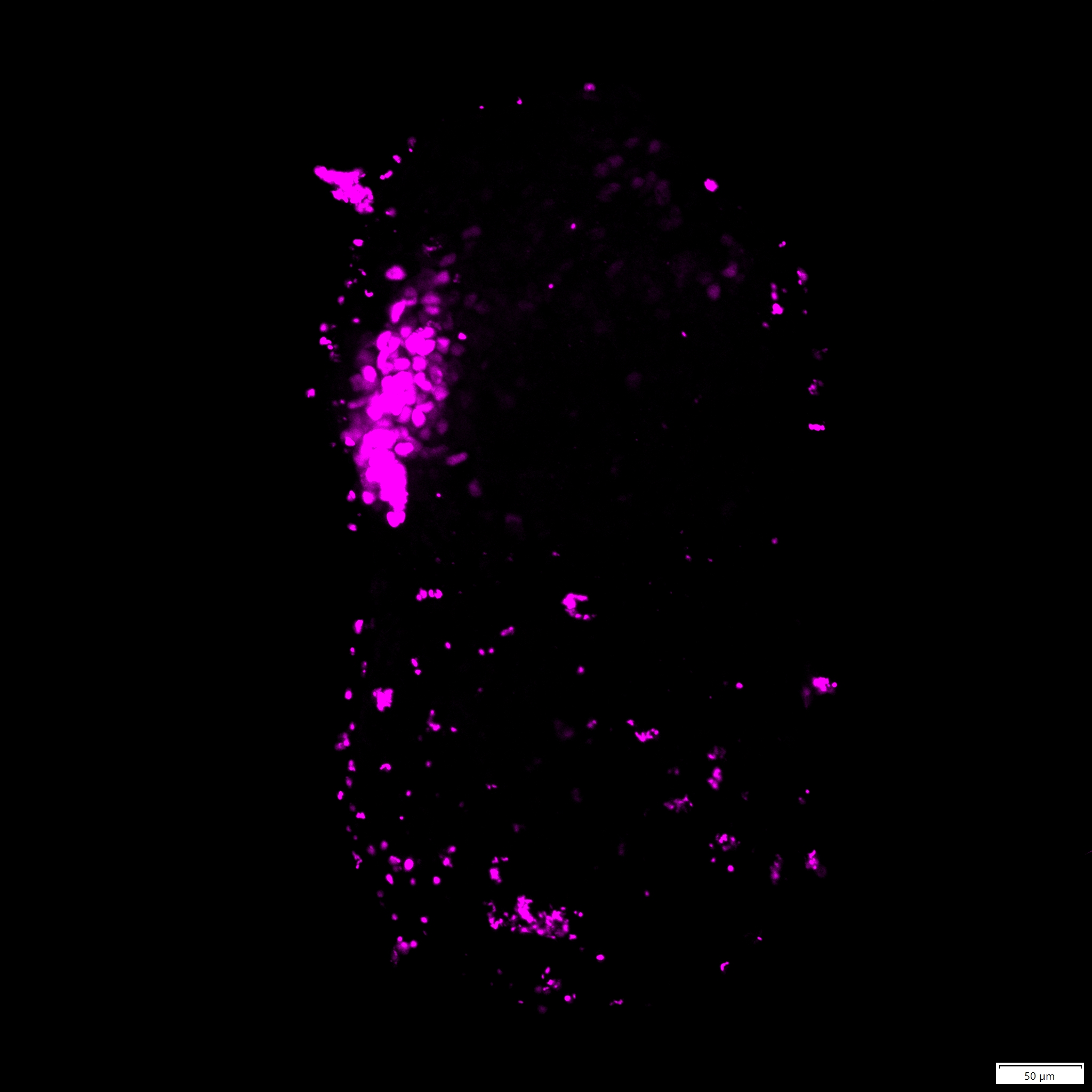

Supplement: Supplementary file 22 — Source data Fig. 6 [file 44318_2025_643_MOESM22_ESM.zip › Figure 6/6E/BMP4 EB_D3_ISH_T.jpg]

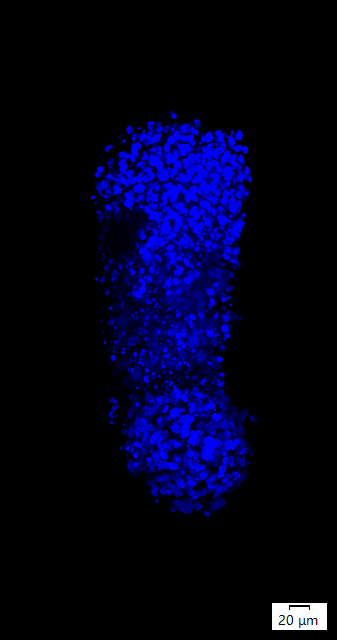

Supplement: Supplementary file 22 — Source data Fig. 6 [file 44318_2025_643_MOESM22_ESM.zip › Figure 6/6F/BMP4 EB_D5_DAPI.tif]

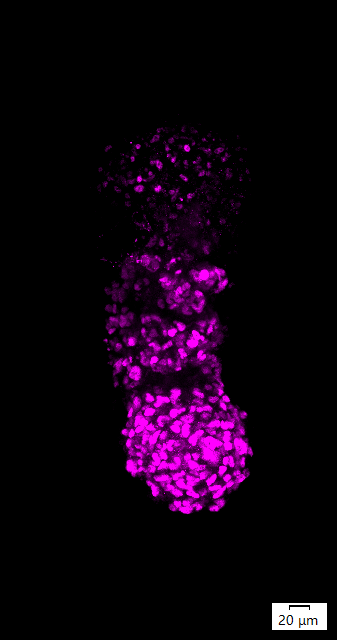

Supplement: Supplementary file 22 — Source data Fig. 6 [file 44318_2025_643_MOESM22_ESM.zip › Figure 6/6F/BMP4 EB_D5_ISH_MEOX1.tif]

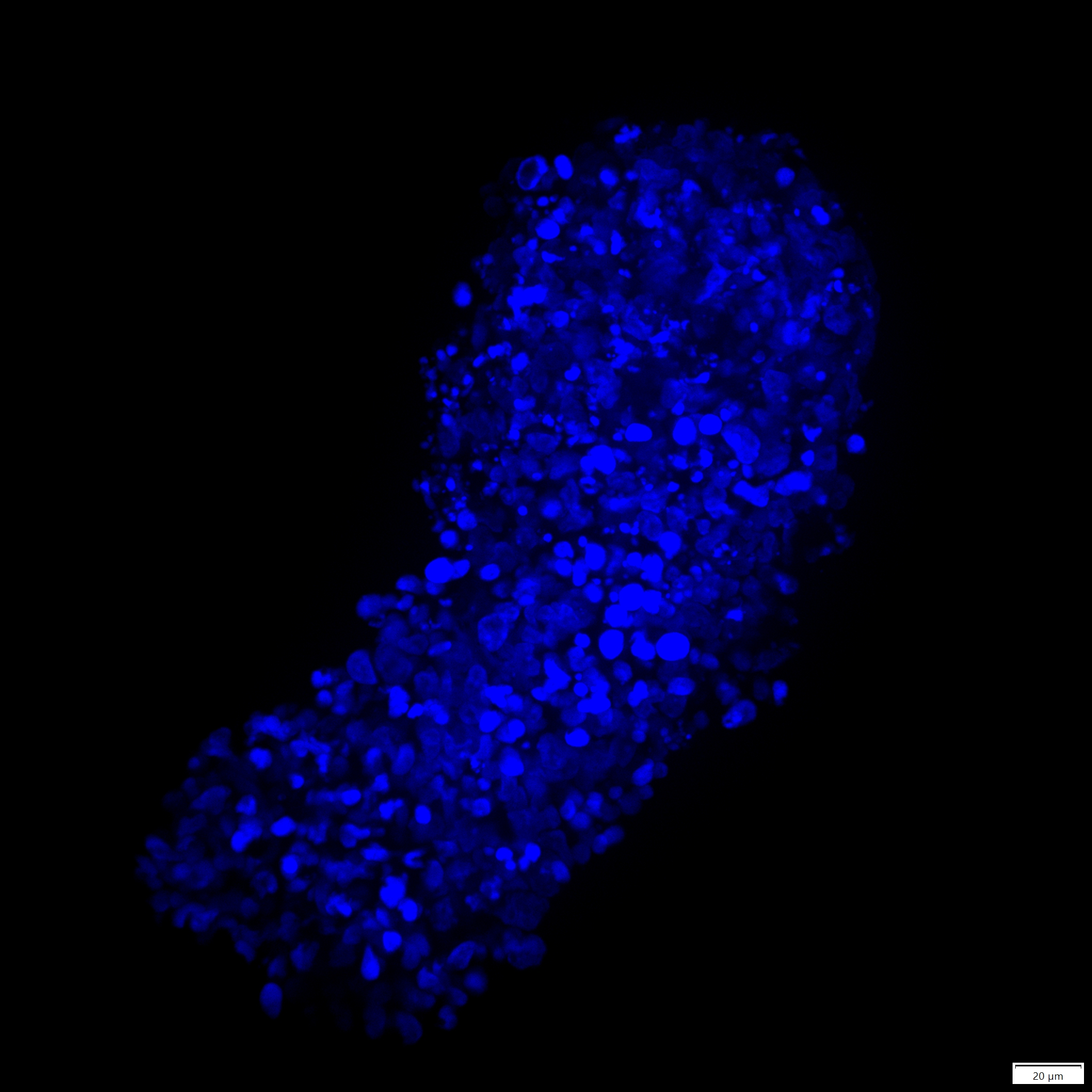

Supplement: Supplementary file 22 — Source data Fig. 6 [file 44318_2025_643_MOESM22_ESM.zip › Figure 6/6G/BMP4 EB_D5_DAPI.jpg]

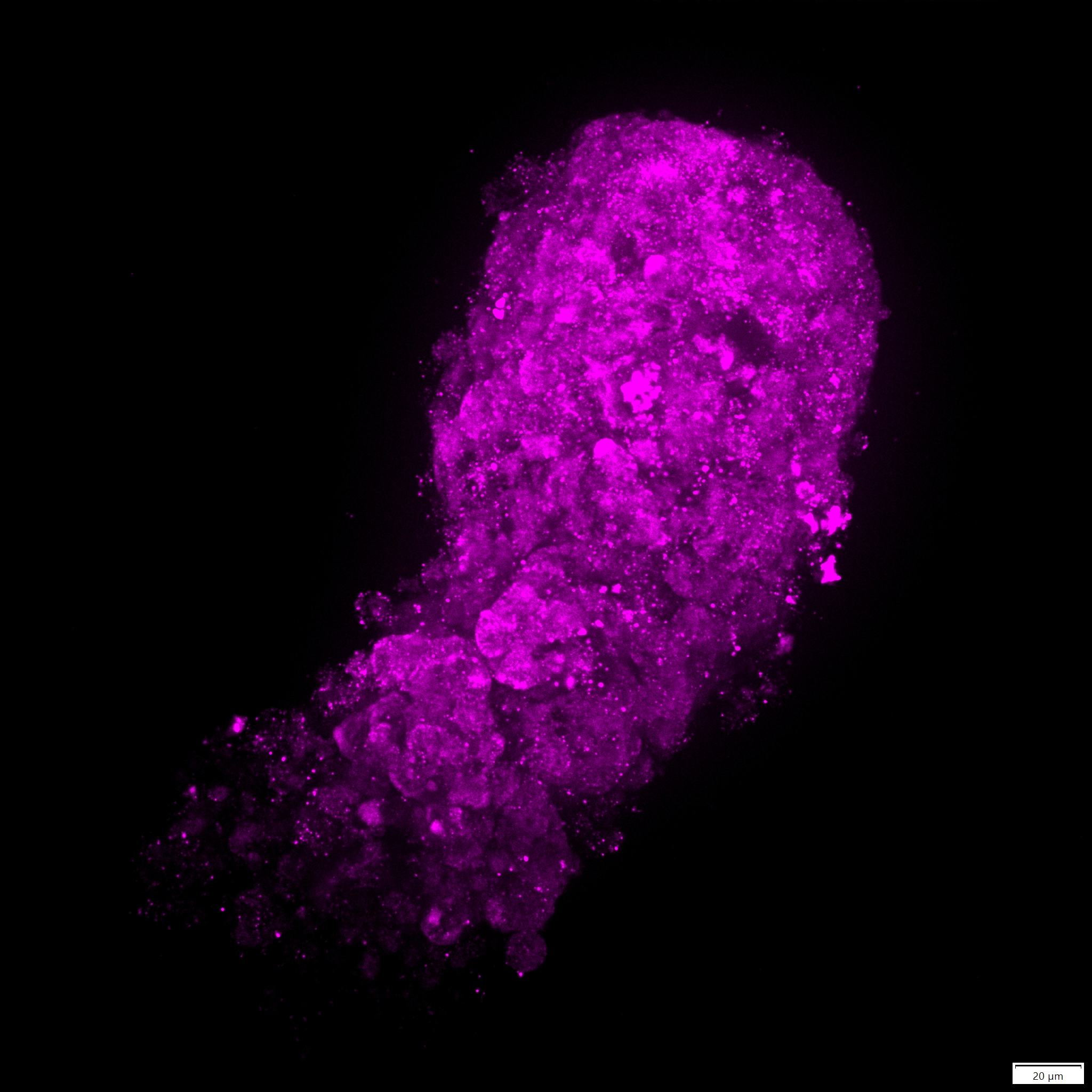

Supplement: Supplementary file 22 — Source data Fig. 6 [file 44318_2025_643_MOESM22_ESM.zip › Figure 6/6G/BMP4 EB_D5_ISH_MESP2.jpg]

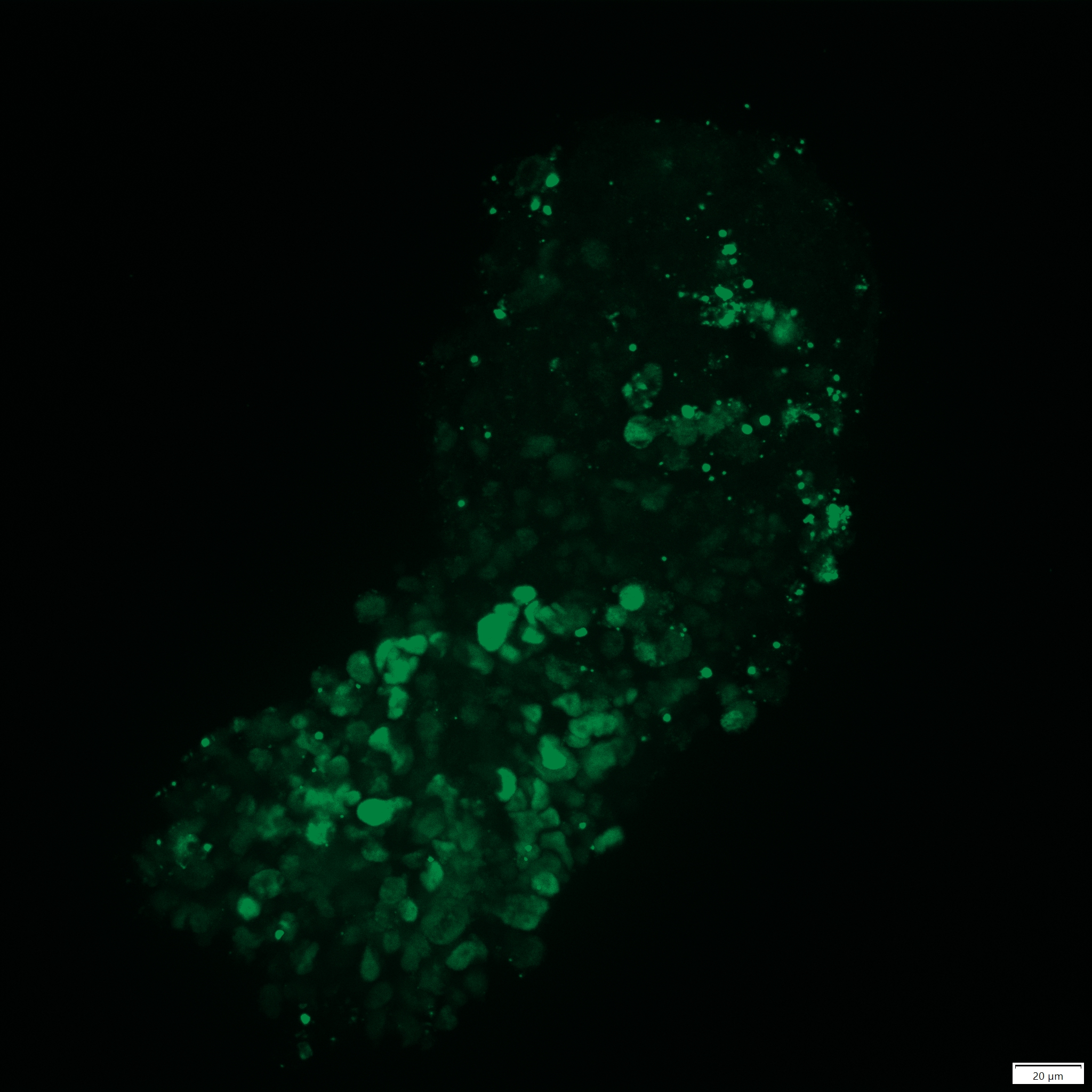

Supplement: Supplementary file 22 — Source data Fig. 6 [file 44318_2025_643_MOESM22_ESM.zip › Figure 6/6G/BMP4 EB_D5_ISH_SOX2.jpg]

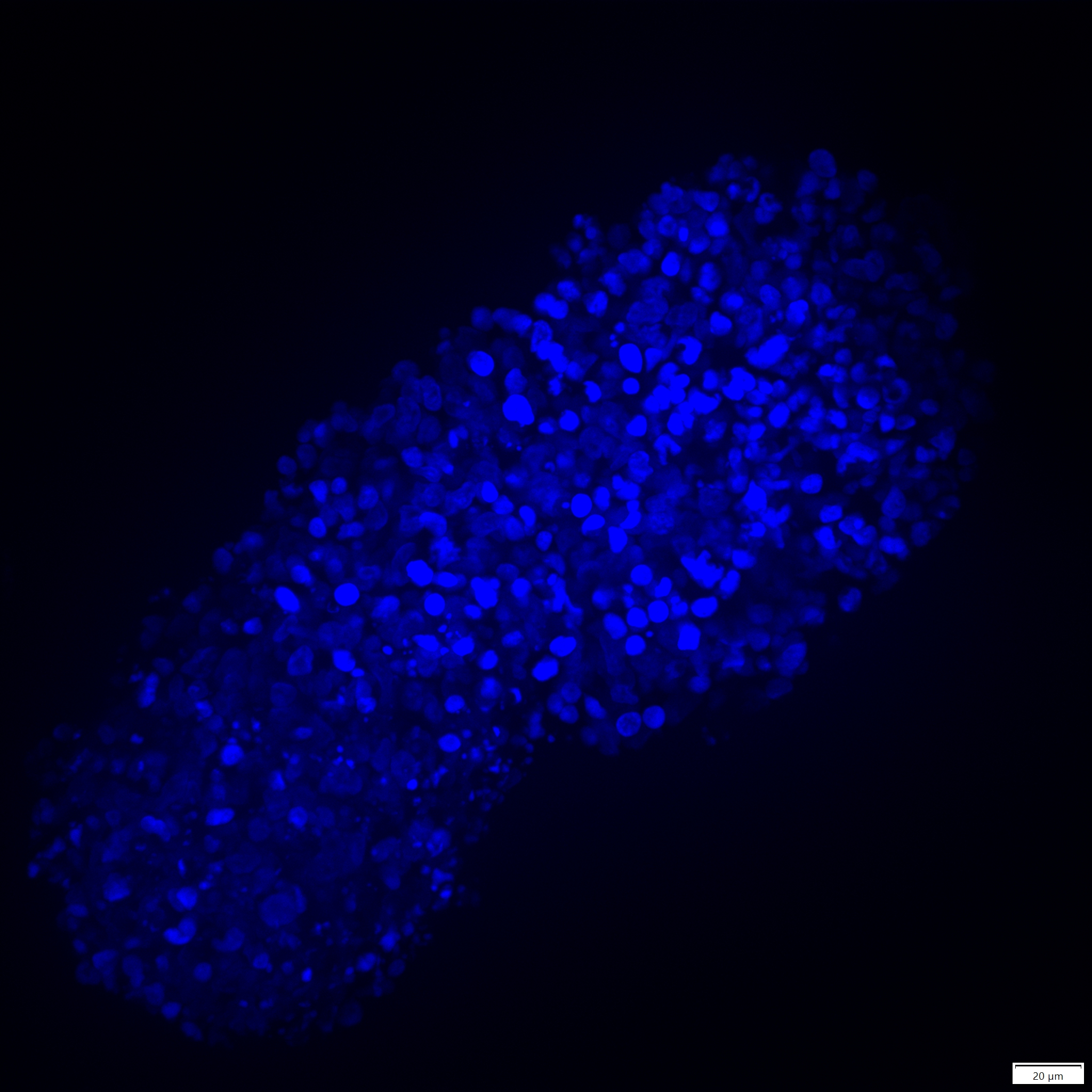

Supplement: Supplementary file 22 — Source data Fig. 6 [file 44318_2025_643_MOESM22_ESM.zip › Figure 6/6H/BMP4 EB_D5_DAPI.jpg]

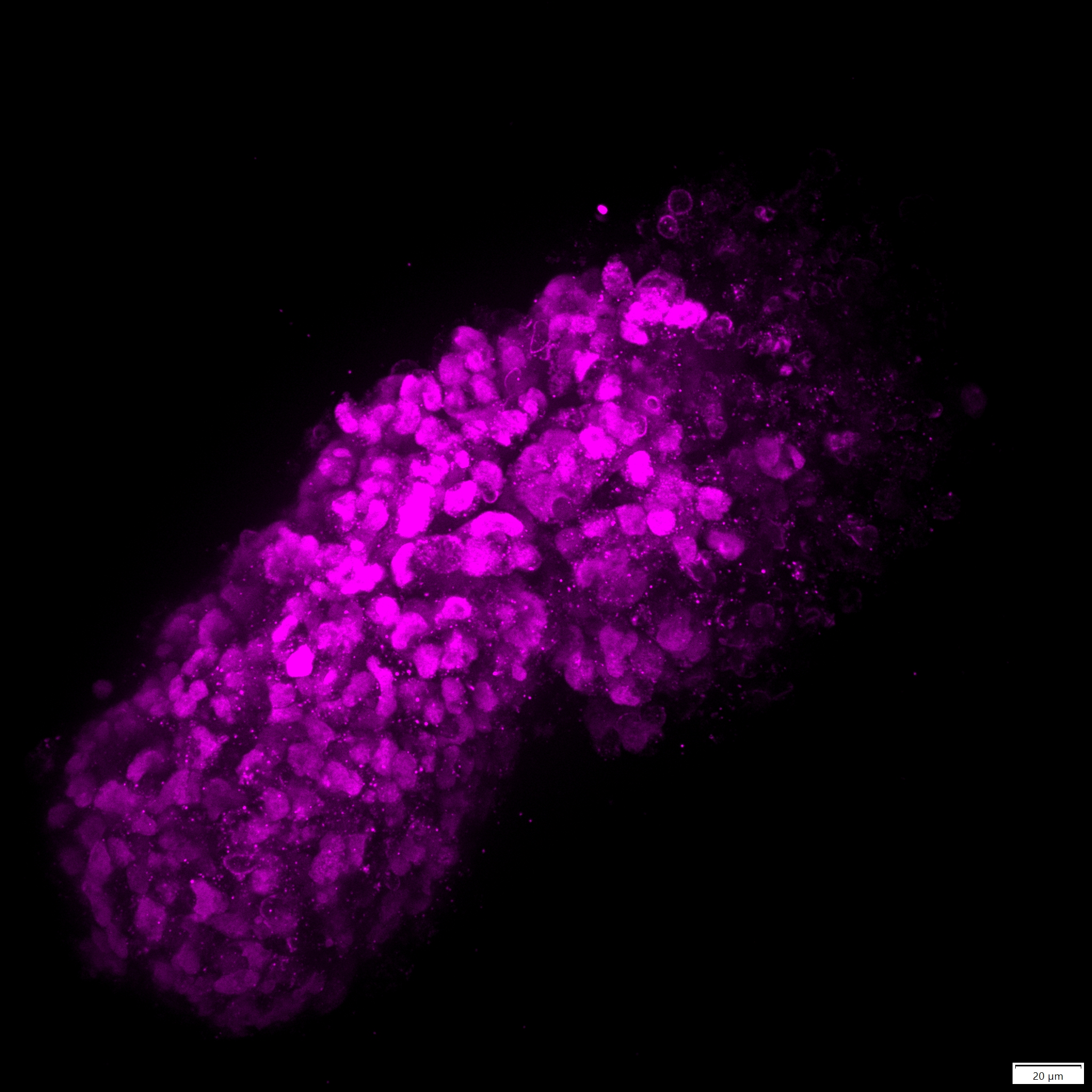

Supplement: Supplementary file 22 — Source data Fig. 6 [file 44318_2025_643_MOESM22_ESM.zip › Figure 6/6H/BMP4 EB_D5_ISH_TBX18.jpg]
