# Supplementary material for: BMP4 initiates and patterns ventral-caudal structures in zebrafish and human pluripotent stem cell aggregates
Source: EMBO J. 2025 Nov 24;45(1):210–42. doi: 10.1038/s44318-025-00643-6 (PMC12759085; doi:10.1038/s44318-025-00643-6)
Supplement: Supplementary file 23 — Expanded View Figures [file 44318_2025_643_MOESM23_ESM.pdf]

Expanded View Figures

Figure EV1. Characterization of *bmp4* injected zebrafish embryos and explants, related to Fig. 1.

(**A**) Immunofluorescence staining for phosphorylated Smad1/5/9 in embryos injected with *bmp4* + *RFP* mRNA (left) and *RFP* mRNA alone (right) at 6 hpf. The injection site was determined by red fluorescence. Signal quantifications along the ventro-dorsal axis are displayed beside the merged images. (**B**) Immunofluorescence staining for pSmad1/5/9 in explants injected with *bmp4* + *RFP* mRNA (left) and *RFP* mRNA alone (right) at 10 hpf. Signal quantifications along the anterior–posterior axis are shown next to the merged images. Clones of *bmp4* injected blastomere would stay at the injected end when they differentiated into enveloping layer cells. But when they differentiate into deep cells, they would involute and migrate anteriorly. Signal intensities of pSmad1/5/9 and RFP were normalized first by the DAPI signal and then by their mean signal intensity. (**C**) Whole mount in situ hybridization (WISH) of *id1* (left) HCR co-staining of *id1, tbxta* and *tbx6* (middle) in Bmp4 explants at 14-18 hpf, with injection sites oriented to the right. Signal quantifications along the white dashed line are presented to the right. (**D**) WISH of *eve1, fgf8a, sox32, cdx4, chrd* in Bmp4 explants at 6 hpf, with injection sites oriented to the right. (**E**) WISH of *tbxta, cdx4* in Bmp4 explants treated with Nodal inhibitor (SB505124, 50 µM), Wnt inhibitor (IWP-L6,25 µM) or FGF inhibitor (SU5402, 25 µM) at 6 hpf, with injection sites oriented to the right. Representative images of treated or untreated Bmp4 explants at 24 hpf are displayed. (**F**) WISH of *wnt8a, ndr2*, and *tbxta* in uninjected, 20 pg *bmp2b* mRNA injected, 0.5 pg *bmp4* mRNA injected explants or embryos at 6 hpf. (**G**) Expression patterns of *fgf8a*, *sox17*, and *eve1* revealed by WISH in *bmp4*-injected embryos at 6 hpf. Views from the animal pole of embryos are displayed, with descendants of the *bmp4*-injected blastomere labeled by DAB staining of GFP (left and middle images). (**H**) WISH of *sox32* in uninjected, 8 pg or 20 pg *bmp4* mRNA injected explants at 10 hpf. (**I**) Expression pattern of *tbxta* revealed by WISH in *bmp4*-injected embryos at 12 hpf. (**J**) WISH for *tbxta, egr2b, sox19a, olig2, foxd3, shha, tnnt2c, myod1, cldn3d, hoxc13b* in *bmp4*-injected embryos 24 hpf. (**K**) WISH for *egr2b, gata6, shha, her1, fn1b*, and *foxd3* in Bmp4 explants at 24 hpf. Each experiment was performed for at least three independent replicates (technical replicates). Scale bars: 100 µm.

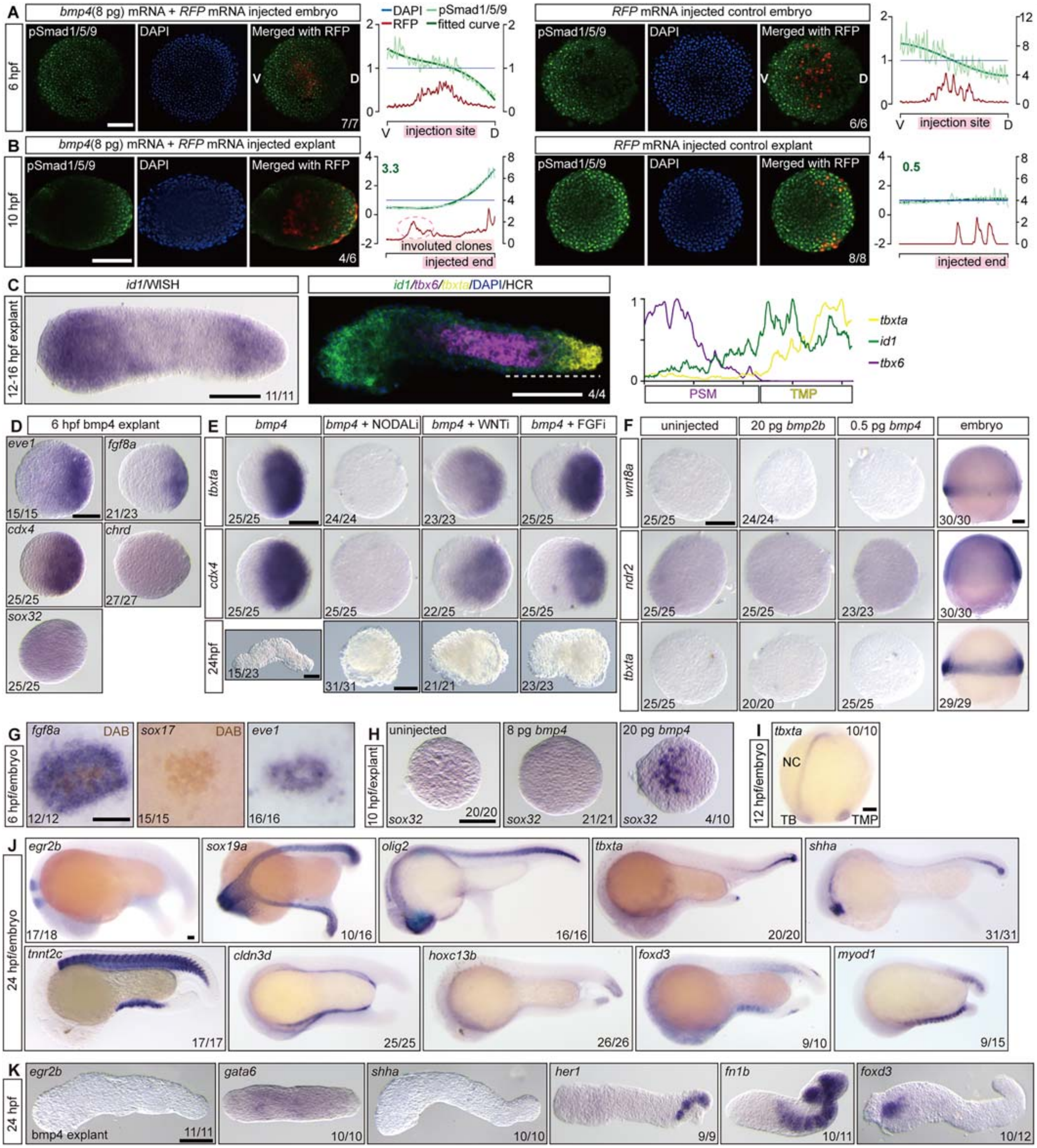
A
bmp4(8 pg) mRNA + RFP mRNA injected embryo
6 hpf
pSmad1/5/9
DAPI
Merged with RFP
V
D
7/7
DAPI
pSmad1/5/9
RFP
fitted curve
injection site
RFP mRNA injected control embryo
6/6
B
bmp4(8 pg) mRNA + RFP mRNA injected explant
10 hpf
4/6
3.3
involuted clones
injected end
RFP mRNA injected control explant
8/8
0.5
C
12-16 hpf explant
id1/WISH
11/11
id1/tbx6/tbxta/DAPI/HCR
4/4
tbxta
id1
tbx6
PSM
TMP
D
6 hpf bmp4 explant
eve1
15/15
fgf8a
21/23
cdx4
25/25
chrd
27/27
sox32
25/25
E
bmp4
bmp4 + NODALi
bmp4 + WNTi
bmp4 + FGFi
tbxta
25/25
24/24
23/23
25/25
cdx4
25/25
25/25
22/25
25/25
24hpf
15/23
31/31
21/21
23/23
F
uninjected
20 pg bmp2b
0.5 pg bmp4
embryo
wnt8a
25/25
24/24
25/25
30/30
ndr2
25/25
25/25
23/23
30/30
tbxta
25/25
20/20
25/25
29/29
G
6 hpf/embryo
fgf8a
DAB
12/12
sox17
DAB
15/15
eve1
16/16
H
10 hpf/explant
uninjected
sox32
20/20
8 pg bmp4
sox32
21/21
20 pg bmp4
sox32
4/10
I
12 hpf/embryo
tbxta
10/10
NC
TB
TMP
J
24 hpf/embryo
egr2b
17/18
sox19a
10/16
olig2
16/16
tbxta
20/20
shha
31/31
tnnt2c
17/17
cldn3d
25/25
hoxc13b
26/26
foxd3
9/10
myod1
9/15
K
24 hpf
egr2b
bmp4 explant
11/11
gata6
10/10
shha
10/10
her1
9/9
fn1b
10/11
foxd3
10/12

 © The Author(s)

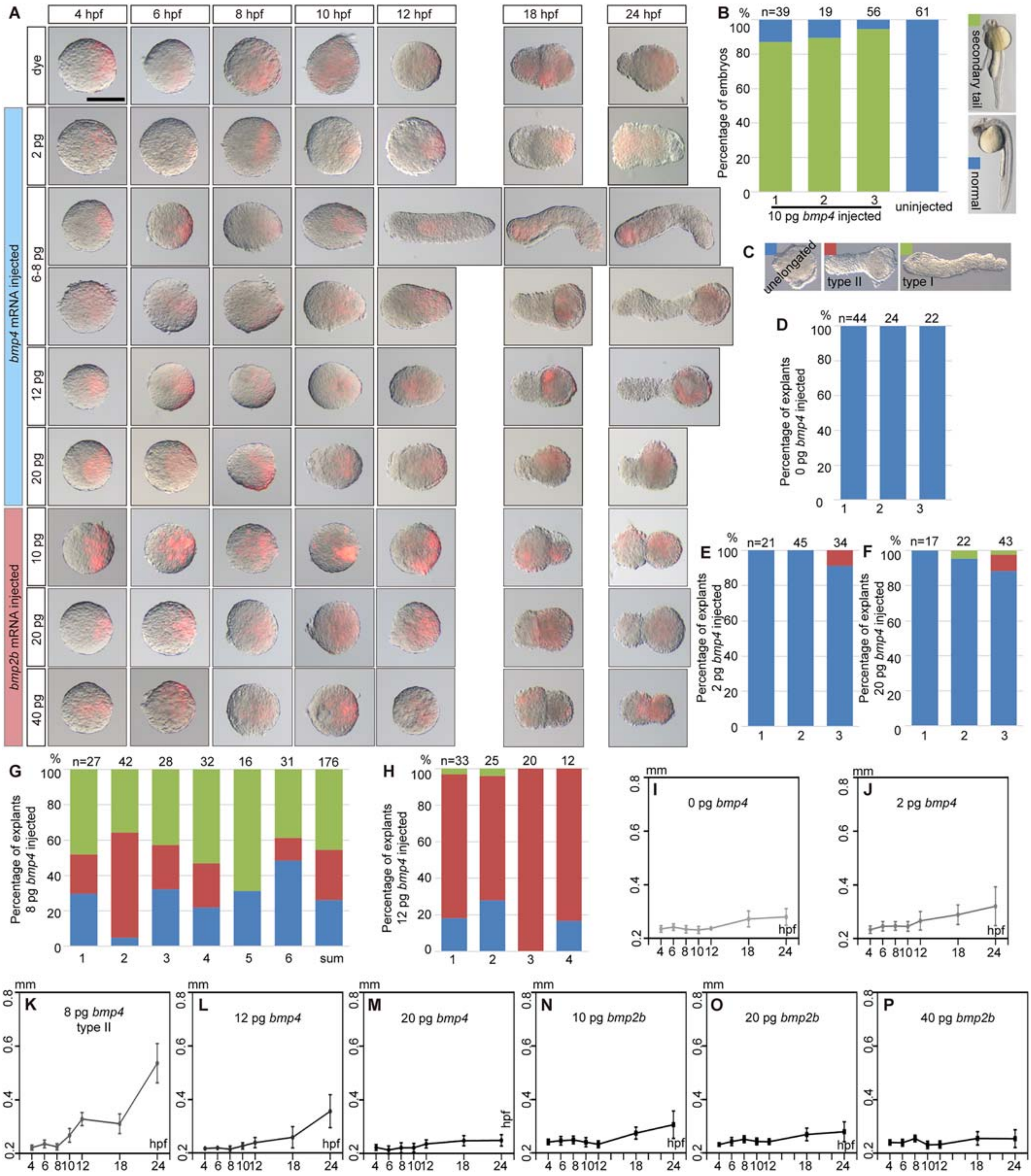
A
4 hpf
6 hpf
8 hpf
10 hpf
12 hpf
18 hpf
24 hpf
dye
bmp4 mRNA injected
2 pg
6-8 pg
12 pg
20 pg
bmp2b mRNA injected
10 pg
20 pg
40 pg
B
Percentage of embryos
n=39 19 56 61
10 pg bmp4 injected
uninjected
secondary tail
normal
C
unelongated
type II
type I
D
Percentage of explants 0 pg bmp4 injected
n=44 24 22
E
Percentage of explants 2 pg bmp4 injected
n=21 45 34
F
Percentage of explants 20 pg bmp4 injected
n=17 22 43
G
Percentage of explants 8 pg bmp4 injected
n=27 42 28 32 16 31 176
sum
H
Percentage of explants 12 pg bmp4 injected
n=33 25 20 12
I
0 pg bmp4
J
2 pg bmp4
K
8 pg bmp4 type II
L
12 pg bmp4
M
20 pg bmp4
N
10 pg bmp2b
O
20 pg bmp2b
P
40 pg bmp2b
mm
hpf

Figure EV2. Titration of optimal *bmp4* dosage for inducing caudal structures in zebrafish explants, related to Fig. 1.

(**A**) Representative images of explants induced by different doses of *bmp4* or *bmp2b* at indicated stages. The injection sites are oriented towards the right. (**B**) Statistical analysis of *bmp4* injected embryos with or without secondary tail. Analysis based on three independent replicated experiments. (**C**) Representative images showing the morphologies of *bmp4* injected explants at 24 hpf: unelongated, elongated but without somites (type II) and elongated with somites (type I) are shown. (**D–H**) Statistical analysis of different morphological outcomes (as defined in (**C**)) for explants injected with 0 pg (**D**), 2 pg (**E**), 8 pg (**F**, only type II explants analyzed), 12 pg (**G**) and 20 pg (**H**) *bmp4* mRNA. Numbers on the top of each column indicate the total number of explants analyzed. At least three independent experiments were done for each dose of *bmp4*. (**I–P**) Statistical analysis detailing the length measurements over time for explants injected with 0 pg (**I**), 2 pg (**J**), 8 pg (**K**, type II without somites), 12 pg (**L**), 20 pg (**M**) *bmp4* mRNA or 10 pg (**N**), 20 pg (**O**), 40 pg (**P**) *bmp2b* mRNA. Each experiment was performed for at least three independent replicates (technical replicates). Scale bars: 200 μm.

© The Author(s)

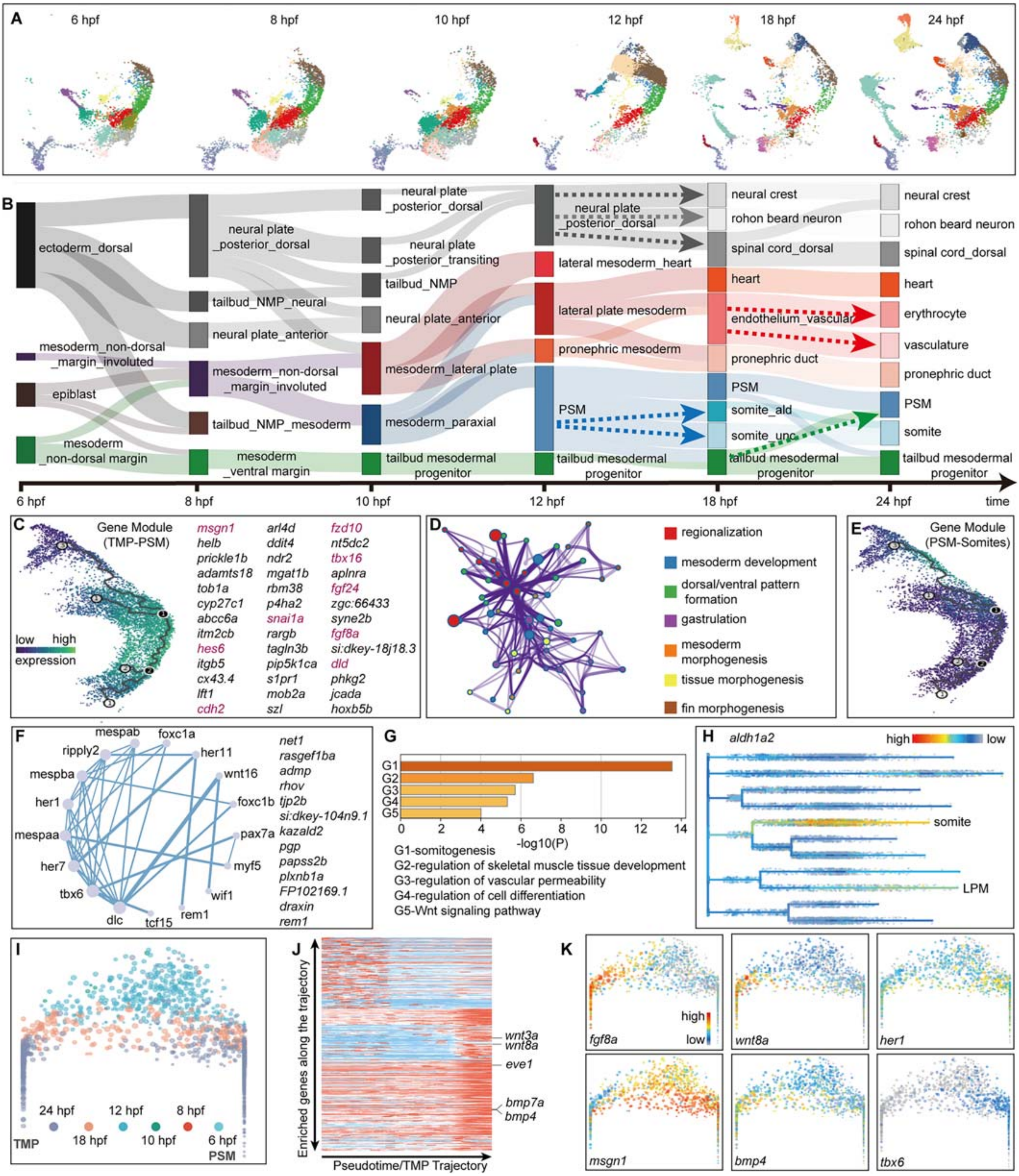
A
6 hpf
8 hpf
10 hpf
12 hpf
18 hpf
24 hpf
B
ectoderm_dorsal
mesoderm_non-dorsal _margin_involuted
epiblast
mesoderm _non-dorsal margin
neural plate _posterior_dorsal
tailbud_NMP_neural
neural plate_anterior
mesoderm_non-dorsal _margin_involuted
tailbud_NMP_mesoderm
mesoderm _ventral margin
neural plate _posterior_dorsal
neural plate _posterior_transiting
tailbud_NMP
neural plate_anterior
mesoderm_lateral plate
mesoderm_paraxial
tailbud mesodermal progenitor
neural plate _posterior_dorsal
lateral mesoderm_heart
lateral plate mesoderm
pronephric mesoderm
PSM
tailbud mesodermal progenitor
neural crest
rohon beard neuron
spinal cord_dorsal
heart
endothelium_vascular
pronephric duct
PSM
somite_ald
somite_unc
tailbud mesodermal progenitor
neural crest
rohon beard neuron
spinal cord_dorsal
heart
erythrocyte
vasculature
pronephric duct
PSM
somite
tailbud mesodermal progenitor
6 hpf
8 hpf
10 hpf
12 hpf
18 hpf
24 hpf
time
C
Gene Module (TMP-PSM)
low high expression
msgn1 helb prickle1b adamts18 tob1a cyp27c1 abcc6a itm2cb hes6 itgb5 cx43.4 lft1 cdh2
arl4d ddit4 ndr2 mgat1b rbm38 p4ha2 snai1a rargb tagln3b pip5k1ca s1pr1 mob2a szl
fzd10 nt5dc2 tbx16 aplnra fgf24 zgc:66433 syne2b fgf8a si:dkey-18j18.3 dld phkg2 jcada hoxb5b
D
regionalization
mesoderm development
dorsal/ventral pattern formation
gastrulation
mesoderm morphogenesis
tissue morphogenesis
fin morphogenesis
E
Gene Module (PSM-Somites)
F
mespab
foxc1a
ripply2
her11
mespba
wnt16
her1
foxc1b
mespaa
pax7a
her7
myf5
tbx6
wif1
dlc
tcf15
rem1
net1 rasgef1ba admp rhov tjp2b si:dkey-104n9.1 kazald2 pgp papss2b plxnb1a FP102169.1 draxin rem1
G
G1
G2
G3
G4
G5
0 2 4 6 8 10 12 14
-log10(P)
G1-somitogenesis
G2-regulation of skeletal muscle tissue development
G3-regulation of vascular permeability
G4-regulation of cell differentiation
G5-Wnt signaling pathway
H
aldh1a2
high low
somite
LPM
I
24 hpf
12 hpf
8 hpf
18 hpf
10 hpf
6 hpf
TMP
PSM
J
Enriched genes along the trajectory
wnt3a
wnt8a
eve1
bmp7a
bmp4
Pseudotime/TMP Trajectory
K
high
low
fgf8a
wnt8a
her1
msgn1
bmp4
tbx6

Figure EV3. Single-cell RNA sequencing analysis of Bmp4 explant, related to Fig. 2.

(**A**) UMAP plot showing the cell clusters in Bmp4 explants at 6 developmental stages (6 hpf, 8 hpf, 10 hpf, 12 hpf, 18 hpf and 24 hpf). Cells are colored by different cell types. Schematic of Bmp4 explants at indicated developmental stage are shown on the top. (**B**) Sankey plot showing inferred relationships between cell states across the development of Bmp4 explant. Gray, blue, green and red arrows highlight the differentiation of neural plate to neural crest/neural tube/Rohan beard neuron, PSM to somites, TMP to PSM and vascular endothelium to vasculature/erythrocyte, respectively. (**C**) The single-cell trajectory of TMP, PSM and Somites was analyzed using Monocle 3 and visualized by UMAP. Cells are color-coded based on the expression levels of gene module of TMP-PSM, which represents the co-upregulated genes in the trajectory from TMP to PSM. The genes in this module are listed on the right, and genes highlighted in purple indicate that they have been reported to be relevant to somite formation. (**D**) Network layout displays the enriched Gene Ontology (GO) terms for gene module of TMP-PSM (**C**). The size of each node is scaled according to the number of input genes associated with that term. The color of the nodes corresponds to their cluster identity, which is labeled on the right side. The terms are connected by edges, where the thickness of the edge indicates the similarity score (score > 0.3) between the terms. (**E**) The single-cell trajectory of TMP, PSM and Somites. Cells are color-coded based on the expression levels of gene module of PSM-Somites. (**F**) The genes in the gene module of PSM-Somites. Several genes reported to show interactions are displayed in a circular network layout (left). (**G**) Heatmap showing the enriched GO terms using the genes from (**F**). (**H**) URD trajectory of Bmp4 explant showing the expression of *aldh1a2*, somite and LPM were labeled. (**I**) URD branchpoint plots of TMP and PSM development are generated using scRNA-seq data from Bmp4 explants. The plots depict pseudotime on the y-axis and the random walk visitation preference from TMP to PSM domains on the x-axis. Cells are colored by developmental stages. (**J**) Heatmap showing the expression of enriched genes along the TMP trajectory (pseudotime, x-axis). Several genes related to the TMP, including *wnt3a*, *wnt8a*, *eve1*, *bmp7a*, and *bmp4*, are labeled on the right. (**K**) The expressions of *fgf8a*, *wnt8a*, *msgn1*, *bmp4*, *her1* and *tbx6* are showed on URD branchpoint plots of TMP and PSM.

 © The Author(s)

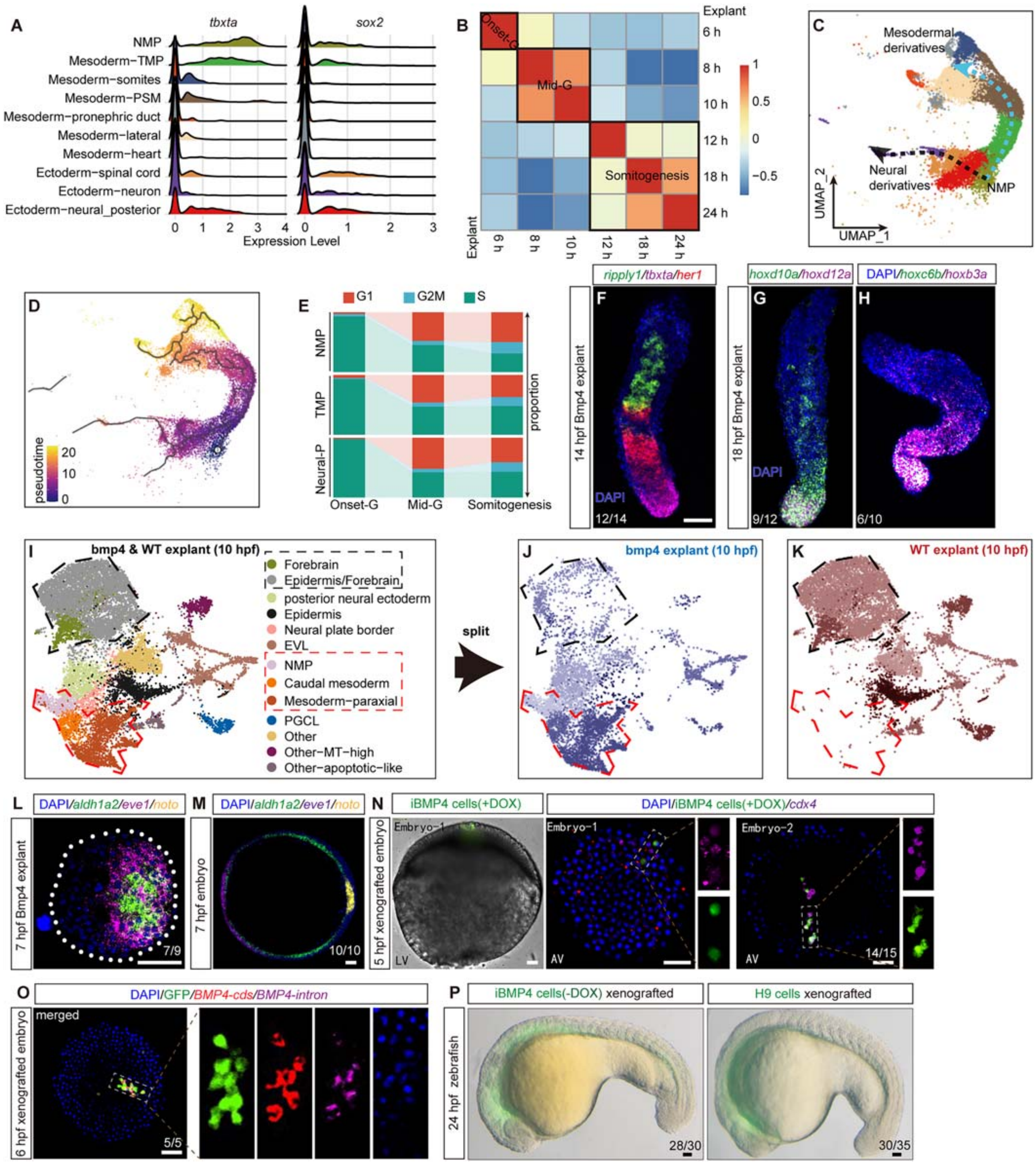
A
tbxta
sox2
NMP
Mesoderm−TMP
Mesoderm−somites
Mesoderm−PSM
Mesoderm−pronephric duct
Mesoderm−lateral
Mesoderm−heart
Ectoderm−spinal cord
Ectoderm−neuron
Ectoderm−neural_posterior
Expression Level
B
Explant
Onset-G
Mid-G
Somitogenesis
6 h
8 h
10 h
12 h
18 h
24 h
C
Mesodermal derivatives
Neural derivatives
NMP
UMAP_1
UMAP_2
D
pseudotime
E
G1
G2M
S
NMP
TMP
Neural-P
proportion
Onset-G
Mid-G
Somitogenesis
ripply1/tbxta/her1
hoxd10a/hoxd12a
DAPI/hoxc6b/hoxb3a
14 hpf Bmp4 explant
18 hpf Bmp4 explant
DAPI
12/14
9/12
6/10
I
bmp4 & WT explant (10 hpf)
Forebrain
Epidermis/Forebrain
posterior neural ectoderm
Epidermis
Neural plate border
EVL
NMP
Caudal mesoderm
Mesoderm−paraxial
PGCL
Other
Other−MT−high
Other−apoptotic−like
split
J
bmp4 explant (10 hpf)
K
WT explant (10 hpf)
L
DAPI/aldh1a2/eve1/noto
7 hpf Bmp4 explant
7/9
M
DAPI/aldh1a2/eve1/noto
7 hpf embryo
10/10
N
iBMP4 cells(+DOX)
DAPI/iBMP4 cells(+DOX)/cdx4
5 hpf xenografted embryo
Embryo-1
Embryo-2
LV
AV
14/15
O
DAPI/GFP/BMP4-cds/BMP4-intron
6 hpf xenografted embryo
merged
5/5
P
iBMP4 cells(-DOX) xenografted
H9 cells xenografted
24 hpf zebrafish
28/30
30/35

Figure EV4. Analyzing NMP-like development in Bmp4 explant, related to Fig. 2.

(**A**) Ridge plot showing the expression of *tbxta* and *sox2* in each cell cluster (including NMP-like, mesodermal derivatives and neural derivatives). (**B**) Heatmap showing the transcriptional similarity between Bmp4 explants at 6 developmental timepoints. We defined three developmental stages: Onset-G (6 hpf), Mid-G (8–10 hpf) and somitogenesis (12–24 hpf). (**C**) UMAP plot displaying cell clusters related to NMP-like, mesodermal derivatives, and neural derivatives. The blue dashed line with an arrow indicates the potential differentiation trajectory from NMP-like to mesodermal derivatives. The black dashed line with an arrow indicates potential differentiation trajectory from NMP-like to neural derivatives. (**D**) UMAP plot showing the inferred trajectories from NMP-like cells to mesodermal derivatives or neural derivatives. Cells are colored by pseudotime. (**E**) Ribbon plot showing the proportions of the computational cell cycle stages (G1, G2M and S) in NMP-like, TMP and Neural-P cells during development. (**F–H**) HCR co-staining of *ripply1*, *tbxta* and *her1* (**F**), *hoxd10a* and *hoxd12a* (**G**), *hoxc6b* and *hoxb3a* (**H**) in Bmp4 explants at 14 hpf (**F**),18 hpf (**G**, **H**). DAPI was co-stained (**F–H**). (**I**) UMAP plots showing integrated single-cell RNA sequencing (scRNA-seq) datasets of Bmp4 explants and WT uninjected explants at 10 hpf (**I**). (**J, K**) UMAP plots showing scRNA-seq datasets of Bmp4 explants (**J**) or WT uninjected explants (**K**) separated from I. Red and black dashed circle indicate caudal cell fates and anterior neural cell fates respectively. (**L**, **M**) HCR co-staining of *aldh1a2*, *eve1* and *noto* in Bmp4 explants (**L**) and zebrafish embryos (**M**) at 7 hpf. (**N**) HCR staining of *cdx4* in xenografted zebrafish embryos at 5 hpf. Green fluorescence indicates grafted iBMP4-induced human PSCs. Representative merged images depicting the location of grafted iBMP4-induced human PSCs in a zebrafish embryo at 5 hpf. The left panel (lateral view) shows the graft site, while the middle panel (animal pole view) of the same embryo reveals the expression of *cdx4* (magenta) in relation to the grafted human cells (green). (**O**) HCR staining of BMP4-cds and BMP4-intron in xenografted zebrafish embryos at 6 hpf. Grafted human cells are labeled with GFP. (**P**) Merged images showing untreated iBMP4 cells grafted (left, labeled with GFP) or H9 grafted (right, labeled with RFP) zebrafish larvae at 24 hpf. Each experiment was performed for at least three independent replicates (technical replicates). Scale bar: 50 µm.

 © The Author(s)

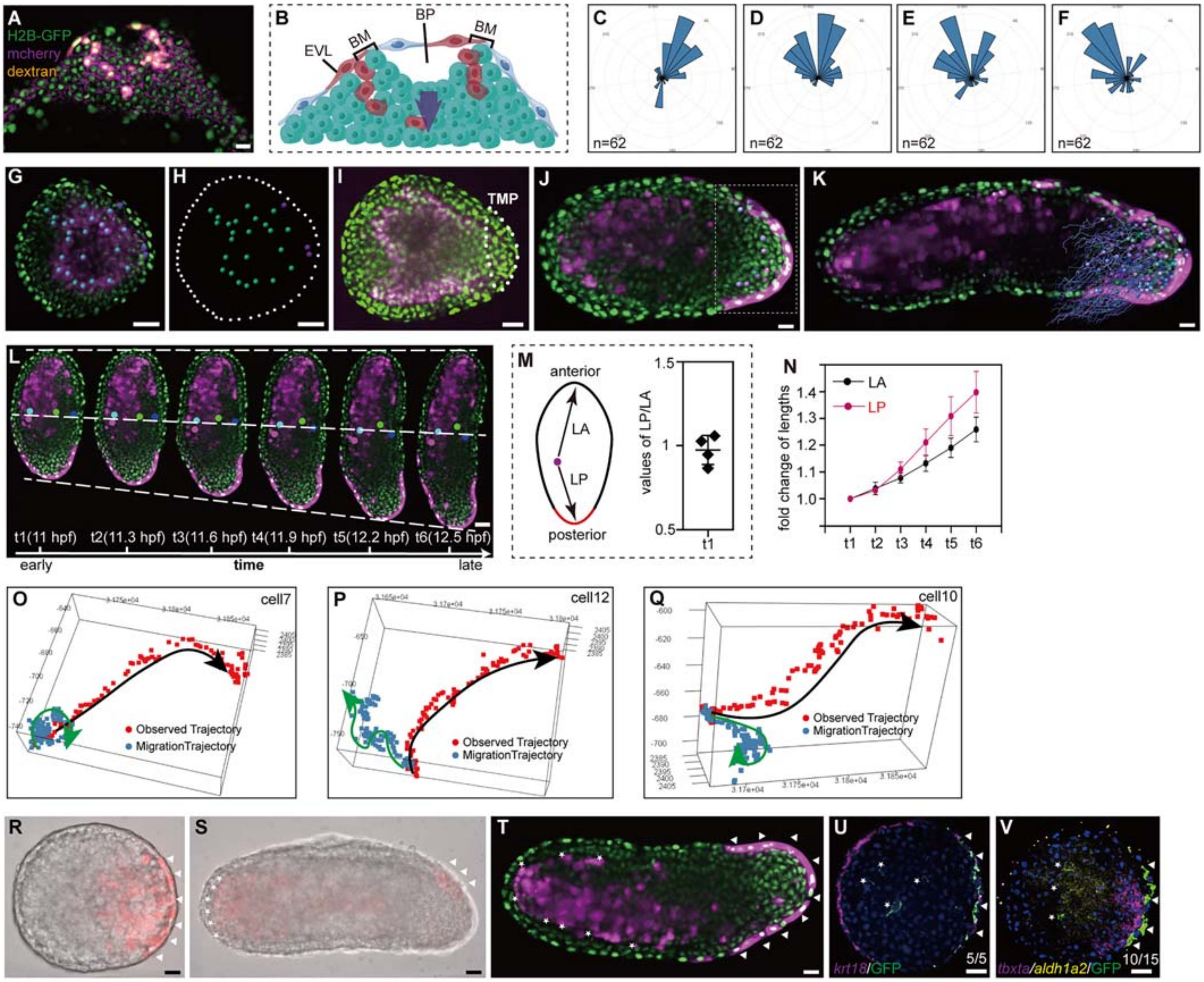

Figure EV5. Cell movements in *bmp4* injected embryos and explants, related to Fig. 4.

(**A**) Confocal image of the protrusion in a *bmp4* injected embryo at 8 hpf. Cell nuclei, cell membrane and descendants of *bmp4* mRNA injected blastomere are labeled by H2B-GFP, mcherry and dextran fluorescein(purple), respectively. (**B**) Schematic summary of structures near *bmp4* injection site in the embryo, based on (**A**). (**C–F**) Rose-plots show the movement direction of internalizing cells at different timepoints, from early (**C**) to late (**F**) timepoints. (**G–I**) Representative images of gastrulation cell movements in a Bmp4 explant generated from Tg (*aldh1a2: H2B-RFP*) transgenic line at the middle (**G**, **H**) and end (**I**) of gastrulation, according to Movies EV6 and EV7. Cells are colored based on the conditions of their movement, as described in Fig. 4B′. Dashed lines outline the boundaries of the embryonic blastoderm (**H**) and explant (**I**). (**H**) Cell movement trajectories in the posterior part of Bmp4 explant (dashed rectangle region) are analyzed at the beginning of segmentation stage. (**I**) Cell movement trajectories of all 13 tracked cells. (**J–L**) Comparison of observed raw trajectories (red spots and black arrows) to the computed migration trajectories (green spots and green arrows). These trajectories illustrate cell movements within the Bmp4 explant, with details on the trajectory analysis strategy outlined in Fig. 4H. Scale bars: 20 µm. (**M**) Measurement of distances from one spot to the anterior or posterior end of the explant, denoted as LA or PA respectively. It is noteworthy that the blastomere injected with bmp4 mRNA contributes both to EVL cells that stay at the injection site and deep cells that migrate to the anterior later. So, we use the red fluorescence labeled EVL cells to determine the injection site and posterior end. The values of LP divided by LA are all around 1, indicating central positioning of the tracked spots. (**N**) Quantification of the elongation of the anterior and posterior halves of Bmp4 explant over time, with lengths of LP/LA at times t2-6 normalized to those at t1. (**O–Q**) The observed raw trajectories (red spots and black arrows) compared to the migration trajectories (cell movements within Bmp4 explant, green spots and green arrows) obtained by the strategy described (details see methods) in Fig. 4H of cell 7 (**O**), 12 (**P**), 10 (**Q**). (**R**, **S**) Merged images showing the morphology and distributions of *bmp4* injected cell clones in Bmp4 explant at 5 hpf (**R**) and 12 hpf (**S**). White triangles indicate the EVL cells. (**T**) Merged image showing the morphology and *bmp4* injected cell clones in Bmp4 explant at 12 hpf. GFP signal: nucleus (H2B-GFP), RFP signal: *bmp4* injected clones. White triangles indicate the EVL cells. (**U**, **V**) HCR staining of *krt18* (**U**), *tbxta*&*aldh1a2* (**V**) in Bmp4 explants at 9 hpf. GFP signals indicate the *bmp4* injected clones. White triangles indicate the EVL cells, white stars indicate the internalized *bmp4* injected clones. Each experiment was performed for at least three independent replicates (technical replicates). Scale bar: 50 µm (**G**, **H**, **L**), 20 µm (**A**, **I**, **J**, **K**, **R–V**).

© The Author(s)

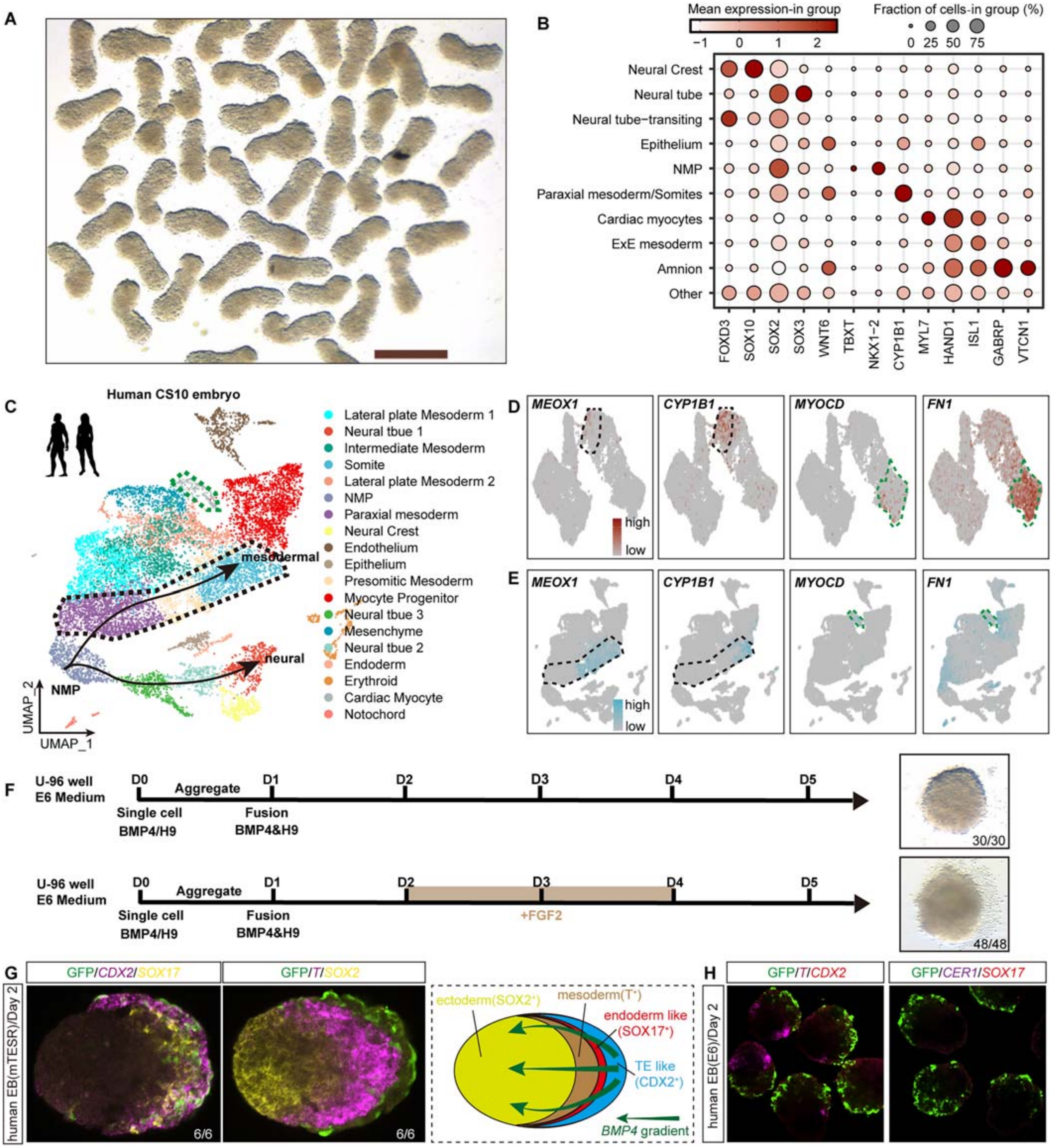
A
B
Mean expression-in group
−1 0 1 2
Fraction of cells-in group (%)
0 25 50 75
Neural Crest
Neural tube
Neural tube−transiting
Epithelium
NMP
Paraxial mesoderm/Somites
Cardiac myocytes
ExE mesoderm
Amnion
Other
FOXD3
SOX10
SOX2
SOX3
WNT6
TBXT
NKX1−2
CYP1B1
MYL7
HAND1
ISL1
GABRP
VTCN1
C
Human CS10 embryo
mesodermal
neural
NMP
UMAP_2
UMAP_1
Lateral plate Mesoderm 1
Neural tbue 1
Intermediate Mesoderm
Somite
Lateral plate Mesoderm 2
NMP
Paraxial mesoderm
Neural Crest
Endothelium
Epithelium
Presomitic Mesoderm
Myocyte Progenitor
Neural tbue 3
Mesenchyme
Neural tbue 2
Endoderm
Erythroid
Cardiac Myocyte
Notochord
D
MEOX1
CYP1B1
MYOCD
FN1
high
low
E
MEOX1
CYP1B1
MYOCD
FN1
high
low
F
U-96 well
E6 Medium
D0
Aggregate
D1
D2
D3
D4
D5
Single cell
BMP4/H9
Fusion
BMP4&H9
30/30
U-96 well
E6 Medium
D0
Aggregate
D1
D2
D3
D4
D5
Single cell
BMP4/H9
Fusion
BMP4&H9
+FGF2
48/48
G
GFP/CDX2/SOX17
GFP/T/SOX2
human EB(mTESR)/Day 2
6/6
6/6
ectoderm(SOX2+)
mesoderm(T+)
endoderm like
(SOX17+)
TE like
(CDX2+)
BMP4 gradient
H
GFP/T/CDX2
GFP/CER1/SOX17
human EB(E6)/Day 2

© The Author(s)

Figure EV6. scRNA-seq analysis of human VCLS at day 5, related to Fig. 6.

(**A**) Bright field image of VCLS at day 5, illustrating typical morphology. (**B**) Dot plot showing the average expression levels of the specified marker genes in each cell cluster. (**C**) UMAP plot showing the cell clusters in human CS10 embryo, with cells colored by different cell types. Black lines with arrows highlight the potential differentiation trajectories from NMP-like cells to neural or mesodermal lineages. (**D**) UMAP plots showing the expression patterns of *MEOX1*, *CYP1B1*, *MYOCD* and *FN1* in VCLS at day 5. These genes are key markers for paraxial mesoderm/somites and cardiac myocytes, which are highlighted with black and green circles, respectively. (**E**) Comparative UMAP plots showing the expression of *MEOX1*, *CYP1B1*, *MYOCD* and *FN1* in human CS10 embryo. (**F**) Bright field images of control human embryoid at day 5 induced without dox and FGF2 (upper panel) or only with FGF2 (lower panel). (**G**) HCR co-staining of *CDX2/SOX17*(left) and *T/SOX2*(middle) in EBs cultured in mTeSR1 at day 2. GFP indicates the location of *BMP4*-producing cells. The pattern of different germ layers is summarized to the right. (**H**) HCR co-staining of *CDX2/T*(left) and *CER1/SOX17*(middle) in EBs cultured in E6 at day 2. GFP indicates the location of *BMP4*-producing cells. Each experiment was performed for at least three independent replicates (technical replicates). Scale bars: 500 μm.

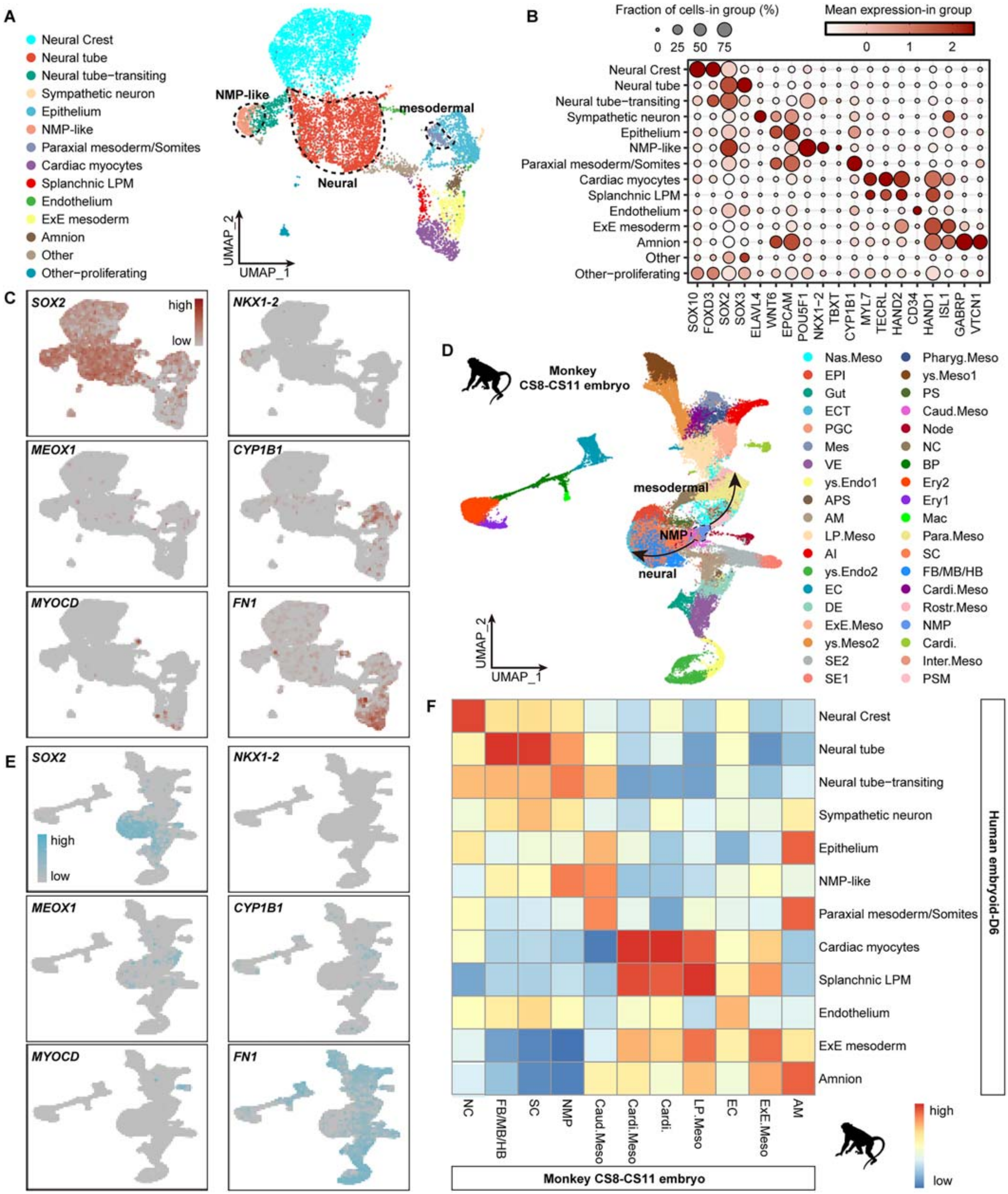
A
Neural Crest
Neural tube
Neural tube−transiting
Sympathetic neuron
Epithelium
NMP-like
Paraxial mesoderm/Somites
Cardiac myocytes
Splanchnic LPM
Endothelium
ExE mesoderm
Amnion
Other
Other−proliferating
NMP-like
mesodermal
Neural
UMAP_2
UMAP_1
B
Fraction of cells-in group (%)
0 25 50 75
Mean expression-in group
0 1 2
SOX10 FOXD3 SOX2 SOX3 ELAVL4 WNT6 EPCAM POU5F1 NKX1-2 TBXT CYP1B1 MYL7 TECRL HAND2 CD34 HAND1 ISL1 GABRP VTCN1
C
SOX2
high
low
NKX1-2
MEOX1
CYP1B1
MYOCD
FN1
D
Monkey
CS8-CS11 embryo
mesodermal
NMP
neural
Nas.Meso
EPI
Gut
ECT
PGC
Mes
VE
ys.Endo1
APS
AM
LP.Meso
AI
ys.Endo2
EC
DE
ExE.Meso
ys.Meso2
SE2
SE1
Pharyg.Meso
ys.Meso1
PS
Caud.Meso
Node
NC
BP
Ery2
Ery1
Mac
Para.Meso
SC
FB/MB/HB
Cardi.Meso
Rostr.Meso
NMP
Cardi.
Inter.Meso
PSM
E
SOX2
high
low
NKX1-2
MEOX1
CYP1B1
MYOCD
FN1
F
Neural Crest
Neural tube
Neural tube−transiting
Sympathetic neuron
Epithelium
NMP-like
Paraxial mesoderm/Somites
Cardiac myocytes
Splanchnic LPM
Endothelium
ExE mesoderm
Amnion
Human embryoid-D6
NC FB/MB/HB SC NMP Caud.Meso Cardi.Meso Cardi. LP.Meso EC ExE.Meso AM
Monkey CS8-CS11 embryo
high
low

© The Author(s)

Figure EV7. scRNA-seq analysis of human VCLS at day 6, related to Fig. 6.

(**A**) UMAP plot showing the cell clusters in VCLS at day 6, cells are colored by different cell types. (**B**) Dot plot showing the averaged expression levels of indicated marker genes of each cell cluster. (**C**) UMAP plots showing the expression of *SOX2*, *NKX1-2*, *MEOX1*, *CYP1B1*, *MYOCD* and *FN1* in VCLS at day 6. (**D**) UMAP plot showing the cell clusters in CS8-11 monkey embryo, cells are colored by different cell clusters. Two black lines with arrows highlight the potential differential trajectories of NMP cells (to neural lineage or mesodermal lineage) (**E**) UMAP plots showing the expression of *SOX2*, *NKX1-2*, *MEOX1*, *CYP1B1*, *MYOCD* and *FN1* in monkey embryo. (**F**) Heatmap showing the Pearson correlation of related cell clusters between monkey embryo and VCLS.

© The Author(s)
